# Xylose- and Nucleoside-Based Polymers via Thiol–ene Polymerization toward Sugar-Derived Solid Polymer Electrolytes

**DOI:** 10.1021/acsapm.3c02119

**Published:** 2024-01-26

**Authors:** Matthew Oshinowo, Marco Piccini, Gabriele Kociok-Köhn, Frank Marken, Antoine Buchard

**Affiliations:** † Department of Chemistry, University of Bath, Claverton Down, Bath BA2 7AY, U.K.; ‡ Materials and Chemical Characterisation Facility (MC2), University of Bath, Claverton Down, Bath BA2 7AY, U.K.; § University of Bath Institute for Sustainability, Claverton Down, Bath BA2 7AY, U.K.

**Keywords:** sugars, xylose, fatty acids, nucleosides, base-pairing, self-healing, polymer electrolytes, lithium-ion conduction

## Abstract

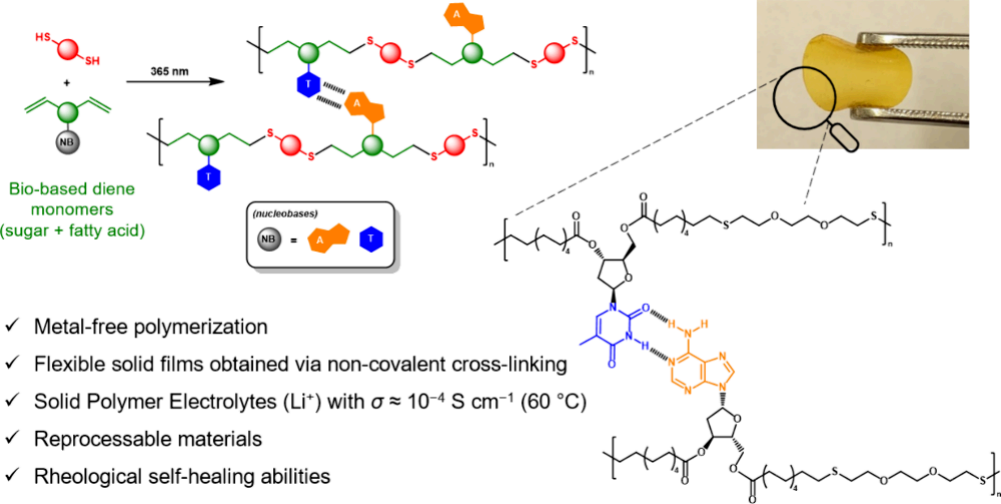

A series of copolymers have been prepared via thiol–ene polymerization of bioderived α,ω-unsaturated diene monomers with dithiols toward application as solid polymer electrolytes (SPEs) for Li^+^-ion conduction. Amorphous polyesters and polyethers with low *T*_g_’s (−31 to −11 °C) were first prepared from xylose-based monomers (with varying lengths of fatty acid moiety) and 2,2′-(ethylenedioxy)diethanethiol (EDT). Cross-linking by incorporation of a trifunctional monomer also produced a series of SPEs with ionic conductivities up to 2.2 × 10^–5^ S cm^–1^ at 60 °C and electrochemical stability up to 5.08 V, a significant improvement over previous xylose-derived materials. Furthermore, a series of copolymers bearing nucleoside moieties were prepared to exploit the complementary base-pairing interaction of nucleobases. Flexible, transparent, and reprocessable SPE films were thus prepared with improved ionic conductivity (up to 1.5 × 10^–4^ S cm^–1^ at 60 °C), hydrolytic degradability, and potential self-healing capabilities.

## Introduction

Lithium-ion batteries (LIBs) are a continually evolving yet well-established technology essential for the transition of society to a more sustainable future. According to a 2019 Global Battery Alliance report for the World Economic Forum, it is predicted that the global demand for LIBs will have increased 14-fold from 184 GWh in 2018 to 2623 GWh by 2030.^1^ This will be primarily driven by our transition to electric vehicles and also by our continually increasing capacity to store renewable energy for the grid. However, the current use of liquid electrolyte components in LIBs poses a myriad of safety and performance issues. Various alternative electrolyte materials are being investigated to address these issues, among which polymer electrolytes are promising contenders. However, while many high-performance polymer electrolytes have been reported in the literature, the majority are derived from fossil fuel feedstocks and therefore do little to address the environmental and sustainability concerns surrounding petrochemically derived polymers.

To this regard, there have been recent examples of polymer electrolytes featuring polysaccharides or carbohydrate-derived synthetic polymers, due to the merits of sugars as renewable polymer building blocks, such as their low cost, high abundance, high oxygen content, and high functionalization potential. For example, in 2022, Chen and co-workers exploited the high functionality of the natural polysaccharide chitosan to form cross-linked organogel polymer electrolyte networks with high room temperature ionic conductivity (1.1 × 10^–3^ S cm^–1^) and lithium-ion transport number (0.82).^2^ In a different approach, we also recently reported an organogel polyether electrolyte derived from d-xylose (a renewable sugar), which was cross-linked with boronic acid groups to achieve even higher room temperature ionic conductivity (3.7 × 10^–3^ S cm^–1^) and lithium transference numbers (*t*_+_ = 0.88–0.92), as well as a wide electrochemical stability window of +4.51 V.^3^

However, solid polymer electrolytes (SPEs) are also promising contenders that offer the potential of improved safety and electrochemical, mechanical, and thermal stability (and thus compatibility with Li metal anodes) relative to liquids and polymer gels. As such, we reported the synthesis and characterization of bioderived cross-linked SPEs from d-xylose and 10-undecenoic acid (derived from castor oil).^4^ The polyester, first reported by our group in 2021,^5^ required cross-linking of the unsaturated C=C bonds in the polymer backbone with 2,2′-(ethylenedioxy)diethanethiol (EDT) to enable film formation with lithium bis(trifluoromethanesulfonyl)imide (LiTFSI). As a proof-of-concept, this study reported ionic conductivity in the region of 10^–5^ S cm^–1^ at 60 °C with a high lithium transference number (*t*_+_) of 0.84.

However, sustainability concerns with the use of the ruthenium-based Grubbs catalyst required for the acyclic diene metathesis (ADMET) polymerization method were noted. This was exacerbated by the fact that the synthesis of low molar mass polymers (beneficial for ionic conductivity) required higher catalyst loadings. Therefore, removing the need for this catalytic method had next been desired.

**Scheme 1:**
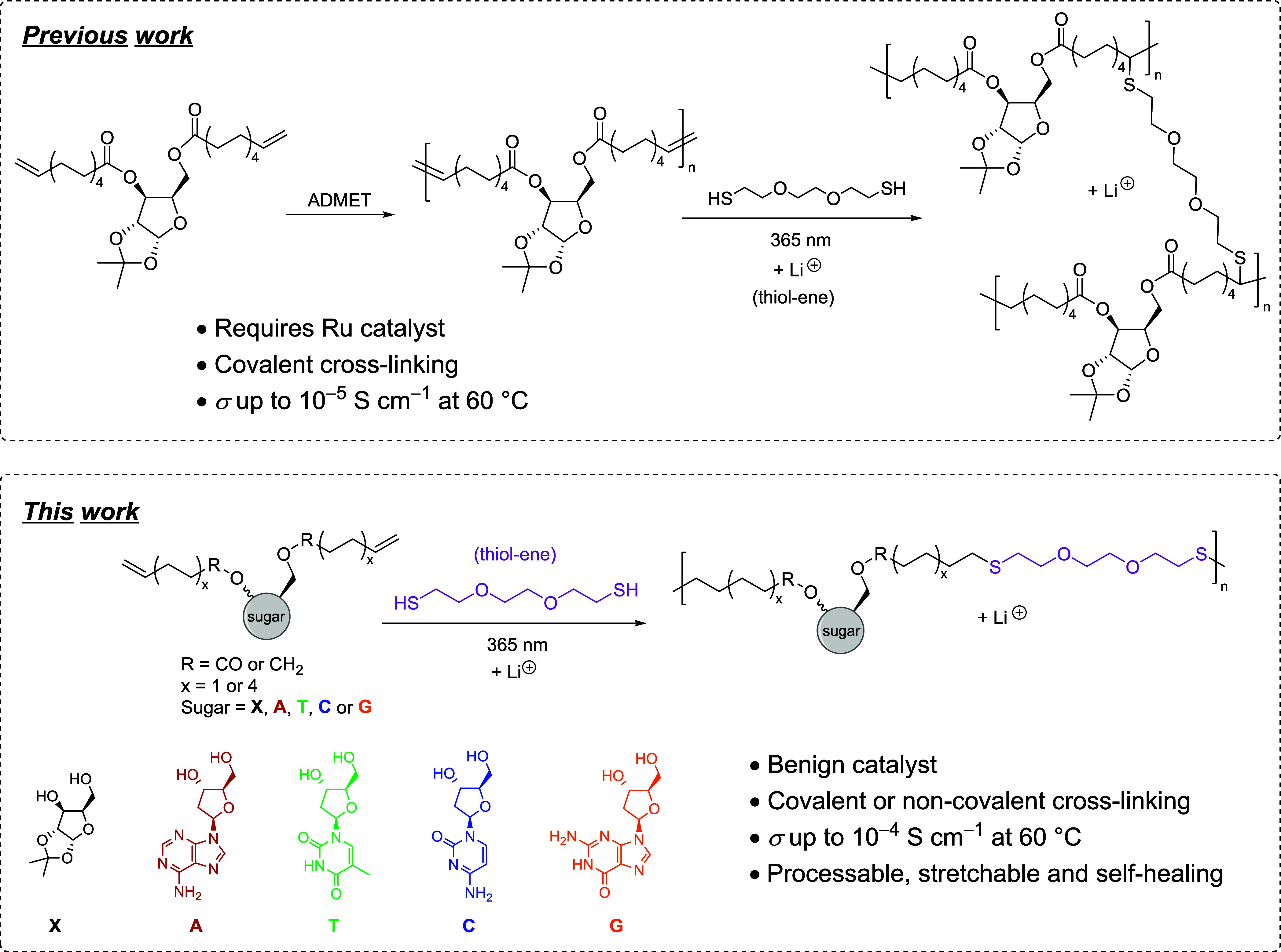
Comparison of the Work Previously Reported versus the Work Carried out for This Study

Previously, thiol–ene “click” chemistry was used as a method of cross-linking the polyester with EDT (Scheme 1). The thiol–ene reaction is a well-known alkene hydrothiolation reaction with many green credentials and has frequently been used for the postpolymerization modification of polymers via the functionalization of alkenes and alkynes.^6^ Although less known, the thiol–ene reaction can also be used to copolymerize dienes and dialkynes with dithiols. Examples of elastomers, albeit often from activated/electron-poor dienes where Michael addition can proceed, have been published by Reineke and team^7−9^ and more recently by Dove and co-workers.^10−12^

To avoid the use of Ru-catalyzed ADMET polymerization, herein, we report the direct copolymerization of α,ω-unsaturated sugar-based diene monomers with EDT using the thiol–ene reaction as an alternative and more sustainable polymerization method. Due to the viscous liquid-like nature of the resulting alternating copolymers at room temperature, several methods of altering the mechanical properties to enable film formation for SPE applications have been investigated in this work, including covalent cross-linking (using a trithiol), introduction of crystallinity (the replacement of EDT with 1,8-octanedithiol), and complementary base-pairing (moving from xylose-based diene to nucleoside-based diene).

Nucleosides feature nucleobases attached to a 5-membered deoxyribose sugar ring analogous to xylose, and it was expected that we could incorporate these moieties into our polymer platform while maintaining its biosourced merits. The base-pairing interaction between complementary nucleobases within DNA is well-known, and we postulated that a similar interaction could be incorporated into our polymers providing rigidity and potentially self-healing abilities via dynamic, noncovalent cross-linking in the form of hydrogen-bonding without hindering ionic conductivity (which can be the case for covalent cross-linking).

Polymers functionalized with nucleobases^13^ or structurally similar synthetic base-pairing moieties featuring several H-bonding arrays (*e.g.*, ureidopyrimidinone (UPy) units), known as “Janus bases”, have been used to prepare a range of self-assembling and/or self-healing supramolecular networks.^14−17^ For example, Bowman and co-workers reported an impressive proof-of-concept platform for the synthesis of sequence-controlled DNA-like polymers.^18^ The base-pairing interaction of a DNA template with the nucleoside-based thiolactone monomers was envisioned to yield a complementary sequence-defined polymer via ring-opening polymerization.

In the context of Li-metal batteries, Xue and co-workers reported polymer electrolytes employing UPy units to impart impressive self-healing abilities to the materials with ionic conductivities up to 1.4 × 10^–5^ S cm^–1^ at 60 °C.^19−21^ In 2020, Wang and co-workers reported a self-healing polymer electrolyte with a room temperature ionic conductivity >10^–3^ S cm^–1^ featuring UPy moieties to bridge the poor interfacial contact between the electrodes and a Li_1.5_Al_0.5_Ge_1.5_P_3_O_12_ solid electrolyte.^22^ The same year, they also reported a high-performance self-healing SPE, which incorporated UPy Janus bases to form a polymer matrix capable of hosting a deep eutectic solvent.^23^

However, to the best of our knowledge, biosourced nucleosides have not been directly incorporated into polymer electrolytes for lithium-ion batteries. This may be due to the limited number of polymerization methods that are tolerant of the nucleobase moieties. For example, previously, we reported the need to protect the thymine moiety of a thymidine-derived cyclic carbonate toward ring-opening polymerization.^24^ Moreover, unsurprisingly, we have also found these nucleobase moieties to be incompatible with ADMET polymerization. To this regard, herein we report the first example of biosourced nucleosides directly incorporated into polymer electrolytes for lithium-ion batteries through thiol–ene polymerization. Dynamic cross-linking has been investigated as a method to improve processability, solubility, and ionic conductivity compared to covalently cross-linked analogues, as well as for the implementation of potential self-healing abilities.

## Results and Discussion

### Xylose-Based Alternating Copolymers and Characterization

**Scheme 2:**
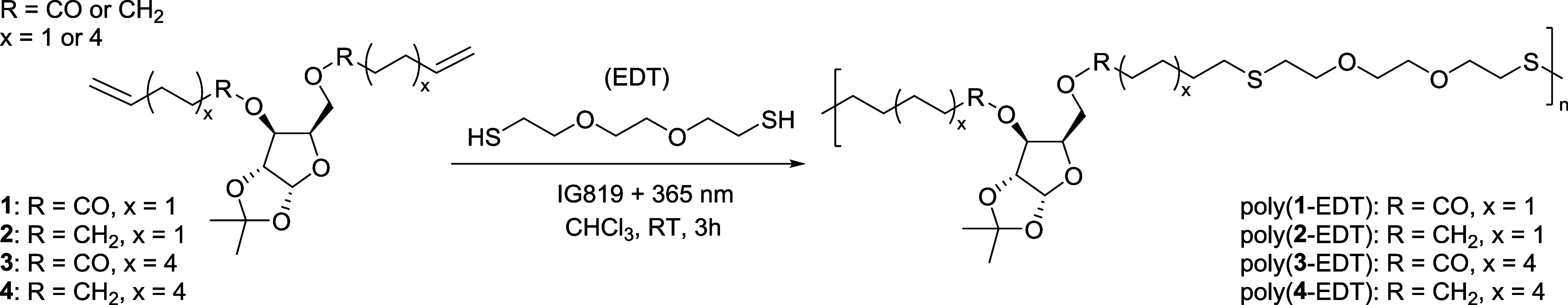
Preparation of Xylose-Based Alternating Copolymers Prepared in This Study

In the initial part of the study, four α,ω-unsaturated xylose-based diene monomers (**1**–**4**), featuring ester (from 10-undecenoic or 4-pentenoic acid) or ether (from 10-undecenol or 4-pentenol) functions, were prepared according to previously reported procedures (Scheme 2). Four corresponding alternating copolymers based on these diene monomers with EDT were prepared in a simple procedure; the comonomers were combined in a stoichiometric 1:1 ratio in CHCl_3_ or THF with a small amount of photoinitiator (IG819) and irradiated for 3 h at ambient temperature in air. Precipitation from an antisolvent, followed by centrifugation, rinsing, and drying, afforded the copolymers as viscous yellow materials (Table 1).

**Table 1:** Selected Data for the Thiol–ene Alternating Copolymerization of Xylose-Based Monomers **1**–**4** with EDT^a^

entry	monomer	functional group^b^	alkyl chain length	polymer nomenclature	*M*_n,SEC_ [*Đ*_M_]^c^ (kg mol^–1^)	*T*_g_ (°C)^d^	*T*_m_ (°C)^d^
1	**1**	ester	C5	poly(**1**-EDT)	6.4 [2.9]	–14	
2	**2**	ether	C5	poly(**2**-EDT)	13.6 [2.3]	–25	
3	**3**	ester	C11	poly(**3**-EDT)	12.3 [5.4]	–34	–11
4^e^	**3**	ester	C11	poly(**3**-EDT)	6.9 [2.8]	–35	–13
5	**4**	ether	C11	poly(**4**-EDT)	10.8 [3.4]	–31	–3
6^f^	**4**	ether	C11	poly(**4**-EDT)	17.1 [2.4]	–32	–6
7^g^	**4**	ether	C11	poly(**4**-EDT)	15.5 [4.1]	–30	–4

a Reaction conditions (unless otherwise stated): 1 equiv of xylose-based monomer, 1 equiv of EDT, 0.1 equiv of IG819, CHCl_3_ (0.5 mol L^–1^ w.r.t. xylose-based monomer), UV irradiation (*λ* = 365 nm), 3 h.

b Refers to the functional group connecting the sugar core with the alkyl chains of the fatty acid/alcohol.

c Calculated by SEC methods and performed in THF using polystyrene calibration standards (*Đ*_M_ = *M*_W_/*M*_n_).

d Taken from the second heating cycle between −60 and +200 °C in the DSC thermogram.

e 1.2 equiv of monomer **3** was used.

f EDT was distilled prior to use.

g Reaction left for 24 h.

Quantitative conversion was observed in all cases, as determined by the disappearance of the olefin signals in the ^1^H NMR spectrum of a crude sample prior to precipitation. The ^1^H NMR spectra of the isolated products were fully assigned, with all polymers exhibiting characteristic triplet signals around 2.5 and 2.7 ppm from the methylene protons surrounding the newly formed thioether bonds (Supporting Information, Figures S6.15, S6.17, S6.21 and S6.28). Assignment of all ^13^C{^1^H} NMR spectra was aided by 2D HSQC and HMBC NMR experiments of poly(**2**-EDT) and poly(**3**-EDT) (Supporting Information, Figures S6.18–6.20 and S6.22–6.24).

Molar masses of 6.4–13.6 kg mol^–1^ were achieved, which, while ideal for SPE applications where lower molar mass is favorable to ion mobility, may be the reason for the viscous nature of the polymers. Although this step-growth method of polymerization offers little control over the molar mass, using pure reagents can help to increase the molar mass, since the stoichiometry will be closer to the ideal 1:1 ratio. For example, sugar-derived isosorbide and isomannide polyurethanes of *M*_n_ > 50 kg mol^–1^ (and even >100 kg mol^–1^) have been reported by Dove and co-workers using the thiol–ene copolymerization method.^11^ In this case, distillation of the commercial sample of EDT increased the molar mass of poly(**4**-EDT) from 10.8 to 17.1 kg mol^–1^ but no significant effect on the thermal properties was noted (Table 1, entries 5 and 6). Moreover, increasing the reaction time to 24 h resulted in an increase in molar mass to 15.5 kg mol^–1^.

Analysis of the thermal properties of the copolymers by differential scanning calorimetry (DSC, Figure S9.1) showed that the copolymers of **1** and **2** with EDT were amorphous materials with low glass transition temperatures (*T*_g_’s) of −25 and −14 °C, respectively, therefore accounting for their sticky, viscous nature. The copolymers of **3** and **4** with EDT were found to be semicrystalline, although their melting temperatures (*T*_m_’s) of −11 and −3 °C, respectively, were well below room temperature and also accounted for their sticky, viscous nature. Therefore, it was clear that it would not be possible to prepare robust, self-standing SPE films from these copolymers without modification to improve their mechanical properties.

### Covalent Cross-Linking

Similarly to our previous work in this area,^4^ cross-linking was investigated as an approach for obtaining films based on this copolymer platform. Incorporation of a trifunctional thiol, trimethylolpropane tris(3-mercaptopropionate) (TMP), into the EDT monomer feed enabled *in situ* cross-linking as the polymerization proceeded. In an initial test, the mole fraction of TMP in EDT required to impart sufficient film-forming capabilities was investigated. It was found that a mixture of EDT and TMP, with a molar ratio of 8:2 or less, resulted in the precipitation of a cross-linked material during the polymerization of **1** (see the Supporting Information, Table S2), despite taking the reaction away from the ideal 1:1 SH:alkene stoichiometry and likely limiting the obtention of high molar masses.

Due to the insolubility of the cross-linked polymers at this EDT:TMP ratio, SPE films were prepared via the *in situ* polymerization of the comonomers in a procedure analogous to that previously reported by our group for the preparation of cross-linked SPE films.^4^ All comonomers were dissolved in anhydrous THF with IG819 and LiTFSI, and the solution was cast into a flat dish. After evaporation of the solvent, the dish was irradiated for 3 h, which resulted in polymerization and simultaneous incorporation of Li^+^ ions into the polymer matrix. Thin, yellow, transparent, cross-linked SPE films were thus obtained (Supporting Information, Figure S4.1).

Cross-linked SPEs based on monomers **1**–**4** were initially prepared using a EDT:TMP ratio of 8:2 and a LiTFSI content of 50 mol % (**SPEs 1**–**4x**, Table 2). The ionic conductivity (σ) at 60 °C was found to be the highest for **SPE-3a** (9.0 × 10^–6^ S cm^–1^), with a trend showing higher conductivity for esters vs ethers and for C11 chains vs C5 chains (Figure S11.1). The former trend was attributed to the absence of carbonyl groups (known to be significant Li^+^ ion coordinators)^25^ in the ether-based polymers and the latter trend to the lower *T*_g_ and therefore greater chain mobility imparted by the longer alkyl groups.

**Table 2:**
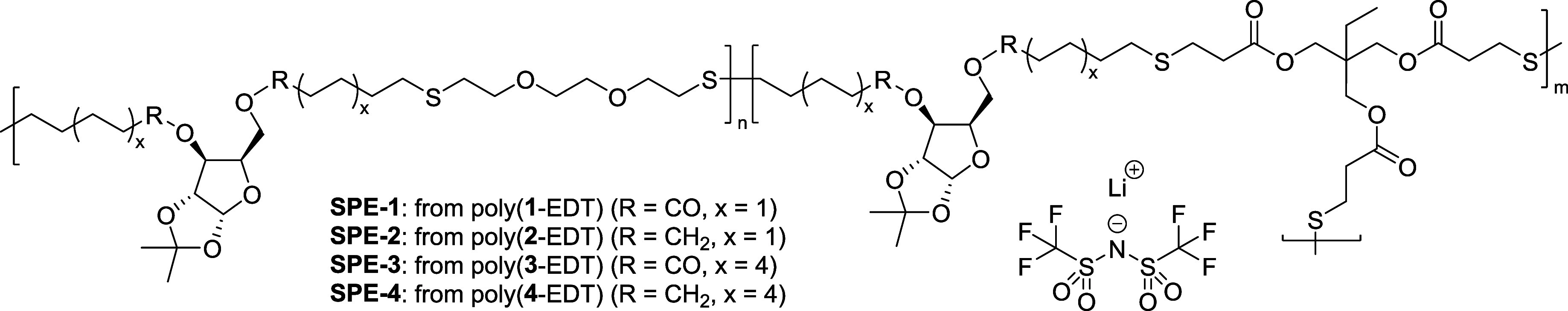
Selected Data for the Preparation of Covalently Cross-Linked Xylose-Based SPE Films^a^

entry	SPE	monomer	EDT:TMP ratio	Li^+^ amount (mol %)^b^[wt %]	*T*_g_ (°C)^c^	σ^d^(× 10^–5^ S cm^–1^)
1	**SPE-1**	**1**	8:2	50 [19]	–5	0.7
2	**SPE-2**	**2**	8:2	50 [21]	0	0.3
3	**SPE-3a**	**3**	8:2	50 [15]	–13	0.9
4	**SPE-3b**	**3**	8:2	25 [8]	–17	0.3
5	**SPE-3c**	**3**	8:2	100 [26]	–2	1.0
6	**SPE-3d**	**3**	9:1	100 [27]	–15	2.2
7	**SPE-3e**	**3**	9:1	150 [36]	–11	1.5
8	**SPE-4**	**4**	8:2	50 [16]	–24	0.5

a SPEs were prepared via solvent casting of a mixture of monomer **1**–**4**, EDT and TMP (one molar equivalent in total), IG819 (10 mol %) and LiTFSI in THF, followed by irradiation for 3 h at room temperature.

b Refers to the molarity of LiTFSI compared to the polymer repeat unit.

c Taken from the second heating cycle between −60 and +200 °C in the DSC thermogram.

d Measured at 60 °C.

Monomer **3** was therefore used for further optimization of the ionic conductivity by varying the salt loading. Similarly to our report of cross-linked ADMET polyesters,^4^ it was found that increasing the salt loading to 100 mol % resulted in an enhanced ionic conductivity of 1.0 × 10^–5^ S cm^–1^ recorded at 60 °C for **SPE-3c** (Figure S11.2). This highlights the importance of providing additional charge carriers despite the increased rigidity imparted by the salt, as demonstrated by the increase in *T*_g_ from −13 to −2 °C.

To counteract this, we then decreased the cross-linking density by increasing the EDT:TMP ratio to 9:1 for **SPE-3d**, which yielded a maximum conductivity of 2.2 × 10^–5^ S cm^–1^ for this system. However, further increasing the salt loading to 150 mol % for **SPE-3e** did not impart higher ionic conductivity. The ionic conductivity of **SPE-3d** at 60 °C (2.2 × 10^–5^ S cm^–1^) is approximately double that of the best performing ADMET SPE from our previous work (*ca.* 1.0 × 10^–5^ S cm^–1^) at the same temperature.^4^ While the thiol–ene polymerization method is a simpler method of preparing SPEs that eliminates the use of a Grubbs catalyst, the cross-linked materials still lack the processability desired for their application and study due to their insolubility imparted by the covalently cross-linked matrix.

Further electrochemical characterization analysis was then performed on **SPE-3d**. Linear sweep voltammetry was performed to determine that the material exhibits good stability (vs Li/Li^+^) with very low currents with applied voltages below 5 V (Supporting Information, Figure S11.2). A sharp spike in current was only observed at 5.08 V. This result is promising for practical applications in LIBs (which require electrochemical stability ≥4.2 V) and notably higher than our previously reported ADMET SPE (+3.88 V).^4^ The transference number (*t*_+_) was also determined using the Bruce–Vincent method.^26^ The polarization profile of the plot in Figure S13.1 (Supporting Information) exhibited an expected gradual decline in current until a steady state was reached after approximately 20 min. The bulk resistance increased slightly after polarization; however, a good transference number of 0.42 was obtained, which is significantly higher than archetypal PEO-based SPEs where *t*_+_ ≈ 0.08.^27^

### Introduction of Additional Crystallinity

Introducing crystallinity into polymers is an alternative approach to incorporating mechanical strength/integrity. While the C11 copolymer analogues were semicrystalline, they existed in the melted state at room temperature. However, further crystallinity could be incorporated by replacing EDT with 1,8-octanedithiol (ODT), in which the ether oxygens are replaced with CH_2_ units. Poly(**3**-ODT) was synthesized as a semicrystalline copolymer of **3** with ODT, which was a solid at room temperature with a *T*_m_ of 43 °C and a *T*_g_ of 7 °C (Table 3).

**Table 3:** Selected Data for the Preparation of Copolymers of Monomer **3** with EDT and ODT^a^

entry	dithiol	polymer nomenclature	*M*_n,SEC_ [*Đ*_M_]^b^(kg mol^–1^)	*T*_g_ (°C)^c^	*T*_m_ (°C)^c^
1^d^	ODT	poly(**3**-ODT)	12.5 [2.7]	5	41
2	EDT & ODT	poly(**3**-EDT-*co*-ODT)	17.4 [2.6]	–32	21
3	EDT & ODT (EDT block preformed)	poly((**3**-EDT)-*b*-(**3**-ODT))	15.7 [1.9]	–35	–13, 39
4	EDT & ODT (both blocks preformed)	poly((**3**-EDT)-*bb*-(**3**-ODT))	9.4 [1.9]	–29, 5	41

a Reaction conditions (unless otherwise stated): 1 equiv of monomer **3**, 1 equiv of dithiol, 0.1 equiv of IG819, CHCl_3_ (0.5 mol L^–1^ w.r.t. monomer **3**), UV irradiation (*λ* = 365 nm), 3 h.

b Calculated by SEC methods and performed in THF using polystyrene calibration standards (*Đ*_M_ = *M*_W_/*M*_n_).

c Taken from the second heating cycle between −60 and +200 °C in the DSC thermogram.

d 1.2 equiv of EDT was used.

Upon addition of 50 mol % LiTFSI, the resulting material turned dark brown and became almost insoluble after thorough drying. Nevertheless, the polymer was plasticized and had become fully amorphous with a *T*_g_ of −25 °C. The film obtained after hot-pressing exhibited poor ionic conductivity as low as 2 × 10^–8^ S cm^–1^ at 60 °C which, when compared to that of poly(**3**-EDT), was attributed to the removal of two significant Li^+^ ion coordinating sites per repeat unit. As such, there are long segments in the polymer backbone with little functionality able to solvate the ions, thus demonstrating the crucial importance of the presence of Li^+^-coordinating oxygen atoms in the polymer backbone.

To combat this, three approaches were taken to prepare copolymers of the three monomers. First, poly(**3**-EDT-*co*-ODT) was prepared by the direct copolymerization of **3** with EDT and ODT in a 2:1:1 ratio (Table 3, entry 2). The resulting polymer possessed a single *T*_m_ at 21 °C and the absence of a lower *T*_m_ for **3**-EDT segments. Presumably, the significant melting point depression and absence of a lower *T*_m_ originated from the short **3**-ODT and **3**-EDT segments due to the random nature of the copolymerization. This polymer also lost all crystallinity and therefore material properties upon incorporation of LiTFSI.

**Figure 1:**
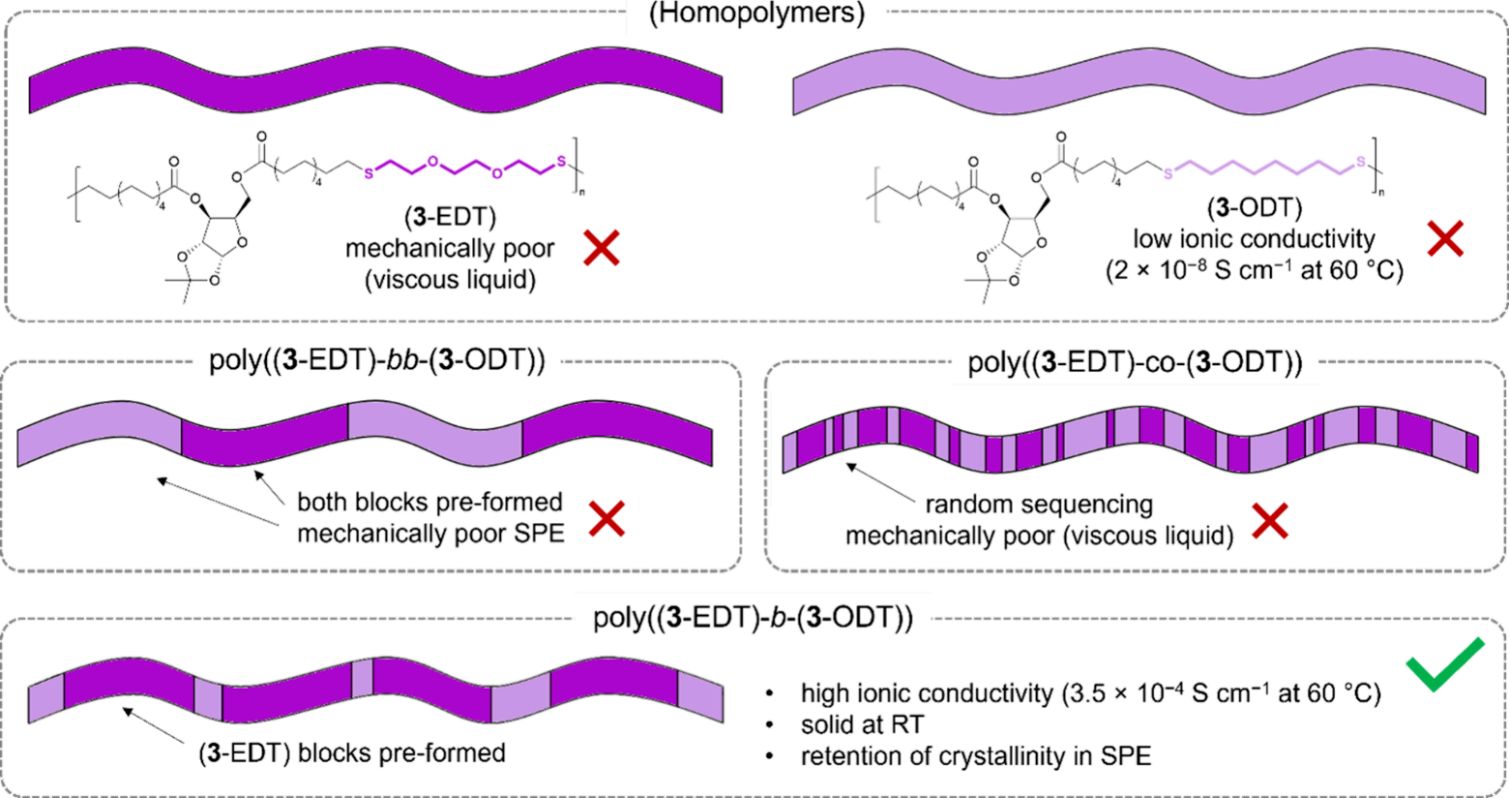
Three approaches to copolymers based on **3**, EDT, and ODT.

Therefore, block copolymers were prepared in the hope that the **3**-EDT blocks would contribute ionic conductivity and the **3**-ODT blocks would contribute mechanical strength (see Figure 1). First, a **3**-EDT prepolymer block was prepared with an excess of EDT and omission of ethyl vinyl ether as an end-capping terminator to ensure polymer chains end-capped with SH groups. Once isolated, the prepolymer was then added to a second polymerization containing additional monomer **3** and an equimolar amount of ODT to obtain poly((**3**-EDT)-*b*-(**3**-ODT)) (Table 3, entry 3).

As expected, poly((**3**-EDT)-*b*-(**3**-ODT)) was a solid at room temperature. The successful chain extension and formation of a block copolymer were supported by SEC analysis (Supporting Information, Figure S14.1), which showed monomodal peaks for the prepolymer and the final polymer as well as a doubling of the molar mass from 7.3 to 15.7 kg mol^–1^. DSC analysis revealed a retention of the thermal transitions for the individual copolymers with a *T*_g_ and *T*_m_ of −35 and −13 °C for the **3**-EDT block, respectively, and a *T*_m_ of 39 °C for the **3**-ODT block. The *T*_m_ of the **3**-ODT block in poly((**3**-EDT)-*b*-(**3**-ODT)), similar to that of poly(**3**-ODT) (41 °C), suggested that a significant block length was achieved. ^1^H diffusion-ordered NMR spectroscopy (DOSY NMR) was also used to confirm the presence of a single species diffusing at the same rate in solution (Supporting Information, Figure S6.27A, diffusion coefficient = 2.0 × 10^–10^ m^2^ s^–1^). To confirm that the species was a new polymer, the experiment was repeated after spiking the NMR tube with a small amount of the **3**-EDT prepolymer block and the resulting spectrum indicated the presence of two different species (Supporting Information, Figure S6.27C).

Poly((**3**-EDT)-*b*-(**3**-ODT)) was mixed with LiTFSI (*ca.* 100 mol %) for SPE preparation, which resulted in contrasting effects on the thermal transitions. On one hand, the *T*_g_ was increased to −26 °C, as expected. The *T*_m_ of the **3**-EDT block was slightly decreased to −15 °C, and the *T*_m_ of the **3**-ODT was significantly decreased to −28 °C (Supporting Information, Figure S9.16). The retention of some crystallinity enabled the resulting material to form a more solid material than poly(**3**-EDT) alone at room temperature, although it still lacked sufficient mechanical strength to form a self-standing film. Moreover, retention of a low melting temperature meant that the material experiences melting during the EIS experiment, which is not practical for SPE applications. Nevertheless, a high ionic conductivity of 3.5 × 10^–4^ S cm^–1^ was recorded at 60 °C.

In the final approach, poly((**3**-EDT)-*bb*-(**3**-ODT)) was prepared by linking preformed blocks of poly(**3**-EDT) and poly(**3**-ODT) with terminal SH and C=C end groups, respectively, by using an excess of one comonomer (Table 3, entry 4). The two separate blocks were then combined in a third thiol–ene reaction, which yielded a single species (Supporting Information, Figure S6.27B, diffusion coefficient = 1.9 × 10^–10^ m^2^ s^–1^). Due to the **3**-ODT block being incorporated as a preformed unit, the *T*_m_ observed remained at 41 °C. Surprisingly, this polymer also lost its crystallinity and therefore material properties upon incorporation of LiTFSI.

### Dynamic (Noncovalent) Cross-Linking Using Nucleosides

Noncovalent cross-linking via base-pairing H-bonding interactions was investigated as an alternative strategy for imparting mechanical properties while retaining solubility, processability, and ability to dissolve Li salts. This was achieved by replacing the xylose moieties in the monomers with nucleosides sourced from DNA-based molecules. These sugars, including deoxythymidine (dThd), deoxyadenosine (dAdo), deoxycytidine (dCyd), and deoxyguanosine (dGuo), consist of a nucleobase attached to 2-deoxyribose.

**Figure 2:**
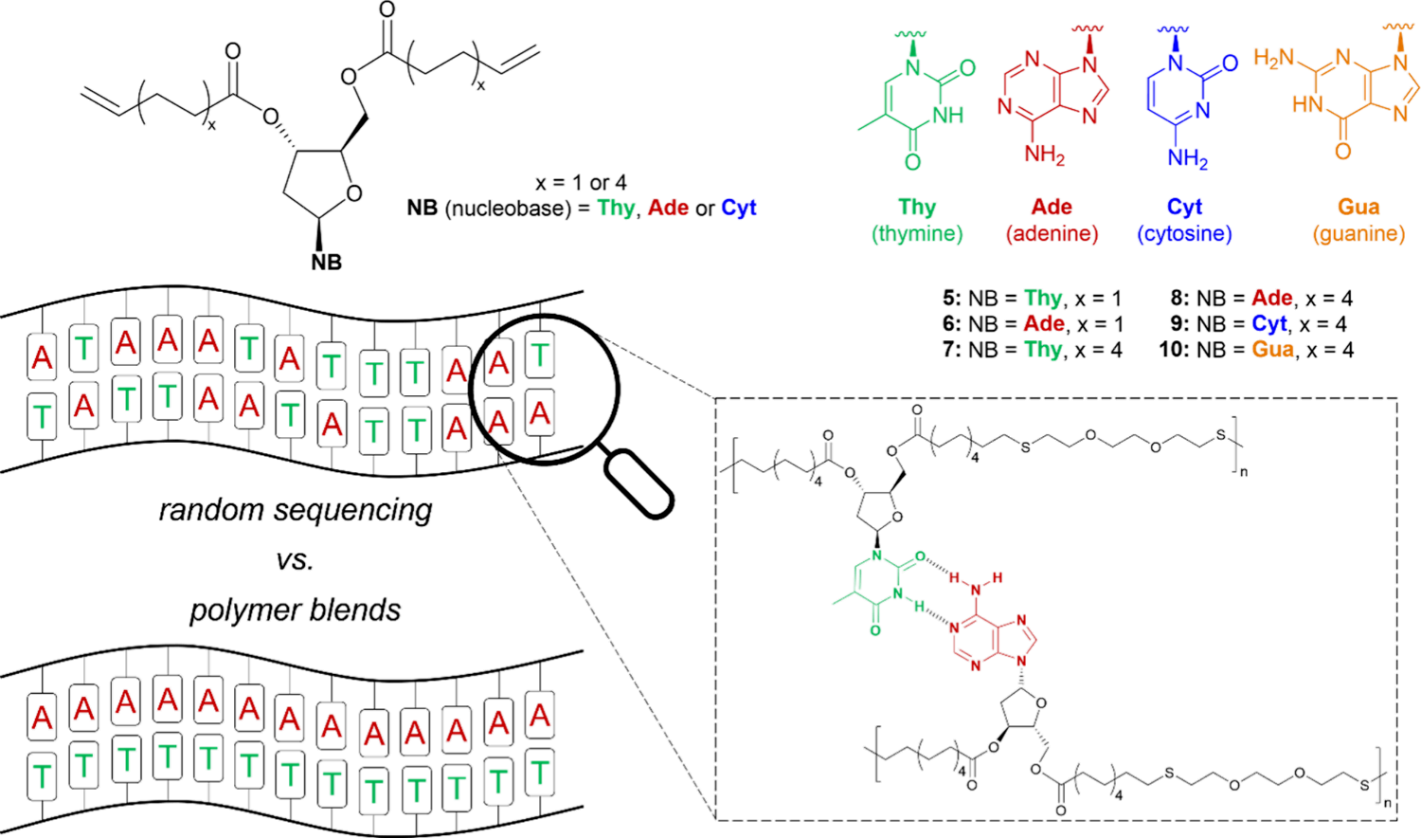
Chemical structures of the nucleoside-based diene monomers prepared in this study and the two alternative approaches to base pairing that were employed.

Ester monomers **5**–**8** (Figure 2) were readily prepared, without the need for any protection strategies, via coupling of 10-undecenoic anhydride and 4-pentenoic anhydride with dThd and dAdo using the same procedure reported for monomers **1** and **3**. Column chromatography afforded the monomers in good yields and purity (determined by ^1^H NMR spectroscopy, see the Supporting Information, Figures S6.5–S6.12), and their structures were confirmed by mass spectrometry. Whereas **5** and **7** were oils, **8** was a white waxy solid and **6** was a crystalline white solid with a crystal structure (Supporting Information, Figure S2.1), as confirmed by X-ray diffraction (XRD). Monomer **9** (from dCyd and 10-undecenoic anhydride) was also successfully synthesized and characterized. However, the selective synthesis of its complementary monomer **10** from dGuo was unsuccessful, likely due to the commercial availability of the sugar in the monohydrate form, which may be incompatible with the esterification coupling reaction.

The copolymers of **5**–**9** with EDT were synthesized using the same procedure for monomers **1**–**4**, all achieving quantitative conversion (Table 4) as determined by ^1^H NMR spectroscopic analysis. Once dried, the Ade- and Cyt-based copolymers were insoluble in common laboratory solvents, the latter possibly due to some cross-linking of the cytosine units by EDT via the C=C bond. This was not observed for any of the thymine-based copolymers; however, this may be due to the decreased reactivity of the trisubstituted alkene of the thymine units vs the disubstituted alkene in the cytosine. The insolubility of poly(**6**-EDT) and poly(**8**-EDT) may be due to the strong π–π interactions of the adenine groups. However, the Thy-based polymers retained solubility once dried, and their ^1^H NMR spectra (Supporting Information, Figures S6.30 and S6.32) exhibited the same characteristic triplet signals as the xylose-based copolymers around 2.5 and 2.7 ppm, indicative of the newly formed thioether bonds. The polymers exhibited a range of *T*_g_ values, but all appeared to be “gummy” in nature in contrast to the “sticky” nature of the xylose-based copolymers.

**Table 4:** Selected Data for the Thiol–ene Alternating Copolymerization of Nucleoside-Based Monomers **5**–**9** with EDT^a^

entry	monomer	sugar^b^	alkyl chain length	polymer nomenclature	*M*_n,SEC_ [*Đ*_M_]^c^(kg mol^–1^)	*T*_g_ (°C)^d^	*T*_m_ (°C)^d^
1	**5**	dThd	C5	poly(**5**-EDT)	3.7 [2.7]	3	
2	**6**	dAdo	C5	poly(**6**-EDT)	2.5 [2.0]	–29	
3	**5**:**6** (1:1)	dThd/dAdo	C5	poly(**5**/**6**-EDT)	4.6 [2.0]	–11	
4	**7**	dThd	C11	poly(**7**-EDT)	14.7 [2.6]	–7	
5	**8**	dAdo	C11	poly(**8**-EDT)	Insol.	–34	45, 66
6	7:8 (1:1)	dThd/dAdo	C11	poly(**7**/**8**-EDT)	23.4 [2.9]	–36	–2
7	**9**	dCyd	C11	poly(**9**-EDT)	insol.	–37	

a Reaction conditions (unless otherwise stated): 1 equiv of nucleoside monomer, 1 equiv of dithiol, 0.1 equiv of IG819, CHCl_3_ (0.5 mol L^–1^ w.r.t. nucleoside monomer), UV irradiation (*λ* = 365 nm), 3 h.

b dThd = deoxythymidine, dAdo = deoxyadenosine, dCyd = deoxycytidine.

c Calculated by SEC methods and performed in THF using polystyrene calibration standards (*Đ*_M_ = *M*_W_/*M*_n_).

d Taken from the second heating cycle between −60 and +200 °C in the DSC thermogram.

Two approaches were taken to realize the H-bonding interaction of the complementary nucleobases: (1) blending of the complementary copolymers and (2) copolymerization using a mixture of the complementary monomers (Figure 2). Interestingly, blends of the complementary polymers resulted in a merging of the *T*_g_’s. For example, poly(**5**-EDT) and poly(**6**-EDT) (Table 4, entries 1 and 2, *T*_g_’s = 3 and −29 °C, respectively) yielded a polymer blend with a *T*_g_ of −17 °C.

For poly(**7**-EDT) and poly(**8**-EDT) (Table 4, entries 4 and 5, *T*_g_’s = −7 and −34 °C, respectively), an amorphous polymer blend with a *T*_g_ of −32 °C was obtained. Unfortunately, incorporation of LiTFSI into the polymer blends did not result in robust SPE films but instead yielded materials that remained gummy and were impossible to redissolve, thus suggesting that the H-bonding interaction in these materials was too strong.

In comparison, the polymers obtained by the second method of using a 1:1 feed of the complementary monomers (Table 4, entries 3 and 6) exhibited *T*_g_’s that were comparable to their corresponding blends, yet they retained solubility. Moreover, it was possible to prepare SPE films via solution casting of the 1:1 monomer feeds with EDT and LiTFSI, followed by irradiation and *in situ* polymerization. It was possible to redissolve the resulting films in THF as well as to reprocess them using hot-pressing equipment while maintaining transparency (Supporting Information, Figure S4.1), therefore demonstrating the largely improved processability of the films obtained via this method.

**Figure 3:**
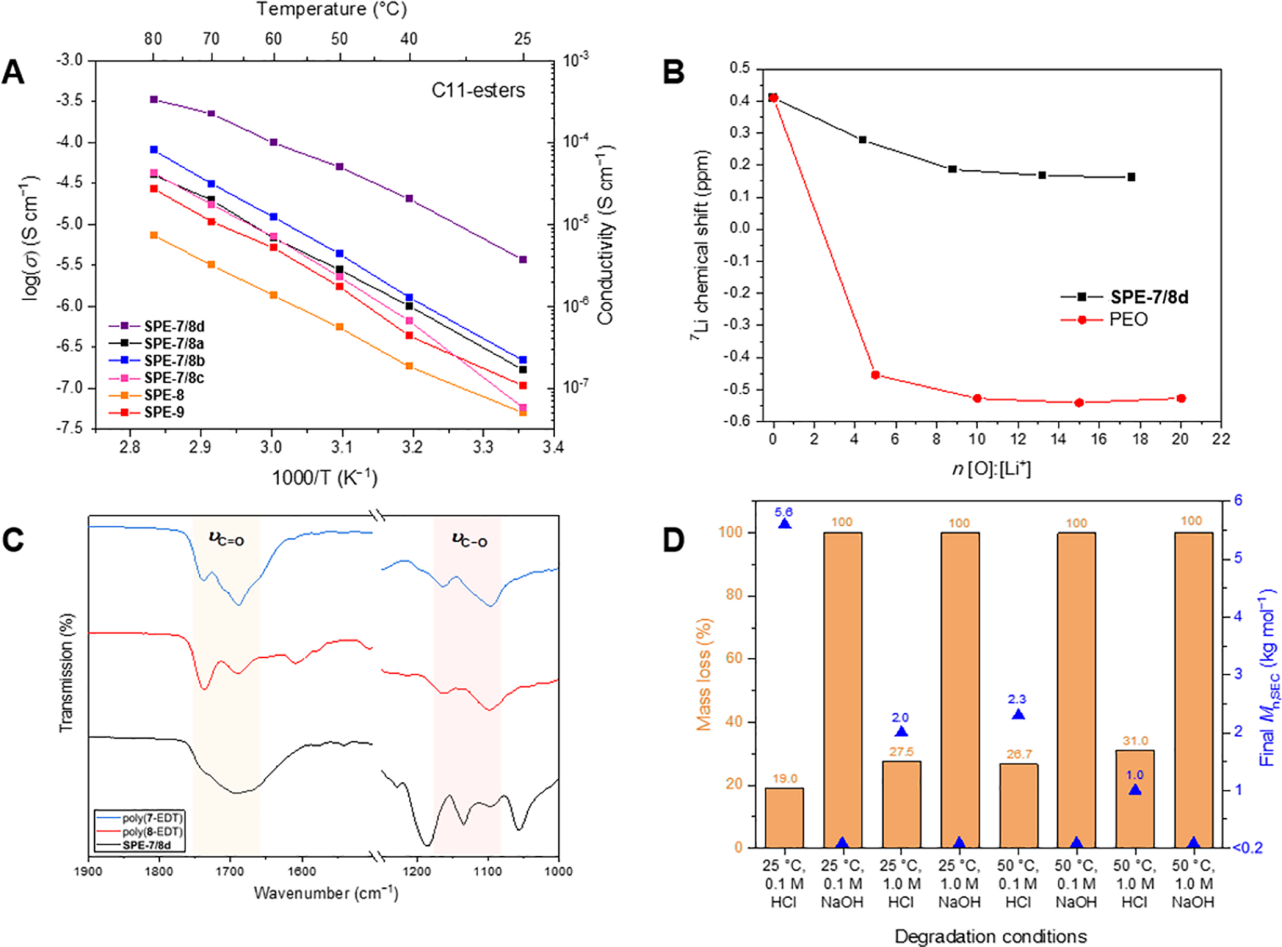
(A) Chart displaying the temperature dependence of the ionic conductivity of selected nucleoside-based films. (B) ^7^Li NMR titration in THF of a **SPE-7/8d** and a commercial PEO sample (100 kg mol^–1^) to determine the solvation strength of the polymers (referenced to a LiCl_(aq)_ solution). (C) Stacked FTIR spectra of selected polymers focusing on the regions of interest. (D) Hydrolytic degradability of **SPE-7/8d** under various conditions.

When optimizing the amount of LiTFSI for SPEs based on a 1:1 ratio of **7**:**8**, the ionic conductivity at 60 °C was found to reach a maximum of 1.2 × 10^–5^ S cm^–1^ for **SPE-7/8b** with a salt loading of 100 mol % (38 wt %) (Table 5 and Figure 3A). Interestingly, this optimal salt loading (in terms of wt % relative to polymer molar mass) is very similar to that of the cross-linked xylose-based SPEs reported in both our previous publication (41 wt %)^4^ and in the previous section of this report (40 wt %). This suggests that for all of the structurally similar polymers reported thus far, there is an optimal amount of LiTFSI equating to approximately 40 wt %, which yields the highest performance.

**Table 5:** Selected Data for the Preparation of Nucleoside-Based Films^a^

entry	SPE	monomer(s)	dithiol(s)	Li^+^(mol %)^b^ [wt %]	*T*_g_ (°C)^c^	*T*_m_ (°C)^c^	σ^d^ (× 10^–5^ S cm^–1^)
1	**SPE-5/6**	**5**:**6** (1:1)	EDT	100 [31]	8	—	0.04
2	**SPE-7**	**7**	EDT	100 [26]	7	—	—e
3	**SPE-8**	**8**	EDT	100 [26]	–18	35, 70	0.1
4	**SPE-9**	**9**	EDT	100 [27]	–20	22, 42	0.4
5	**SPE-7/8a**	**7**:**8** (1:1)	EDT	50 [15]	–17	—	0.7
6	**SPE-7/8b**	**7**:**8** (1:1)	EDT	100 [26]	–7	—	1.2
7	**SPE-7/8c**	**7**:**8** (1:1)	EDT	200 [42]	–26	—	0.7
8	**SPE-7/8d**	**7**:**8** (9:1)	EDT	100 [26]	–9	—	9.8
9	**SPE-7/8e**	**7**:**8** (95:5)	EDT	100 [26]	0	—	15.0
10	**SPE-7/8f**	**7**:**8** (9:1)	EDT:TMP (9:1)	100 [26]	5	—	0.2
11	**SPE-7/8g**	**7**:**8** (9:1)	EDT:TMP (95:5)	100 [26]	4	—	0.6^f^

a SPEs were prepared via solvent casting of a mixture of monomer(s) **5–9,** thiol(s) (one molar equivalent in total), IG819 (10 mol %) and LiTFSI in THF, followed by irradiation for 3 h at room temperature.

b Refers to the molarity of LiTFSI compared to the polymer repeat unit.

c Taken from the second heating cycle between −60 and +200 °C in the DSC thermogram.

d Measured at 60 °C.

e — means that the SPE film was unable to form.

f Measured at 80 °C.

It was also possible to improve the ionic conductivity of the SPE by changing the **7**:**8** ratio. Increasing the ratio to 9:1 or 95:5 in SPEs with 100 mol % LiTFSI (**SPEs 7/8b, 7/8d** and **7/8e**) offered significant improvements in ionic conductivity, as high as 1.5 × 10^–4^ S cm^–1^ for **SPE-7/8e** at 60 °C (Figure 3A). This trend in conductivity was expected due to the lower degree of H-bonding in SPEs with larger **7**:**8** ratios, which rendered them more viscous than solid. Notably, **SPE-7/8e** was easily deformed and remained slightly sticky at room temperature even after thorough drying and, as such, was not studied further despite its high conductivitys. **SPE-7/8b** and **SPE-7/8d** were selected for further studies due to their high ionic conductivity, mechanical robustness, and preferable processability compared to the other approaches described in earlier sections.

The solvation strength of **SPE-7/8d** was compared to that of PEO via a ^7^Li NMR titration measuring the change in ^7^Li chemical shift at 0.4 ppm in THF (Figure 3B).^28^ The results indicate that **SPE-7/8d** is a weaker coordinator of Li^+^ ions compared to a commercial sample of PEO (100 kg mol^–1^) as demonstrated by the much smaller change in chemical shift upon addition of polymer. Since the plateau of chemical shift is much lower for PEO *vs***SPE-7/8d** (−0.5 ppm vs 0.2 ppm, respectively), PEO remains the stronger solvator even when the chart in Figure 3B is adjusted to include other heteroatoms that could solvate Li^+^ ions are present (i.e., S and N). The lower coordination strength would suggest an enhanced ionic conductivity compared to an SPE prepared with the commercial PEO (100 kg mol^–1^, LiTFSI = 8 mol %, *T*_g_ = −36 °C, *T*_m_ = 44 °C, *σ* = 2.0 × 10^–4^ S cm^–1^ at 60 °C, Figure S11.4), but since this is not the case, there must be other factors at play. It is likely that the H-bonding network induced by the nucleobases plays a much larger role on Li^+^ ion solvation in the solid film state than is observed in solution.

The polymer–cation interaction was then studied by FTIR analysis. The FTIR spectrum of **SPE-7/8d** shows a broad peak at 1690 cm^–1^ due to the stretching vibrations of the C=O groups from the ester and thymine functionalities (Figure 3C). The peak occurs at a much lower wavenumber compared to those in the pure polymer (around 1737 cm^–1^), therefore highlighting the strong interaction of the Li^+^ ions with the C=O bonds. Moreover, the broadness of the peak supports the dynamic nature of the C=O···Li^+^ interactions with the many ester bonds in the SPE, which may be interrupting some of the base-pairing interactions present in the pure polymer. In the C–O stretching region, there appears to be no change in the frequency of the C–O bond in the ribose unit 1098 cm^–1^. There may well be a slight change in the frequency of the ester C–O bond around 1161 cm^–1^, although the strong peaks attributed to LiTFSI at 1185, 1134, and 1057 cm^–1^ dominate the region.

The Li^+^ stripping and plating behavior of **SPE-7/8d** in a symmetric Li||SPE||Li cell were then investigated. However, short-circuiting of the cell was observed, as indicated by the extremely small voltage response to an applied current of 0.1 mA cm^–2^ (Supporting Information, Figure S15.1). This was attributed to the dynamic nature of the cross-links which can be broken at elevated temperatures, thus resulting in a more liquid-like material which is not capable of withstanding the stack pressure of the electrochemical cell. The two lithium electrodes may then come close enough to touch either directly or via dendrites that form across the electrolyte. The gradual decrease in resistance over the first 10 cycles suggests the progressive formation of dendrites. However, even at lower temperatures, the ±1.0 V set cutoff limit was reached immediately after starting the measurement because of the high cell resistance resulting from the low ionic conductivity of **SPE-7/8d** at these temperatures. Clearly, these materials require a balance between mechanical integrity and ionic conductivity to serve as a functioning SPE, a finding which is in accordance with ionic conductivity studies previously reported.^4^

It was therefore postulated that the incorporation of a small amount of covalent cross-linking would provide enough mechanical integrity to prevent the issue of short-circuiting. Indeed, literature reports have found that covalent cross-linking can offer increased protection against short-circuiting due to impact or suppression of lithium dendrite growth, for example, by Coates, Archer and co-workers in 2014 with cross-linked SPEs based on PEO and polyethylene.^29^**SPE-7/8f** and **SPE-7/8g** were thus prepared (Table 5, entries 10 and 11) with a small degree of covalent cross-linking by incorporating small amounts of TMP. Although the ionic conductivity was sacrificed (*e.g.*, 1.5 × 10^–5^ S cm^–1^ at 80 °C for **SPE-7/8f**), a cycling behavior without short-circuiting was then observed, albeit with poor performance at 60 and 80 °C with a low current density of 0.025 mA cm^–2^ (Supporting Information, Figure S15.2). Although no short-circuiting was also observed for **SPE-7/8g** prepared with only 5 mol % TMP, the cycling performance at 80 °C and 0.025 mA cm^–2^ (Supporting Information, Figure S15.3) was equally poor. Due to the inability of these materials to operate within a reasonable voltage window in a symmetric lithium cell, their investigation with active cathode materials was not explored.

### Rheology and Self-Healing

The rheological properties of **SPE-7/8b** (**7**:**8** = 1:1) and **SPE-7/8d** (**7**:**8** = 9:1) were then investigated to gain some insight into the dynamic nature of the H-bonding. Frequency sweep experiments at 1–100 Hz revealed contrasting results for the two materials (Figure S16.1). For **SPE-7/8b**, the storage modulus (*G*′), representing the rigid elastic component of the complex modulus (*G*), was greater than the loss modulus (*G*″), representing the inelastic viscous component of *G*, across all measured oscillatory frequencies. This implies that **SPE-7/8b** is an elastic material. For **SPE-7/8d**, *G*′ only becomes greater than *G*′′ after a crossover frequency around 4 Hz, thus implying that **SPE-7/8d** behaves as a rheological viscoelastic gel.^30^ Such frequency-dependent moduli highlight the dynamic nature of the cross-links for **SPE-7/8d**, as seen in the literature for other viscoelastic organogel materials with dynamic cross-links. For example, our group recently published a report on biobased organogels with dynamic boronate ester bonds based on d-xylose, which were also demonstrated to function as gel polymer electrolytes.^3^

The same contrasting behavior was observed in temperature sweep experiments (30-80 °C; Figure S16.2). **SPE-7/8b** was found to behave as an elastic material across the whole temperature range whereas **SPE-7/8d** was found to exhibit a crossover temperature at 71 °C, after which *G*′′ overtook *G*′, suggesting a transition to a viscous material. This is supported by a small *T*_g_ occurring in the first heating cycle of the DSC experiment at 68 °C. The difference in rheological behavior of the two materials is not unexpected given the lower degree of H-bonding and therefore higher viscosity of **SPE-7/8d** compared to **SPE-7/8b**. Moreover, **SPE-7/8b** was more mechanically robust than **SPE-7/8d** as shown by the larger magnitude of the moduli across both experiments.

The dynamic nature of the cross-links in this polymer system suggested that the materials may display self-healing capabilities. In the context of polymers, several good reviews have covered the many approaches taken to allow the reformation of cross-linked polymer networks after tearing.^31−34^ Polymer networks featuring dynamic H-bonding have also been heavily investigated in this regard.^35^

Quantitative analysis of the rheological self-healing capabilities was investigated. First, a single strain ramp measurement was performed at 60 °C with a 1 Hz oscillatory frequency (Supporting Information, Figures S16.3 and S16.4). This allowed the determination of the critical strain required to break the dynamic cross-links and force the material into a viscous liquid-like state, which was identified by a sharp drop in the moduli. For **SPE-7/8b** and **SPE-7/8d**, this was found to be *ca.* 4% and 7%, respectively. After the strain was further increased to 100%, both materials exhibited severe drops in their moduli by at least an order of magnitude (*ca.* 95–98%). Upon release of the strain back to 0.007%, both materials recovered 100% of their original moduli after only 90 s. These results therefore demonstrate that the materials can rapidly and fully reform their broken cross-links at 60 °C.

The dynamic nature of the cross-linking, which could result in rheological self-healing abilities, was further investigated via sequential step strain measurements at fixed temperature (60 °C) and oscillating frequency (1 Hz). In this experiment, the strain was sequentially increased from 0% to 10%, 20%, 40%, 60%, 80%, and 100% with a hold period of 60 s followed by a 90 s rest and then a release back to 0.007% strain for each elevated strain (Supporting Information, Figure S16.5). Though the data is somewhat erratic at certain strains, which may be due to the rigid, elastic nature of the materials at that temperature and frequency, the experiment succeeded in further demonstrating the recovery of the storage modulus and the self-healing capabilities of the materials in a relatively short time frame.

Finally, the hydrolytic degradability of these materials was demonstrated on **SPE-7/8d** in various conditions over 72 h. Incubation of the films in 1.0 mol L^–1^ NaOH_(aq)_ solutions resulted in complete disintegration of the films within 2 h at 25 and 50 °C or 24 h for 0.1 mol L^–1^ NaOH_(aq)_ solutions at 25 and 50 °C (Figure 3D). In all four cases, no polymeric material was detected in the dry residue (as determined by SEC). The mass loss observed after degradation in HCl was much lower (19–31%). While some of this may be due to removal of LiTFSI from the SPE via dissolution into the aqueous solution, SEC analysis of the remaining residue did reveal significant reduction in molar mass and multimodal peaks.

## Conclusions

This report has described the preparation and characterization of a class of synthetic carbohydrate polymers for SPE applications derived from natural d-xylose and nucleosides. In contrast to our previous work where EDT was employed as a cross-linker for unsaturated xylose-based polymers made by ADMET, EDT was here employed as a comonomer for the preparation of copolymers with bioderived α,ω-unsaturated diene monomers. The thiol–ene polymerization method is considered a more sustainable polymerization method (compared to ADMET) for this application due to the simpler and less energy intensive conditions required in addition to a reduced number of steps, and therefore represents an advance on previously reported sugar-based materials and methods.

Thiol–ene copolymers of xylose-based monomers **1**–**4** (C5/C11, ester/ether variants) and EDT were prepared and found to exhibit very similar material properties as their ADMET counterparts. Importantly, their amorphous, viscous natures with low *T*_g_'s (−31 to −11 °C) did not yield the desired material properties for SPE applications. Covalent cross-linking was therefore investigated to impart mechanical integrity, and several SPE materials were prepared, with a maximum ionic conductivity of 2.2 × 10^–5^ S cm^–1^ obtained at 60 °C for **SPE-3d**, which also displayed an impressive electrochemical stability of 5.08 V and a transference number of 0.42. Copolymers were also prepared by incorporating ODT into the monomer feed to introduce additional crystallinity and enhance mechanical integrity.

Noncovalent cross-linking in the form of complementary H-bonding base-pairing interactions of nucleobases was also investigated. Diene monomers **5**–**9** bearing nucleoside moieties were prepared and were copolymerized with EDT. Flexible and transparent SPE films based on these copolymers were obtained. They retained solubility in organic solvents and could be reprocessed via hot-pressing, which was hailed as a significant advantage over previous covalently cross-linked materials. The dynamic nature of the base-pairing interactions resulted in self-healing and viscoelastic properties as determined by rheological measurements, where the ability of the materials to fully recover their moduli after only 90 s at elevated strain levels was demonstrated. Moreover, the ionic conductivity was also improved by a factor of 10 to 1.5 × 10^–4^ S cm^–1^ at 60 °C for **SPE-7/8e**, which is greater than other self-healing polymer electrolytes dynamically cross-linked with UPy units reported in the literature (*e.g.*, Xue and co-workers in 2021, *σ* = 1.4 × 10^–5^ S cm^–1^ at 60 °C).^19^

## Supporting Information Available

Experimental methods and synthetic procedures, NMR and FTIR spectra, DSC and TGA traces, SEC analysis, representative Nyquist plots, and additional electrochemistry data (PDF)
Crystallographic data for monomer **6**: CCDC 2234496 (CIF)

## Supplementary Material

ap3c02119_si_001.pdf

ap3c02119_si_002.cif

## ABBREVIATIONS

*σ*: ionic conductivity
*t*_+_: transference number
ADMET: acyclic diene metathesis
dAdo: deoxyadenosine
dCyd: deoxycytidine
dGuo: deoxyguanosine
dThd: thymidine
DSC: differential scanning calorimetry
EDT: 2,2′-(ethylenedioxy)diethanethiol
EIS: electrochemical impedance spectroscopy
IG819: phenyl bis(2,4,6-trimethylbenzoyl)phosphine oxide
LiTFSI: lithium bis(trifluoromethanesulfonyl)imide
SPE: solid polymer electrolyte
