# Supplementary material for: Xylose- and Nucleoside-Based Polymers via Thiol–ene Polymerization toward Sugar-Derived Solid Polymer Electrolytes
Source: ACS Appl Polym Mater. 2024 Jan 26;6(3):1622–32. doi: 10.1021/acsapm.3c02119 (PMC10862469; doi:10.1021/acsapm.3c02119)
Supplement: Supplementary file 1 — ap3c02119_si_001.pdf [file ap3c02119_si_001.pdf]

Supporting Information

Xylose- and Nucleoside-Based Polymers via Thiol-ene Polymerization toward Sugar-derived Solid Polymer Electrolytes

*Matthew Oshinowo,[a,c] Marco Piccini,[a,c] Gabriele Kociok-Köhn,[b] Frank Marken,[a,c] and Antoine Buchard[a,c]**

[a] Department of Chemistry, University of Bath, Claverton Down, Bath, BA2 7AY, UK.

[b] Materials and Chemical Characterisation Facility (MC2), University of Bath, Claverton Down, Bath, BA2 7AY, UK.

[c] University of Bath Institute for Sustainability, Claverton Down, Bath, BA2 7AY, UK.

Email: A.Buchard@bath.ac.uk

Contents

1. Experimental Procedures S2
2. Monomer Synthesis S5
3. Co-polymer Synthesis S13
4. SPE Preparation Procedures S19
5. Determination of TMP mole fraction for covalently cross-linked xylose-based SPE films S21
6. NMR Spectra S22
7. FTIR Spectra S39
8. Mass Spectrometry S48
9. Differential scanning calorimetry (DSC) S53
10. Thermogravimetric analysis (TGA) S63
11. Electrochemical impedance spectroscopy (EIS) S71
12. Linear Sweep Voltammetry (LSV) S73
13. Transference Number S74
14. Size Exclusion Chromatography (SEC) S75
15. Electrochemical Stripping/Plating Experiments S77
16. Rheological Self-Healing Experiments S79
17. References S82

1. Experimental Procedures

1.1 Materials and methods

All reagents were purchased from Sigma-Aldrich or Fisher Scientific without additional purification except from LiTFSI which was dried at 110 °C in a vacuum oven for 24 hours before being stored in an argon-filled glovebox to prevent absorption of moisture. IPXF and deoxy-nucleosides were purchased from Carbosynth. PEO (100 kg mol^{-1}) was purchased from Alfa Aesar (Fisher Scientific). Lithium iron phosphate (LFP) cathode material coated on aluminium foil was purchased from Cambridge Energy Solutions and was also stored in an argon-filled glovebox. Unless otherwise stated, solvents for monomer synthesis and thiol-ene co-polymerization in solution were supplied by VWR and used without purification. Anhydrous solvents from Thermo Scientific were used for SPE preparation. All chemicals were stored at room temperature except from ethyl vinyl ether which was stored in a fridge at 3 °C. Thiol-ene reactions were performed in a PhotoRedox TC light box by HepatoChem with a 30 W UV lamp (λ = 365 nm). Silica gel was used as the stationary phase for column chromatography and plates were visualised with phosphomolybdic acid (10 wt% in ethanol) staining solution.

1.2 Characterisation methods

Nuclear magnetic resonance (NMR) spectra were recorded on a 500 MHz Bruker NMR spectrometer and referenced to residual proton or ^{13}C peaks of the $CDCl_3$ solvent. Spectra were processed and analysed using Mnova software by Mestrelab.

X-Ray Diffraction (XRD) crystallographic data was collected on a SuperNova EOS detector diffractometer using radiation Cu-Kα (λ= 1.54184 Å) or Mo-Kα (λ = 0.71073 Å) radiation, all recorded at 150K. All structures were solved by direct methods and refined on all F2 data. All hydrogen atoms were included in ideal positions and refined using the riding model.

Mass spectrometry (MS) analysis was performed using an Agilent QTOF 6545 with Jetstream ESI spray source coupled to an Agilent 1260 Infinity II Quat pump HPLC with 1260 autosampler, variable wavelength detector and column oven compartment. The MS was operated in positive mode with the drying gas, the gas temperature and the nebulizer gas at 12 L min^{-1}, 250 °C and 45 psi (3.10 bar), respectively. The sheath gas flow and temperature were set to 12 L min^{-1} and 350 °C, respectively. The MS was calibrated using reference calibrant from the independent ESI reference sprayer. The VCap, Skimmer and Fragmentor was set to 3500, 45 and 125 respectively. Samples were prepared in acetonitrile with a concentration of 10 μg ml^{-1}.

Differential scanning calorimetry (DSC) was used to measure the glass transition temperatures (T_g) of samples on a TA Instruments DSC Q20 employing the Q Series program. The

experiment was performed under nitrogen gas (flow rate = 18 mL min^{-1}) and samples were heated and cooled at a rate of 10 °C min^{-1} in a 10 µL Tzero aluminium pan with lid. The T_g was taken from the second heating cycle between −60 °C and +200 °C.

Size exclusion chromatography (SEC) was performed with a 1260 GPC/SEC MDS system from Agilent. Separation was achieved using two PLgel 5 µm MIXED-D 300 × 7.5 mm columns with a PLgel 5 µm MIXED 50 × 7.5 mm guard column. SEC-grade THF was used as the mobile phase and refractive index (RI) was used as a detection method. The columns and RI detector were all maintained at 35 °C. The system was calibrated using polystyrene standards in THF which allowed the determination of the number-average molar mass ($M_{n,SEC}$) and dispersities ($Đ_M$) of polymer samples.

Fourier-transform infrared spectra (FTIR) were recorded on a Spectrum Two FT-IR Spectrometer by Perkin Elmer in the range of 450−4000 cm^{-1} and processed using Perkin Elmer Spectrum IR software. Peaks are described by their appearance (s = sharp, m = medium, w = weak, br = broad).

Thermogravimetric analysis (TGA) was performed using the Calisto program on a Setaram Setsys Evolution TGA 16/18. The analytical chamber was purged with argon (200 mL min^{-1}) for 40 minutes prior to heating under an argon flow (20 mL min^{-1}) from 30 °C to 600 °C with a ramp of 10 °C min^{-1}. $T_{d,5\%}$ refers to the temperature at which 5 % mass loss has been reached and $T_{d(max)}$ refers to the temperature of the peak of the mass loss derivative.

Electrochemical measurements were performed using a TCS battery cell (RHD instruments) with blocking stainless steel current collectors connected to a Metrohm Autolab PGSTAT204 potentiostat with a FRA32M module. The sample and cell components were dried in a vacuum oven at 70 °C prior to cell assembly inside an argon-filled glovebox. Temperature control of the cell was achieved using a Microcell HC stand (RHD instruments) containing a peltier element for active heating and cooling of the measuring cell.

Ionic conductivity (*σ*) was determined by a two-electrode electrochemical impedance spectroscopy (EIS) measurement in the typical frequency range of 0.1 Hz–0.5 MHz with an applied amplitude of 50 mV in a symmetrical SS||SPE||SS cell. The samples were annealed to the electrode surfaces for 16 hours at 80 °C and then 5 repeat EIS measurements recorded at each temperature with a one hour temperature equilibration between different temperatures. NOVA 2.1 (Metrohm) software was used to analyse the results and apply a Randles equivalent circuit fitting to the obtained Nyquist plot to obtain the bulk resistance (R_b). R_b was used to calculate σ using the equation:

$$\sigma = \left(\frac{l}{A}\right) \times \left(\frac{1}{R_\mathrm{b}}\right)$$

where l = film thickness (typically 200−500 μm measured by digital callipers) and A = electrode surface area (0.503 cm^2).

Lithium transference number (t_+) was determined using a combined EIS and chronoamperometry method using a Li||SPE|Li symmetrical cell using lithium foil of 0.75 mm thickness and an applied voltage of 10 mV. The measurement was recorded at 80 °C and t_+ calculated using the Bruce-Vincent equation:[3]

$$t_+ = \frac{I_{SS}(\Delta V - I_0 R_{b,0})}{I_0(\Delta V - I_{SS} R_{b,SS})}$$

where ΔV is the applied voltage and I_0 and I_{SS} represent the initial and steady state current before and after DC polarisation, respectively. $R_{b,0}$ and $R_{b,SS}$ represent the bulk resistance obtained from EIS measurements before and after DC polarisation, respectively. The measurement was taken five times and t_+ reported as an average with standard error.

Linear sweep voltammetry was used to determine the electrochemical stability in a Li||SPE||SS cell using a lithium counter/reference electrode at 50 °C with a scan rate of 1 mV s^{-1}.

Galvanostatic cycling (Li stripping/plating) experiments were performed in a symmetric Li||SPE||Li cell at 80 °C with one hour half cycle durations. The applied current was determined by multiplying the desired current density (in mA cm^{-2}) by the exposed surface area of the lithium electrodes (0.503 cm^2). The cells were typically pre-conditioned with three full cycles with a current density of 0.025 mA cm^{-2} prior to starting the stripping/plating cycles.

Rheological measurements were conducted using a Discovery HR-2 hybrid rheometer from TA Instruments with a 25 mm parallel plate geometry. A temperature of 25 °C was maintained during frequency sweep experiments which were conducted at 0.007 % strain. An oscillating frequency of 1 Hz was maintained during temperature sweep experiments. Oscillatory strain amplitude measurements for the investigation of self-healing behaviour were conducted at a frequency of 1 Hz at 60 °C with 90 second rest periods at the end of each elevated strain segment.

2. Monomer Synthesis

2.1 Synthesis of xylose-based α,ω-unsaturated ester monomers 1 & 3

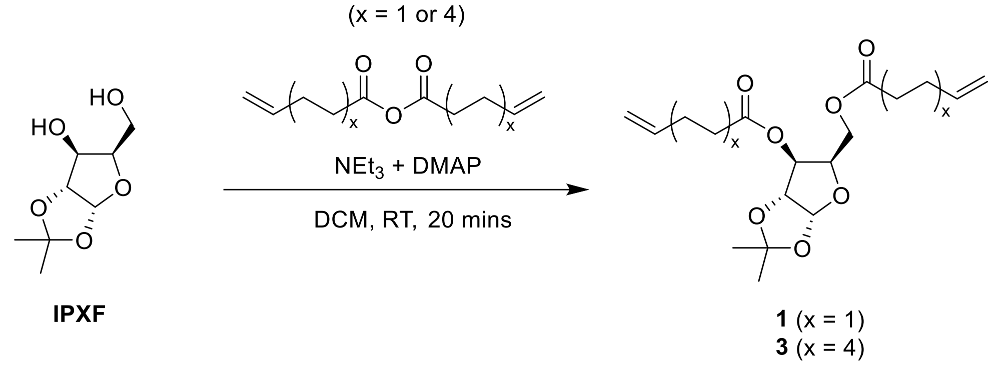

4-Pentenoic and 10-undecenoic anhydride precursors were prepared according to previously reported literature procedures by our group.[1,2] 1,2-*O*-Isopropylidene-α-D-xylofuranose (IPXF) (1 equiv.), triethylamine (3.0 equivs.) and the desired ω-unsaturated anhydride (2.5 equivs.) were dissolved in DCM in a round-bottom flask, followed by portion-wise addition of 4-dimethylaminopyridine (DMAP) (0.3 equivs.). After 20 minutes, Amberlyst A-26(OH) ion-exchange resin (500−1000 wt% *w.r.t.* IPXF) was added and the suspension stirred for 1 hour. The mixture was filtered and the resin washed with additional DCM. The solvent was removed from the filtrate *in vacuo* to yield a crude product which was purified by silica chromatography.

2.1.1 Monomer 1

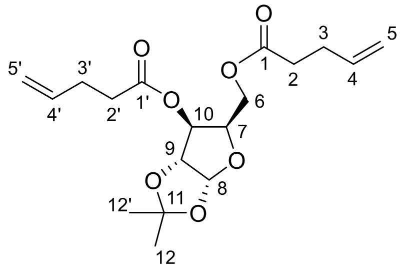

(1)

Column eluent = 5−15% ethyl acetate in petroleum spirit. Appearance = yellow oil. **Yield** = 3.90 g (96 %). **^{1}H NMR** (400 MHz, $CDCl_3$): *δ* 5.91 (1H, d, *J* = 3.8 Hz, H-8), 5.86–5.69 (2H, m, H-4, H-4'), 5.25 (1H, d, *J* = 3.2 Hz, H-10), 5.10–4.95 (4H, m, H-5, H-5'), 4.53–4.44 (2H, m, H-7, H-9), 4.30–4.16 (2H, m, H-6), 2.43 (4H, q, *J* = 6.5 Hz, H-2, H-2'), 2.40–2.30 (4H, m, H-3, H-3'), 1.51 (3H, s, H-12), 1.30 (3H, s, H-12') ppm; **^{13}C{^{1}H}** (126 MHz, $CDCl_3$) *δ* 172.7 (C-1), 171.8 (C-1’), 136.6 (C-4), 136.2 (C-4’), 116.0 (C-5), 115.7 (C-5’), 112.4 (C-11), 105.0 (C-8), 83.5 (C-9), 76.9 (C-7), 76.1 (C-10), 61.4 (C-6), 33.3 (C-3, C-3’), 28.8 (C-2, C-2’), 26.8 (C-12’), 26.3 (C-12’) ppm. NMR spectroscopic data is in agreement with literature values.[1]

2.1.2 Monomer 3

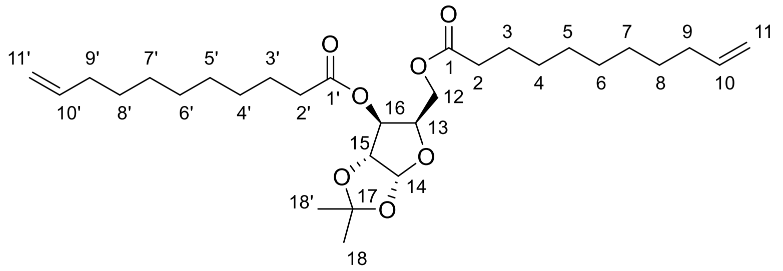

(3)

Column eluent = 1−3% ethyl acetate in petroleum spirit. Appearance = colourless oil. **Yield** = 6.32 g, 60 % .**^{1}H NMR** (400 MHz, $CDCl_3$): *δ* 5.90 (1H, d, *J* = 3.7 Hz, H-14), 5.77 (2H, m, H-10, H-10'), 5.23 (1H, d, *J* = 3.1 Hz, H-16), 4.98–4.86 (4H, m, H-11, H-11'), 4.48–4.45 (2H, m, H-13, H-15), 4.25–4.13 (2H, m, H-12), 2.33–2.25 (4H, m, H-10, H-10'), 2.03–1.97 (4H, m, H-9, H-9'), 1.62–1.53 (4H, m, H-3, H-3'), 1.49 (3H, s, H-18'), 1.37–1.22 (24H, m, H-4–H-8, H-4'–H-8', H-18) ppm; **^{13}C{^{1}H}** (126 MHz, $CDCl_3$) *δ* 173.4 (C-6), 172.4 (C-6'), 139.2 (C-10, C-10'), 114.2 (C-11, C-11'), 112.3 (C-17), 105.0 (C-14), 83.5 (C-15), 76.9 (C-13), 75.9 (C-16), 61.2 (C-12), 34.1 (C-2, C-2'), 33.8 (C-9, C-9'), 29.3−28.9 (C-4−C-8, C-4'−C-8'), 26.8 (C-18'), 26.3 (C-18), 24.9 (C-3, C-3') ppm. NMR data is in agreement with literature values.[2]

2.2 Synthesis of xylose-based α,ω-unsaturated ether monomers 2 & 4

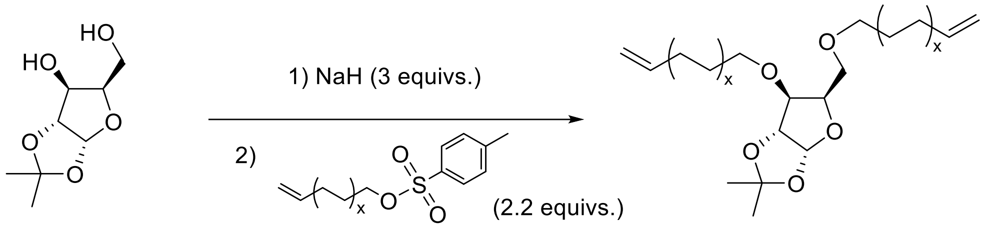

IPXF

2 (x = 1)
4 (x = 4)

4-Pentenyl *p*-toluenesulfonate and 10-undecenyl *p*-toluenesulfonate precursors were prepared according to previously reported literature procedures by our group.[1] NaH (0.75 g, 31.4 mmols, 3 equivs.) was measured out into an oven-dried Schlenk flask inside a glovebox. Anhydrous DMF (25 mL) was added and the suspension stirred for 30 minutes at 0 °C and then for 30 minutes at room temperature. The flask was re-cooled to 0 °C before dropwise addition of a solution of 4-pentenyl *p*-toluenesulfonate or 10-undecenyl *p*-toluenesulfonate (23.0 mmols, 2.2 equivs.) in anhydrous DMF (10 mL). The flask was allowed to warm to room temperature and left stirring overnight. The resulting brown mixture was carefully quenched with saturated NH_4Cl solution (50 mL), followed by water (50 mL). The product was extracted from the aqueous layer with ethyl acetate (3 × 150 mL) and the combined organic layer dried over $MgSO_4$. Filtration and removal of the solvent *in vacuo* afforded an orange oil which was purified by column chromatography (0−5% MeOH in DCM) to yield the diene diether monomer as a clear, yellow oil.

2.2.1 Monomer 2

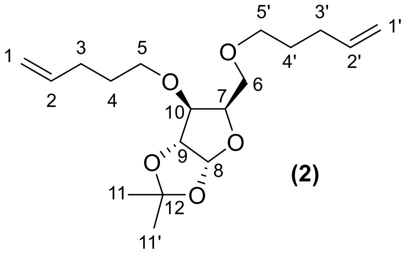

(2)

Yield = 55%. ^{1}**H NMR** (400 MHz, $CDCl_3$): δ 5.89 (1H d, J = 3.9 Hz, H-8), 5.87–5.73 (2H, m, H-2, H2'), 5.05–4.93 (4H, m, H-1, H-1'), 4.52 (1H, d, J = 3.9 Hz, H-9), 4.36–4.31 (1H, m, H-7), 3.81 (d, J = 3.1 Hz, H-10), 3.71–3.39 (6H, m, H-5, H-5', H-6), 2.15–2.05 (4H, m, H-3, H-3'), 1.72–1.61 (4H, m, H-4, H-4'), 1.49 (3H, s, H-11'), 1.31 (3H, s, H-11) ppm; ^{13}**C{^{1}H}** (126 MHz, $CDCl_3$) δ 138.4 (C-2), 138.2 (C-2'), 115.1 (C-1), 114.8 (C-1'), 111.7 (C-12), 105.2 (C-8), 82.6 (C-9), 82.5 (C-10), 79.4, (C-7), 71.1 (C-5), 69.8 (C-5'), 68.1 (C-6), 30.4 (C-3, C-3'), 29.0 (C-4, C-4'), 27.0 (C-11), 26.5 (C-11') ppm. NMR spectroscopy data is in agreement with literature values.[1]

2.2.2 Monomer 4

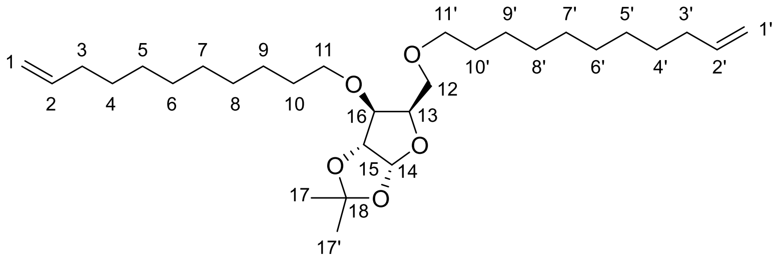

(4)

Yield = 60%. ^{1}**H NMR** (400 MHz, $CDCl_3$): δ 5.88 (1H, d, J = 3.8 Hz, H-14), 5.84–5.76 (2H, m, H-2, H-2'), 5.03–4.87 (4H, m, H-1, H-1'), 4.51 (1H, d, J = 3.8 Hz, H-15), 4.36–4.29 (1H, m, H-13), 3.80 (1H, d, J = 3.2 Hz, H-16), 3.69–3.35 (6H, m, H-11, H-11', H-12), 2.03 (4H, q, J = 7.0 Hz, H-3, H-3'), 1.59–1.50 (4H, m, H-10, H-10'), 1.48 (3H, s, H-17'), 1.40–1.23 (27H, m, H-4–H-9, H-4'–H-9', H-17) ppm; ^{13}**C{^{1}H}** (101 MHz, $CDCl_3$) δ 139.3 (C-2, C-2’), 114.3 (C-1, C-1’), 111.7 (C-18), 105.2 (C-11, C-11’), 82.5 (C-15), 79.4 (C-13), 71.9 (C-16), 70.7 (C-12), 68.0, 33.9 (C-3, C-3’), 29.8−29.1 (C-4−C-9, C-4’−C-9’), 26.9 (C-17), 26.4 (C-17’), 26.2 (C-10, C-10’) ppm. NMR spectroscopy data is in agreement with literature values.[1]

2.3 Synthesis of nucleoside-based α,ω-unsaturated ester monomers 5−9

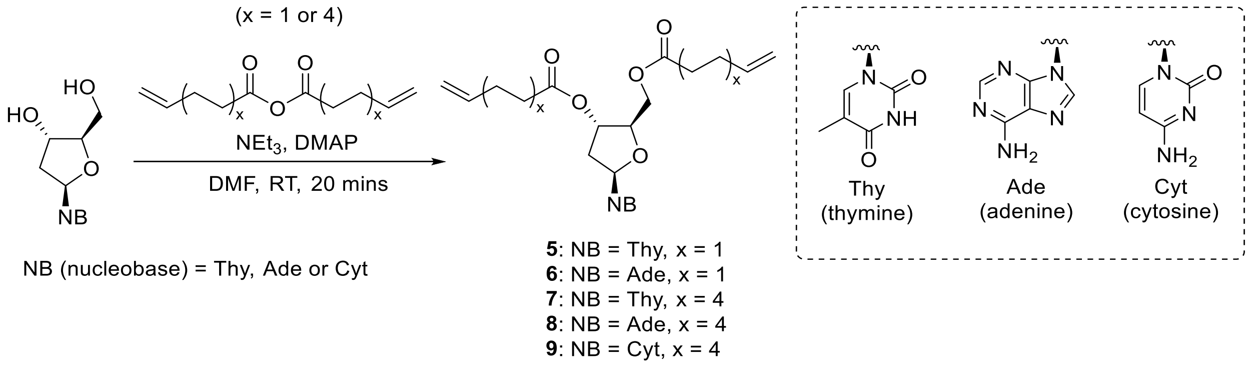

Nucleoside-based α,ω-unsaturated ester monomers were synthesised using an adapted procedure to the one used to synthesis the xylose-based analogues.[2] In a typical procedure, thymidine or deoxyadenosine (10.0 mmols, 1 equiv.) and the desired C5 or C11 ω-unsaturated anhydride (25.0 mmols, 2.5 equivs.) were added to a 100 mL round-bottom flask and dissolved in anhydrous DMF (40 mL). Triethylamine (4.18 mL, 30.0 mmols, 3 equivalents) was added followed by portion-wise addition of DMAP (0.37 g, 3.0 mmols, 0.3 equivs.). After 20 minutes, Amberlyst A-26(OH) ion-exchange resin (400 wt% *w.r.t.* nucleoside) was added and the suspension stirred for 2 hours. The resin was removed by sinter funnel filtration, washed with ethyl acetate (3 × 50 mL) and the combined filtrate washed with water (3 × 50 mL) and brine (50 mL). The organic layer was dried over $MgSO_4$, filtered and evaporated to dryness to yield an orange oil which was purified by column chromatography.

2.3.1 Monomer 5

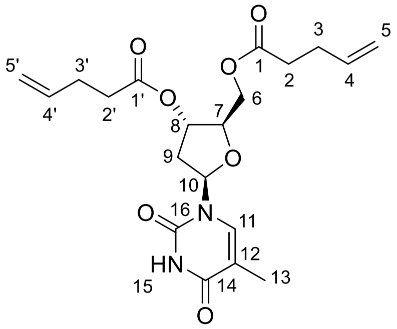

(5)

Chromatography eluent = 50:50 ethyl acetate/hexane. Appearance = clear, colourless oil. **Yield** = 63 %. **^{1}H NMR** ($CDCl_3$, 400 MHz) *δ* 9.55 (1H, s, H-15), 7.26 (1H, d, *J* = 1.3 Hz, H-11), 6.30 (1H, dd, *J* = 8.6, 5.5 Hz, H-10), 5.88–5.65 (2H, m, H-4, H-4'), 5.23–5.14 (1H, m, H-8), 5.11–4.95 (4H, m, H-5, H-5'), 4.43–4.27 (2H, m, H-6), 4.24–4.16 (1H, m, H-7), 2.49–2.41 (5H, m, H-3, H-3', H-9), 2.41–2.34 (4H, m, H-2, H-2'), 2.13 (1H, m, H-9), 1.91 (3H, d, *J* = 1.3 Hz, H-13) ppm; **^{13}C{^{1}H} NMR** ($CDCl_3$, 101 MHz) *δ* 172.6 (C-1), 172.4 (C-1'), 163.8 (C-14), 150.6 (C-16), 136.2 (C-4), 136.1 (C-4'), 134.6 (C-11), 116.1 (C-5, C-5'), 116.1 (C-5, C-5'), 111.7 (C-12), 84.9 (C-7), 82.3 (C-10), 74.2 (C-8), 63.9 (C-6), 37.6 (C-9), 33.5 (C-3), 33.3 (C-3'), 28.8 (C-2), 28.7 (C-2'), 12.8 (C-13) ppm. **FTIR (ATR, cm^{-1}):** 2923 & 2864 (CH (alkane

stretch, br), 1735 (C=O (ester) stretch, s), 1686 (C=O (thymine) stretch, s), 1461 (C−H (methyl) bend), 1168 (C−O (ester) stretch), C−O (ether) stretch, s) 751 (C−H bend, s). **MS** (+ve, MeCN): calculated m/z $[(C_{20}H_{26}N_2O_7)+H]^+$ = 407.1813, found m/z 407.1813.

2.3.2 Monomer 6

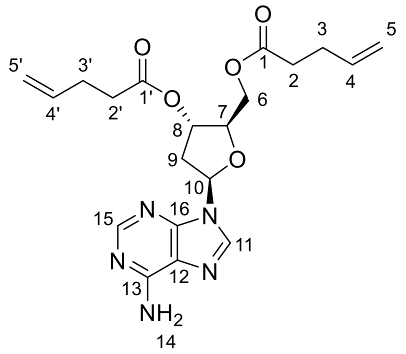

(6)

Chromatography eluent = 100 % ethyl acetate. Appearance = white, crystalline solid. **Yield** = 63 %. **^{1}H NMR** ($CDCl_3$, 500 MHz) δ 8.36 (1H, s, H-15), 7.98 (1H, s, H-11), 6.42 (1H, dd, J = 8.2, 5.8 Hz), 5.91–5.74 (2H, m), 5.63 (2H, s, H-14), 5.44 (1H, d, J = 6.5 Hz, H-8), 5.14–4.96 (4H, m, H-5), 4.45–4.30 (3H, m, H-6, H-7), 3.01–2.89 (1H, m, H-9), 2.61 (1H, dd, J = 14.1, 5.9 Hz, H-9), 2.54–2.31 (8H, m, H-2, H-2', H-3, H-3') ppm; **^{13}C NMR** ($CDCl_3$, 126 MHz) δ 172.6 (C-1), 172.5 (C-1'), 155.6 (C-13), 153.3 (C-15), 149.8 (C-16), 138.8 (C-11), 136.4 (C-4), 136.3 (C-4'), 120.3 (C-12), 116.1 (C-5), 116.0 (C-5'), 84.7 (C-7), 82.7 (C-10), 74.6 (C-8), 63.8 (C-6), 37.7 (C-9), 33.5 (C-3), 33.4 (C-3'), 28.9 (C-2), 28.8 (C-2') ppm. **FTIR (ATR, cm^{-1}):** 3123 (N−H stretch, br), 1737 (C=O (ester) stretch, s), 1676 (C=N stretch, s), 1606 (N−H (amine) bend, s), 1159 (C−O (ester) stretch, s), 1086 (C−N bend, s), 943 (C=C bend). **MS** (+ve, MeCN): calculated m/z $[(C_{20}H_{25}N_5O_5)+H]^+$ = 416.1929, found m/z 416.1929.

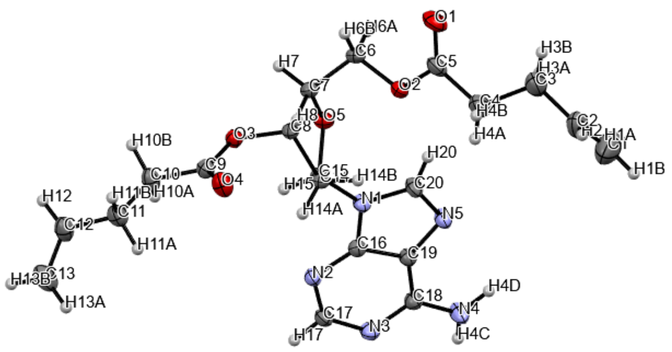

Figure S2.1: Obtained crystal structure of monomer **6** (CCDC-2289122).

Table S1: Crystallographic data for monomer **6** (*CCDC-2289122*).

Parameter	Units	Value
Empirical formula		C20 H25 N5 O5
Temperature	K	150
Wavelength	Å	1.54184
Crystal system		Monoclinic
Space group		$P2_1$
a	Å	10.02577
b	Å	8.19633
c	Å	13.46291
a	°	90
b	°	106.2144
g	°	90
Volume	$Å^3$	1062.303
Z		2
Density (calculated)	mg/m^3	1.299
Absorption coefficient	mm^{-1}	0.791
F(000)		440
Crystal size	mm^3	0.303 x 0.216 x 0.137
Theta range for data collection	°	3.419 to 73.306
Index ranges		−12<=h<=12, −10<=k<=10, −16<=l<=16
Reflections collected		18748
Independent reflections		4208 [R(int) = 0.0244]
Completeness to theta = 67.684°	%	100.0
Absorption correction		Semi-empirical from equivalents
Max. and min. transmission		1.00000 and 0.76095
Refinement method		Full-matrix least-squares on F^2
Data / restraints / parameters		4208 / 1 / 280
Goodness-of-fit on F^2		1.041
Final R indices [I>2sigma(I)]		R1 = 0.0258, wR2 = 0.0677
R indices (all data)		R1 = 0.0259, wR2 = 0.0679
Absolute structure parameter		−0.03
Extinction coefficient		0.0067
Largest diff. peak and hole	$e.Å^{-3}$	0.193 and -0.127

2.3.3 Monomer 7

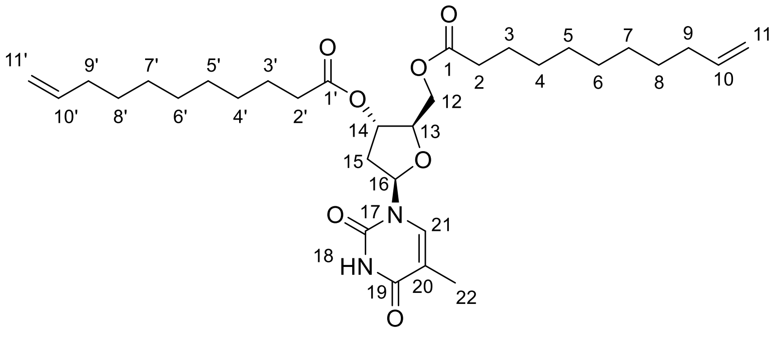

(7)

Chromatography eluent = 60:40 hexane/ethyl acetate. Appearance = clear, colourless oil. **Yield** = 78 %. **^{1}H NMR** (400 MHz, $CDCl_3$): δ 8.97 (1H, s, H-18), 7.28 (1H, s, H-21), 6.32 (1H, dd, J = 8.7, 5.6 Hz, H-16), 5.87–5.71 (2H, m, H-10), 5.20 (1H, d, J = 6.6 Hz, H-14), 5.04–4.85 (4H, m, H-11), 4.36 (2H, ddd, J = 46.3, 12.1, 3.7 Hz, H-12), 4.22 (1H, q, J = 3.0 Hz, H-13), 2.46 (1H, dd, J = 13.1, 6.6 Hz, H-15), 2.40–2.27 (4H, m, H-2, H-2'), 2.17–2.09 (1H, m, H-15), 2.08–1.98 (4H, m, H-9, H-9'), 1.93 (3H, s, H-22), 1.66–1.59 (4H, m, H-3, H-3'), 1.40–1.23 (20H, m, H-4–H-8, H-4'–H-8') ppm; **^{13}C{1H} NMR** (101 MHz, $CDCl_3$) δ 173.4 (C-1), 173.2 (C-1'), 163.6 (C-19), 150.4 (C-17), 139.3 (C-10), 139.2 (C-10'), 134.6 (C-21), 114.3 (C-11, C-11'), 111.7 (C-20), 84.9 (C-13), 82.5 (C-16), 74.1 (C-14), 63.8 (C-12), 37.8 (C-15), 34.3 (C-9), 34.2 (C-9'), 29.4−24.9 (C-2−C-8, C-2'−C-8'), 12.8 (C-22) ppm. **FTIR (ATR, cm^{-1}):** 3075 (N−H stretch, br), 2926 & 2854 (C−H (alkane) stretches, s), 1738 (C=O (ester) stretch, s), 1690 (C=O (thymine) stretch, s), 1465 (C−H (methyl) bend), 1162 (C−O (ester) stretch), 1097 (C−O (ether) stretch), 908 (C=C bend). **MS** (+ve, MeCN): calculated m/z $[(C_{32}H_{50}N_2O_7)+H]^+$ = 575.3691, found m/z = 575.3687.

2.3.4 Monomer 8

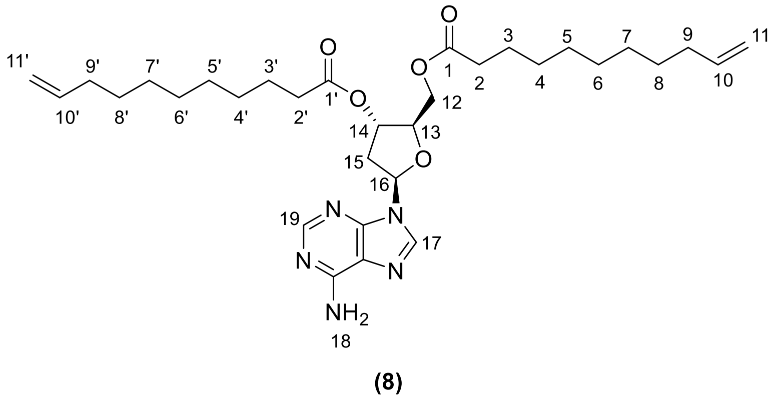

(8)

Chromatography eluent = 100% ethyl acetate. Appearance = waxy white solid. **Yield** = 76 %. **^{1}H NMR** (400 MHz, $CDCl_3$): δ 8.32 (1H, s, H-19), 8.26 (1H, s, H-17), 7.46 (2H, s, H-18), 6.48–6.42 (1H, m, H-16), 5.90–5.67 (2H, m, H-10), 5.41 (1H, d, J = 6.3 Hz, H-14), 5.02–4.87 (4H, m, H-11), 4.42–4.35 (3H, m, H-12, H-13), 2.90 (1H, t, J = 14.3 Hz, H-15), 2.72–2.64 (1H, m, H-15), 2.34 (4H, dt, J = 25.2, 7.6 Hz, H-2, H-2'), 2.06–1.97 (4H, m, H-9, H-9'), 1.68–1.55 (4H, m, H-3, H-3'), 1.39–1.21 (20H, m, H-4–H-8, H-

4'–H-8') ppm; **^{13}C{^{1}H}** NMR (101,MHz, $CDCl_3$) δ 173.4 (C-1), 173.3 (C-1'), 152.6 (C-22), 149.0 (C-19), 147.6 (C-20), 140.9 (C-17), 139.3 (C-10), 139.2 (C-10'), 119.6 (C-21), 114.3 (C-11, C-11'), 85.2 (C-13), 83.2 (C-16), 74.2 (C-14), 63.6 (C-12), 38.0 (C-15), 34.2 (C-9, C-9'), 29.4−24.9 (C-2−C-8, C-2'−C-8') ppm. **FTIR (ATR, cm^{-1}):** 3359–3123 (N−H stretches, s), 2919 & 2853 (C−H (alkane) stretches, s) 1746 (C=O (ester) stretch), 1678 (C=N stretch, s), 1603 (N−H (amine) bend, s), 1470 (C−H (alkane) bend, s), 1146 (C−O (ester) stretch, s), 1086 (C−N (amine) stretch, s), 991 (C=C bend, s). **MS** (+ve, MeCN): calculated m/z [($C_{32}H_{49}N_5O_5$)+H]$^+$ = 584.3807, found m/z = 584.3804.

2.3.5 Monomer 9

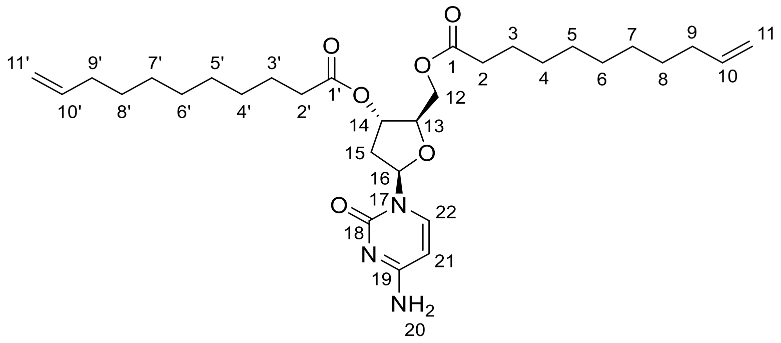

Chromatography eluent = 100% ethyl acetate. Appearance = clear, transparent solid. **Yield** = 86 %. **^{1}H NMR** (400 MHz, $CDCl_3$): δ 7.62 (1H, d, J = 7.4 Hz, H-22), 6.27 (1H, dd, J = 8.1, 5.5 Hz, H-16), 5.86–5.75 (2H, m, H-10, H-10'), 5.74 (1H, d, J = 7.5 Hz, H-21), 5.19 (1H, dt, J = 6.6, 2.3 Hz, H-14), 5.03–4.88 (4H, m, H-11, H-11'), 4.38–4.30 (2H, m, H-12), 4.26 (1H, q, J = 3.8 Hz, H-13), 2.69 (1H, ddd, J = 14.2, 5.5, 2.1 Hz, H-15), 2.36–2.28 (4H, m, H-2, H-2'), 2.09–1.99 (5H, m, H-9, H-9', H-15), 1.66–1.55 (4H, m, H-3, H-3'), 1.41–1.24 (20H, m, H-4–H-8, H-4'–H-8') ppm; **^{13}C{^{1}H}** NMR (101,MHz, $CDCl_3$) δ 173.5 (C-1), 173.3 (C-1'), 165.5 (C-19), 155.5 (C-18), 140.6 (C-22), 139.3 (C-10, C-10'), 114.3 (C-11, C-11'), 94.2 (C-21), 86.8 (C-13), 82.8 (C-16), 74.3 (C-14), 63.9 (C-12), 38.9 (C-15), 34.3 (C-9, C-9'), 29.4−24.9 (C-2−C-8, C-2'−C-8') ppm. **FTIR (ATR, cm^{-1}):** (3356 (N−H (amine) stretch, br), 3200 & 3084 (C−N stretches, br), 2924 & 2854 (C−H (alkane) stretches, s), 1735 (C=O (ester) stretch, s), 1622 (C=O (cytosine) stretch, s), 1364 (N−H (amine) bend, s), 1169 (C−O (ester) stretch, s), 1070 (C−O (ether) stretch), 784 (C−H bend, s). **MS** (+ve, MeCN): calculated m/z [($C_{31}H_{49}N_3O_6$)+H]$^+$ = 560.3699, found m/z = 560.3706.

3. Co-polymer Synthesis

3.1 General procedure

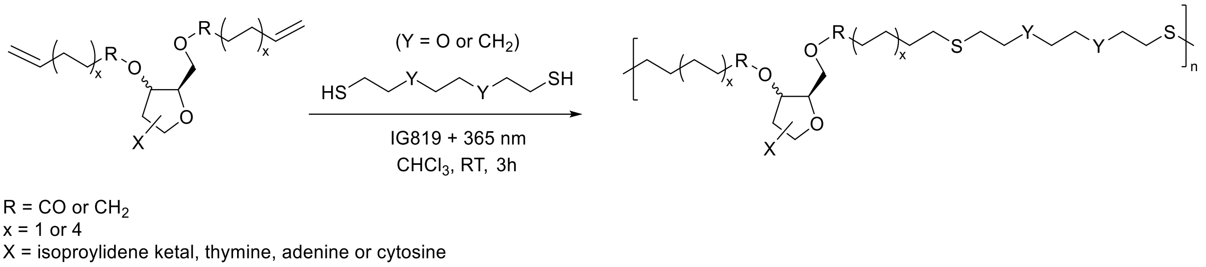

In a typical procedure, the desired α,ω-unsaturated ester or ether comonomer (1 equiv.) was measured into a vial and dissolved in chloroform (0.5 mol L^{-1}) followed by addition of 2,2'-(ethylenedioxy)diethanethiol (1 equiv.) and Irgacure 819 (0.1 equivs.). The solution was irradiated (λ = 365 nm) for 3 hours before precipitation into cold methanol (*ca.* 25 mL). The resulting suspension centrifuged (4500 rpm) for 5 minutes, the supernatant decanted and the product washed with cold methanol (3 × 10 mL). After drying in a vacuum oven overnight (70 °C), the corresponding co-polymer was obtained as a viscous yellow material.

3.1.1 Poly(1-EDT)

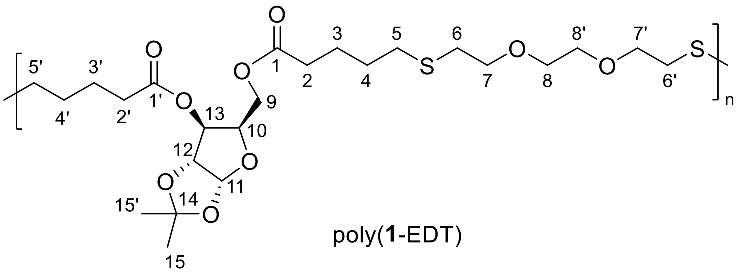

Yield = 47 % (some yield lost as spillage). **^{1}H NMR** (400 MHz, $CDCl_3$): *δ* 5.93 (1H, d, *J* = 3.8 Hz, H-11), 5.25 (1H, d, *J* = 3.1 Hz, H-13), 4.55–4.44 (2H, m, H-10, H-12), 4.30–4.14 (2H, m, H-9), 3.67–3.57 (8H, m, H-7, H-7', H-8, H-8'), 2.70 (4H, t, *J* = 7.6 Hz, H-5, H-5'), 2.56 (4H, td, *J* = 7.2, 4.3 Hz, H-6, H-6'), 2.40–2.31 (4H, m, H-2, H-2'), 1.79–1.67 (4H, m, H-3, H-3'), 1.66–1.56 (4H, m, H-4, H-4'), 1.52 (3H, s, H-15'), 1.31 (3H, s, H-15) ppm; **^{13}C{^{1}H}** NMR (101,MHz, $CDCl_3$) *δ* 173.0 (C-1), 172.1 (C-1'), 112.5 (C-14), 105.0 (C-11), 83.5 (C-12), 78.4 (C-10), 76.1 (C-12), 74.6 (C-13), 71.2−71.1 (C-8, C-8'), 70.4 (C-7, C-7'), 61.4 (C-9), 33.7−33.4 (C-2, C-2'), 32.2−31.5 (C-5, C-5'), 29.2−29.1 (C-6, C-6'), 26.8 (C-15'), 26.3 (C-15), 24.0 (C-3, C-3') ppm. **FTIR (ATR, cm^{-1}):** 2920 & 2864 (C−H stretches, w), 1736 (C=O stretch, s), 1106 & 1072 (C−O stretches), 857 (C−H bend). $\boldsymbol{M}_{n,SEC}$ (THF) = 6.4 kg mol^{-1} ($Đ_M$ = 2.85). $\boldsymbol{T}_g$ = −14 °C, $\boldsymbol{T}_m$ not observed. $\boldsymbol{T}_{d,5\%}$ = 254 °C, $\boldsymbol{T}_{d,max}$ = 281 and 333 °C.

3.1.2 Poly(2-EDT)

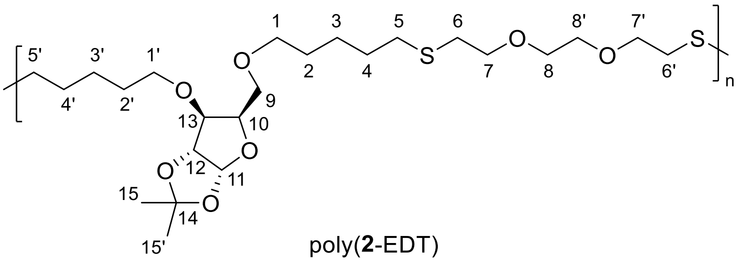

poly(**2**-EDT)

Yield = 84 %. **^{1}H NMR** (400 MHz, $CDCl_3$): δ 5.89 (1H, d, J = 3.8 Hz, H-11), 4.51 (1H, J = 3.9 Hz, H-12), 4.32 (1H, td, J = 6.0, 3.3 Hz, H-10), 3.79 (1H, d, J = 3.3 Hz, H-13), 3.66–3.37 (14H, m, H-1, H-1', H-7, H-7', H-8, H-8', H-9), 2.70 (4H, t, J = 7.1 Hz, H-5, H-5'), 2.54 (4H, t, J = 7.1 Hz, H-6, H-6'), 1.66–1.51 (8H, m, H-2, H-2', H-4, H-4'), 1.48 (3H, s, H-15'), 1.46–1.36 (4H, m, H-3, H-3'), 1.31 (3H, s, H-15) ppm; **^{13}C{^{1}H}** NMR (101,MHz, $CDCl_3$) δ 111.7 (C-14), 105.2 (C-11), 82.7 (C-13), 82.5 (C-12), 79.4 (C-10), 71.6 (C-8', C-8), 71.2 (C-7', C-7), 70.4 (C-1', C-1), 68.2 (C-9), 32.6 (C-6', C-6), 31.6 (C-5', C-5), 29.8, 29.67, 29.51, 29.47, 27.0 (C-15'), 26.47 (C-15), 25.6 (C-3), 25.5 (C-3') ppm. $\boldsymbol{M}_{\mathbf{n,SEC}}$ (THF) = 13.6 kg mol^{-1} ($Đ_M$ = 2.23). $\boldsymbol{T}_{\mathbf{g}}$ = −25 °C, $\boldsymbol{T}_{\mathbf{m}}$ not observed. $\boldsymbol{T}_{\mathbf{d,5\%}}$ = 216 °C, $\boldsymbol{T}_{\mathbf{d,max}}$ = 304 °C.

3.1.3 Poly(3-EDT)

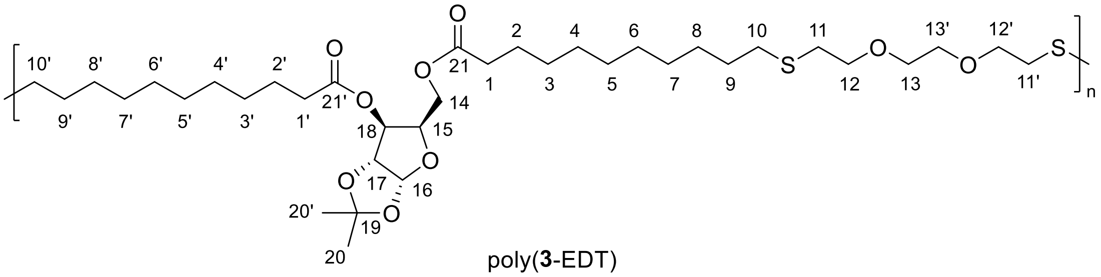

poly(**3**-EDT)

Yield = 100 %. **^{1}H NMR** (400 MHz, $CDCl_3$): δ 5.92 (1H, d, J = 3.8 Hz, H-16), 5.25 (1H, d, J = 3.3 Hz, H-18), 4.55–4.44 (2H, m, H-15, H-17), 4.28–4.15 (2H, m, H-14), 3.67–3.59 (8H, m, H-12, H-12', H-13, H-13'), 2.70 (4H, t, J = 7.1 Hz, H-10, H-10'), 2.57–2.49 (4H, m, H-11, H-11'), 2.32–2.26 (4H, m, H-1, H-1'), 1.64–1.50 (11H, m, H-2, H-2', H-9, H-9', H-20), 1.39–1.23 (27H, m, H-3–H-8, H-3'–H-8', H-20') ppm; **^{13}C{^{1}H}** NMR (101,MHz, $CDCl_3$) δ 173.5 (C-21), 172.5 (C-21’), 112.4 (C-19), 105.1 (C-16), 83.6 (C-15), 76.0 (C-18), 71.2 (C-13), 70.4 (C-12, C-12’), 61.3 (C-14), 34.2 (C-1, C-1’), 32.7 (C-11, C-11’), 31.5 (C-10, C-10’), 30.0–29.0 (C-2–C-8, C-2’–C-8’), 26.9 (C-20), 26.4 (C-20), 25.0 (C-9), 24.9 (C-9’) ppm. **FTIR (ATR, cm^{-1}):** 2923 & 2852 (C–H stretches), 1738 (C=O stretch), 1467 (C–H bend), 1113, 1074 & 1020 (C–O stretches), 753 (C–H bend). $\boldsymbol{M}_{\mathbf{n,SEC}}$ (THF) = 12.3 kg mol^{-1} ($Đ_M$ = 5.41). $\boldsymbol{T}_{\mathbf{g}}$ = −34 °C, $\boldsymbol{T}_{\mathbf{m}}$ −11 °C. $\boldsymbol{T}_{\mathbf{d,5\%}}$ = 281 °C, $\boldsymbol{T}_{\mathbf{d,max}}$ = 293 and 362 °C.

3.1.4 Poly(3-ODT)

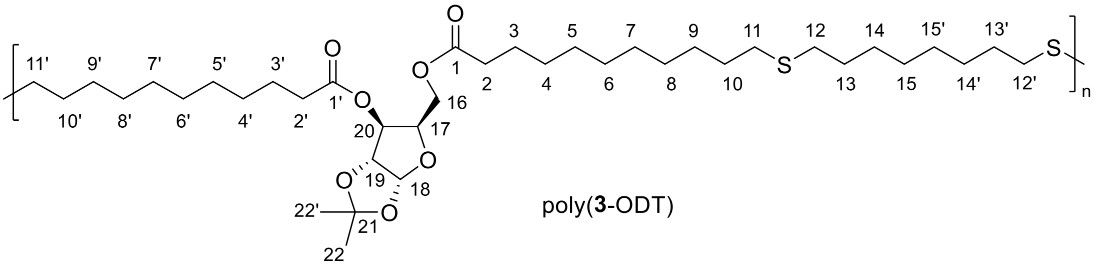

poly(**3**-ODT)

Yield = 100 %. **^{1}H NMR** (400 MHz, $CDCl_3$): *δ* 5.92 (1H, d, *J* = 3.8 Hz, H-18), 5.25 (1H, d, *J* = 3.2 Hz, H-20), 4.51–4.47 (2H, m, H-17, H-19), 4.28–4.15 (2H, m, H-16), 2.48 (8H, t, *J* = 7.5 Hz, H-11, H-11', H-12, H-12'), 2.33–2.28 (4H, m, H-2, H-2'), 1.63–1.53 (12H, m, H-3, H-3', H-10, H-10', H-13, H-13'), 1.51 (3H, s, H-22'), 1.41–1.21 (35H, m, H-4−H-9, H-4'−H-9', H-14, H-14', H-15, H-15', H-22) ppm; **^{13}C{^{1}H}** NMR (101,MHz, $CDCl_3$) *δ* 173.5 (C-1), 172.6 (C-1'), 112.4 (C-21), 105.1 (C-18), 83.6 (C-17), 76.0 (C-20), 61.3 (C-16), 34.2 (C-2, C-2'), 32.4 (C-12), 32.3 (C-12'), 29.9–29.0 (C-3–C-9, C-3'–C-9', C-13–C-15), 26.9 (C-22), 26.4 (C-22'), 25.0 (C-10), 24.9 (C-10') ppm. **FTIR (ATR, cm^{-1}):** 2920 & 2851 (C–H stretches), 1740 (C=O stretch), 1467 (C–H bend), 1161, 1072 & 1017 (C–O stretches), 719 (C–H bend). $M_{n,SEC}$ (THF) = 12.5 kg mol^{-1} ($Đ_M$ = 2.70). T_g = 5 °C, T_m 41 °C. $T_{d,5\%}$ = 269 °C, $T_{d,max}$ = 279 and 395 °C.

3.1.5 Poly(4-EDT)

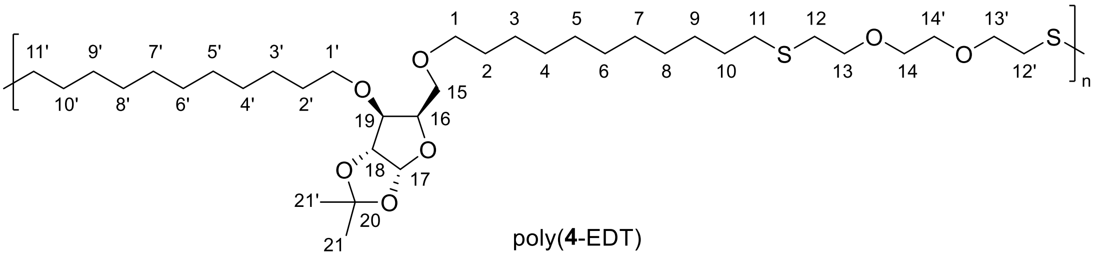

poly(**4**-EDT)

Yield = 100 %. **^{1}H NMR** (400 MHz, $CDCl_3$): *δ* 5.89 (1H, d, *J* = 3.8 Hz, H-17), 4.52 (1H, d, *J* = 3.9 Hz, H-18), 4.33 (1H, td, *J* = 6.1, 3.2 Hz, H-16), 3.80 (1H, d, *J* = 3.3 Hz, H-19), 3.69–3.37 (14H, m, H-1, H-1', H-13, H-13', H-14, H-14', H-15), 2.71 (4H, t, *J* = 7.1 Hz, H-11, H-11'), 2.53 (4H, t, *J* = 7.4 Hz, H-12, H-12'), 1.60–1.52 (8H, m, H-2, H-2'), 1.49 (3H, s, H-21), 1.38–1.22 (31H, m, H-3–H-9, H-3'–H-9', H-21') ppm; **^{13}C{^{1}H}** NMR (101,MHz, $CDCl_3$) *δ* 111.7 (C-20), 105.3 (C-17), 82.6 (C-19, C-18), 79.4 (C-16), 71.9 (C-14, C-14'), 71.2 (C-13, C-13'), 70.4 (C-1, C-1'), 68.0 (C-15), 32.8 (C-12, C-12'), 31.5 (C-11, C-11'), 30.0–29.1 (C-2–C-9, C-2'–C-9'), 27.0 (C-21'), 26.3 (C-21), 26.2 (C-10, C-10') ppm. **FTIR (ATR, cm^{-1}):** 2992 & 2853 (C–H stretches), 1108, 1081 & 1014 (C–O stretches), 887 & 856 (C–H bends). $M_{n,SEC}$ (THF) = 10.8 kg mol^{-1} ($Đ_M$ = 3.41). T_g = −31 °C, T_m −3 °C. $T_{d,5\%}$ = 271 °C, $T_{d,max}$ = 350 °C.

3.1.6 Poly(5-EDT)

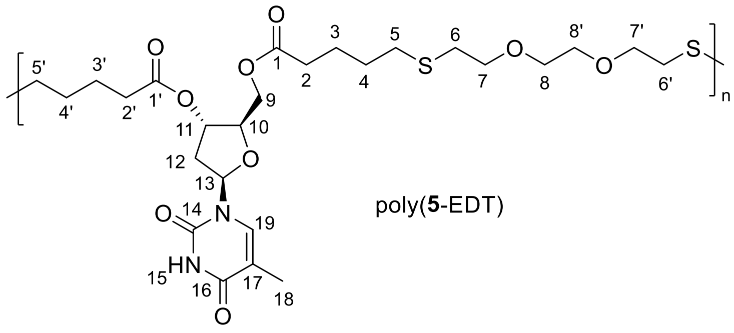

poly(**5**-EDT)

Yield = 100 %. **^{1}H NMR** ($CDCl_3$, 500 MHz) δ 9.61 (1H, (br)s, H-15), 7.26 (1H, s, H-11), 6.31–6.20 (1H, m, H-13), 5.19 (1H, d, J = 6.5 Hz, H-11), 4.45–4.25 (2H, m, H-9), 4.23–4.16 (1H, m, H-10), 3.65–3.56 (8H, m, H-7, H-7’, H-8, H-8’), 2.76–2.63 (4H, m, H-6, H-6’), 2.55 (4H, q, J = 7.1 Hz, H-5, H-5’), 2.48–2.42 (1H, m, H-12), 2.40–3.34 (4H, d, J = 7.6 Hz, H-2, H-2’), 2.25–2.10 (1H, m, H-12), 1.91 (3H, s, H-18), 1.76–1.68 (4H, m, H-3, H-3’), 1.65–1.57 (4H, m, H-4, H-4’) ppm; **^{13}C{^{1}H}** NMR (126,MHz, $CDCl_3$) δ 172.9 (C-1), 172.8 (C-1’), 150.6 (C-16), 134.8 (C-14), 133.7 (C-19), 111.6 (C-17), 85.1 (C-10), 82.4 (C-13), 74.2 (C-11), 71.1 (C-8, C-8’), 70.4 (C-7, C-7’), 63.9 (C-9), 37.7 (C-12), 33.8–33.7 (C-6, C-6’), 32.2–32.1 (C-5, C-5’), 31.5 (C-4, C-4’), 29.2–29.1 (C-3, C-3’), 24.2–23.9 (C-2, C-2’), 12.9 (C-18) ppm. **FTIR (ATR, cm^{-1}):** 2923 & 2864 (C–H (alkane) stretches, br), 1735 (C=O (ester) stretch, s), 1686 (C=O (thymine) stretch, s), 1461 (C–H (methyl) bend), (1168 (C–O (ester) stretch), 1098 (C–O (ether) stretch, s), 751 (C–H bend). $\boldsymbol{M}_{\mathbf{n,SEC}}$ (THF) = 3.7 kg mol^{-1} ($Đ_M$ = 2.66). $\boldsymbol{T}_{\mathbf{g}}$ = 3 °C, $\boldsymbol{T}_{\mathbf{m}}$ not observed. $\boldsymbol{T}_{\mathbf{d,5\%}}$ = 142 °C, $\boldsymbol{T}_{\mathbf{d,max}}$ = 301 °C.

3.1.7 Poly(6-EDT)

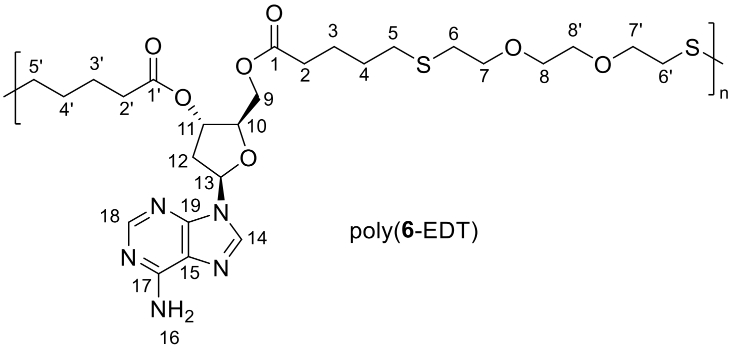

poly(**6**-EDT)

Yield = 100 %. ^{1}H and ^{13}C{^{1}H} NMR spectra not obtained due to insolubility of product. **FTIR (ATR, cm^{-1}):** 3321 (N–H (amine) stretch, br), 2920 & 2863 (C–H (alkane) stretches), 1734 (C=O (ester) stretch, s), 1163 (C–O (ester) stretch), 1099 (C–N bend), 749 (C–H bend, s). $\boldsymbol{M}_{\mathbf{n,SEC}}$ (THF) = 2.5 kg mol^{-1} ($Đ_M$ = 2.0). $\boldsymbol{T}_{\mathbf{g}}$ = −29 °C, $\boldsymbol{T}_{\mathbf{m}}$ not observed. $\boldsymbol{T}_{\mathbf{d,5\%}}$ = 123°C, $\boldsymbol{T}_{\mathbf{d,max}}$ = 354 °C.

3.1.8 Poly(7-EDT)

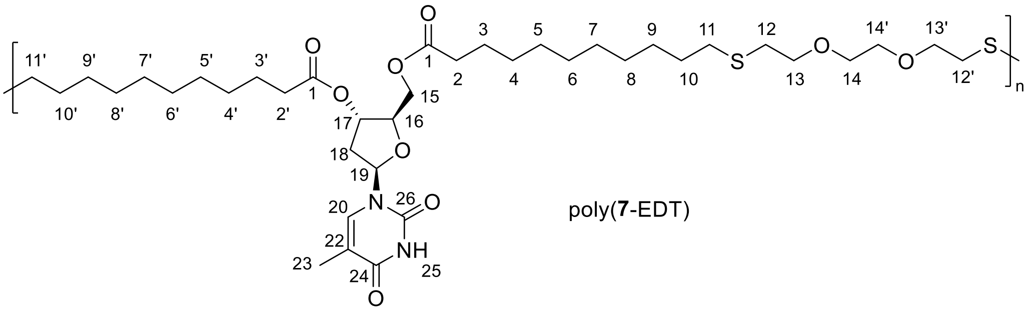

poly(**7**-EDT)

Yield = 93 %. **^{1}H NMR** ($CDCl_3$, 400 MHz) *δ* 9.15 (1H, s, H-25), 7.29 (1H, d, *J* = 1.3 Hz, H-20), 6.32 (1H, dd, *J* = 8.4, 5.4 Hz, H-19), 5.20 (1H, d, *J* = 6.6 Hz, H-17), 4.45–4.28 (2H, m, H-15), 4.23 (1H, q, *J* = 3.0 Hz, H-16), 3.66–3.61 (8H, m, H-13, H-13’, H-14, H-14’), 2.70 (4H, t, *J* = 7.1 Hz, H-11, H-11’), 2.53 (4H, td, *J* = 7.5, 1.6 Hz, H-12, H-12’), 2.46 (1H, ddd, *J* = 14.1, 5.6, 2.0 Hz, H-18), 2.35 (4H, td, *J* = 7.8, 3.6 Hz, H-2, H-2’), 2.17–2.08 (1H, m, H-18), 1.93 (3H, s, H-23), 1.67–1.51 (8H, m, H-3, H-3’, H-10, H-10’), 1.10–1.21 (24H, m, H-4–H-9, H-4’–H-9’) ppm; **^{13}C{^{1}H}** NMR (126, MHz, $CDCl_3$) *δ* 173.4 (C-1), 173.2 (C-1’), 163.8 (C-24), 150.5 (C-26), 134.6 (C-20), 111.5 (C-22), 84.9 (C-16), 82.4 (C-19), 74.2 (C-17), 71.1–70.2 (C-13, C-13’, C-14, C-14’), 63.9 (C-15), 37.8 (C-18), 34.3–34.2 (C-12, C-12’), 32.7 (C-11, C-11’), 31.5 (C-10, C-10’), 31.5–28.9 (C-3–C-9, C-3’–C-9’), 24.9 (C-2, C-2’), 12.8 (C-23) ppm. **FTIR (ATR, cm^{-1}):** 2923 & 2852 (C–H (alkane) stetches, s) 1738 (C=O (ester) stretch, s), 1689 (C=O (thymine) stretch, s), 1464 (C–H (methyl) bend), 1163 (C–O (ester) stretch), 1096 (C–O (ether) stretch, s). $\boldsymbol{M}_{\mathbf{n,SEC}}$ (THF) = 14.7 kg mol^{-1} ($Đ_M$ = 2.6). $\boldsymbol{T}_{\mathbf{g}}$ = −7 °C, $\boldsymbol{T}_{\mathbf{m}}$ not observed. $\boldsymbol{T}_{\mathbf{d,5\%}}$ = 288 °C, $\boldsymbol{T}_{\mathbf{d,max}}$ = 312 and 394 °C.

3.1.9 Poly(8-EDT)

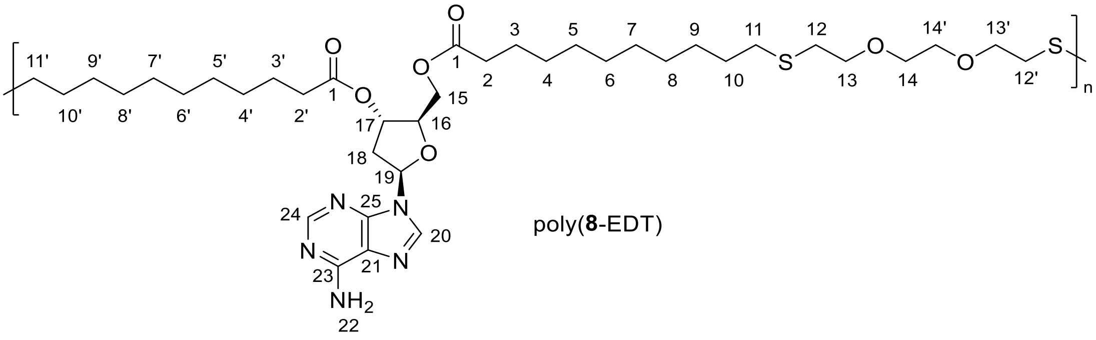

poly(**8**-EDT)

Yield = 69 %. ^{1}H and ^{13}C{^{1}H} NMR spectra not obtained due to insolubility of product. $\boldsymbol{M}_{\mathbf{n,SEC}}$ not determined due to insolubility of product. **FTIR (ATR, cm^{-1}):** 2923 & 2852 (C–H (alkane) stretches, s), 1737 (C=O (ester) stretch, s), 1609 (N–H (amine) bend), 1162 (C–O (ester) stretch), 1098 (C–O (ether) stretch, s). $\boldsymbol{T}_{\mathbf{g}}$ = −34 °C, $\boldsymbol{T}_{\mathbf{m}}$ 45 and 66 °C. $\boldsymbol{T}_{\mathbf{d,5\%}}$ = 239 °C, $\boldsymbol{T}_{\mathbf{d,max}}$ = 374 °C.

3.1.10 Poly(9-EDT)

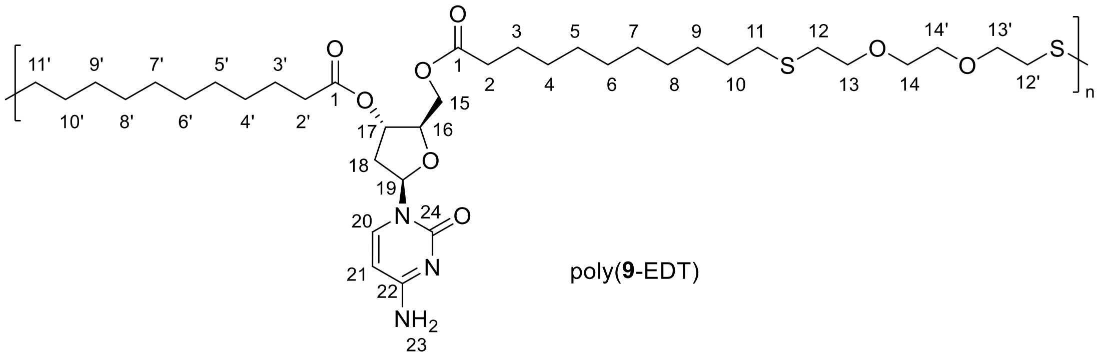

poly(**9**-EDT)

Yield = 61 %. ^{1}H and $^{13}C\{^{1}H\}$ NMR spectra not obtained due to insolubility of product. $\boldsymbol{M_{n,SEC}}$ not obtained due to insolubility of product. **FTIR (ATR, cm^{-1}):** 3331 (N–H (amine) stretch, br), 2920 & 2851 (C–H (alkane) stretches, s), 1735 (C=O (ester) stretch, s), 1652 (C=O (cytosine) stretch, s), 1103 (C–O (ether) stretch, s). $\boldsymbol{T_g}$ = −37 °C, $\boldsymbol{T_m}$ not observed. $\boldsymbol{T_{d,5\%}}$ = 254 °C, $\boldsymbol{T_{d,max}}$ = 363 °C.

4. SPE Preparation Procedures

4.1 Preparation of covalently cross-linked xylose-based SPE films

SPE synthesis was performed inside a glovebox to prevent contact with moisture. The desired xylose-based α,ω-unsaturated ester or ether monomer (approximately 400 mg, 1 equiv.) was measured out into a vial inside an argon-filled glovebox, followed by addition of the desired amount of lithium bis(trifluorosulfonylimide) (LiTFSI) and phenylbis(2,4,6-trimethylbenzoyl)phosphine oxide (IG819) (0.1 equivs.). A mixture of 2,2'-(ethylenedioxy)diethanethiol and trimethylolpropane tris(3-mercaptopropionate) totalling 1 equiv. was then transferred into the vial and anhydrous THF (2.5 mL) was added. The vial was covered in foil and the contents stirred for at least 2 hours before the solution was transferred into a PTFE evaporating dish. After evaporation of the solvent overnight, the dish was irradiated (λ = 365 nm) for 3 hours. The resulting film was then dried in a vacuum oven (70 °C) for at least 24 hours before being stored under argon.

4.2 Preparation of nucleoside-based SPE films

The desired ratio of the nucleoside-based α,ω-unsaturated ester monomers (either **5** & **6** or **7** & **8**, totalling 1 equiv., approximately 400 mg) was measured out into a vial inside an argon-filled glovebox, followed by addition of the desired amount of lithium bis(trifluorosulfonylimide) (LiTFSI) and phenylbis(2,4,6-trimethylbenzoyl)phosphine oxide (IG819) (0.1 equivs.). 2,2'-(ethylenedioxy)diethanethiol (1 equiv.) was added, followed by anhydrous THF (2.5 mL). The vial was covered in foil and the contents stirred for at least 2 hours before the solution was transferred into a PTFE evaporating dish. After evaporation of the solvent overnight, the dish was irradiated (λ = 365 nm) for 3 hours. The resulting film was then dried in a vacuum oven (70 °C) for at least 24 hours and then reprocessed using a hot press (80 °C) if required. The resulting film was stored under argon.

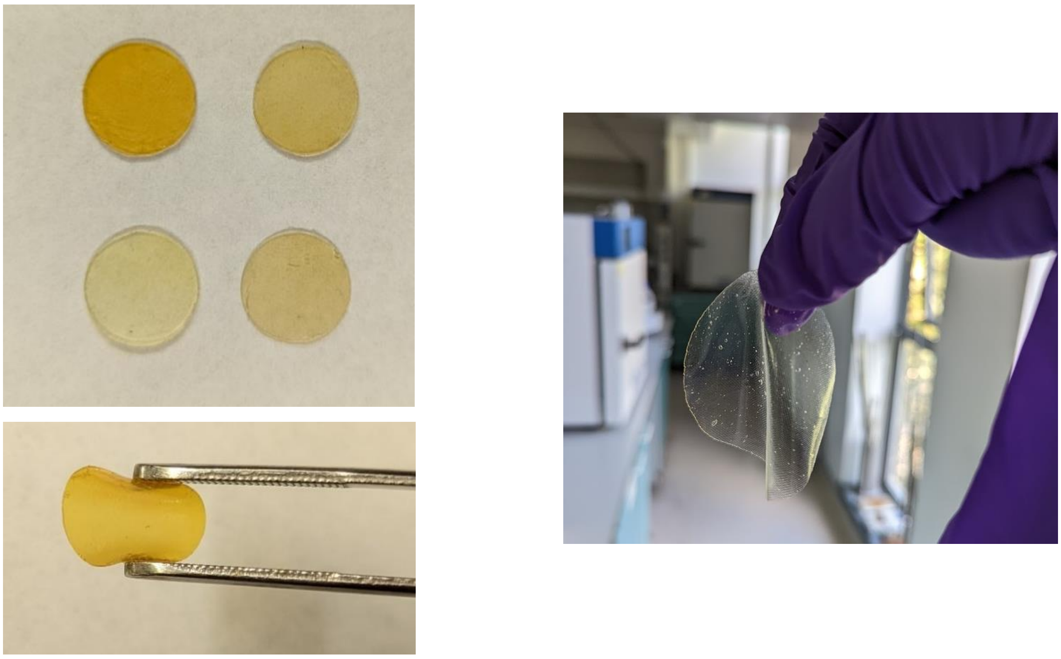

Figure S4.1: Photos of representative covalently crosslinked SPEs (left) and non-covalently cross-linked nucleoside-based SPE (right).

4.3 Preparation of a PEO-based SPE film

PEO (M_n = 100 kg mol^{-1}, 0.50 g, 11.4 mmols C−O, 1 equiv.) and LiTFSI (0.26 g, 34 wt%, 0.9 mmols, 8 mol%,) were stirred in anhydrous acetonitrile under an inert atmosphere until the polymer had dissolved (gentle heating required). The solution was cast to a PTFE evaporating dish and left until the solvent had evaporated. The material was then dried in a vacuum oven (50 °C) for 3 days to yield a semi-transparent, white PEO SPE film. The resulting film was then stored under argon.

5. Determination of TMP mole fraction for covalently cross-linked xylose-based SPE films

The data from the following table was used in the determination of the TMP mole fraction required to sufficiently cross-link poly(**1**-EDT).

Table S2: Polymerisation data from an initial study of the crosslinking thiol-ene co-polymerisation of monomer **1** with EDT and TMP.[a]

Entry	EDT:TMP	Conversion[b] (%)	$M_{n,SEC}$ [$Đ_M$] [c] (kg mol^{-1})	T_g (°C) [d]	Comments
1	99:1	100	7.5 [3.8]	−16	Sticky
2	97.5:2.5	100	7.2 [3.5]	−19	Sticky
3	95:5	100	5.1 [3.0]	−26	Sticky
4	90:10	100	6.2[f]	−16	Solid, poorly soluble
5	80:20	–[e]	–[e]	−6	Solidified during polymerisation
6	50:50	–[e]	–[e]	0	Solidified during polymerisation

[a] Polymerisations were performed at room temperature for 3 hours in $CHCl_3$ (0.5 M) under UV irradiation (λ = 365 nm).

[b] Calculated by comparing the relative integration of the terminal alkene signals of the diene comonomer (around 4.8 and 5.2 ppm) with the proton signals of the furanose ring (around 5.4 ppm) in the ^{1}H NMR spectrum of the crude product.

[c] Calculated by SEC using refractive index methods relative to polystyrene standards in THF, $Đ_M = M_W/M_n$.

[d] Determined from the second cooling and heating cycle between −60 and 200 °C.

[e] Not determined dye to insolubility of polymer.

[f] Not reliable as polymer was only partially soluble.

The results from the table above indicate that a sufficiently high mole fraction (>0.1) of TMP should be used to produce a solid co-polymer based on monomer **1**. Interestingly, as the mole fraction of TMP was increased from 0.01 to 0.05 (entries 1−3), the molar mass achieved decreased from 7.5 to 5.1 kg mol^{-1}. This decrease explains the decrease of T_g from −16 to −26 °C which was observed. It may seem surprising that the molar mass decreased with an increasing amount of TMP since there is more crosslinker to link the chains. However, since TMP replaced EDT in an equimolar proportion despite containing an extra SH group, the overall stoichiometry of C=C to SH in the solution would have deviated further from the correct 1:1 ratio, therefore limiting the molar mass.

The polymers obtained from entries 1−3 remained soluble in $CHCl_3$ and their ^{1}H NMR spectra were identical. No signals from TMP were identified, therefore suggesting that little/no units of TMP were incorporated into the polymer at these low mole fractions. Entry 4 remained soluble throughout the polymerisation but was poorly soluble once isolated and dried, suggesting that TMP had been incorporated into the polymer, and entries 5 and 6 resulted in a solidification of the polymerisation. Based on these results, a ratio of 8:2 (EDT:TMP) was selected for the rest of the study.

6. NMR Spectra

Monomers

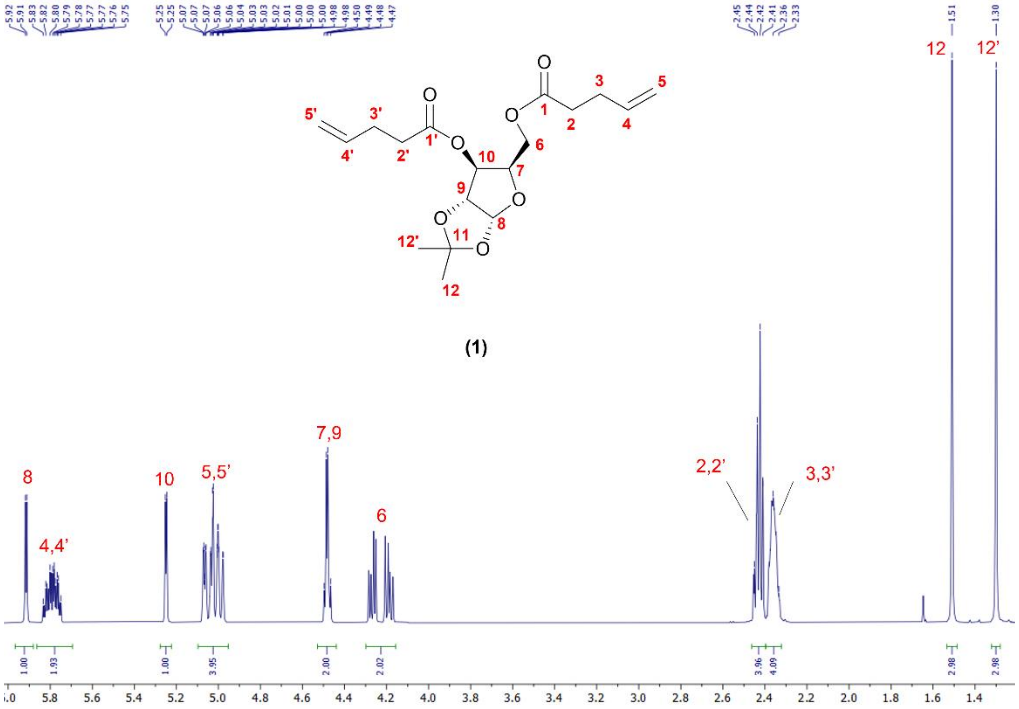

Figure S6.1: ^{1}H NMR spectrum of monomer **1** in $CDCl_3$.

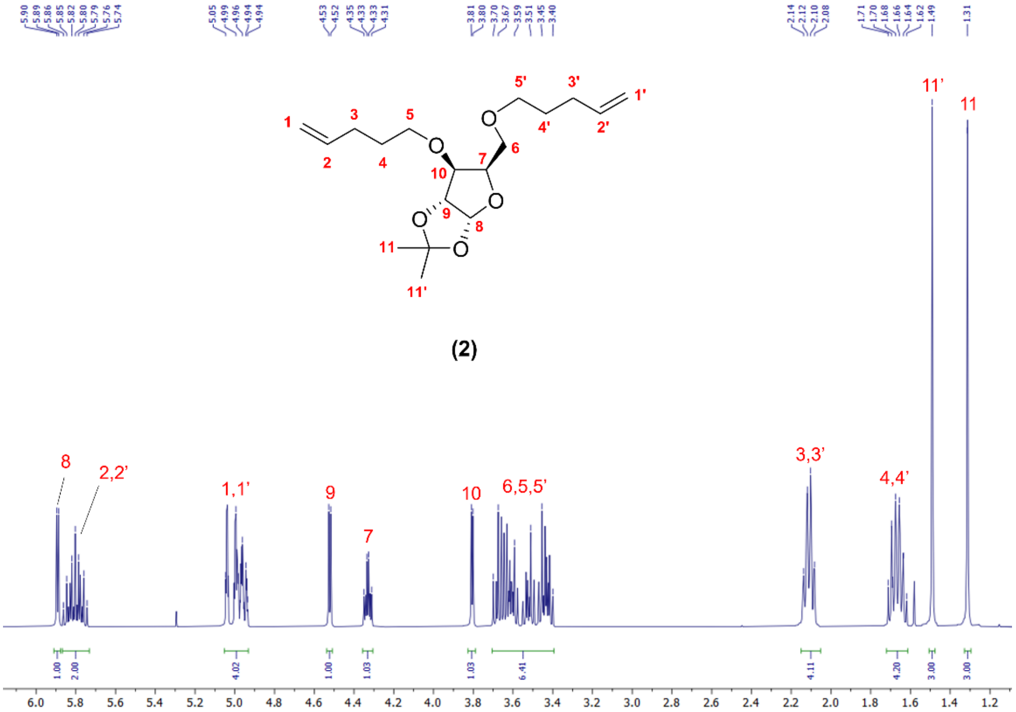

Figure S6.2: ^{1}H NMR spectrum of monomer **2** in $CDCl_3$.

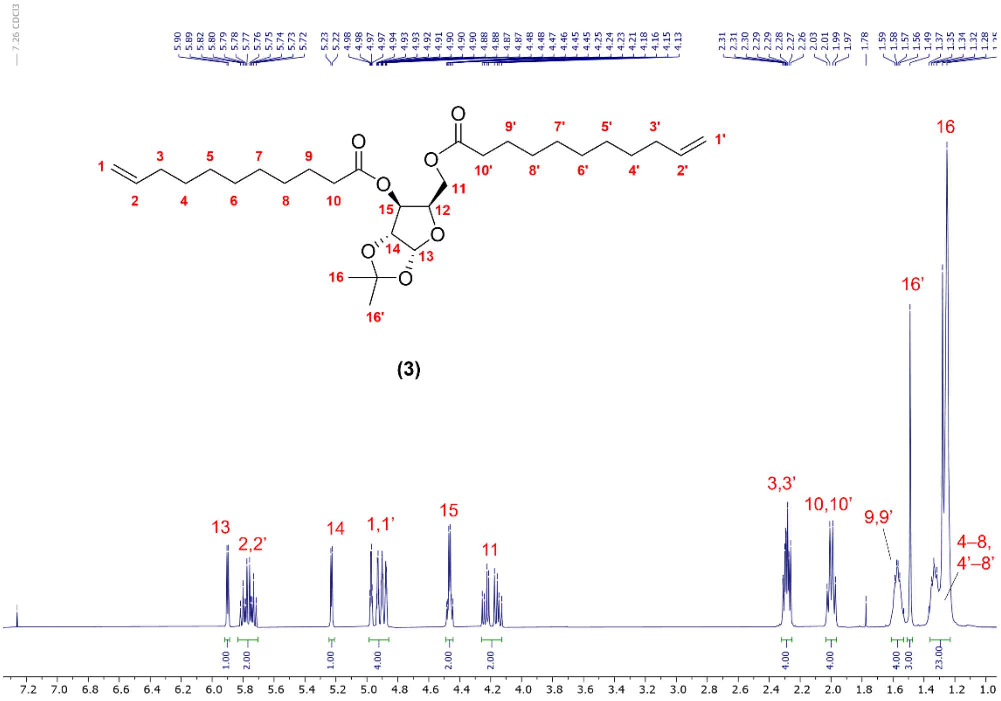

Figure S6.3: ^{1}H NMR spectrum of monomer **3** in $CDCl_3$.

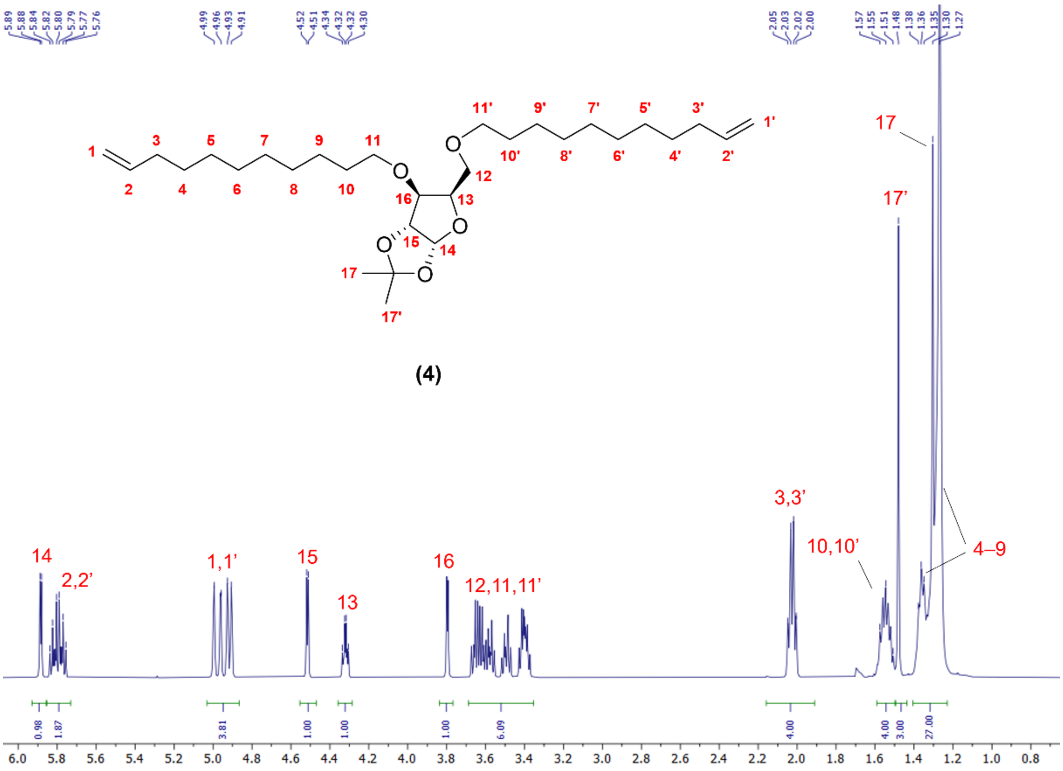

Figure S6.4: ^{1}H NMR spectrum of monomer **4** in $CDCl_3$.

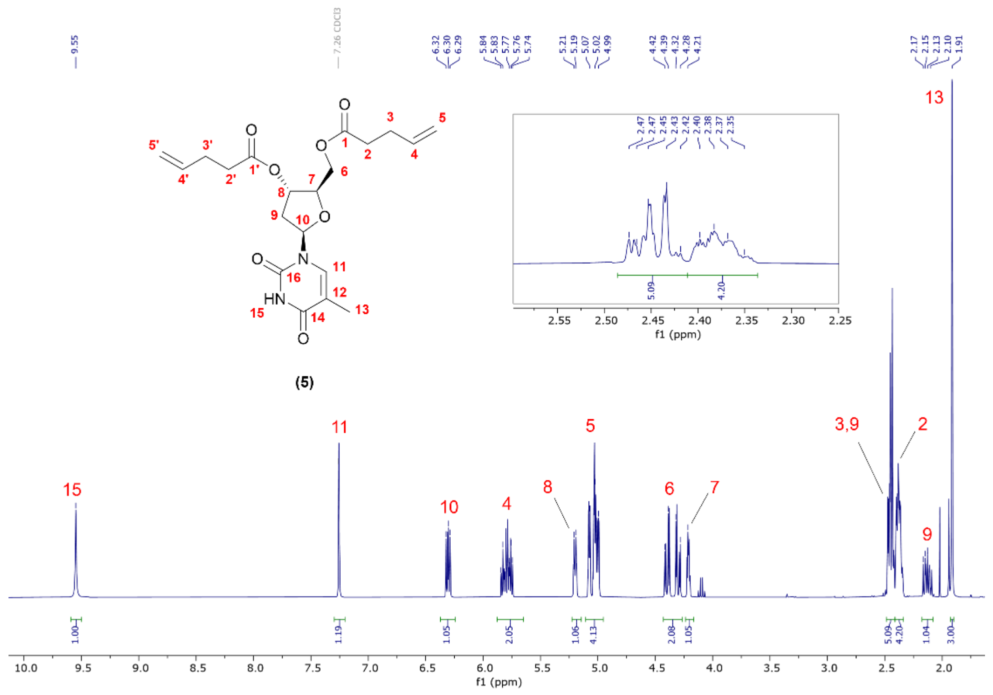

Figure S6.5: ^{1}H NMR spectrum of monomer **5** in $CDCl_3$.

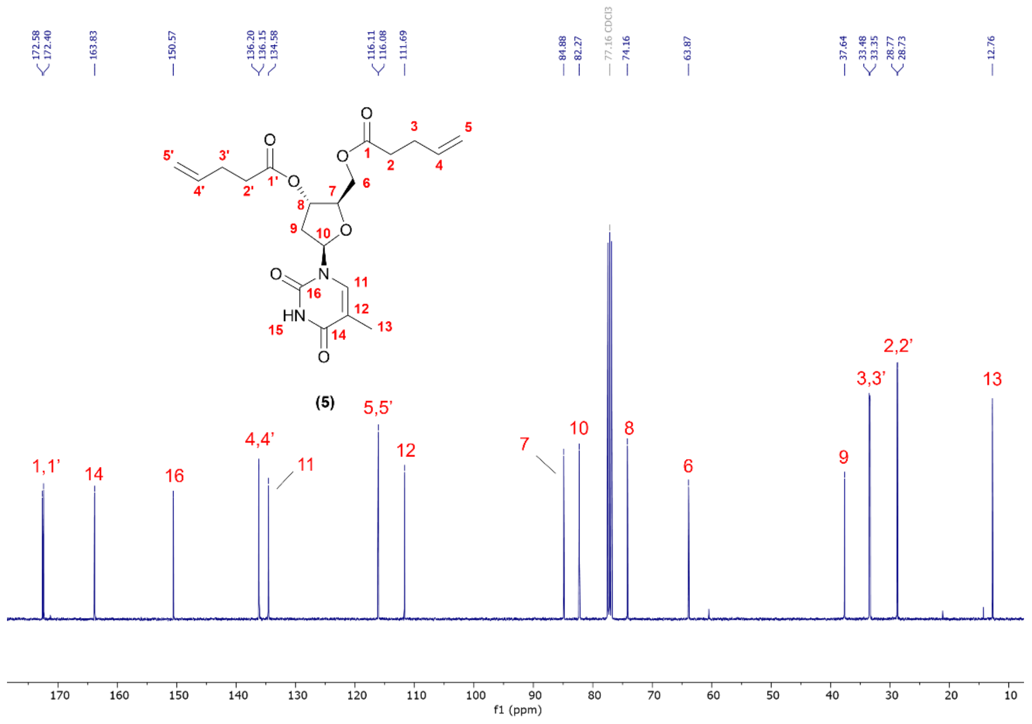

Figure S6.6: $^{13}C\{^{1}H\}$ NMR spectrum of monomer **5** in $CDCl_3$.

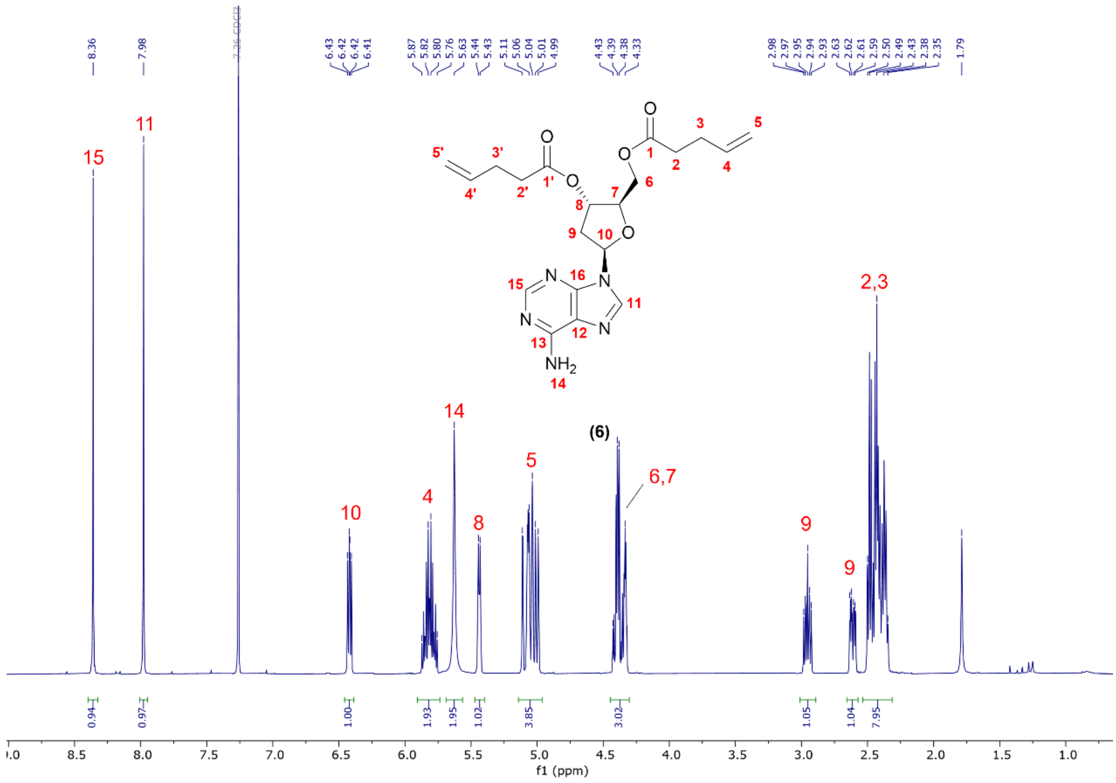

Figure S6.7: ^{1}H NMR spectrum of monomer **6** in $CDCl_3$.

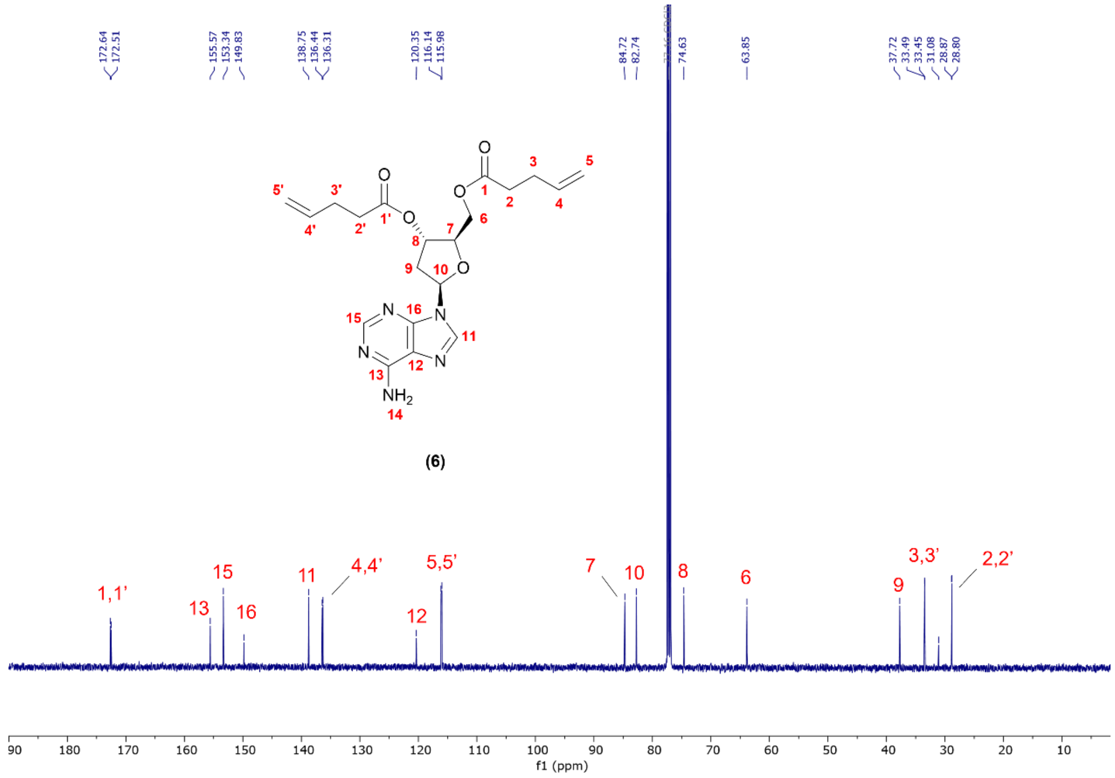

Figure S6.8: $^{13}C\{^1H\}$ NMR spectrum of monomer **6** in $CDCl_3$.

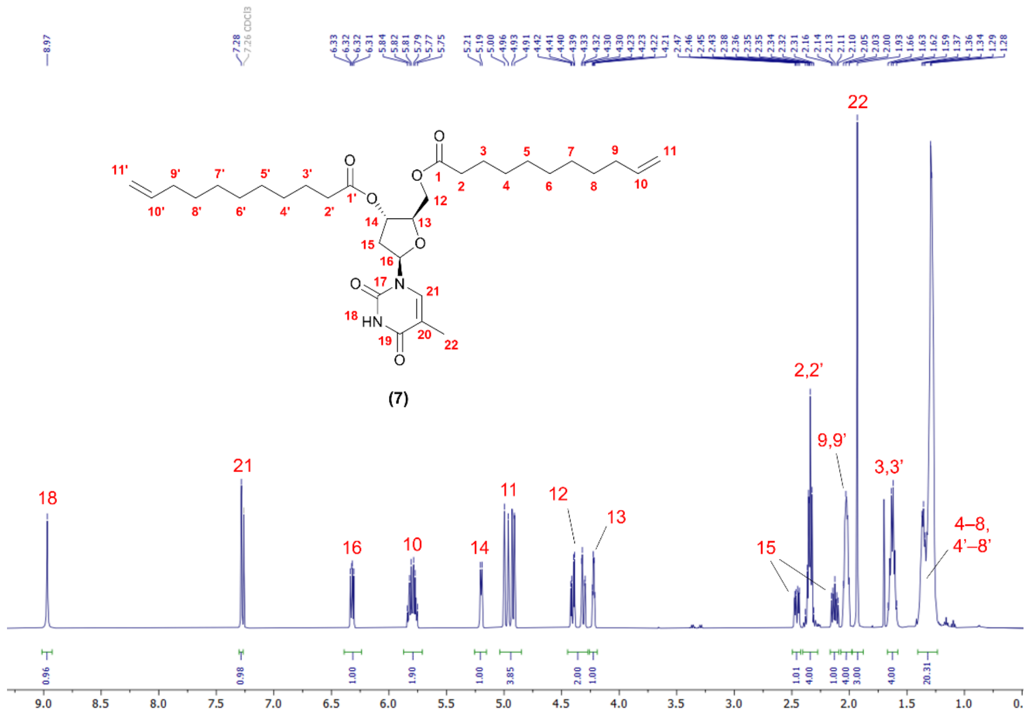

Figure S6.9: ^{1}H NMR spectrum of monomer **7** in $CDCl_3$.

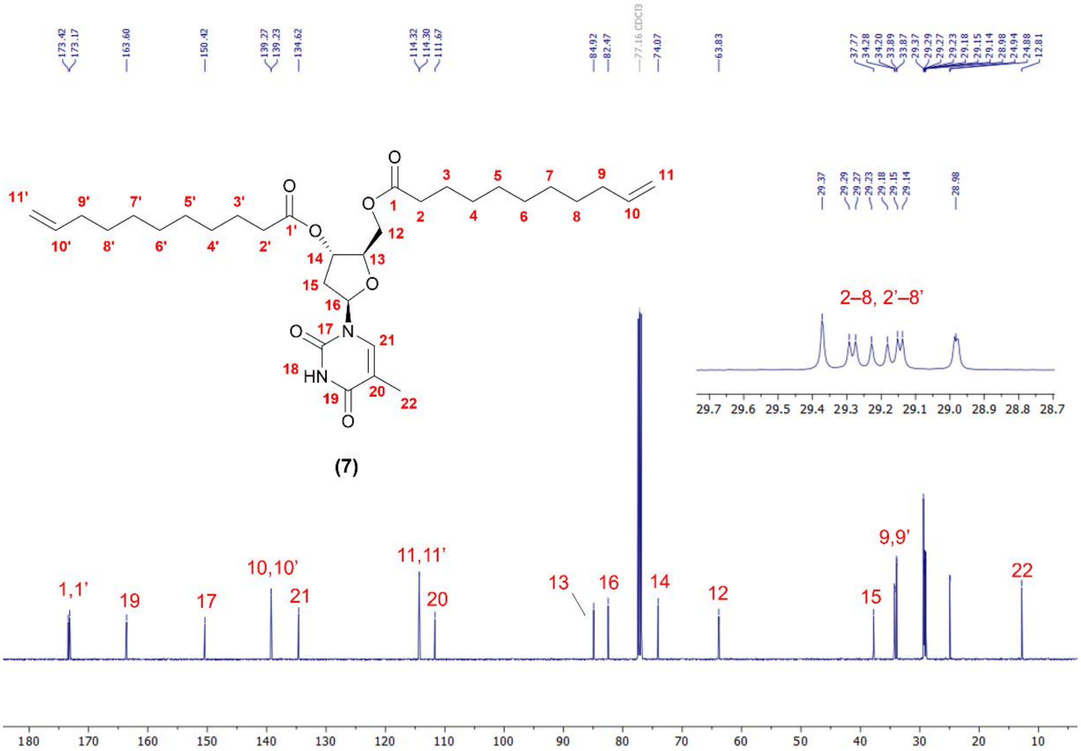

Figure S6.10: $^{13}C\{^1H\}$ NMR spectrum of monomer **7** in $CDCl_3$.

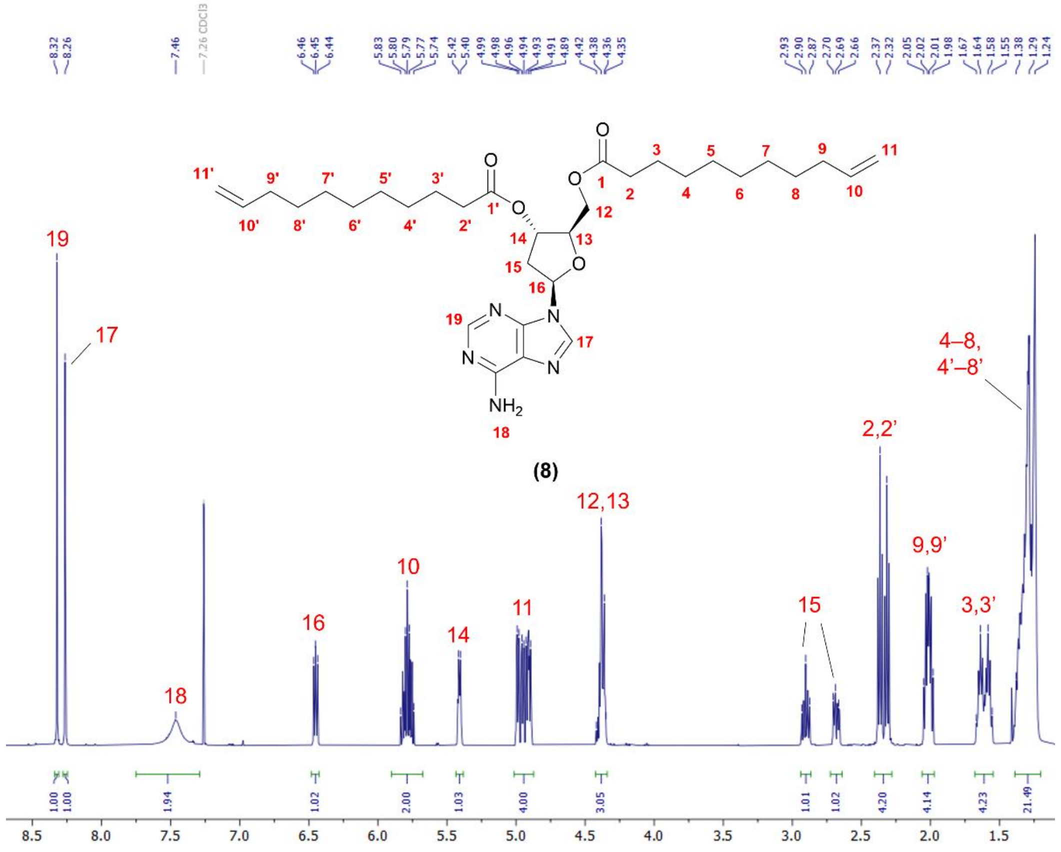

Figure S6.11: ^{1}H NMR spectrum of monomer **8** in $CDCl_3$.

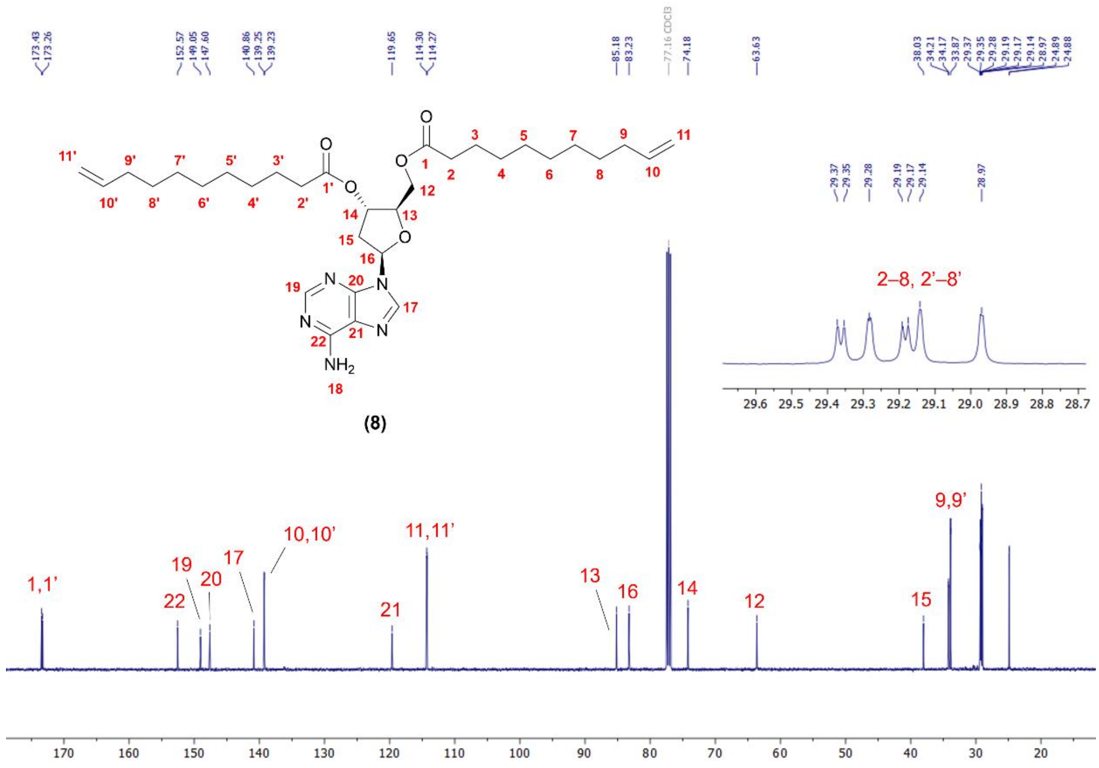

Figure S6.12: $^{13}C\{^{1}H\}$ NMR spectrum of monomer **8** in $CDCl_3$.

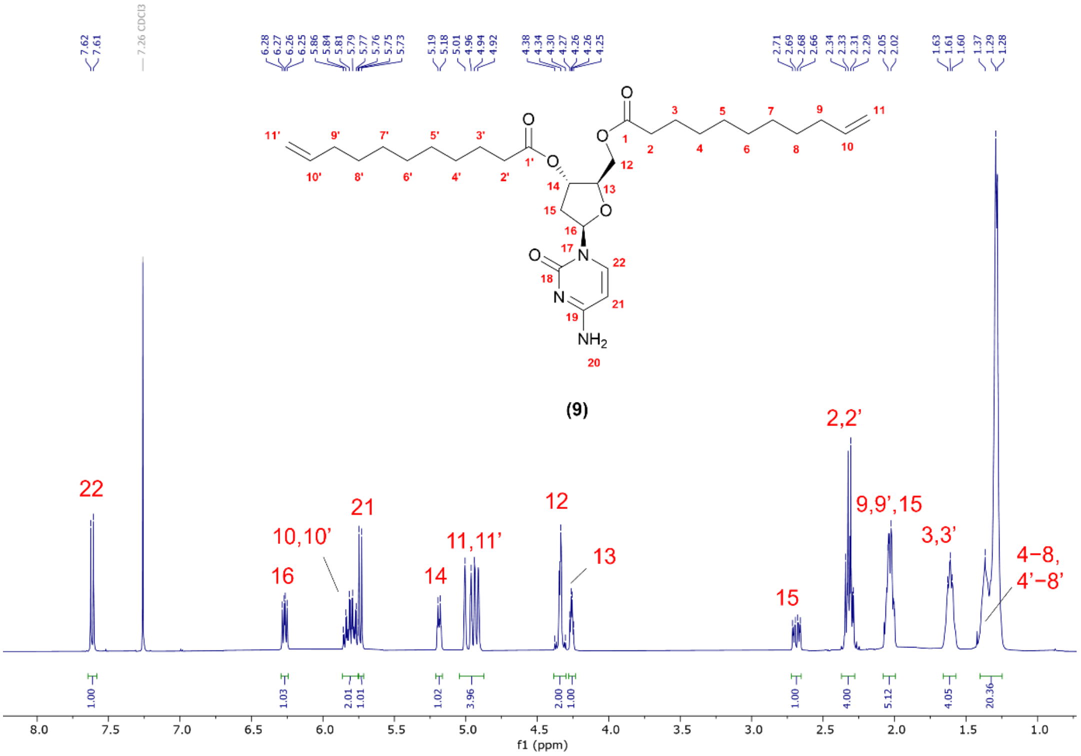

Figure S6.13: ^{1}H NMR spectrum of monomer **9** in $CDCl_3$.

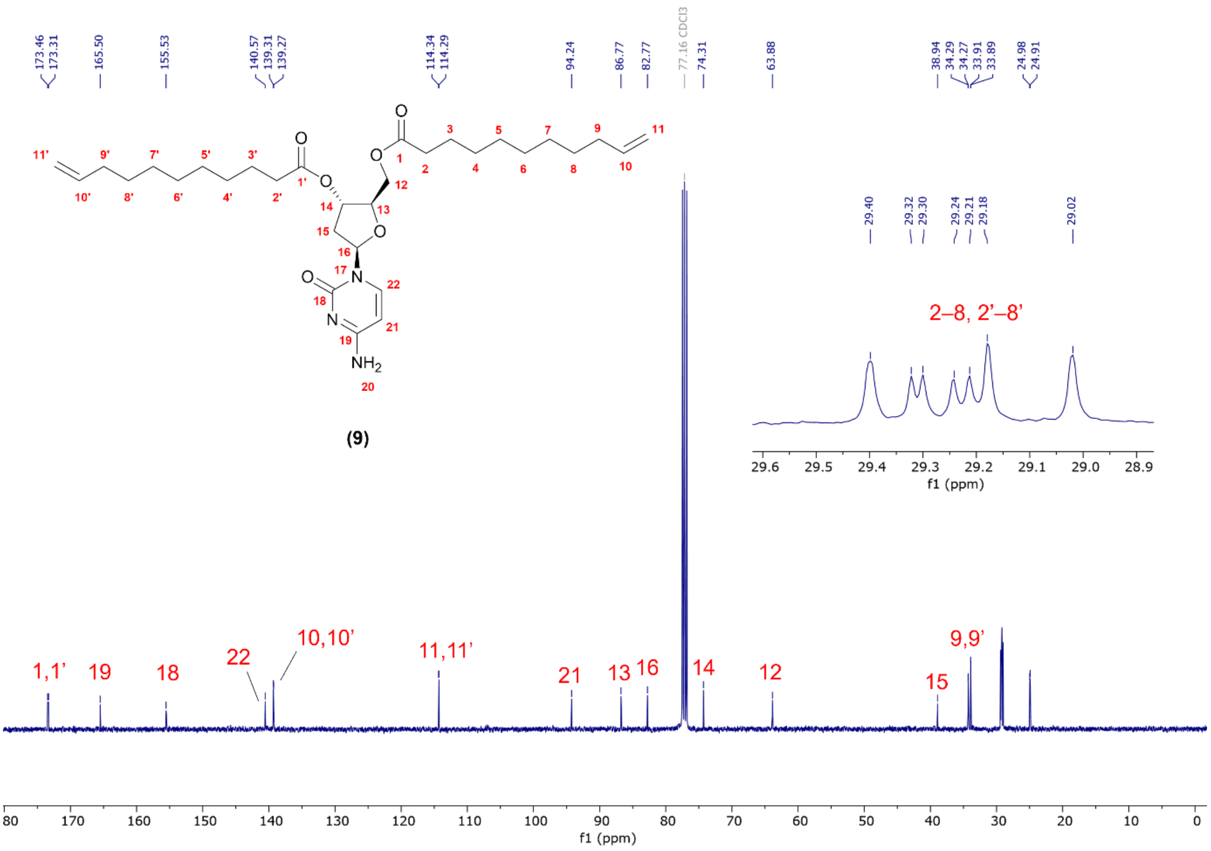

Figure S6.14: $^{13}C\{^1H\}$ NMR spectrum of monomer **9** in $CDCl_3$.

Co-polymers

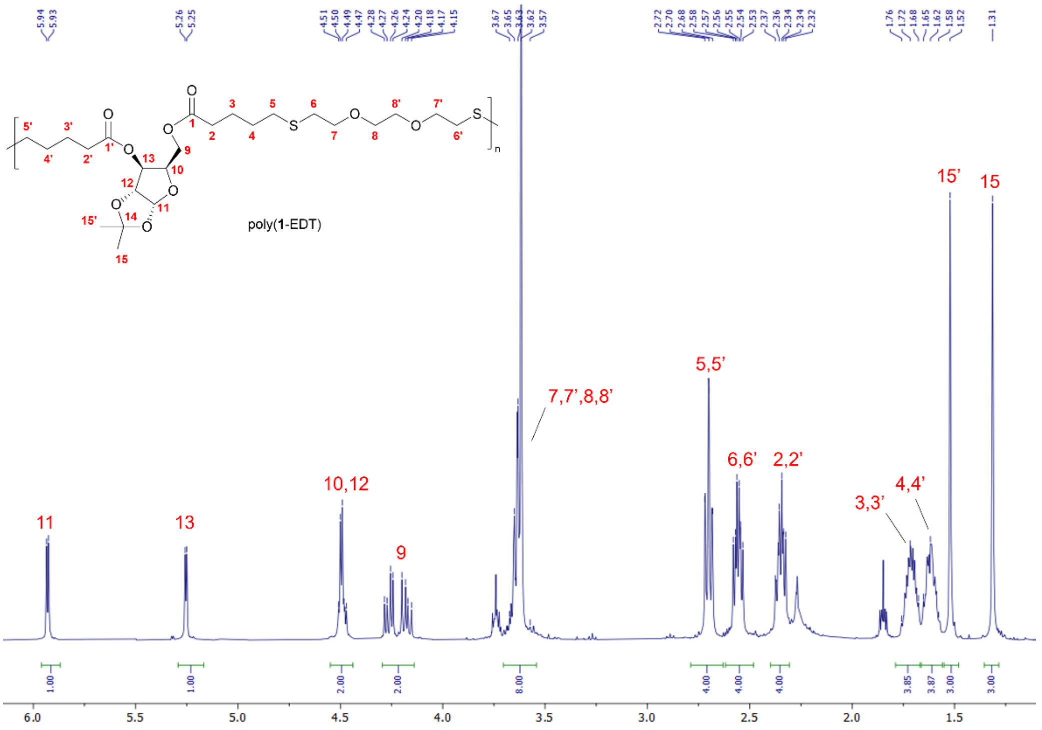

Figure S6.15: ^{1}H NMR spectrum of poly(**1**-EDT) in $CDCl_3$.

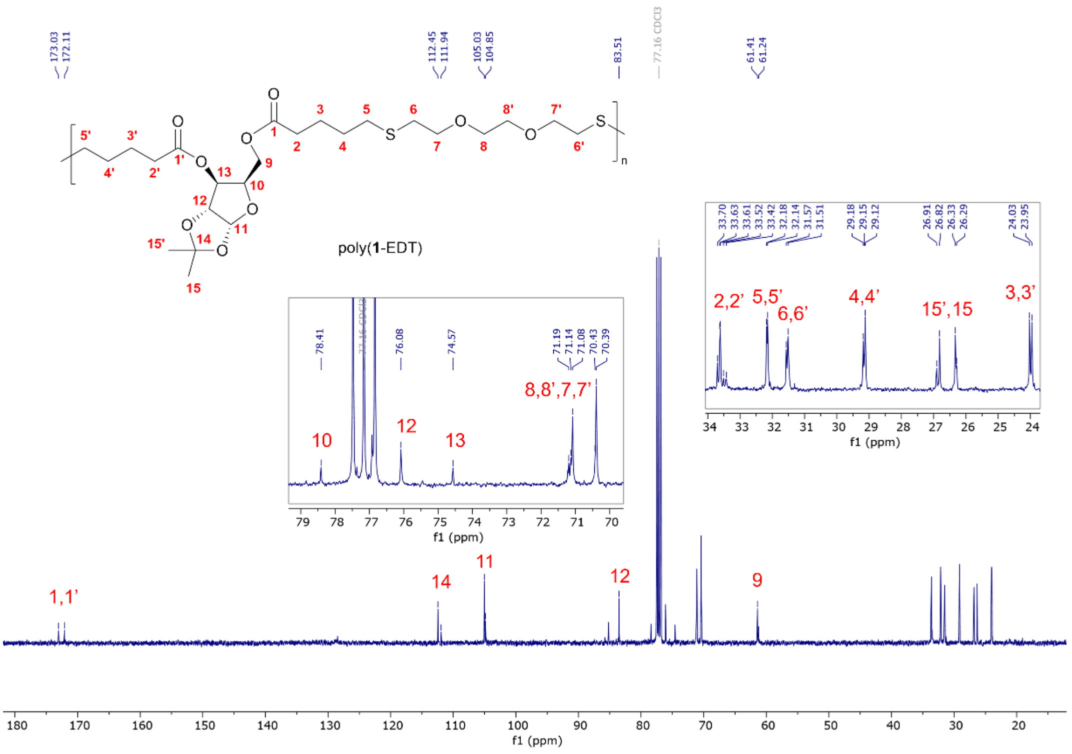

Figure S6.16: $^{13}C\{^{1}H\}$ NMR spectrum of poly(**1**-EDT) in $CDCl_3$.

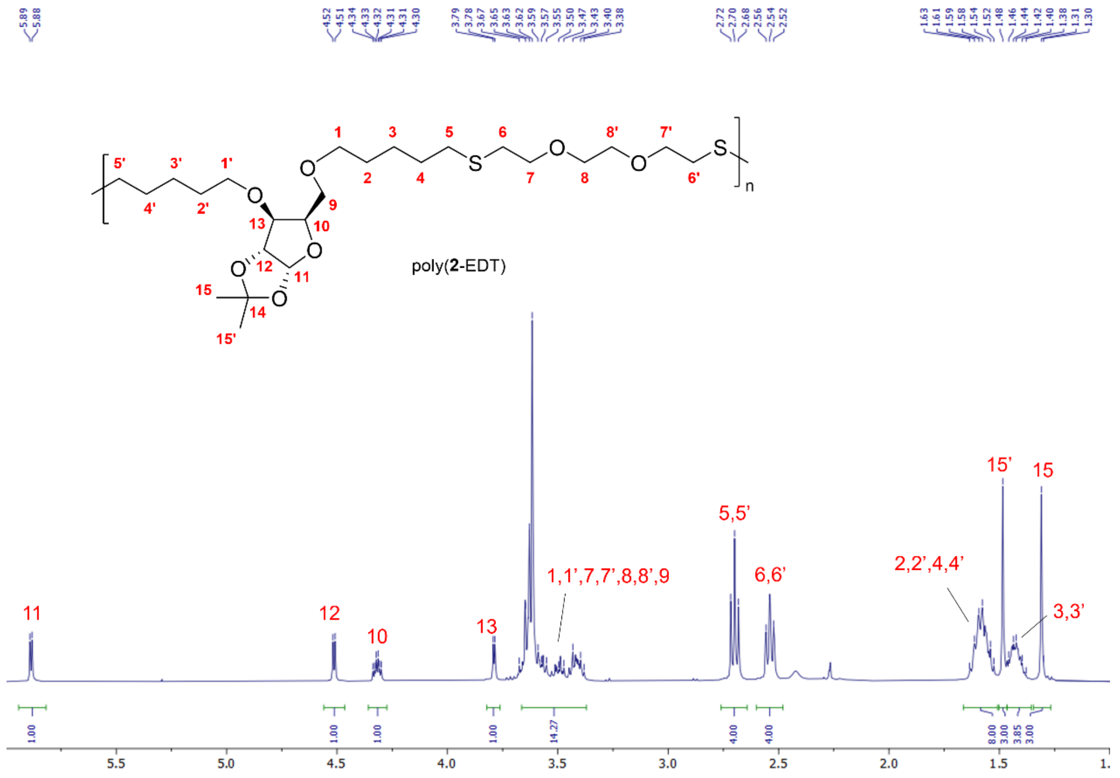

Figure S6.17: ^{1}H NMR spectrum of poly(**2**-EDT) in $CDCl_3$.

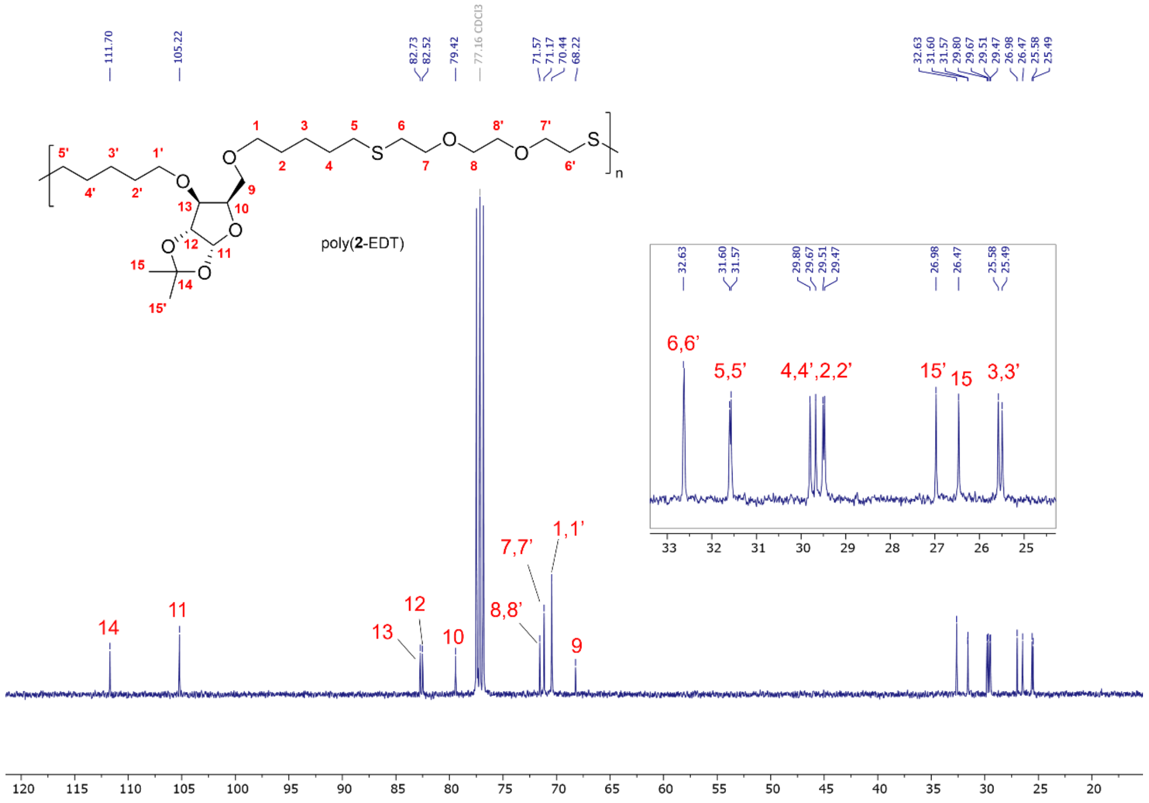

Figure S6.18: $^{13}C\{^{1}H\}$ NMR spectrum of poly(**2**-EDT) in $CDCl_3$.

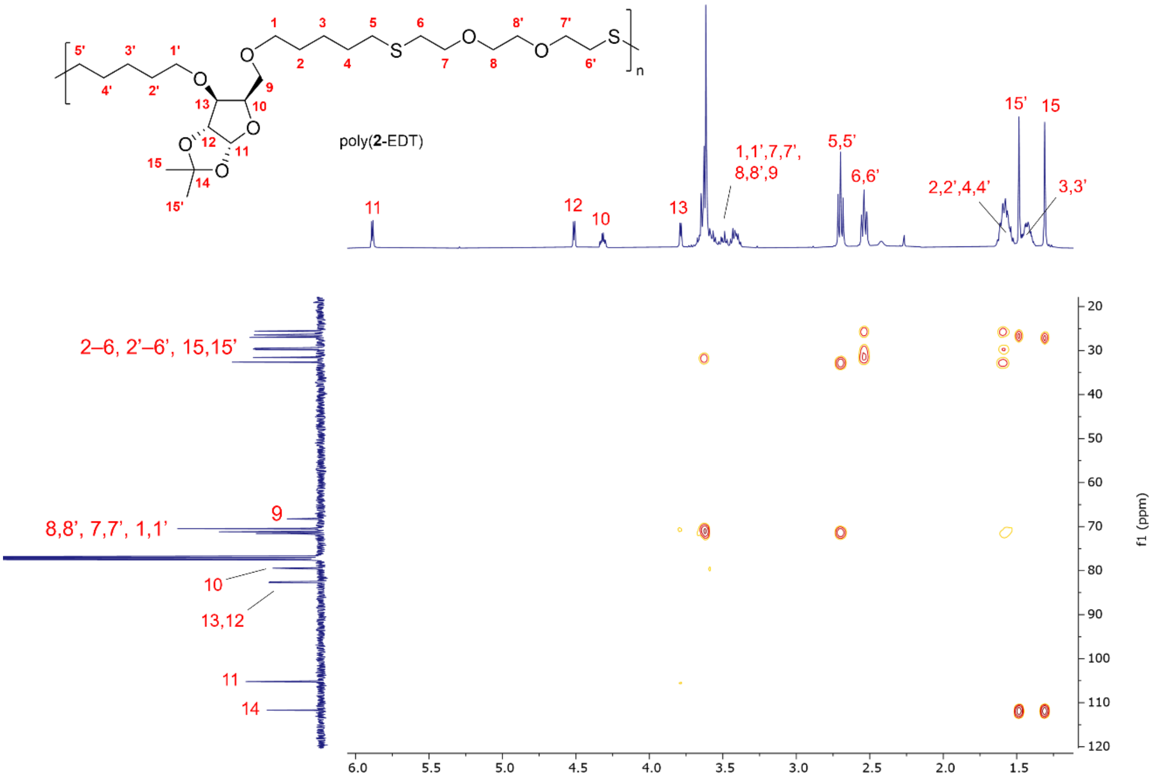

Figure S6.19: ^{1}H-^{13}C HMBC NMR spectrum of poly(**2**-EDT) in $CDCl_3$.

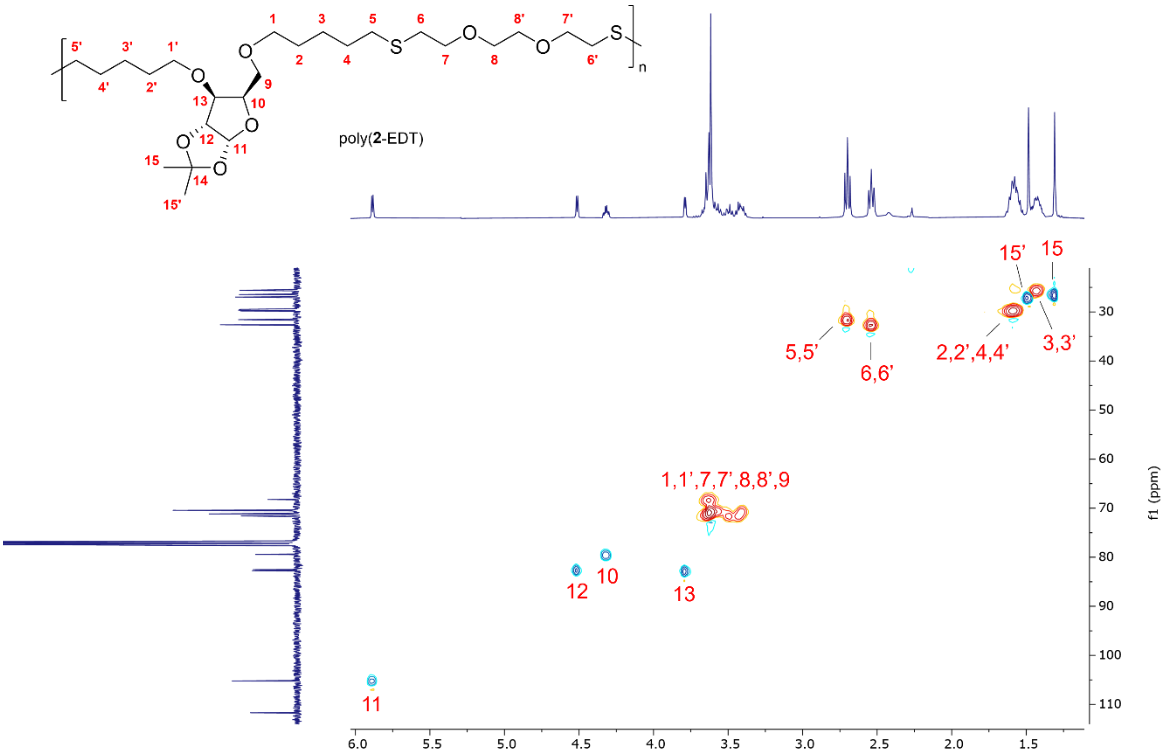

Figure S6.20: ^{1}H-^{13}C HSQC NMR spectrum of poly(**2**-EDT) in $CDCl_3$. Blue and red correlations correspond to CH_2 and CH or CH_3 signals, respectively.

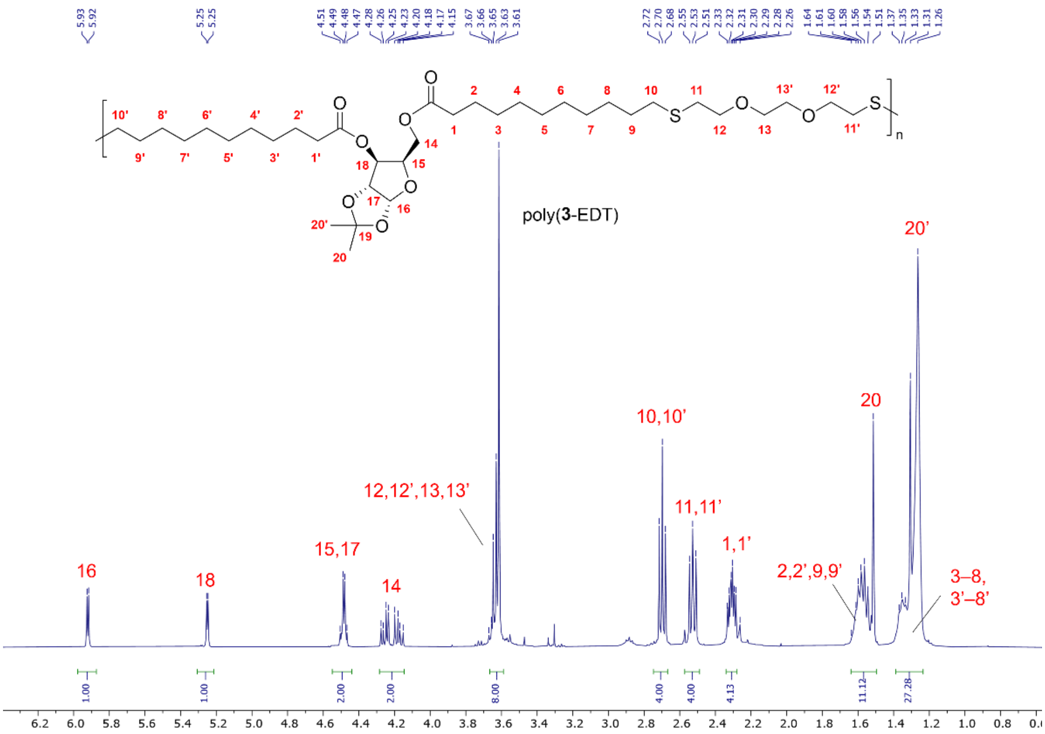

Figure S6.21: ^{1}H NMR spectrum of poly(**3**-EDT) in $CDCl_3$.

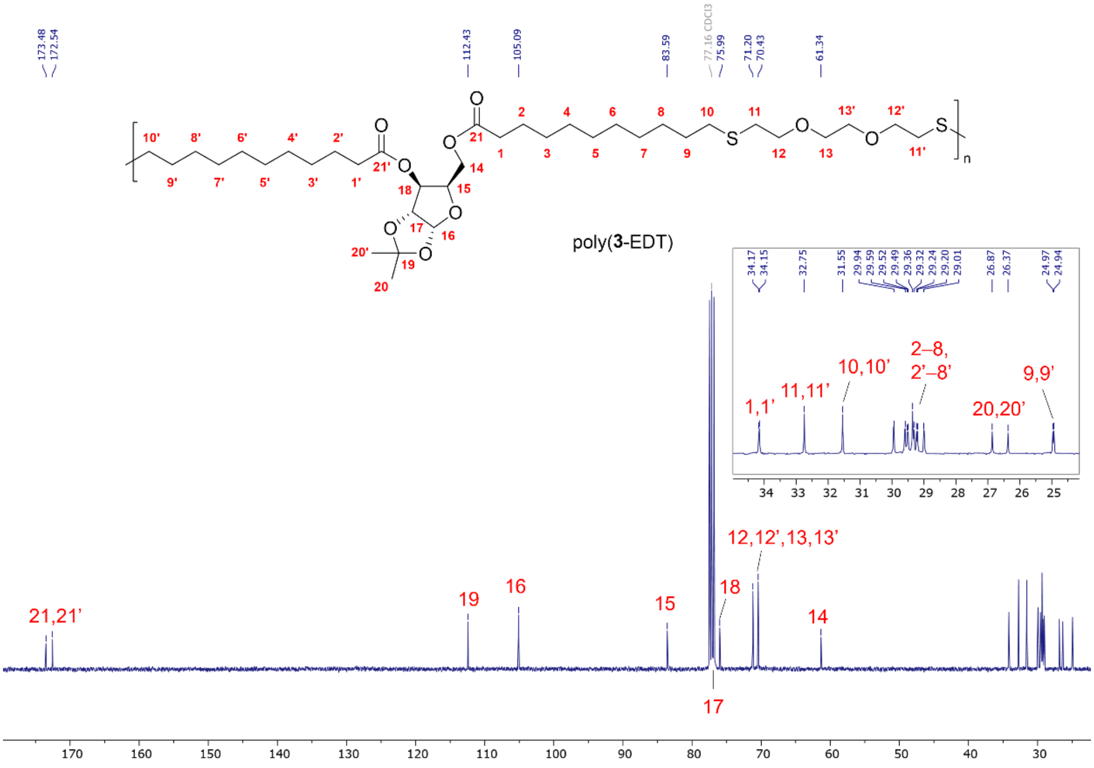

Figure S6.22: $^{13}C\{^1H\}$ NMR spectrum of poly(**3**-EDT) in $CDCl_3$.

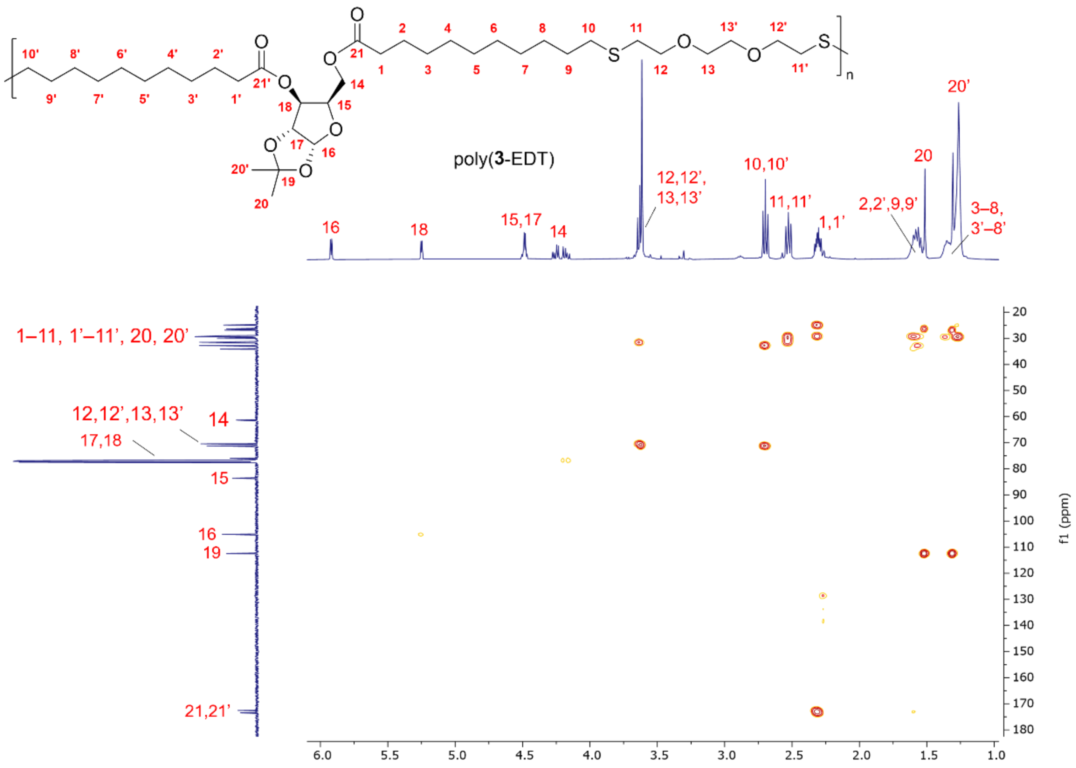

Figure S6.23: ^{1}H-^{13}C HMBC NMR spectrum of poly(**3**-EDT) in $CDCl_3$.

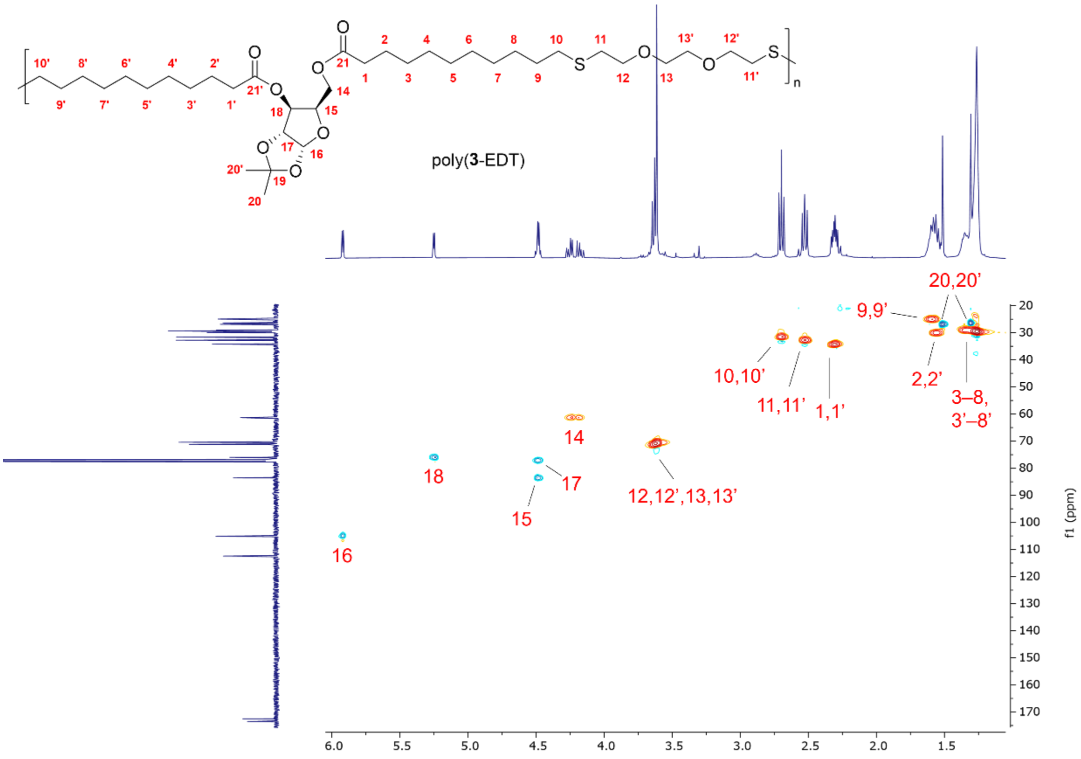

Figure S6.24: ^{1}H-^{13}C HSQC NMR spectrum of poly(**3**-EDT) in $CDCl_3$. Blue and red correlations correspond to CH_2 and CH or CH_3 signals, respectively.

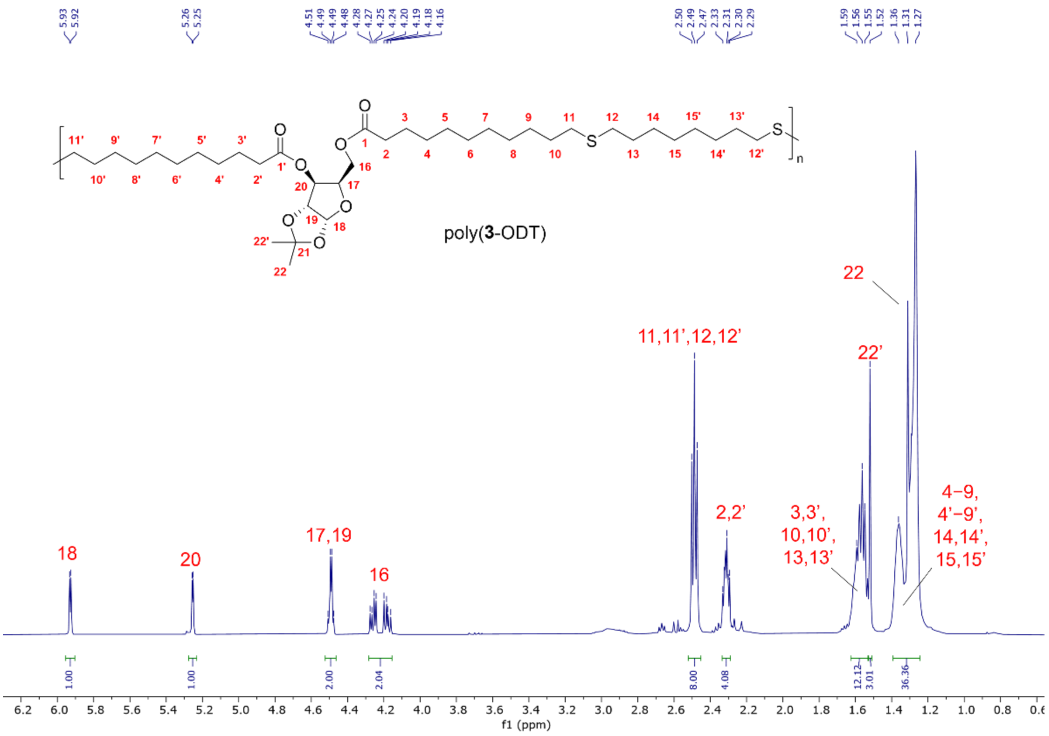

Figure S6.25: ^{1}H NMR spectrum of poly(**3**-ODT) in $CDCl_3$.

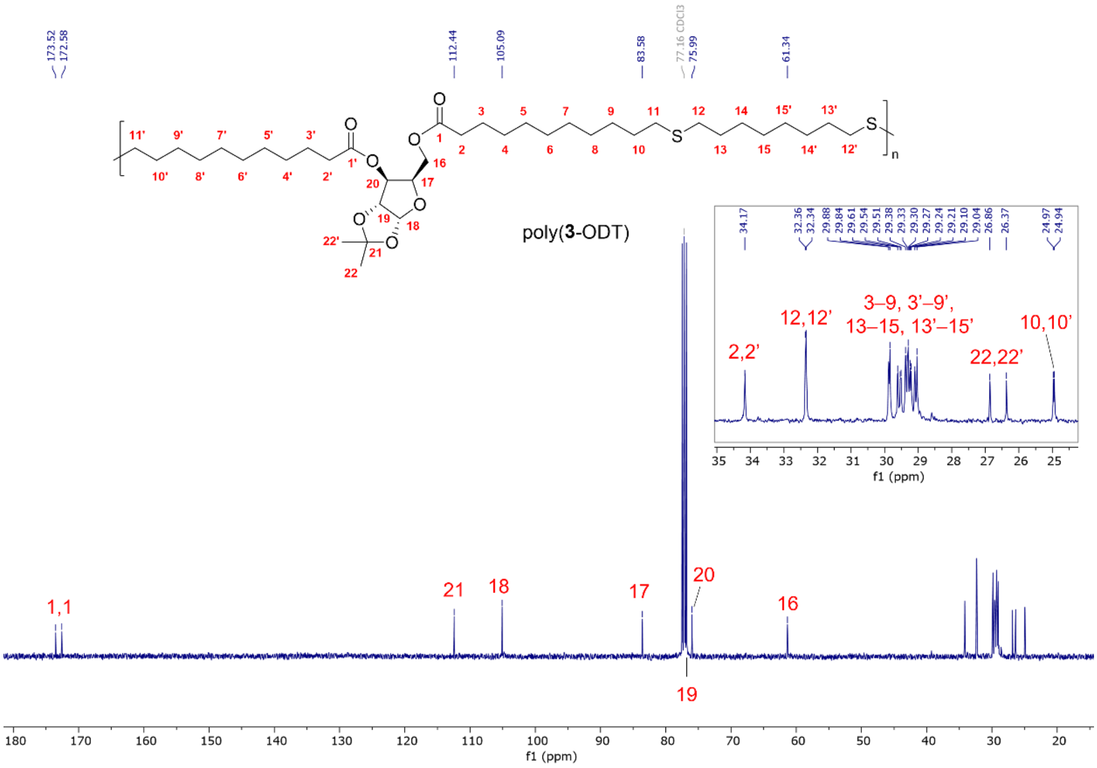

Figure S6.26: $^{13}C\{^1H\}$ NMR spectrum of poly(**3**-ODT) in $CDCl_3$.

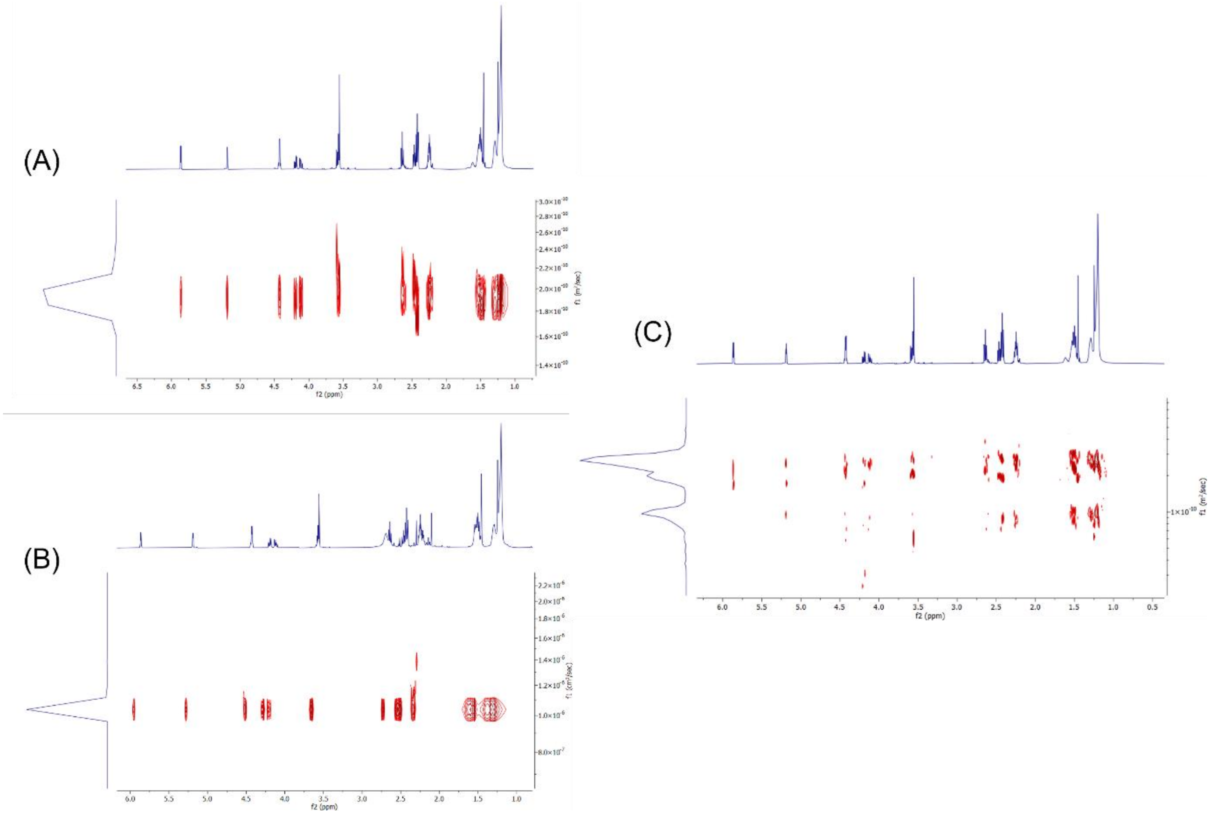

Figure S6.27: ^{1}H NMR spectrum of poly((**3**-EDT)-*b*-(**3**-ODT)) prepared with preformed **3**-EDT blocks (A) or with both block preformed (B), confirming the presence of a single species diffusing at the same rate (diffusion coefficients = 2.0×10^{-10} m^2 s^{-1} and 1.9×10^{-10} m^2 s^{-1}, respectively). (C) ^{1}H NMR spectrum of poly((**3**-EDT)-*b*-(**3**-ODT)) spiked with the **3**-EDT pre-polymer block. The spectrum clearly shows two different polymeric species (diffusion coefficients = 2.7×10^{-10} m^2 s^{-1} and 9.7×10^{-11} m^2 s^{-1}), thus confirming that poly((**3**-EDT)-*b*-(**3**-ODT)) is a true co-polymer.

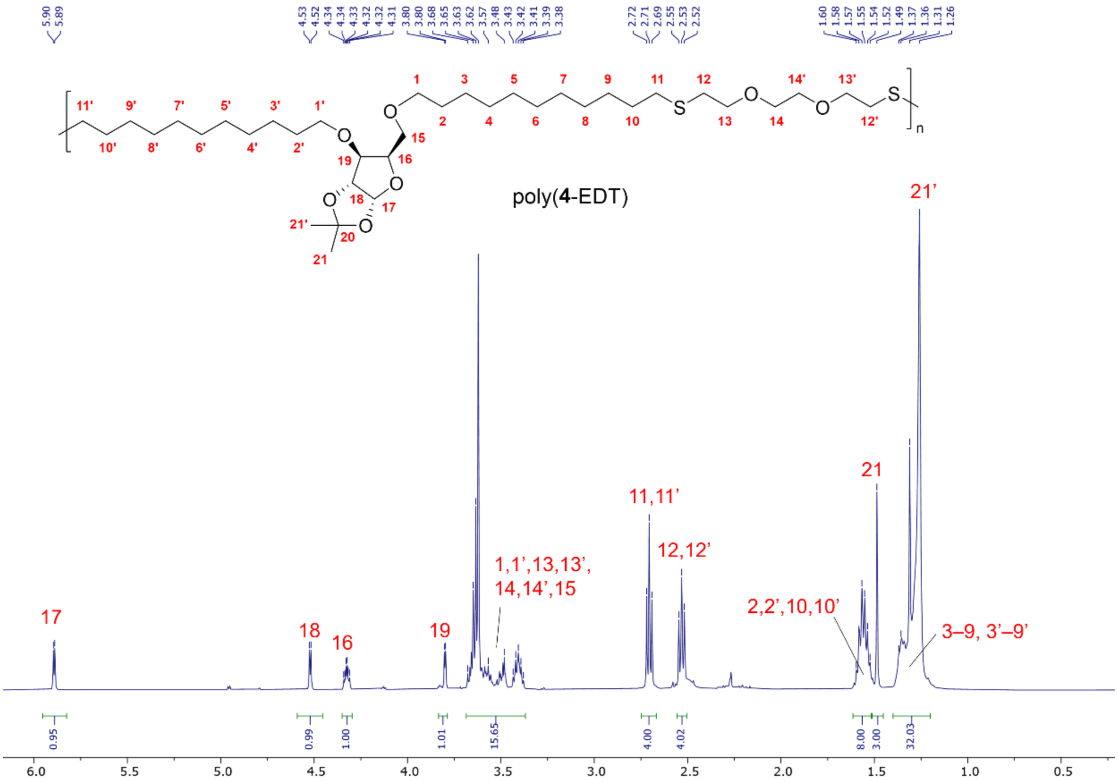

Figure S6.28: ^{1}H NMR spectrum of poly(**4**-EDT) in $CDCl_3$.

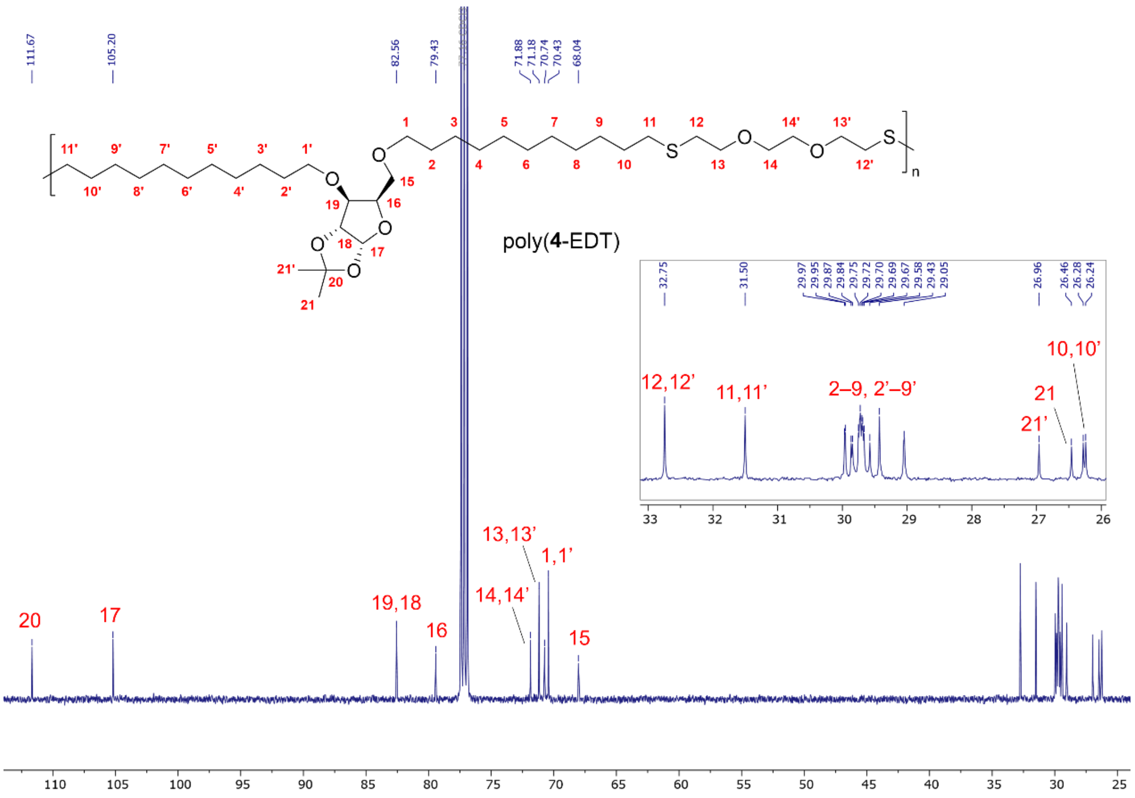

Figure S6.29: $^{13}C\{^1H\}$ NMR spectrum of poly(**4**-EDT) in $CDCl_3$.

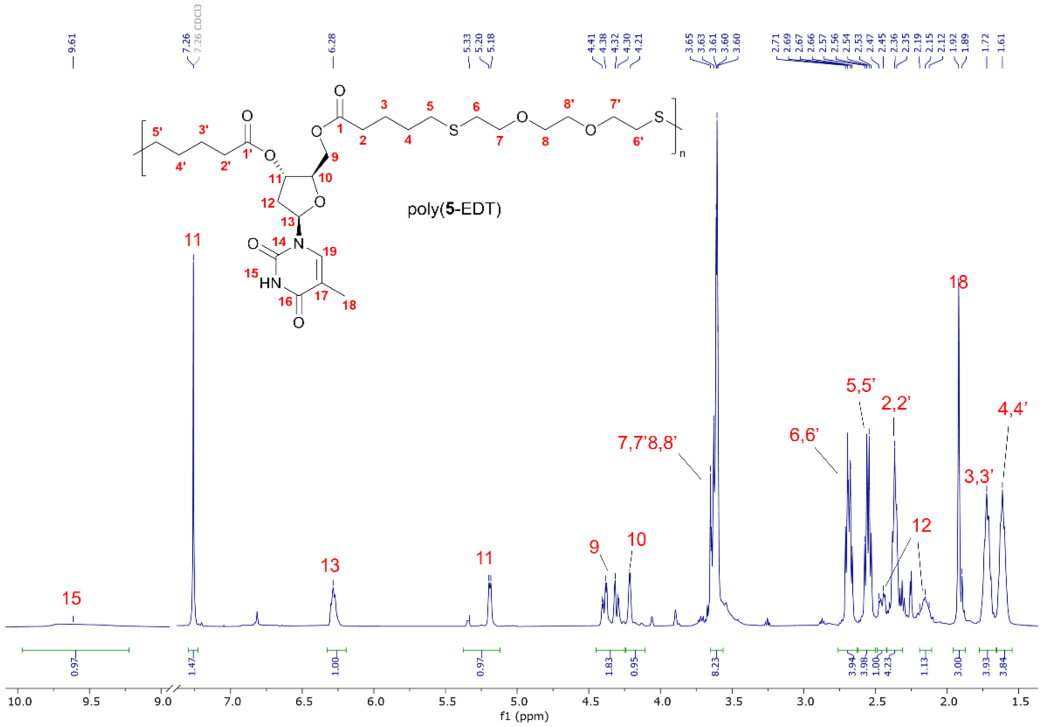

Figure S6.30: ^1H NMR spectrum of poly(**5**-EDT) in $CDCl_3$.

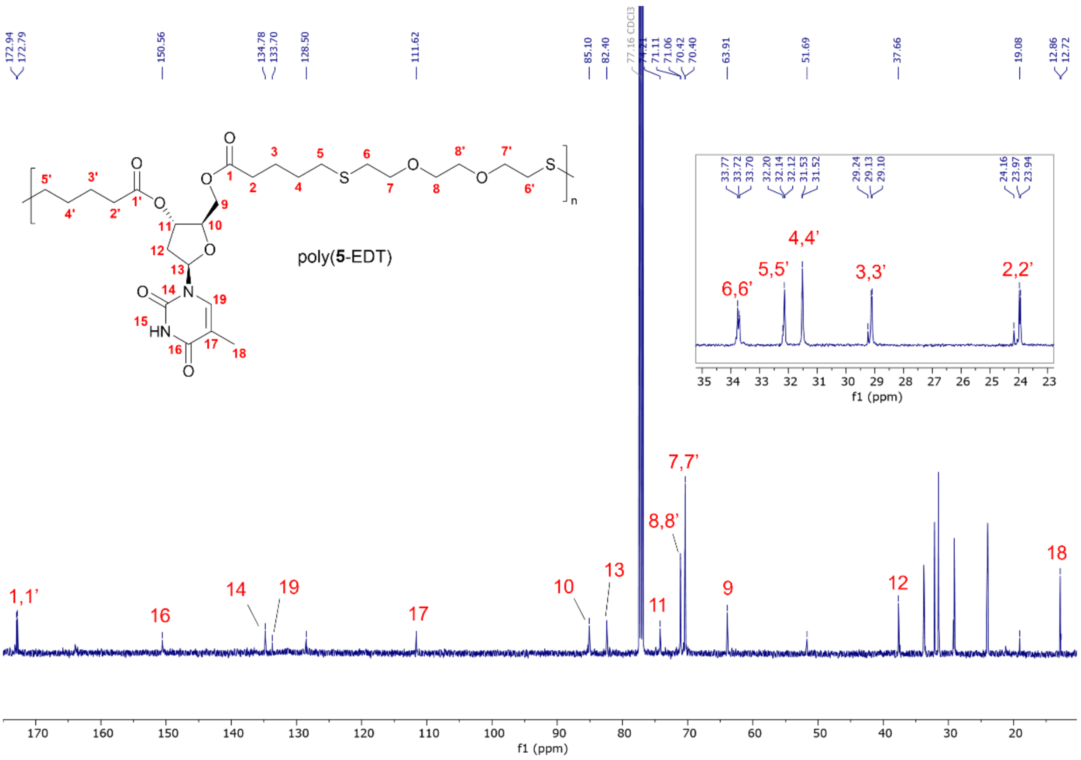

Figure S6.31: $^{13}C\{^{1}H\}$ NMR spectrum of poly(**5**-EDT) in $CDCl_3$.

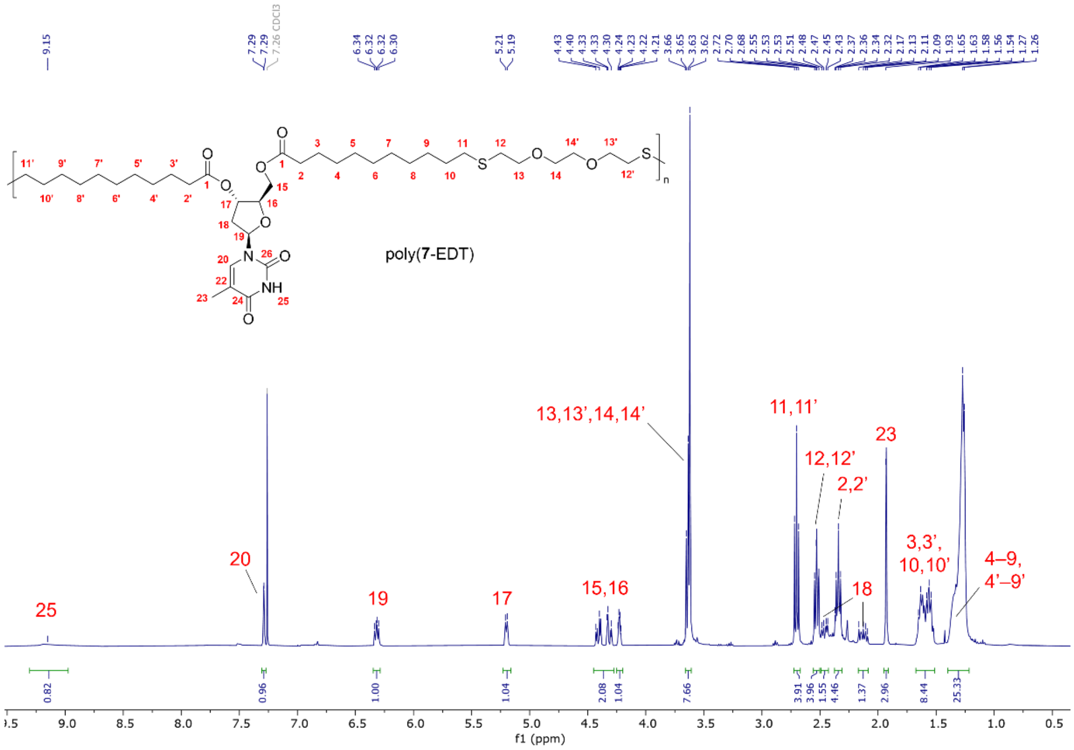

Figure S6.32: ^{1}H NMR spectrum of poly(**7**-EDT) in $CDCl_3$.

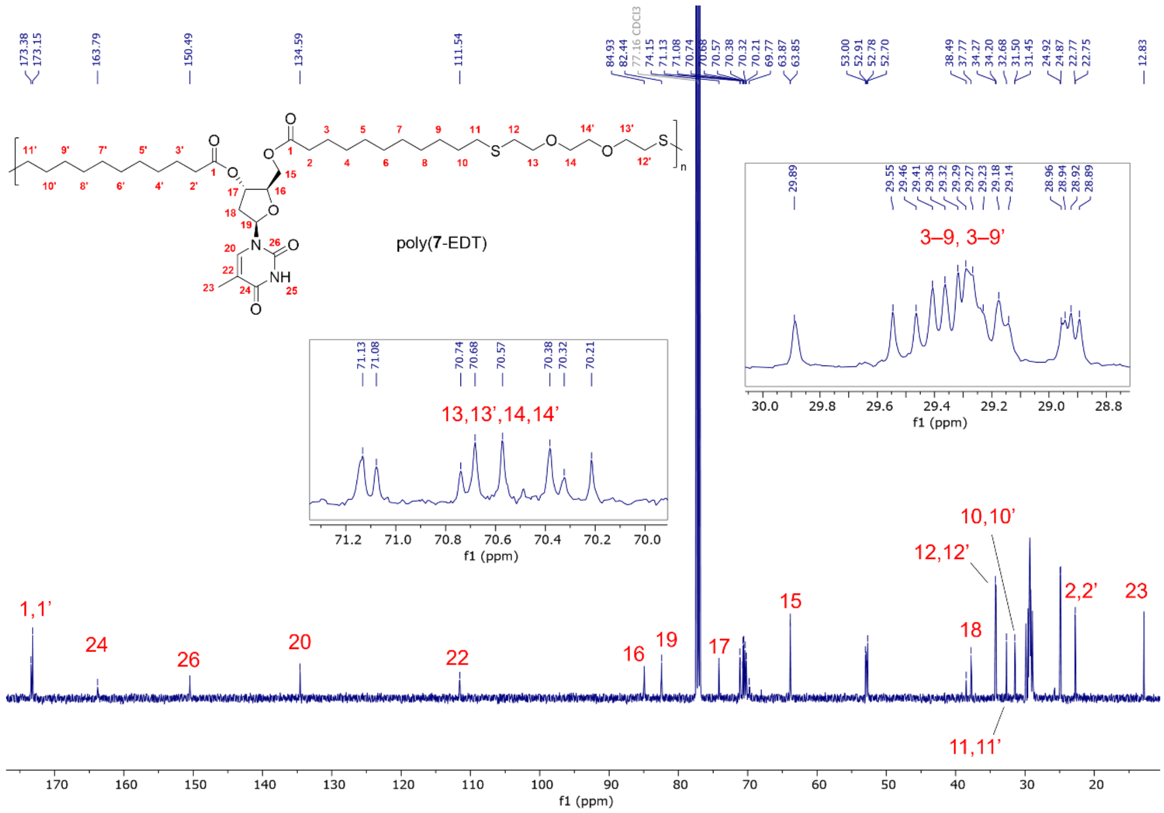

Figure S6.33: $^{13}C\{^1H\}$ NMR spectrum of poly(**7**-EDT) in $CDCl_3$.

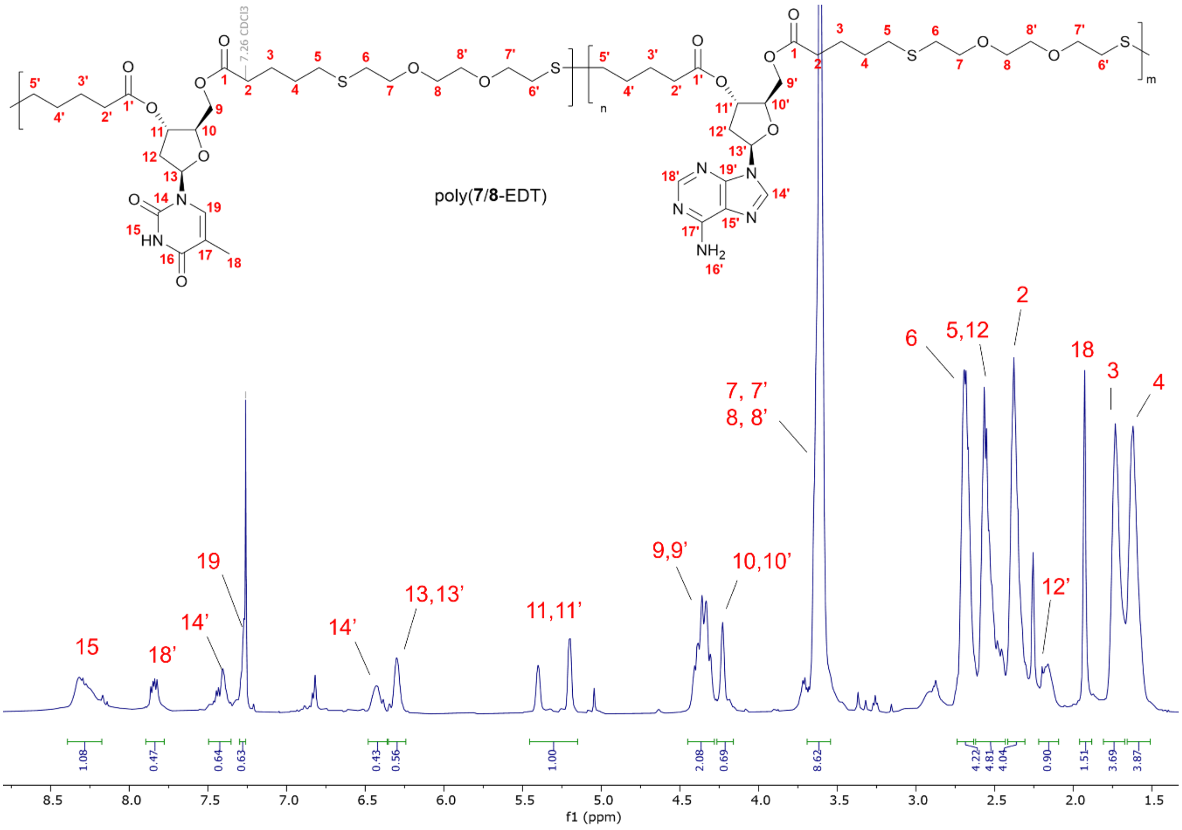

Figure S6.34: ^1H NMR spectrum of poly(**7**/**8**-EDT) in $CDCl_3$.

7. FTIR Spectra

Monomers

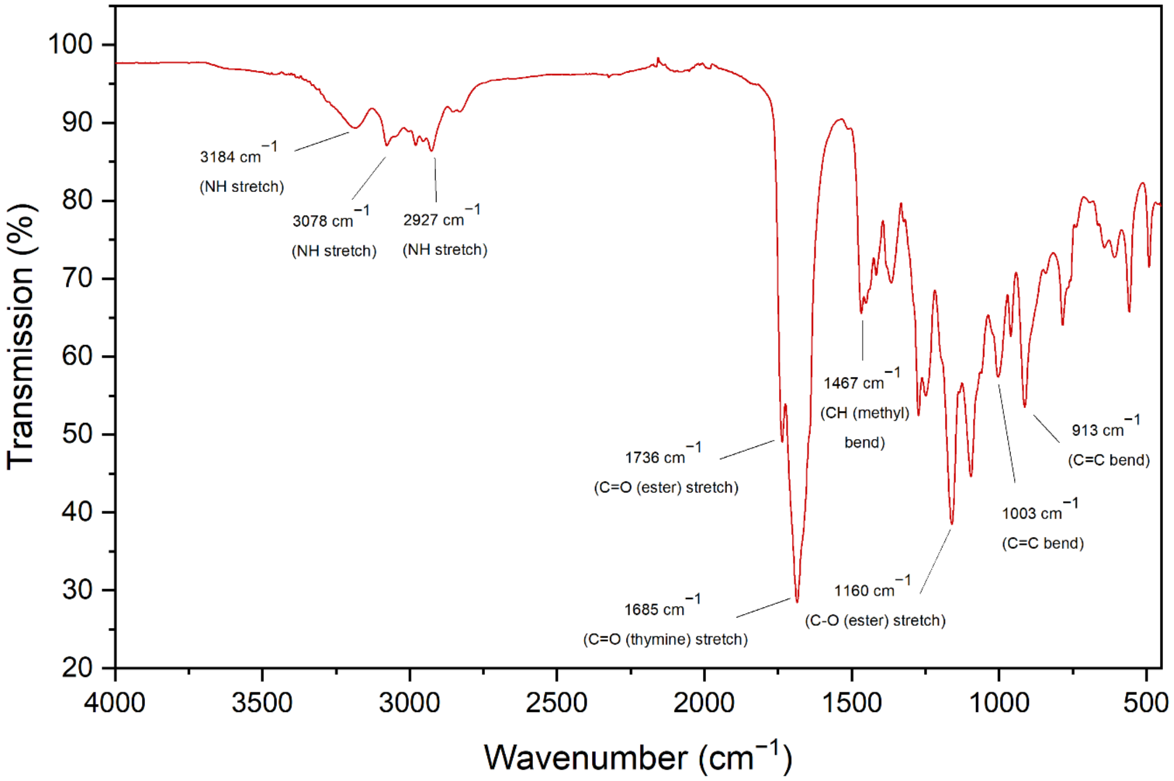

Figure S7.1: FTIR spectrum of monomer **5**.

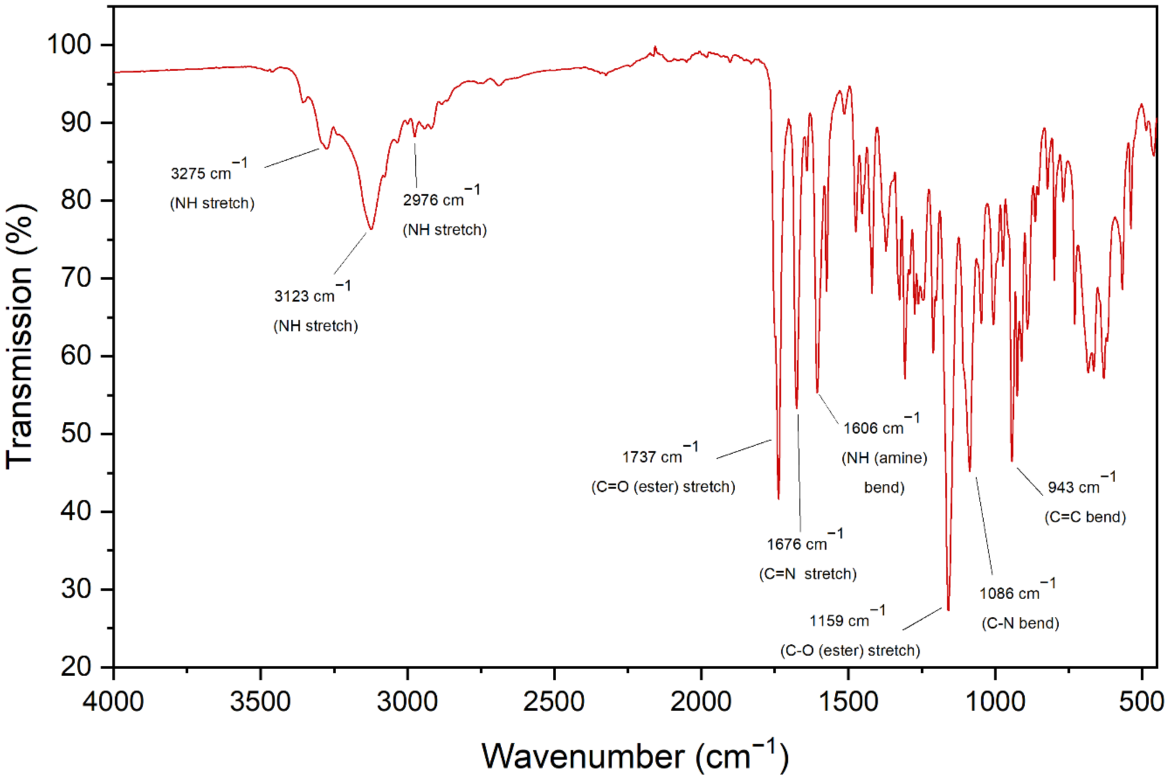

Figure S7.2: FTIR spectrum of monomer **6**.

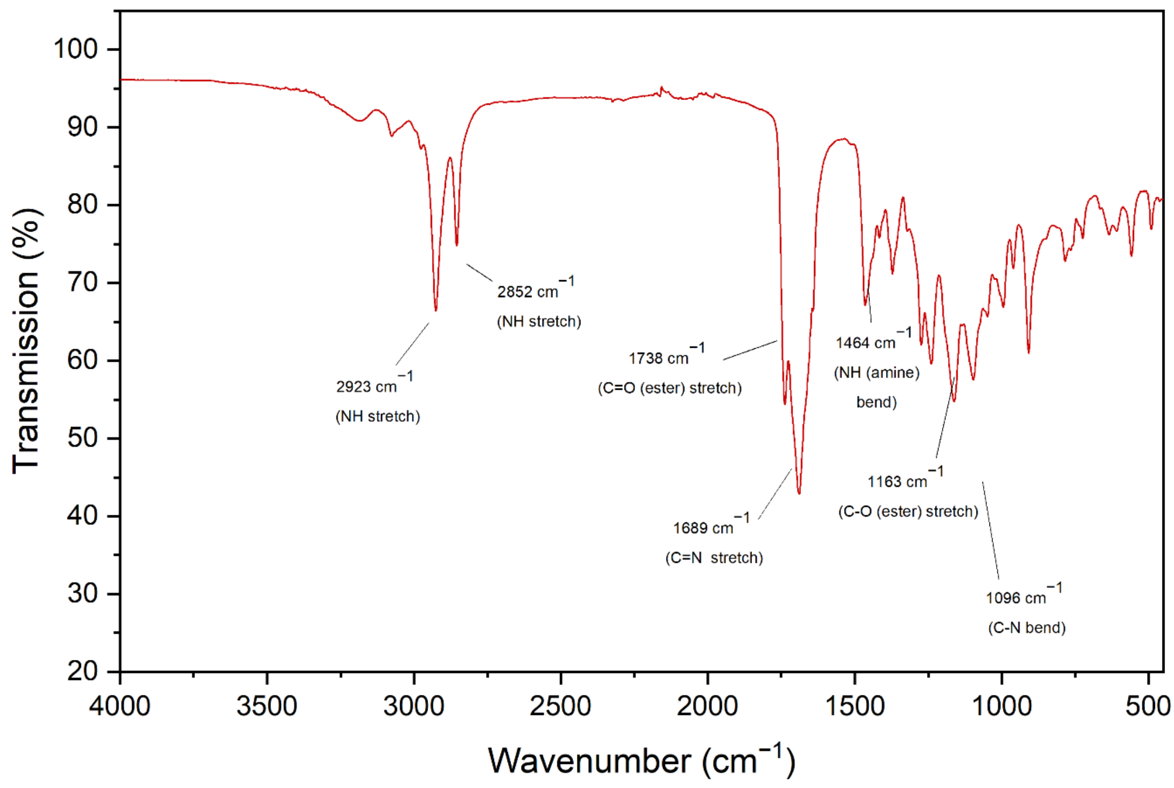

Figure S7.3: FTIR spectrum of monomer **7**.

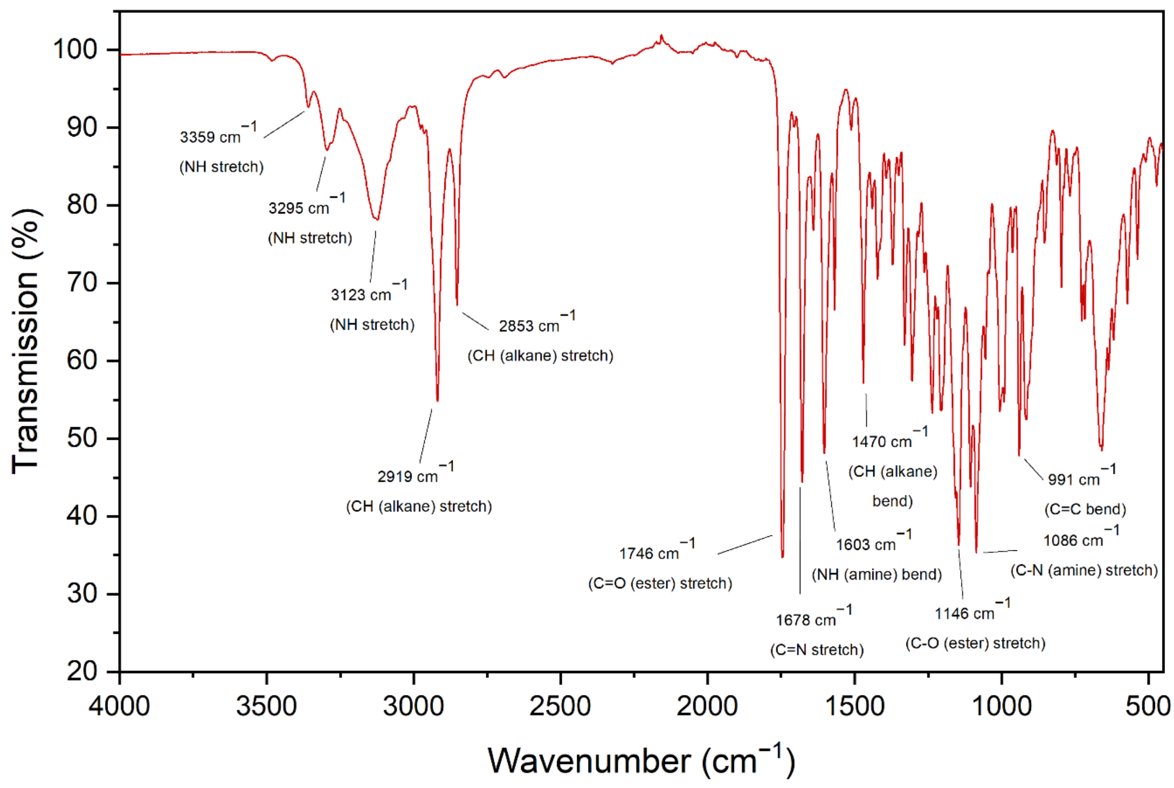

Figure S7.4: FTIR spectrum of monomer **8**.

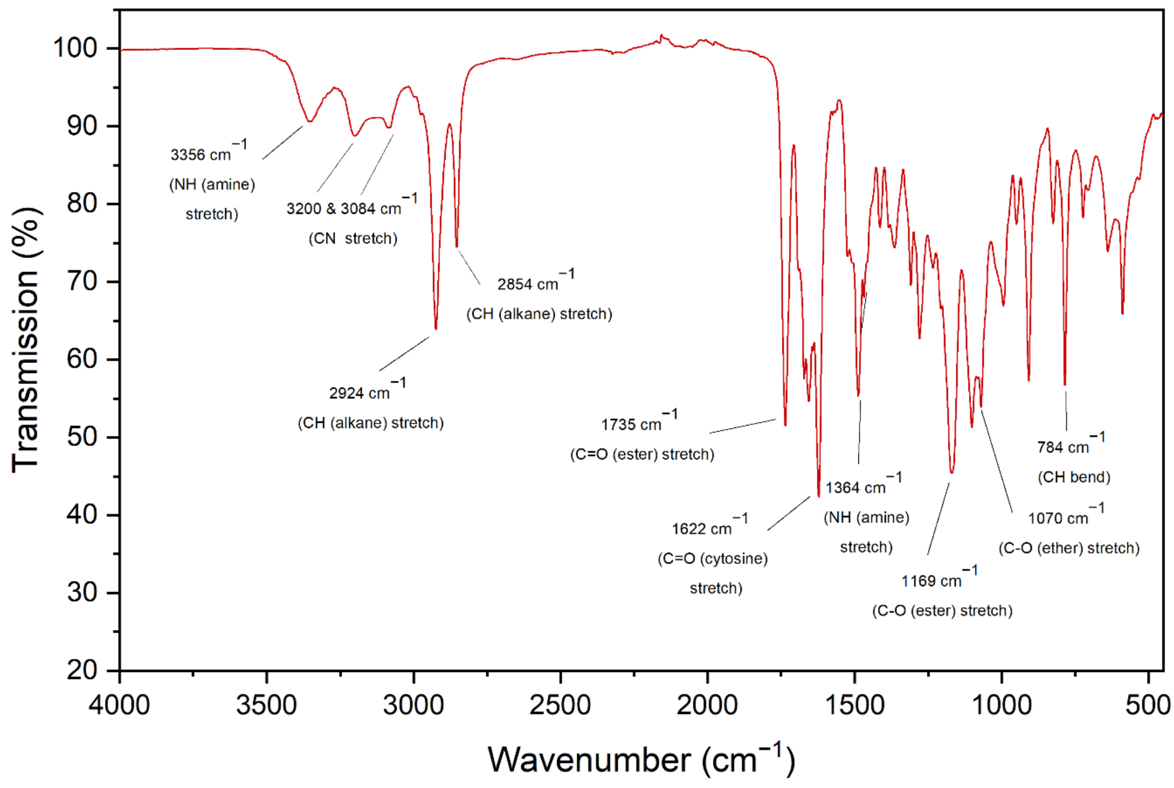

Figure S7.5: FTIR spectrum of monomer **9**.

Alternating Thiol-ene Co-polymers

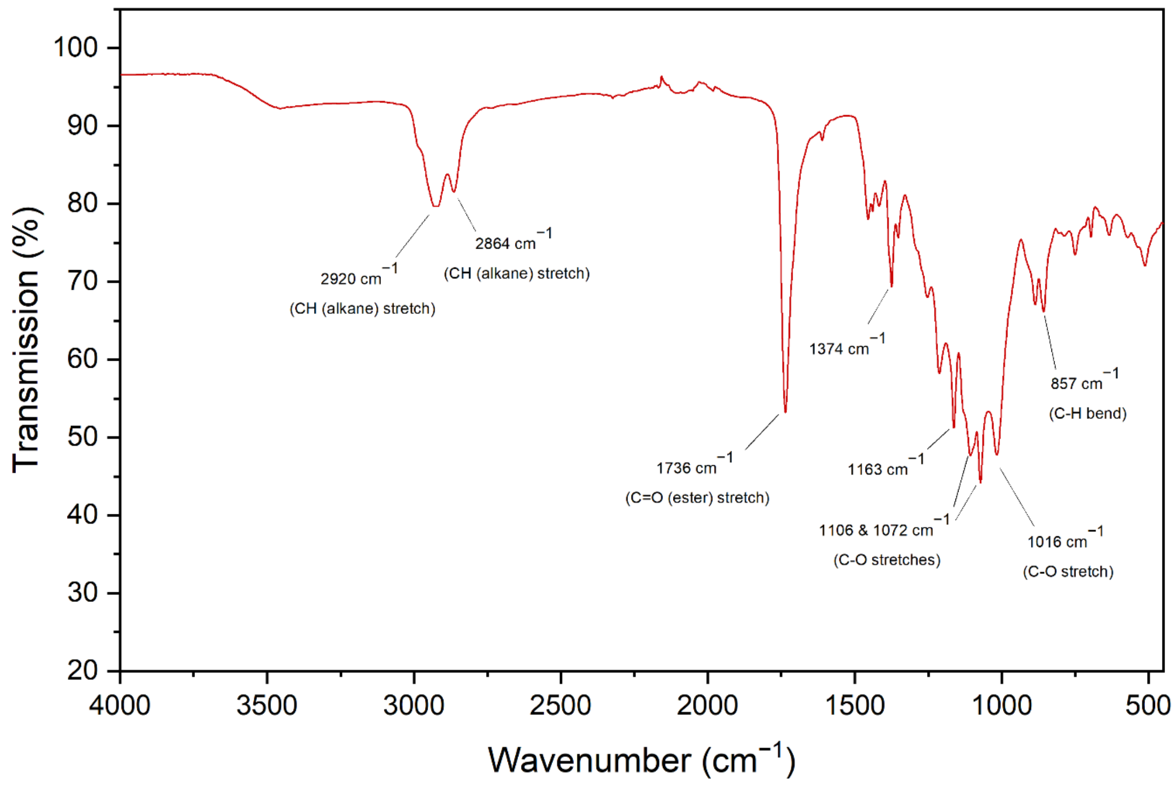

Figure S7.6: FTIR spectrum of poly(**1**-EDT).

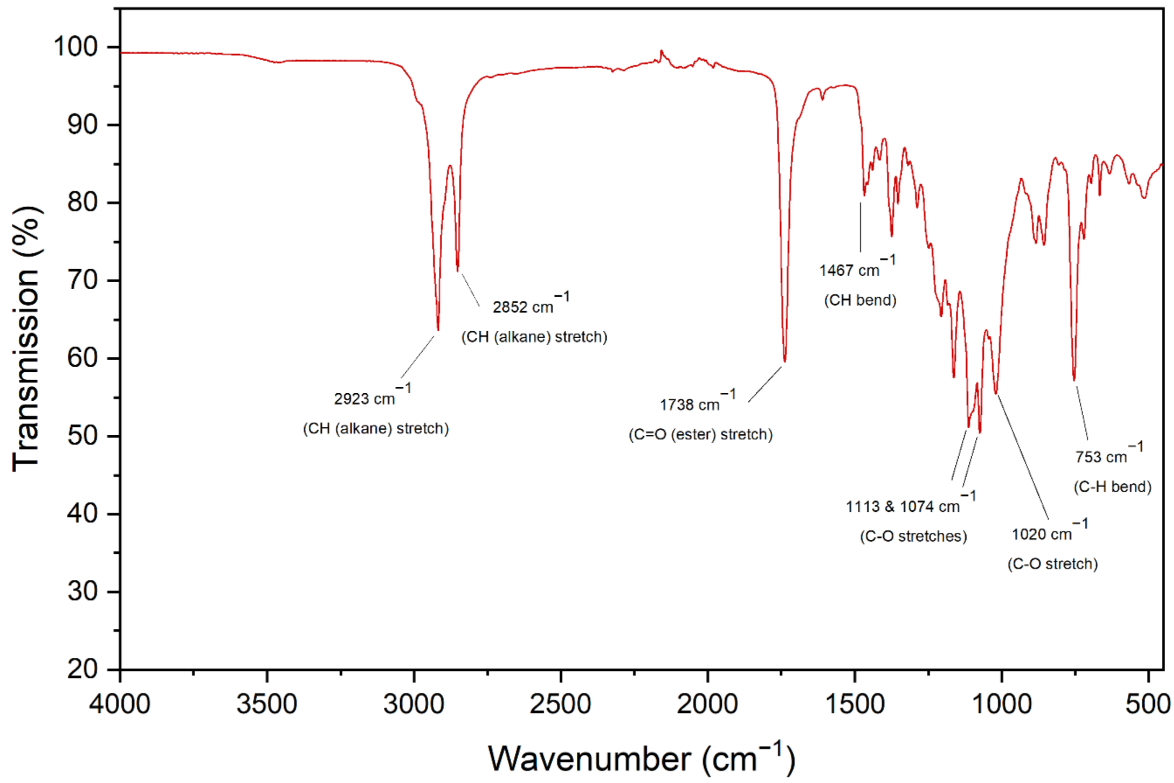

Figure S7.7: FTIR spectrum of poly(**3**-EDT).

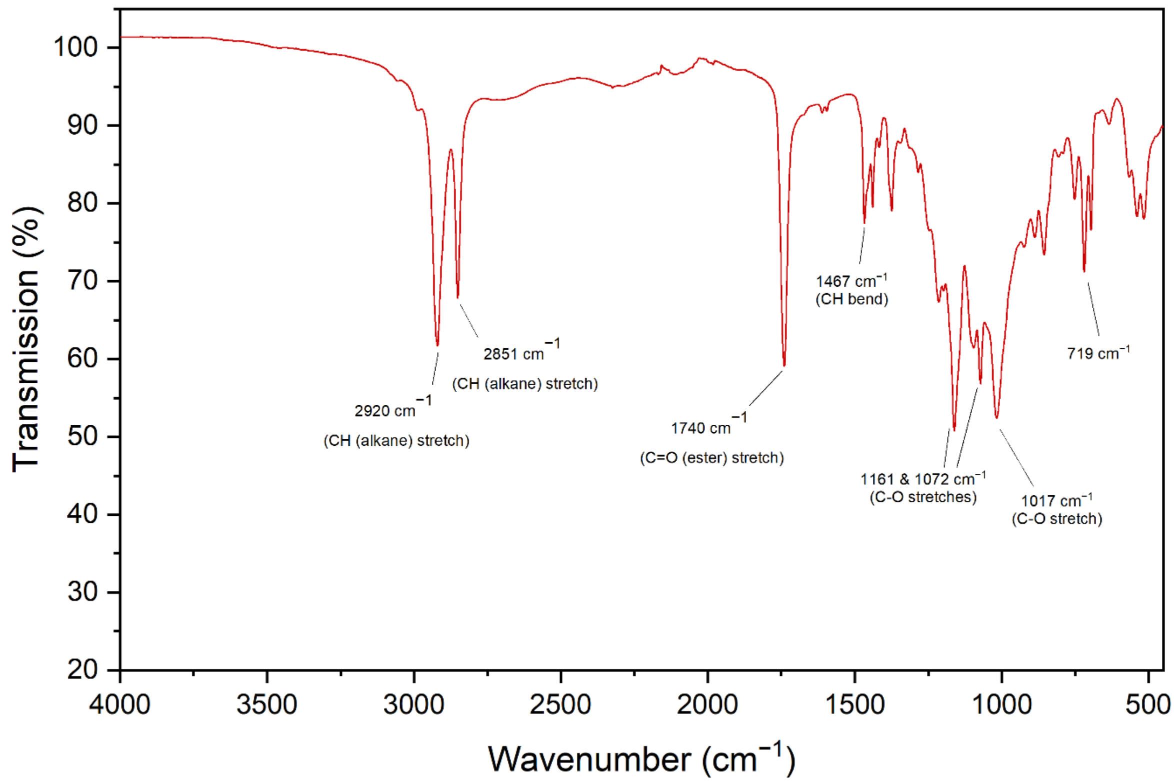

Figure S7.8: FTIR spectrum of poly(**3**-ODT).

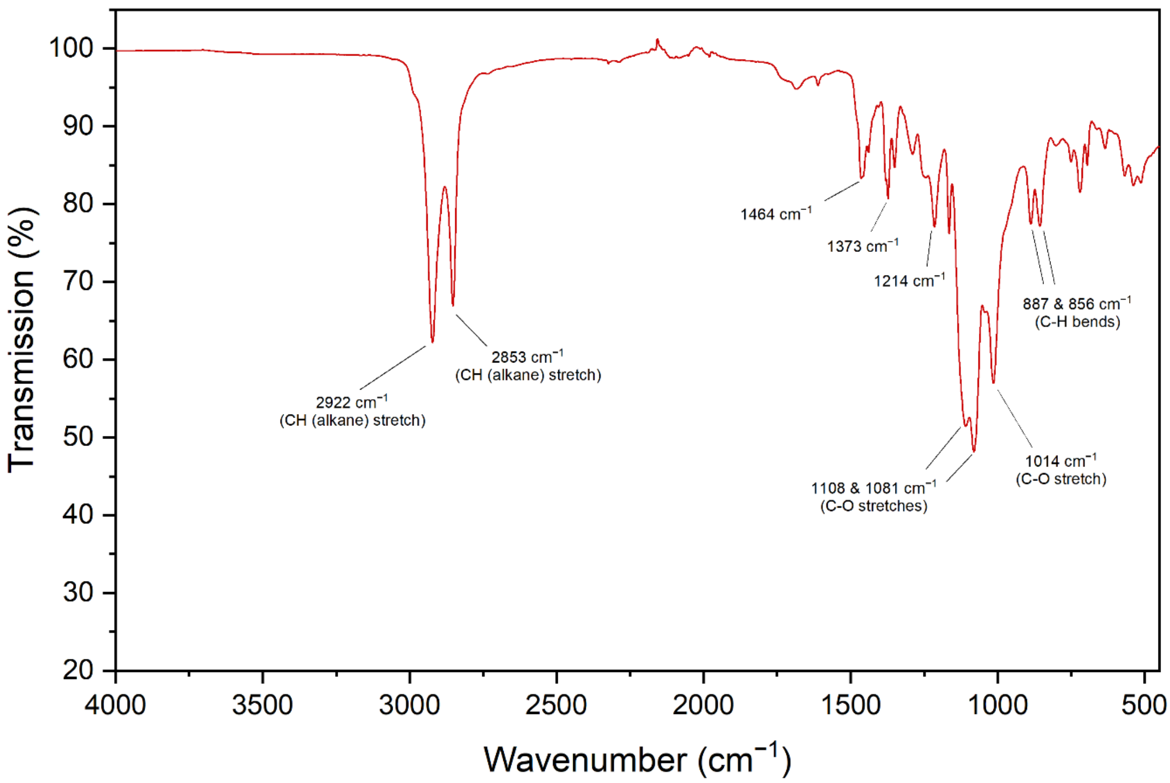

Figure S7.9: FTIR spectrum of poly(**4**-EDT).

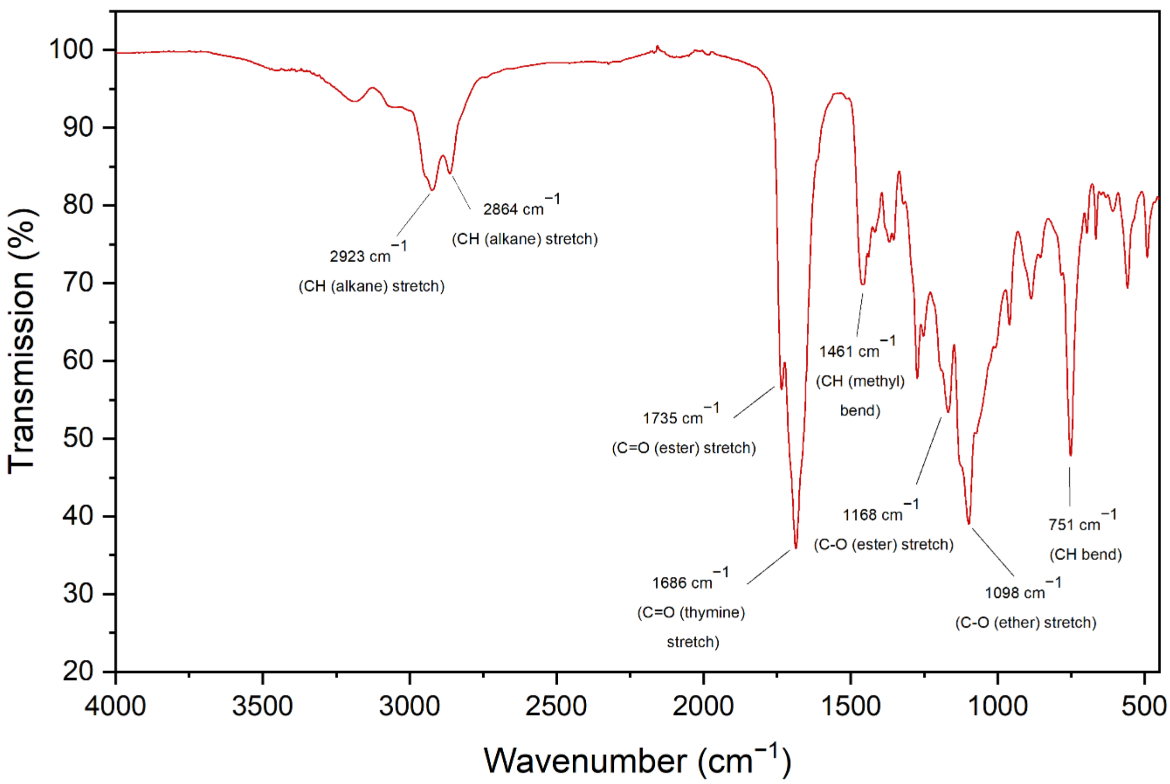

Figure S7.10: FTIR spectrum of poly(**5**-EDT).

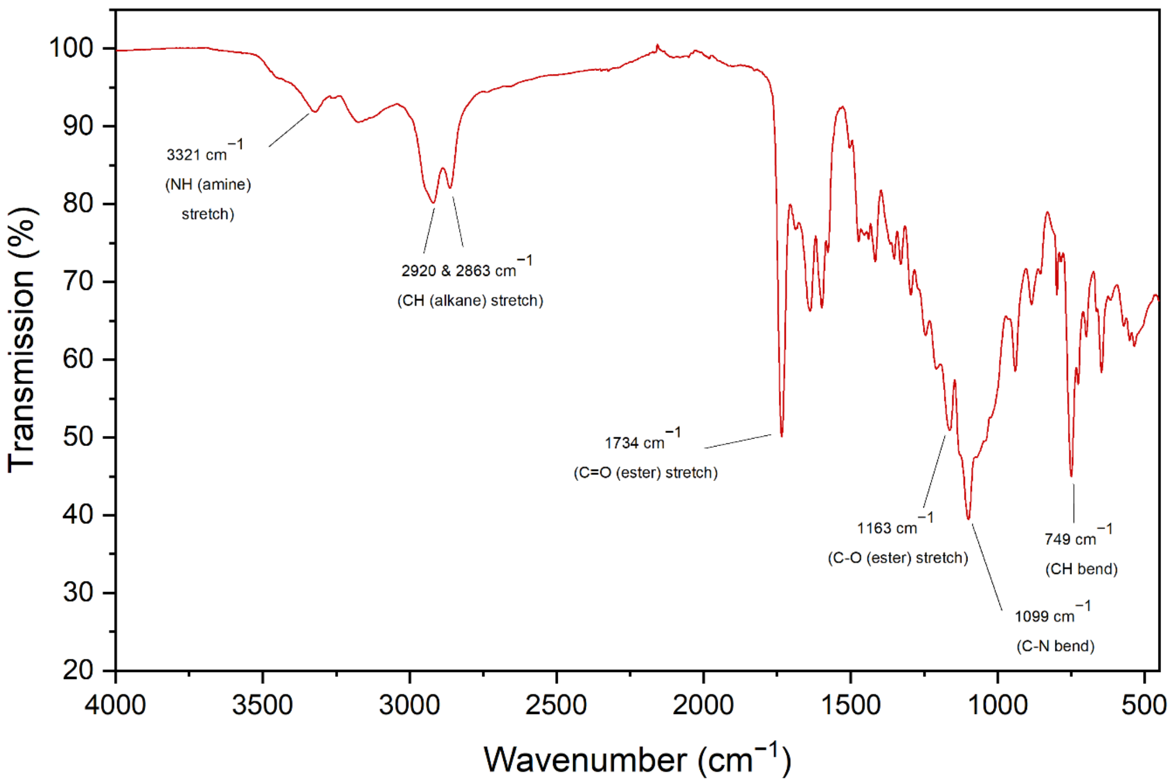

Figure S7.11: FTIR spectrum of poly(**6**-EDT).

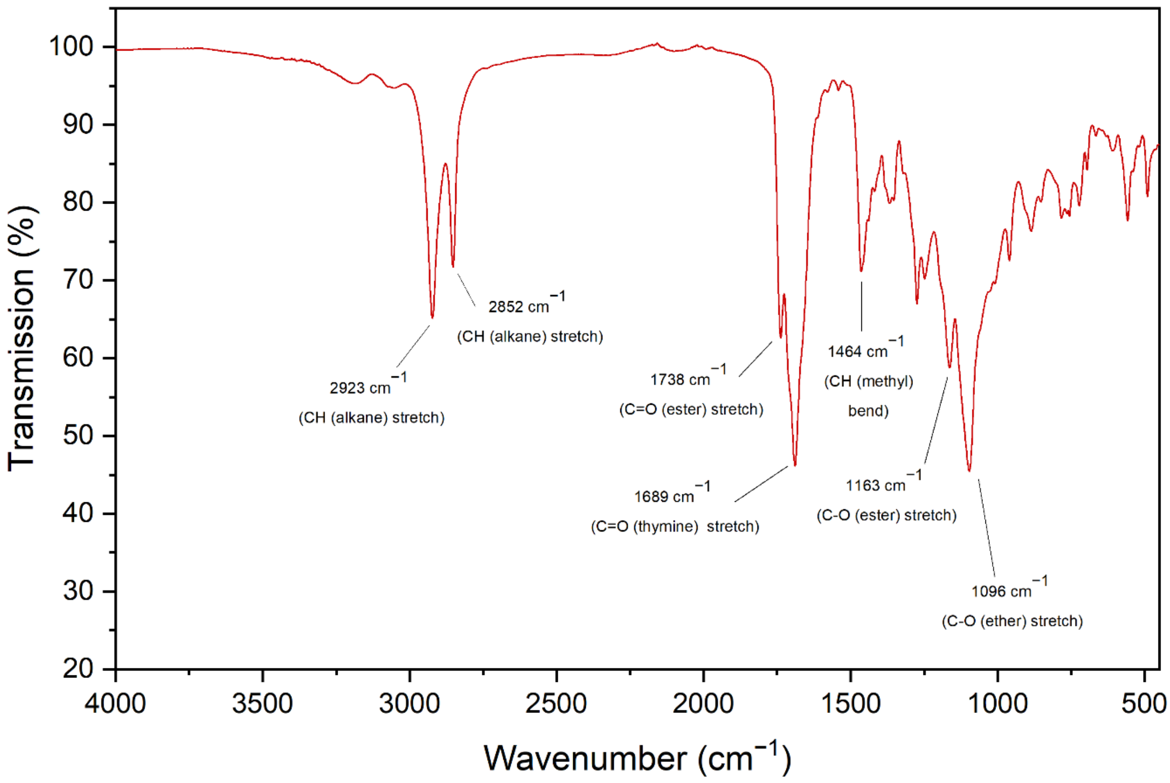

Figure S7.12: FTIR spectrum of poly(**7**-EDT).

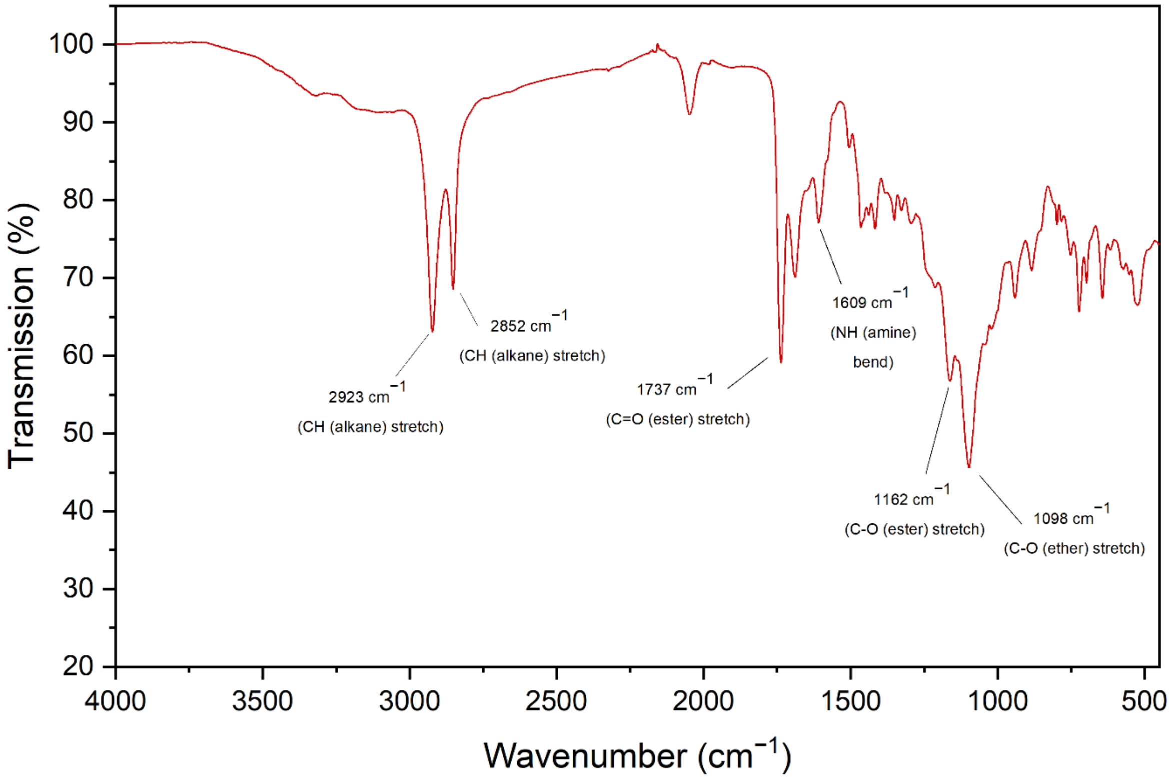

Figure S7.13: FTIR spectrum of poly(**8**-EDT).

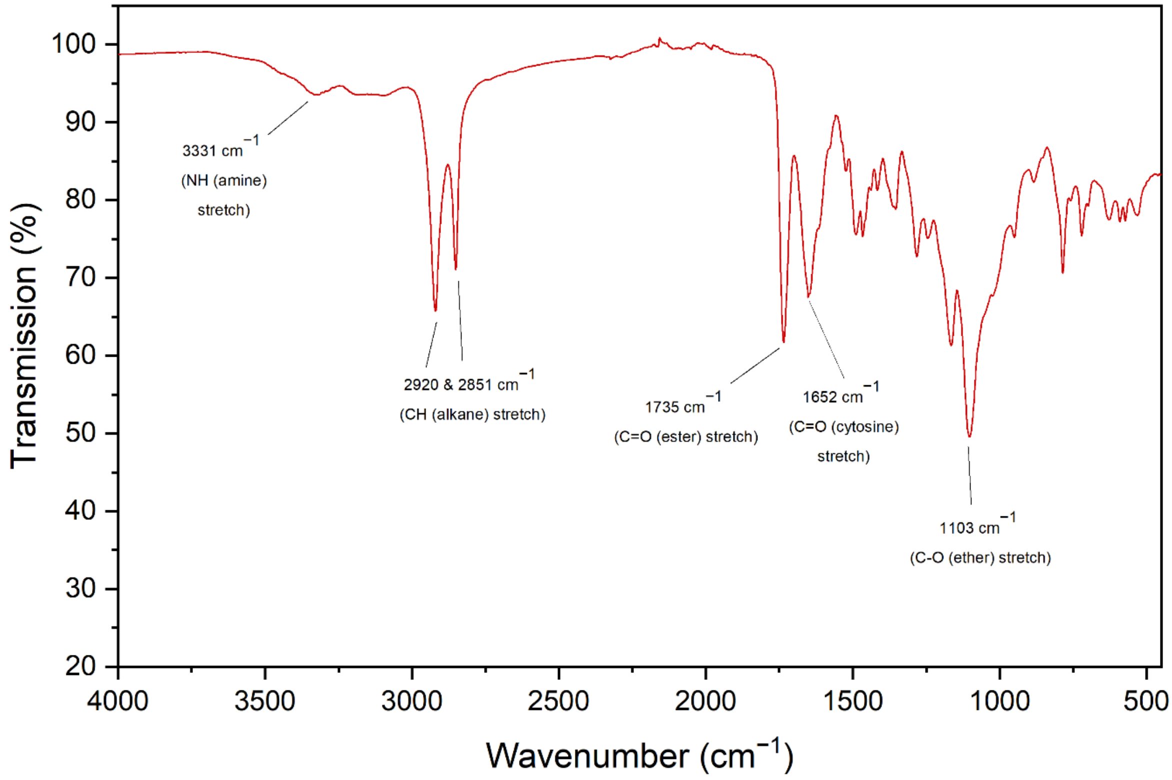

Figure S7.14: FTIR spectrum of poly(**9**-EDT).

SPEs

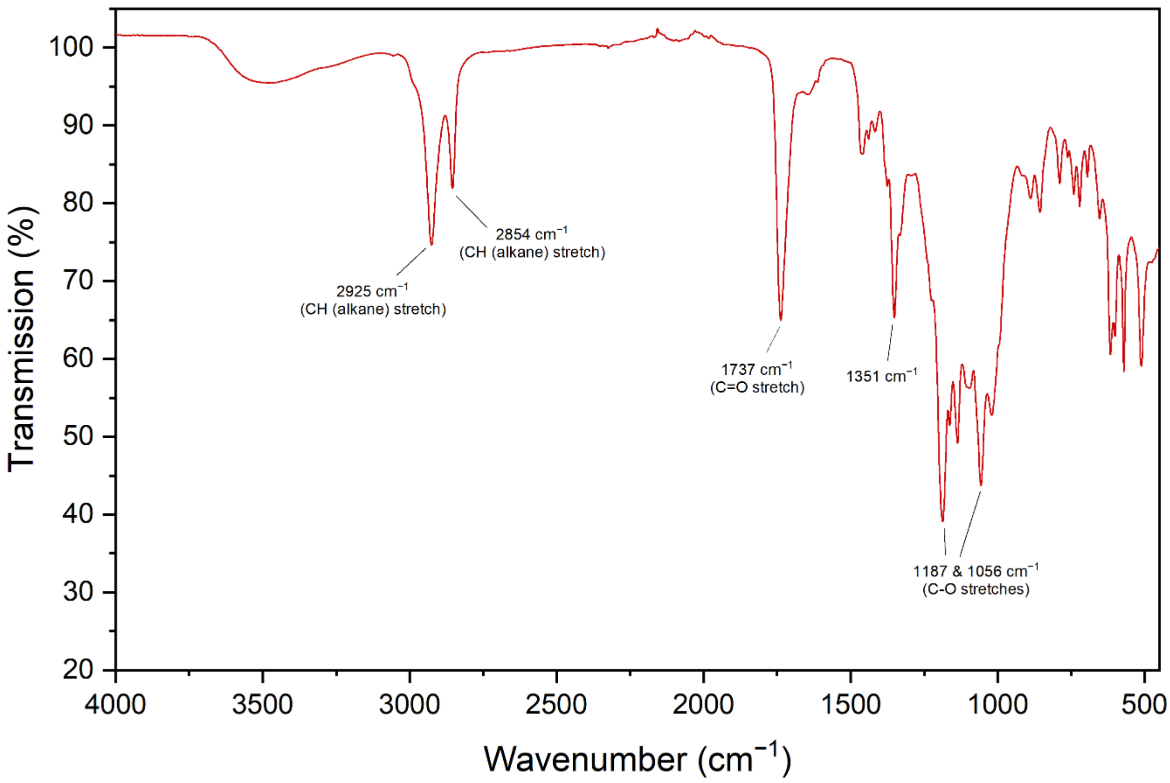

Figure S7.15: Representative FTIR spectrum of a covalently cross-linked SPE (**SPE-3d**).

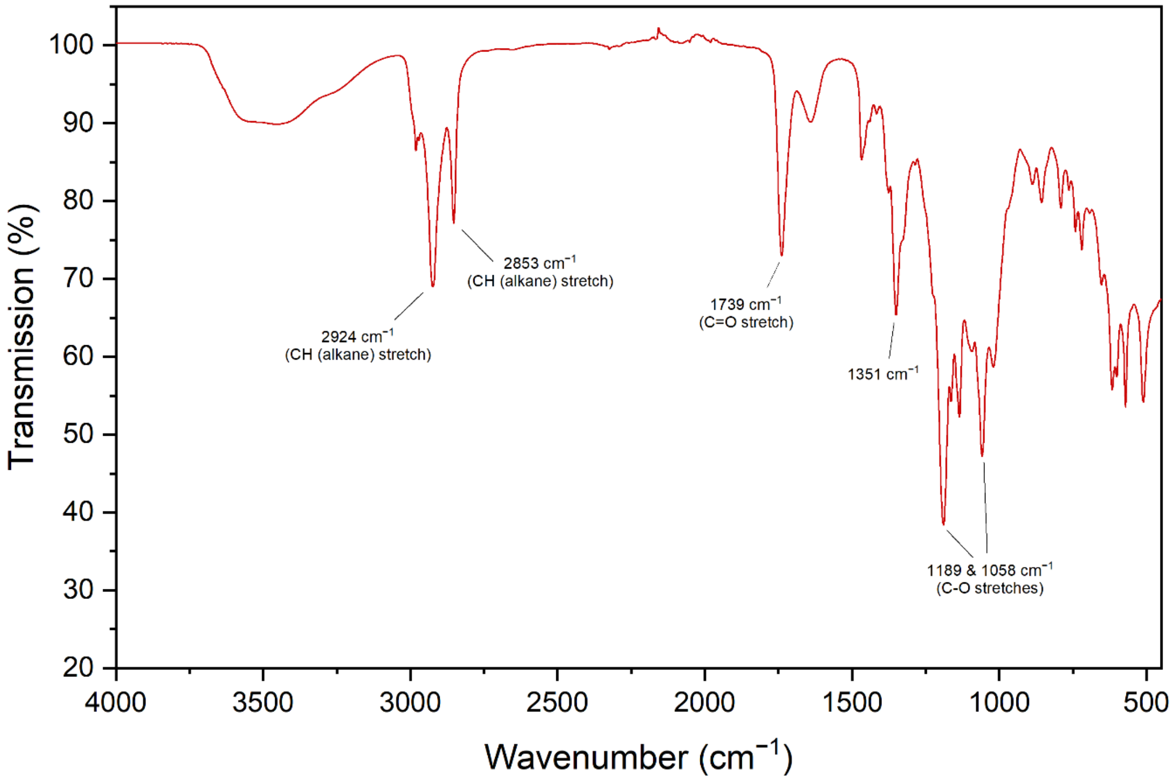

Figure S7.16: Representative FTIR spectrum of poly((**3**-EDT)-*b*-(**3**-ODT)) + 100 mol% LiTFSI.

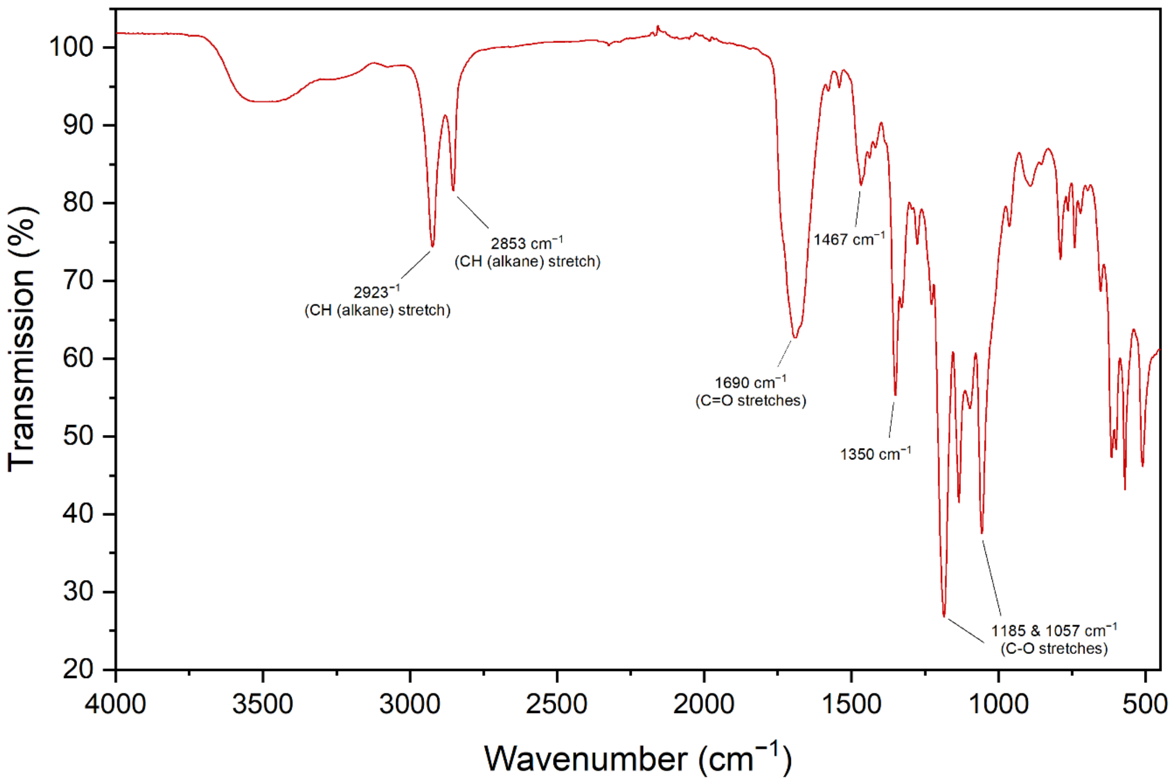

Figure S7.17: Representative FTIR spectrum of a nucleoside-based SPE (**SPE-7/8d**).

8. Mass Spectrometry

Compound specific information

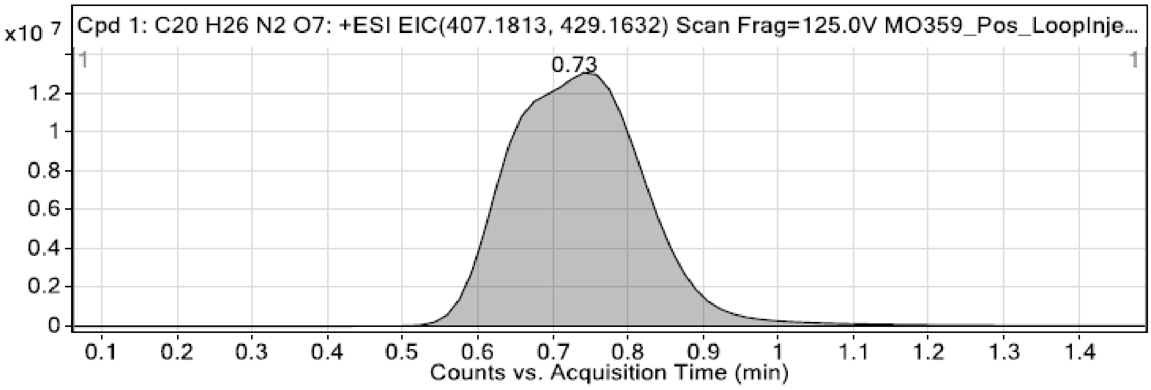

Figure: Extracted ion chromatogram (EIC) of compound.

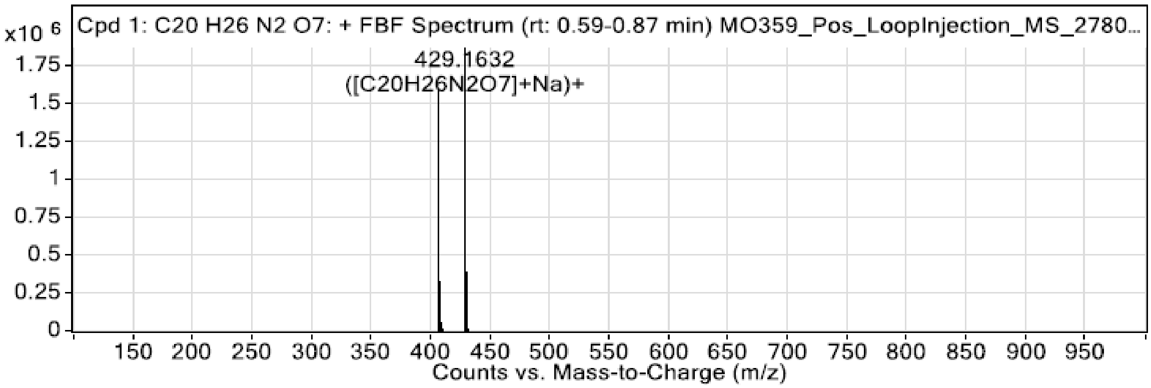

Figure: Full range view of Compound spectra and potential adducts.

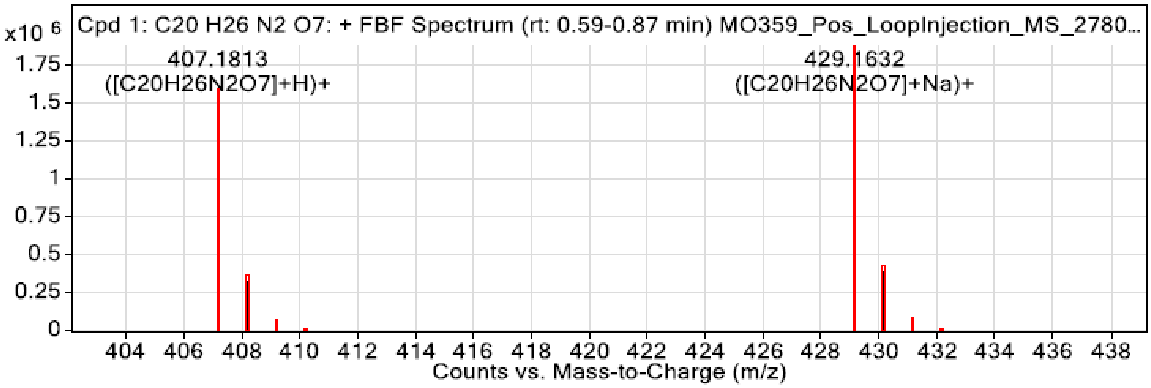

Figure S8.1: Extracted ion chromatogram and the predicted (red) *vs.* found (black) isotope pattern for $[(C_{20}H_{26}N_2O_7)+H]^+$ in the mass spectrometry analysis of monomer **5**.

Compound specific information

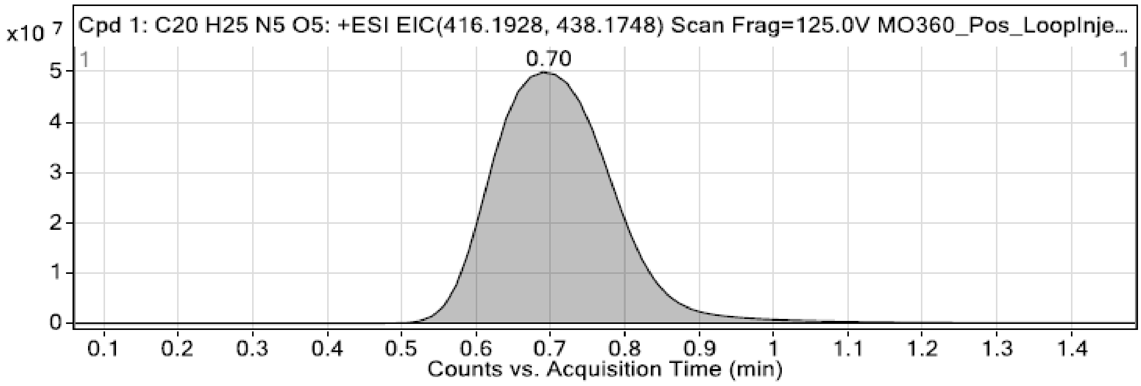

Figure: Extracted ion chromatogram (EIC) of compound.

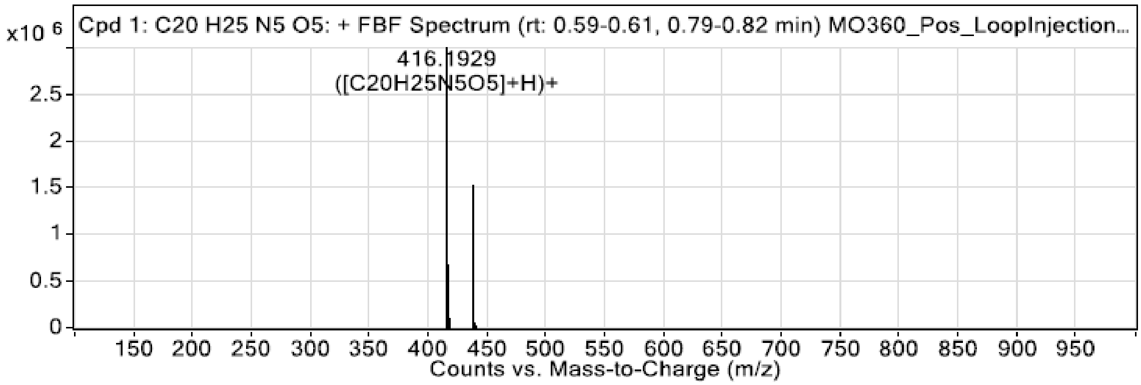

Figure: Full range view of Compound spectra and potential adducts.

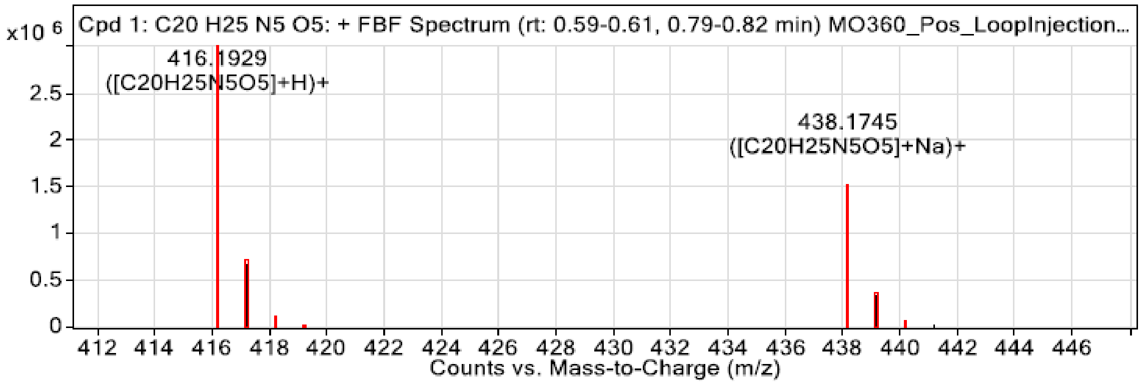

Figure S8.2: Extracted ion chromatogram and the predicted (red) *vs.* found (black) isotope pattern for $[(C_{20}H_{25}N_5O_5)+H]^+$ in the mass spectrometry analysis of monomer **6**.

Compound specific information

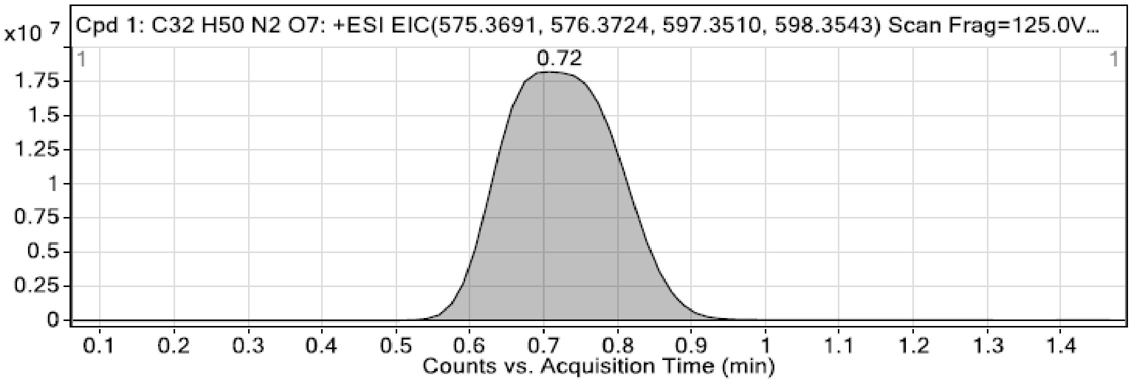

Figure: Extracted ion chromatogram (EIC) of compound.

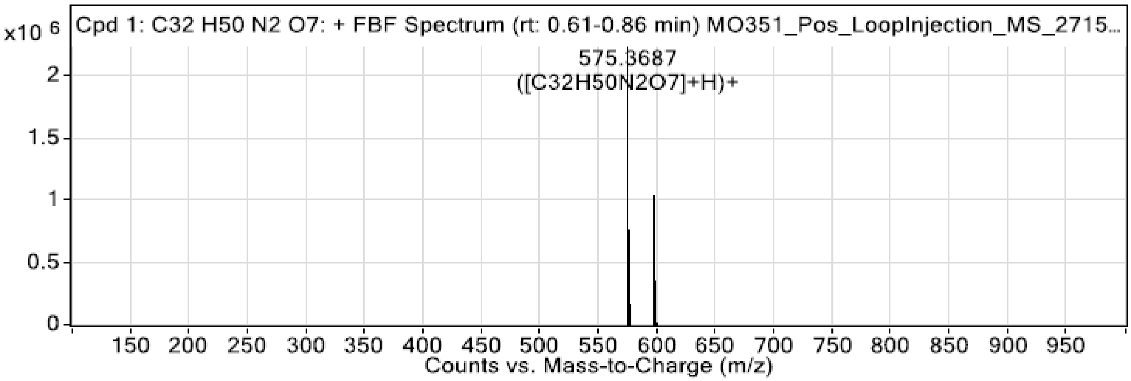

Figure: Full range view of Compound spectra and potential adducts.

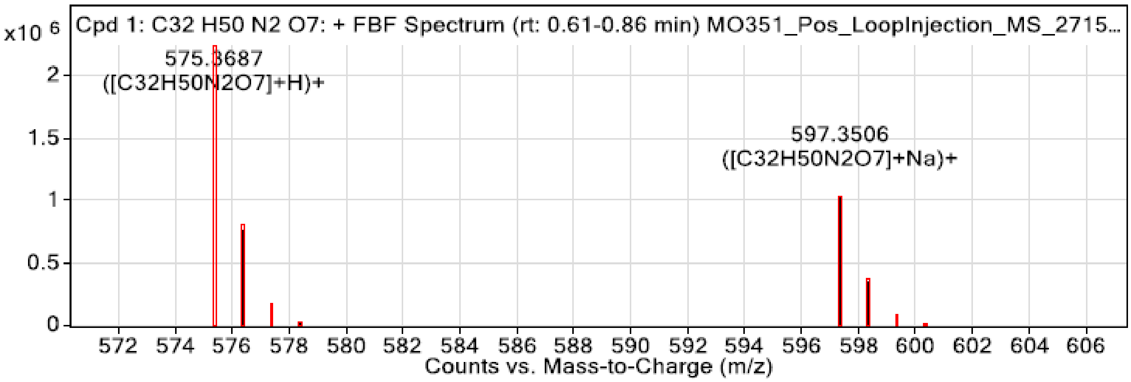

Figure S8.3: Extracted ion chromatogram and the predicted (red) *vs.* found (black) isotope pattern for $[(C_{32}H_{50}N_2O_7)+H]^+$ in the mass spectrometry analysis of monomer **7**.

Compound specific information

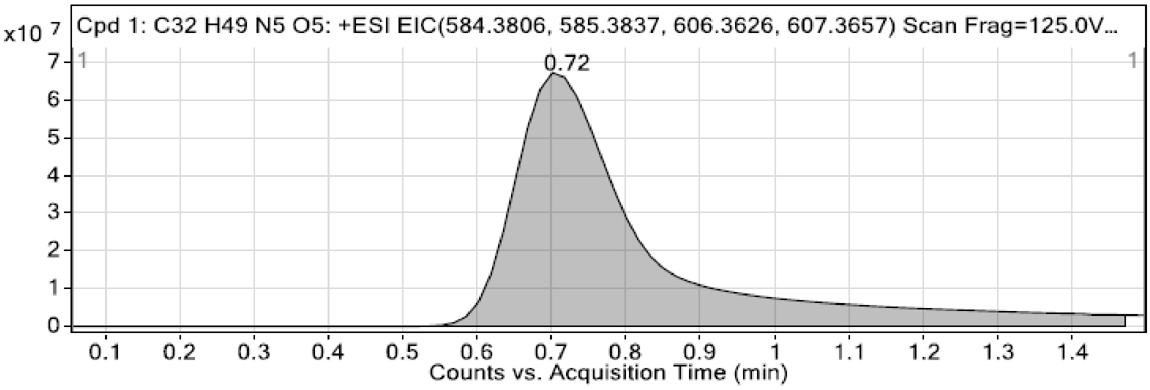

Figure: Extracted ion chromatogram (EIC) of compound.

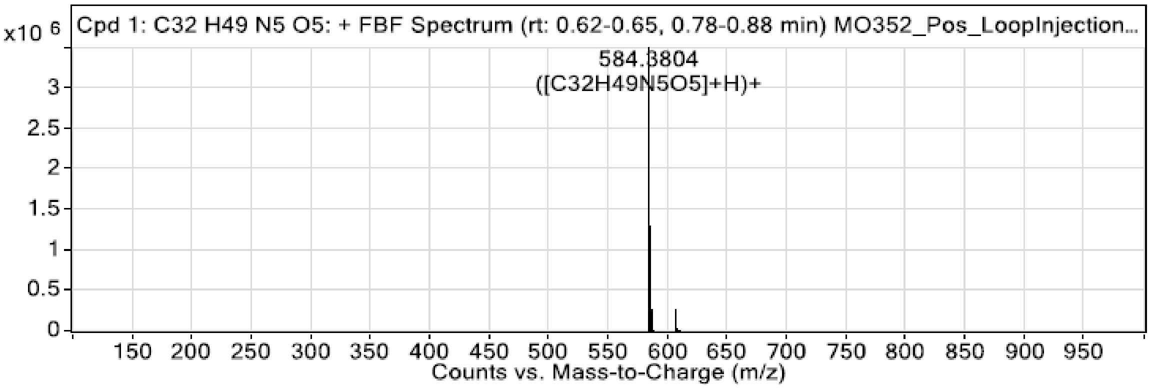

Figure: Full range view of Compound spectra and potential adducts.

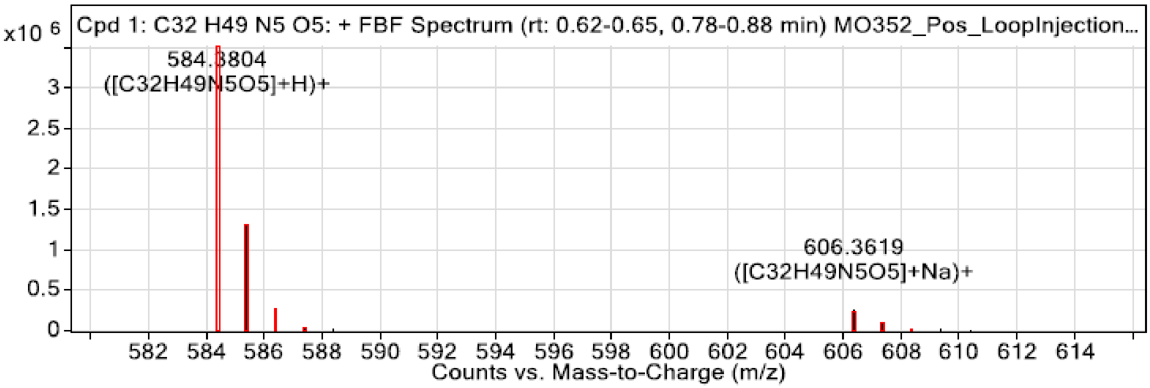

Figure S8.4: Extracted ion chromatogram and the predicted (red) *vs.* found (black) isotope pattern for $[(C_{32}H_{49}N_5O_5)+H]^+$ in the mass spectrometry analysis of monomer **8.**

Compound specific information

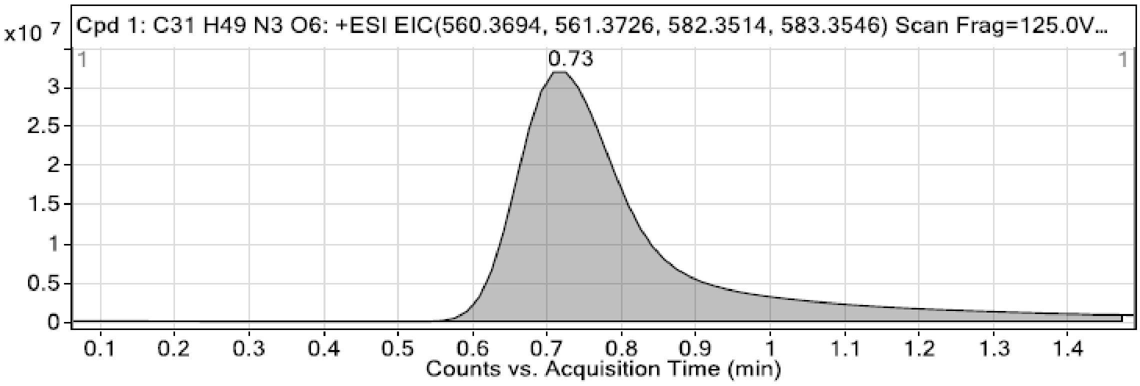

Figure: Extracted ion chromatogram (EIC) of compound.

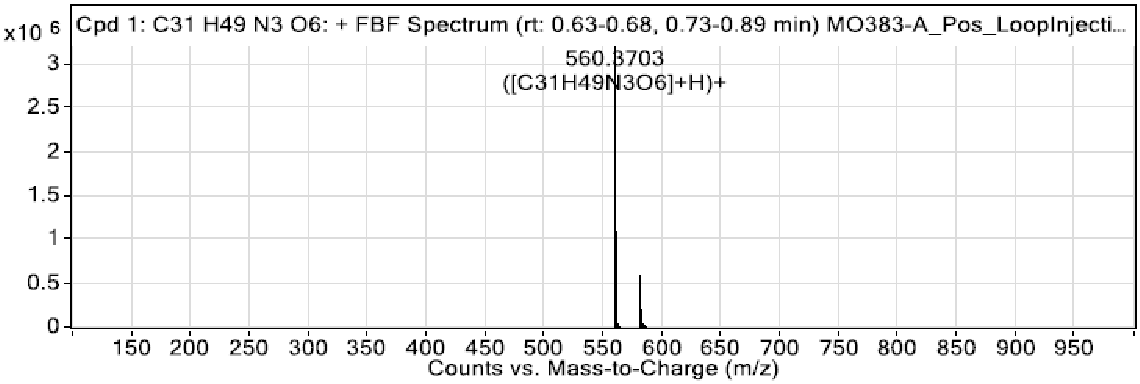

Figure: Full range view of Compound spectra and potential adducts.

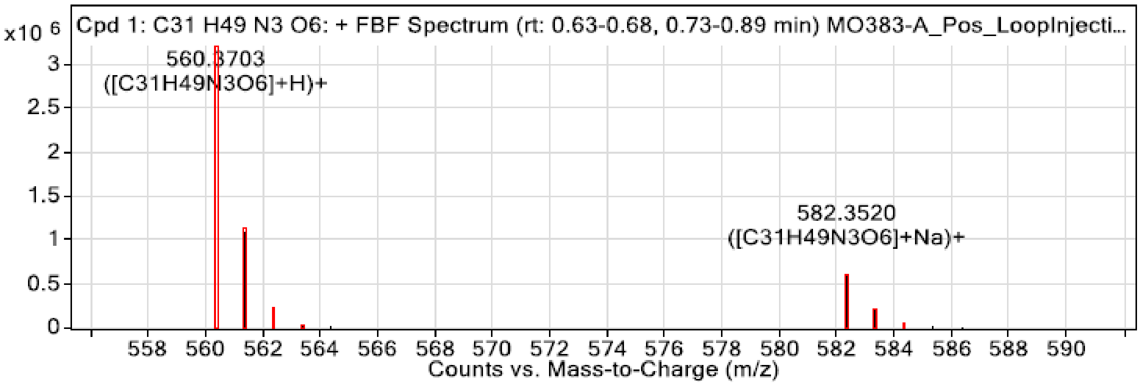

Figure S8.5: Extracted ion chromatogram and the predicted (red) *vs.* found (black) isotope pattern for $[(C_{31}H_{49}N_3O_6)+H]^+$ in the mass spectrometry analysis of monomer **9**.

9. Differential scanning calorimetry (DSC)

All DSC analysis was performed under a flow of N_2 gas.

Co-polymers

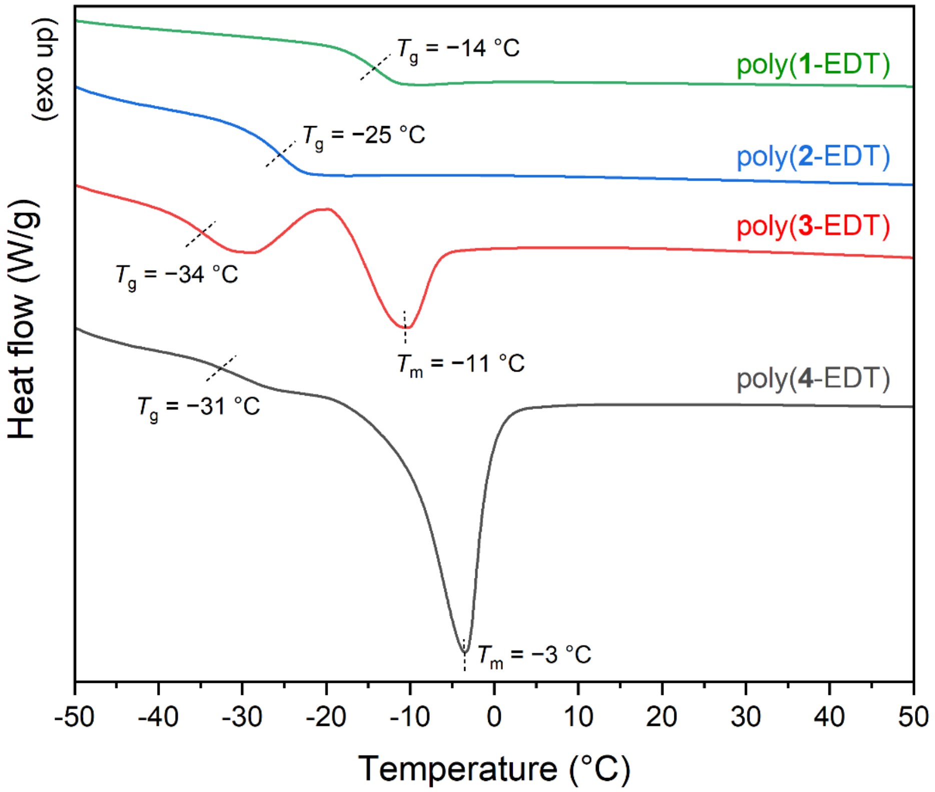

Figure S9.1: Overlaid DSC thermograms showing the second heating cycle of xylose-based thiol-ene co-polymer poly(**1**-EDT) between −50 °C and 50 °C.

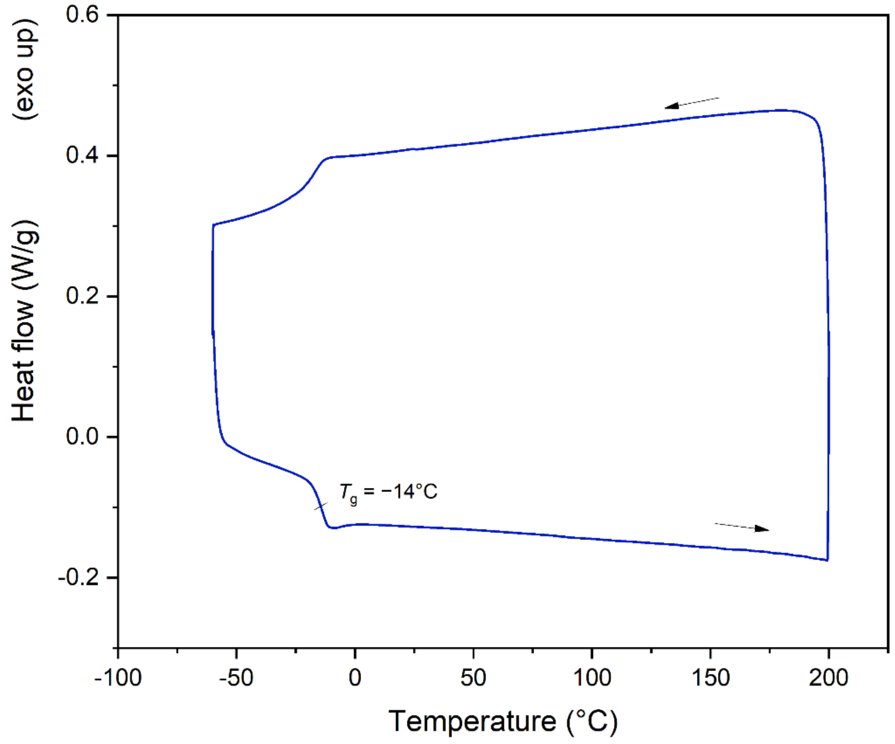

Figure S9.2: DSC thermogram showing the second cooling and heating cycle of poly(**1**-EDT) between −60 °C and 200 °C. $\boldsymbol{T_g}$ = −14 °C.

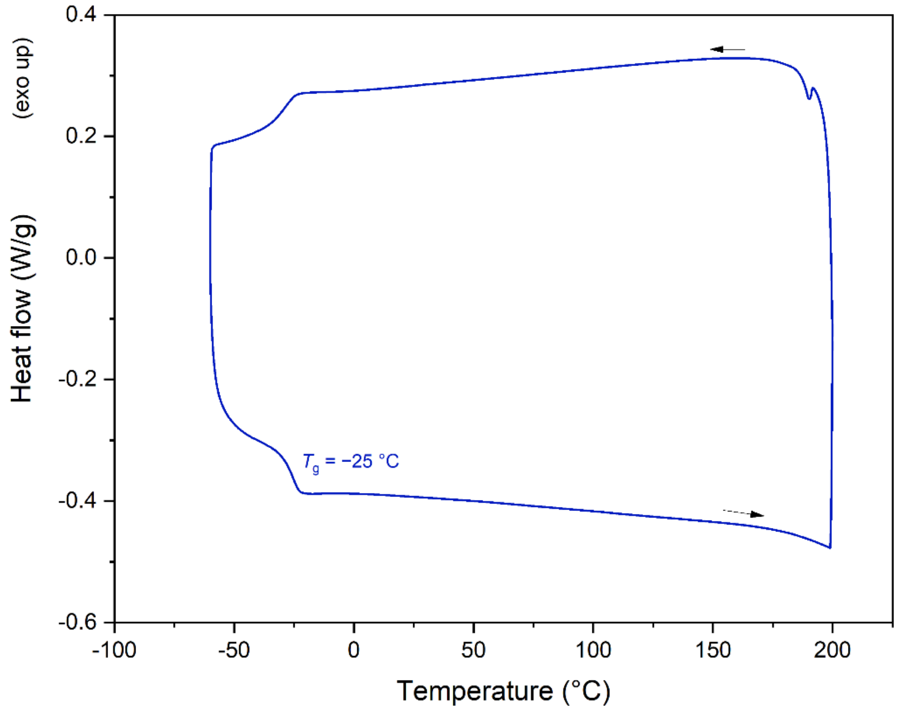

Figure S9.3: DSC thermogram showing the second cooling and heating cycle of poly(**2**-EDT) between −60 °C and 200 °C. $\boldsymbol{T_g}$ = −25 °C.

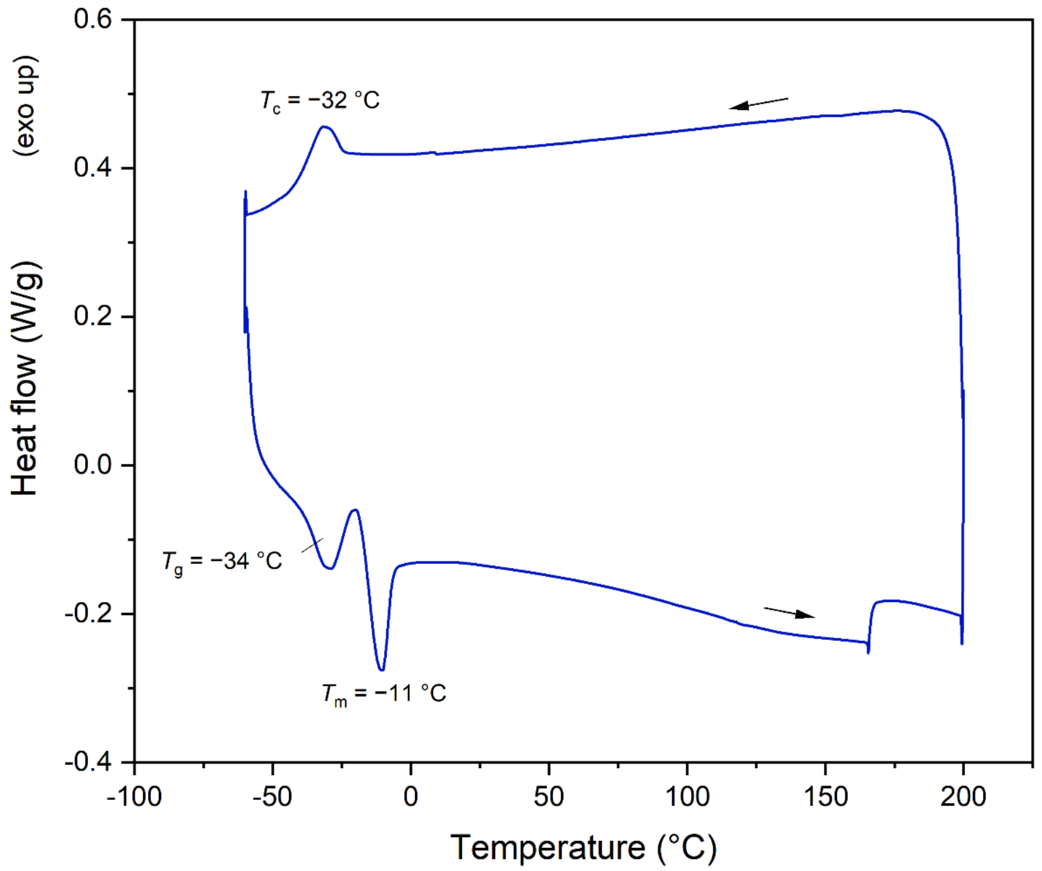

Figure S9.4: DSC thermogram showing the second cooling and heating cycle of poly(**3**-EDT) between −60 °C and 200 °C. $\boldsymbol{T_g}$ = −34 °C, $\boldsymbol{T_m}$ = −11 °C, $\boldsymbol{T_c}$ = −32 °C.

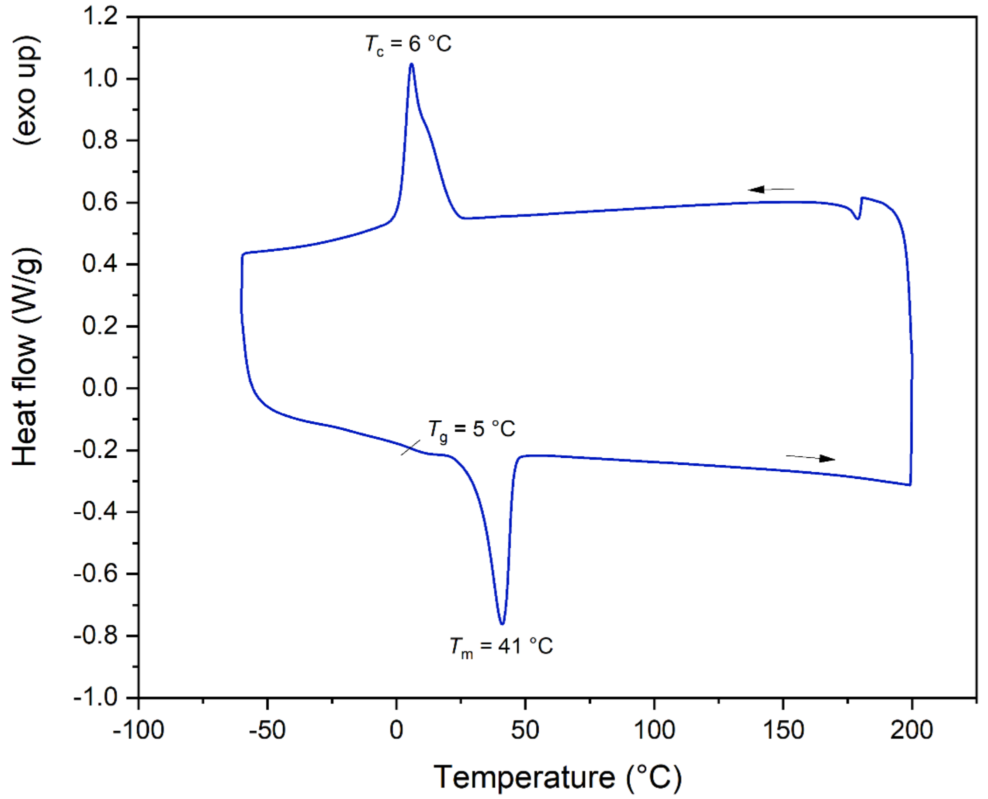

Figure S9.5: DSC thermogram showing the second cooling and heating cycle of poly(**3**-ODT) between −60 °C and 200 °C. $\boldsymbol{T_g}$ = 5 °C, $\boldsymbol{T_m}$ = 41 °C, $\boldsymbol{T_c}$ = 6 °C.

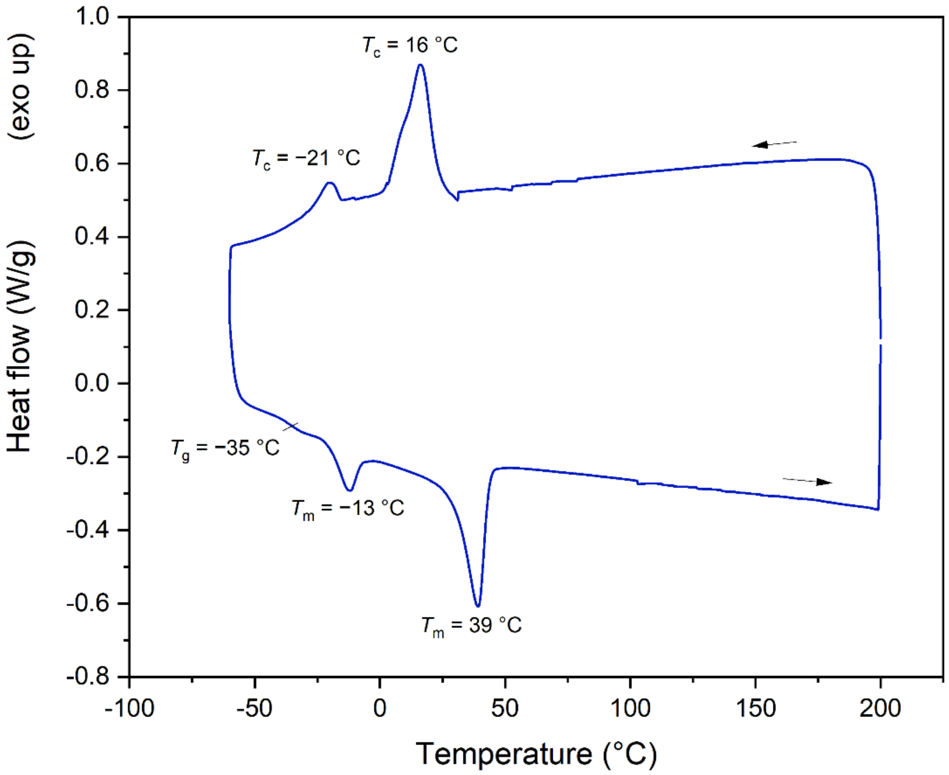

Figure S9.6: DSC thermogram showing the second cooling and heating cycle of poly((**3**-EDT)-*b*-(**3**-ODT)) (**Table 3**, entry 2) between −60 °C and 200 °C. $\boldsymbol{T_g}$ = −35 °C, $\boldsymbol{T_m}$ = −13 and 39 °C, $\boldsymbol{T_c}$ = 21 and 16 °C.

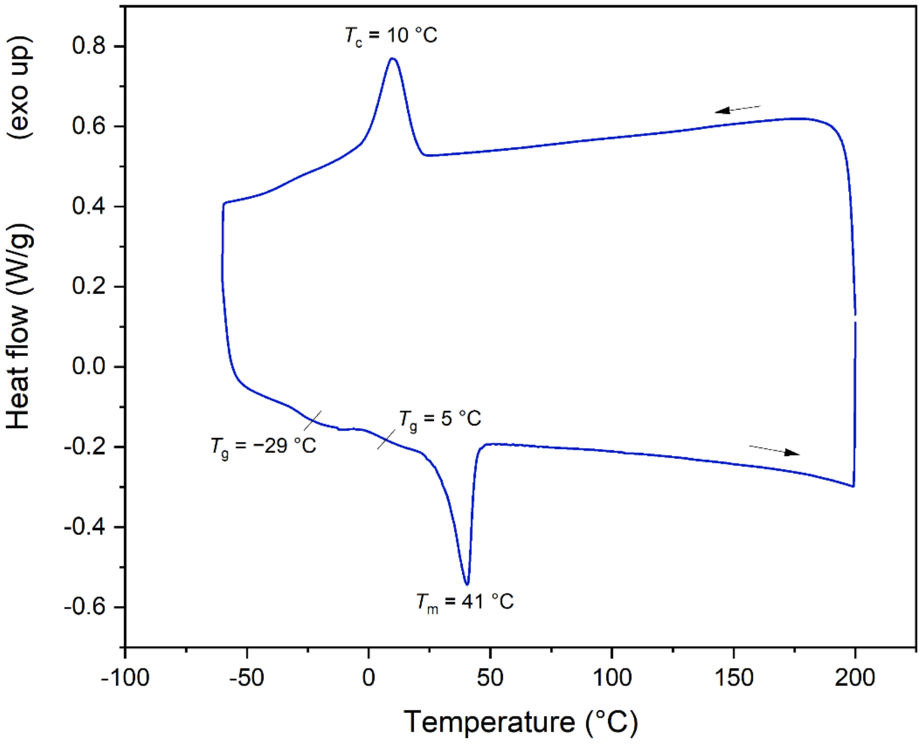

Figure S9.7: DSC thermogram showing the second cooling and heating cycle of poly((**3**-EDT)-*b*-(**3**-ODT)) (**Table 3**, entry 3) between −60 °C and 200 °C. $\boldsymbol{T_g}$ = −29 and 5 °C, $\boldsymbol{T_m}$ = 41 °C, $\boldsymbol{T_c}$ = 10 °C.

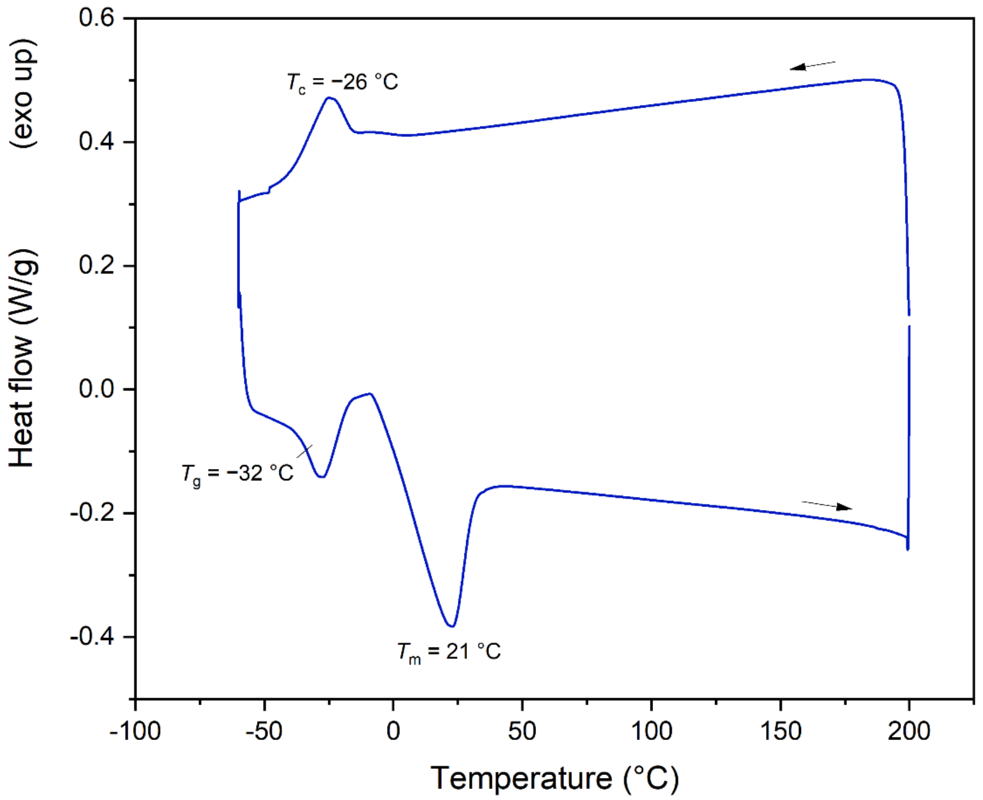

Figure S9.8: DSC thermogram showing the second cooling and heating cycle of poly(**3**-EDT/ODT) (**Table 3**, entry 4) between −60 °C and 200 °C. $\boldsymbol{T_g}$ = −32 °C, $\boldsymbol{T_m}$ = 21 °C, $\boldsymbol{T_c}$ = −26 °C.

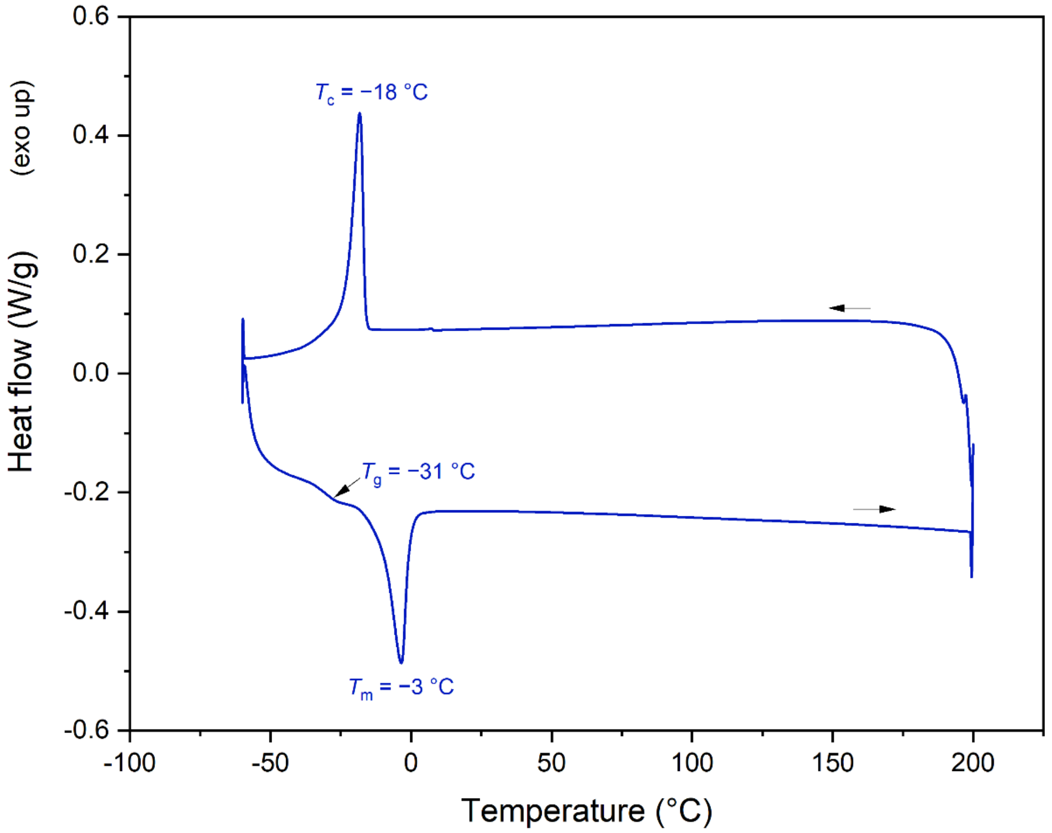

Figure S9.9: DSC thermogram showing the second cooling and heating cycle of poly(**4**-EDT) between −60 °C and 200 °C. $\boldsymbol{T_g}$ = −31 °C, $\boldsymbol{T_m}$ = −3 °C, $\boldsymbol{T_c}$ = −18 °C.

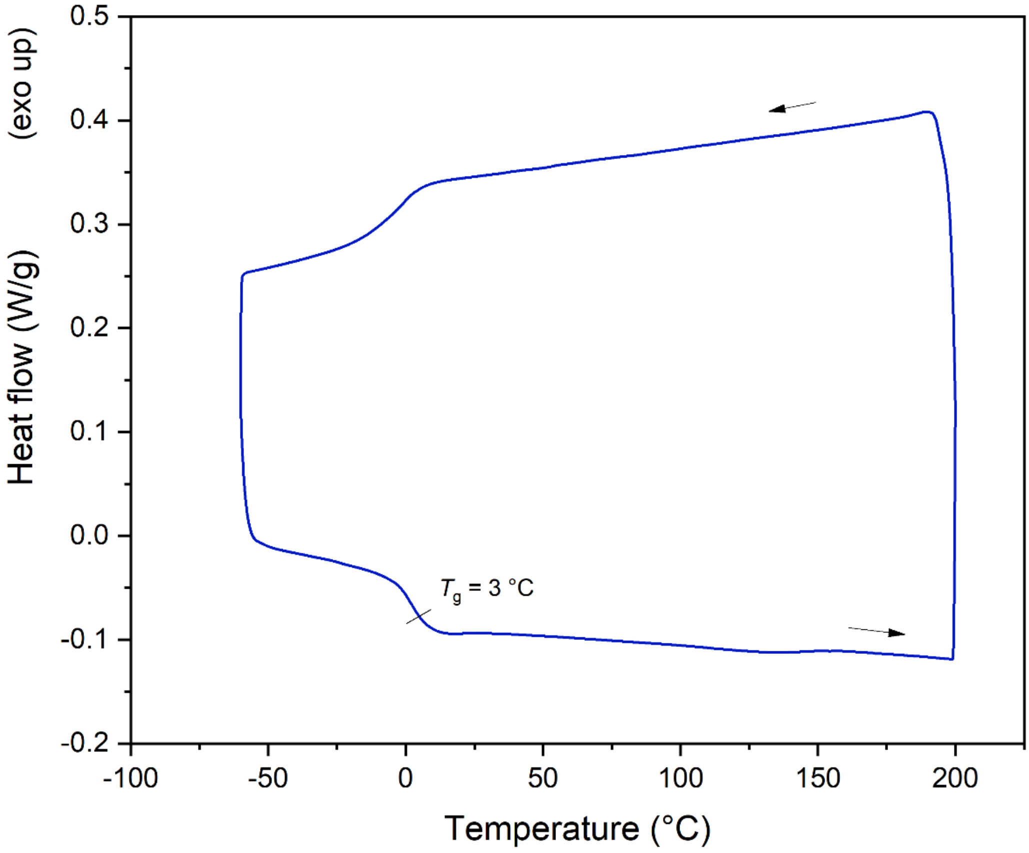

Figure S9.10: DSC thermogram showing the second cooling and heating cycle of poly(**5**-EDT) between −60 °C and 200 °C. $\boldsymbol{T_g}$ = 3 °C.

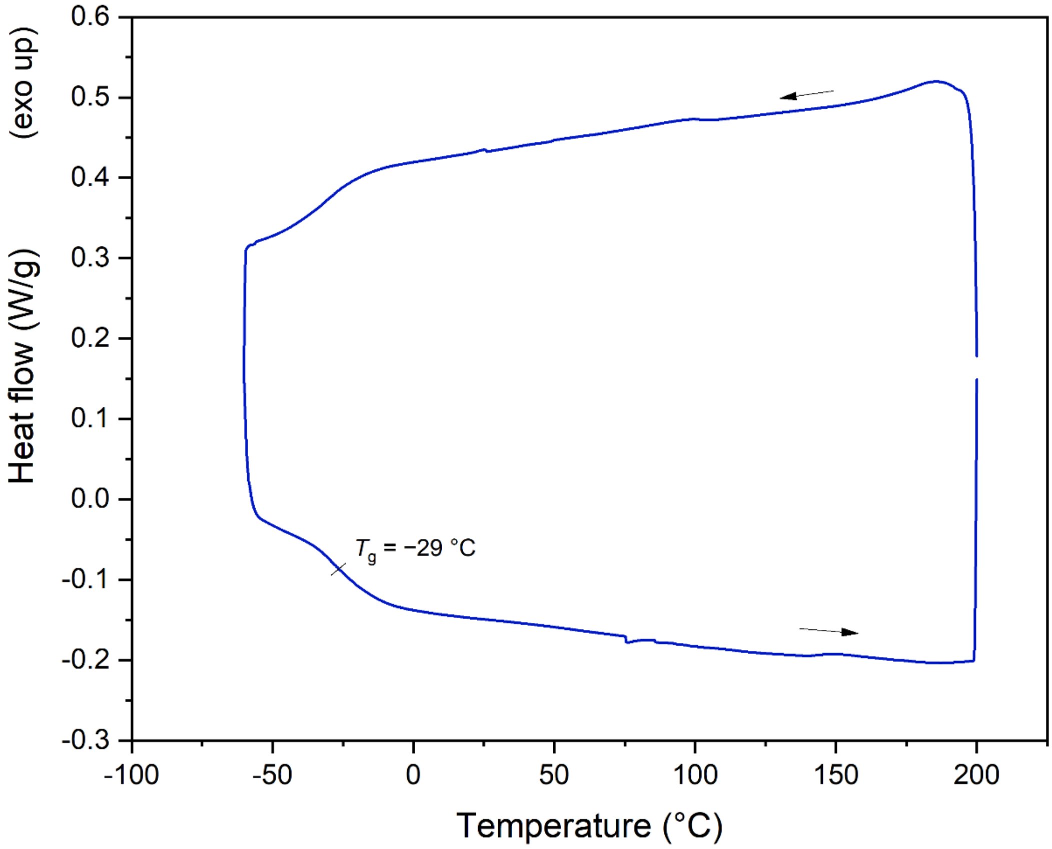

Figure S9.11: DSC thermogram showing the second cooling and heating cycle of poly(**6**-EDT) between −60 °C and 200 °C. $\boldsymbol{T_g}$ = −29 °C.

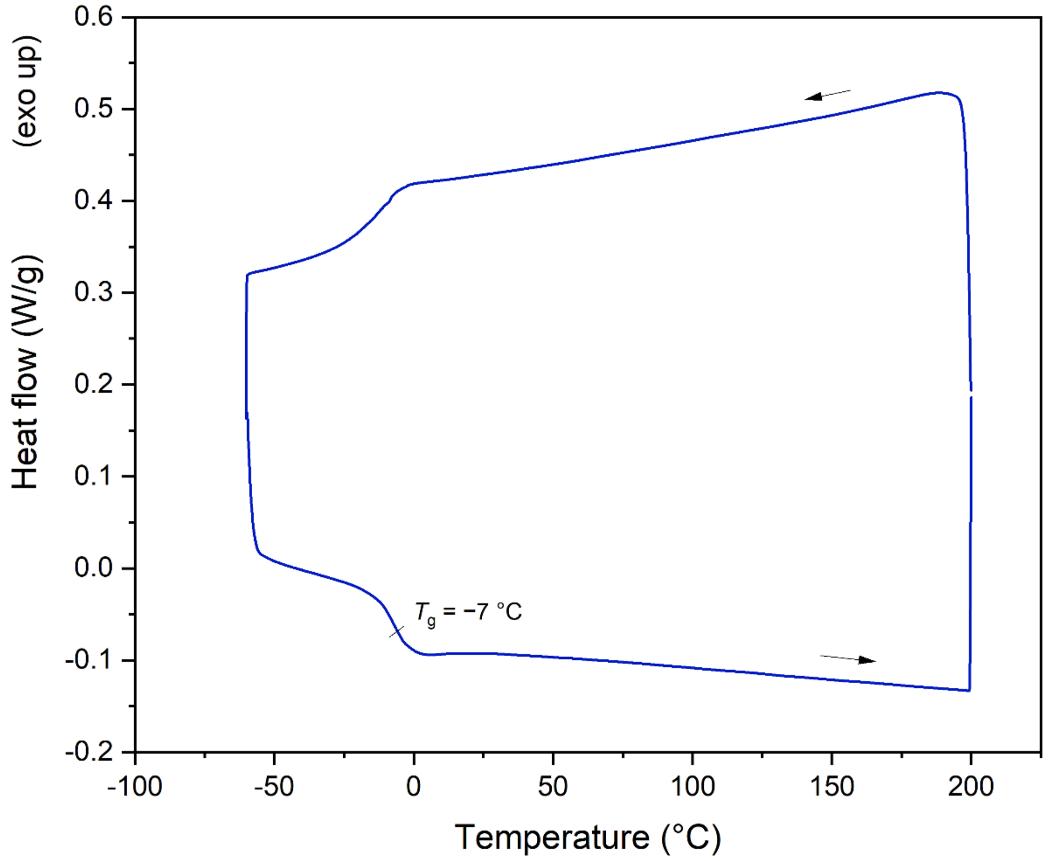

Figure S9.12: DSC thermogram showing the second cooling and heating cycle of poly(**7**-EDT) between −60 °C and 200 °C. $\boldsymbol{T_g}$ = −7 °C.

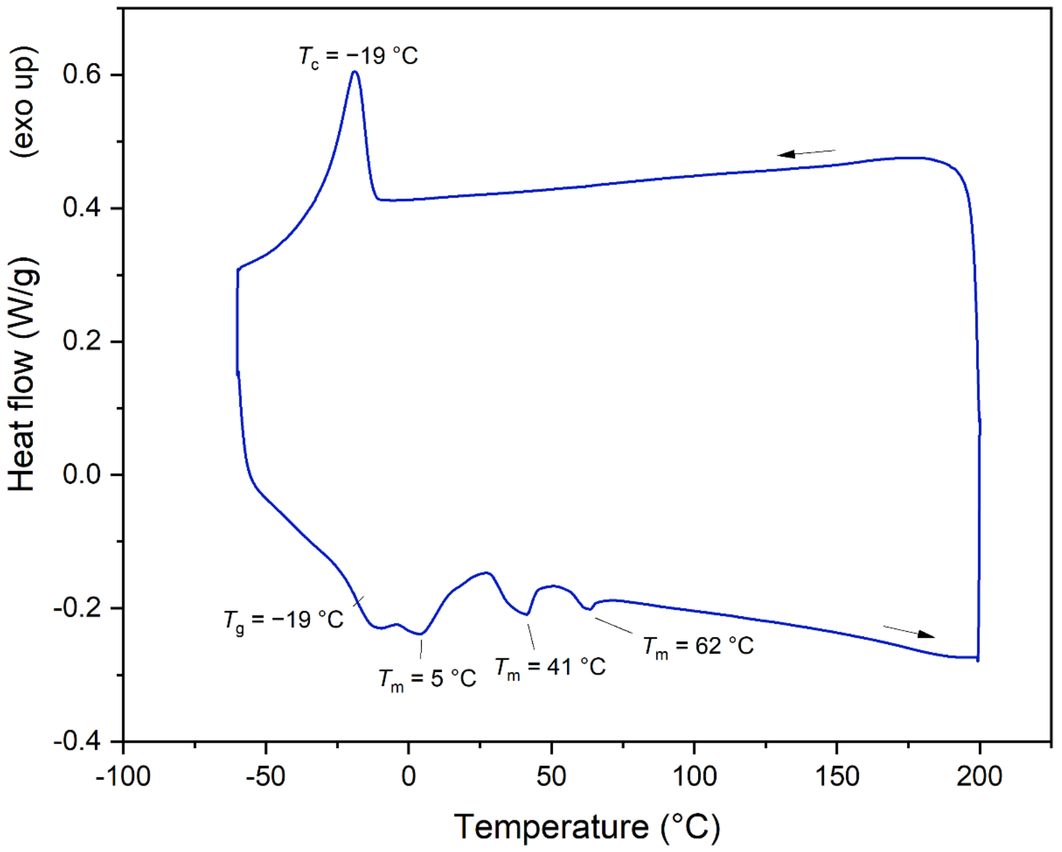

Figure S9.13: DSC thermogram showing the second cooling and heating cycle of poly(**8**-EDT) between −60 °C and 200 °C. $\boldsymbol{T_g}$ = −19 °C, $\boldsymbol{T_m}$ = 5, 41 and 62 °C, $\boldsymbol{T_c}$ **=** −19 °C.

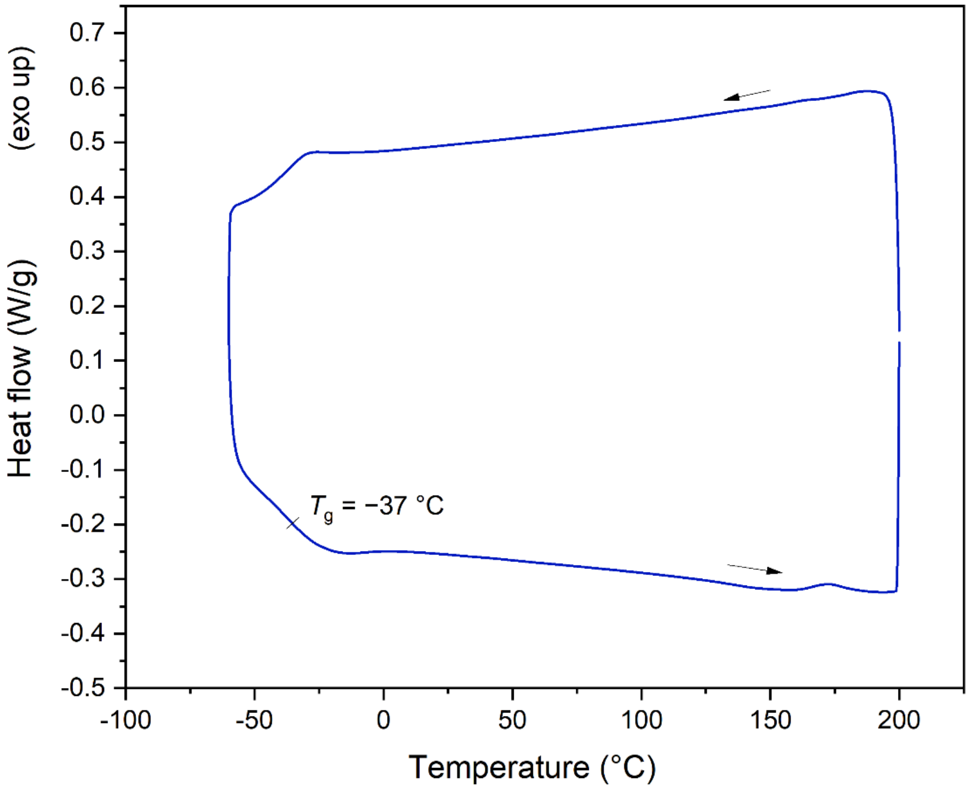

Figure S9.14: DSC thermogram showing the second cooling and heating cycle of poly(**9**-EDT) between −60 °C and 200 °C. $\boldsymbol{T_g}$ = −37 °C.

Representative SPEs

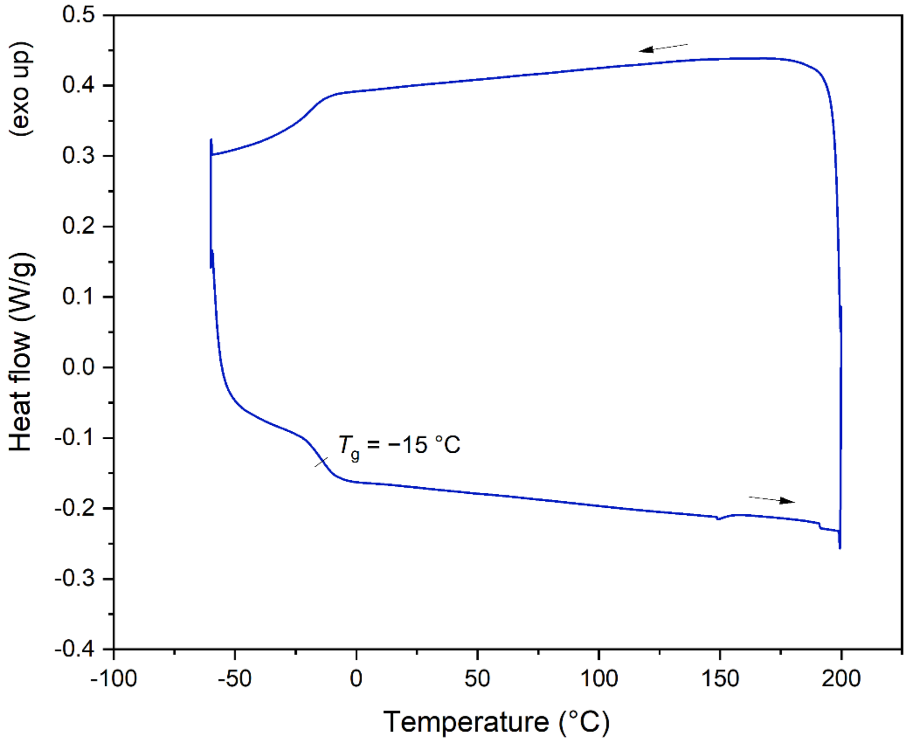

Figure S9.15: DSC thermogram showing the second cooling and heating cycle of a representative covalently cross-linked SPE based on monomer **3**, EDT and TMP (9:1) (**SPE-3d**, **Table 2**, entry 7) between −60 °C and 200 °C. $\boldsymbol{T_g}$ = −15 °C.

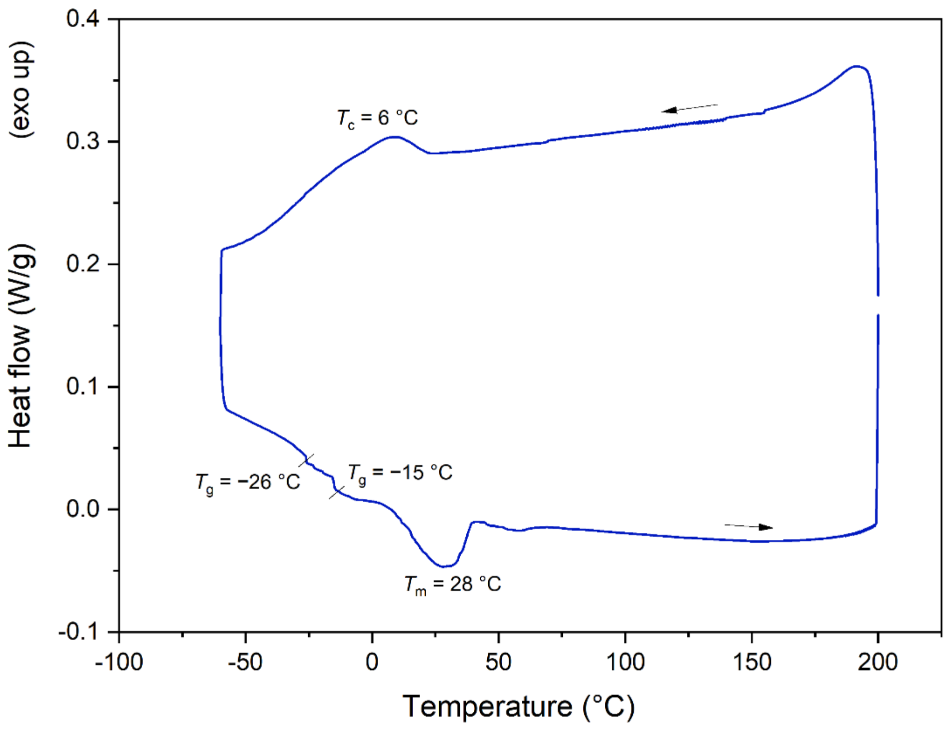

Figure S9.16: DSC thermogram showing the second cooling and heating cycle of a semi-crystalline SPE based on monomer **3**, EDT and ODT between −60 °C and 200 °C. $\boldsymbol{T_g}$ = −26 and −15 °C, $\boldsymbol{T_m}$ = 28 °C, $\boldsymbol{T_c}$ = 6 °C.

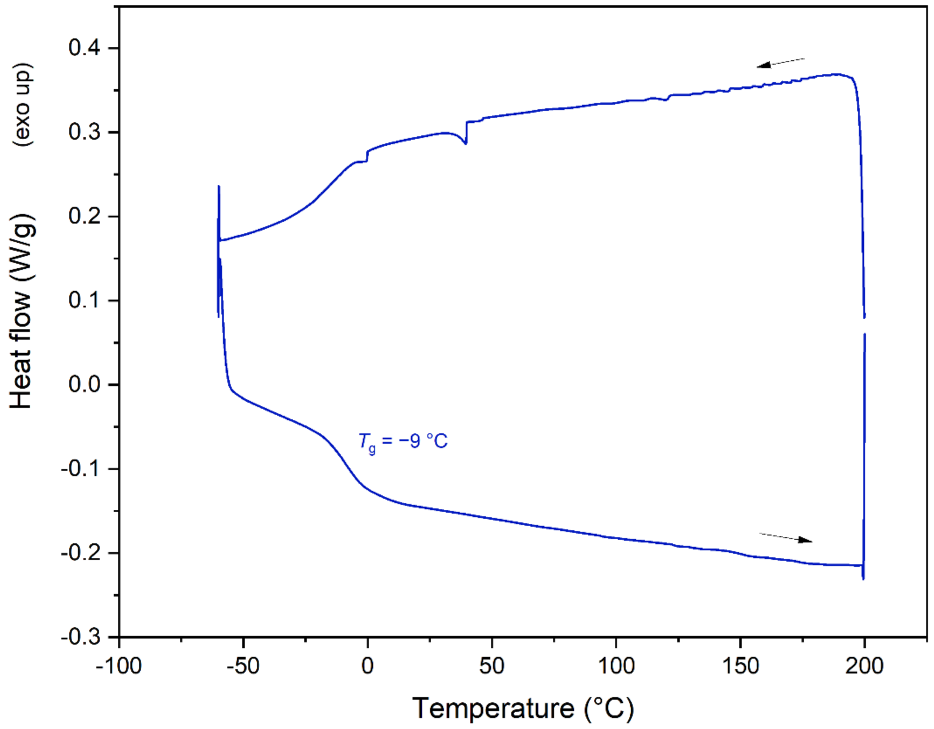

Figure S9.17: DSC thermogram showing the second cooling and heating cycle of a representative nucleoside-based SPE based on monomers **7** & **8** (9:1) and EDT (**SPE-7/8d**, **Table 5**, entry 8) between −60 °C and 200 °C. $\boldsymbol{T_g}$ = −9 °C.

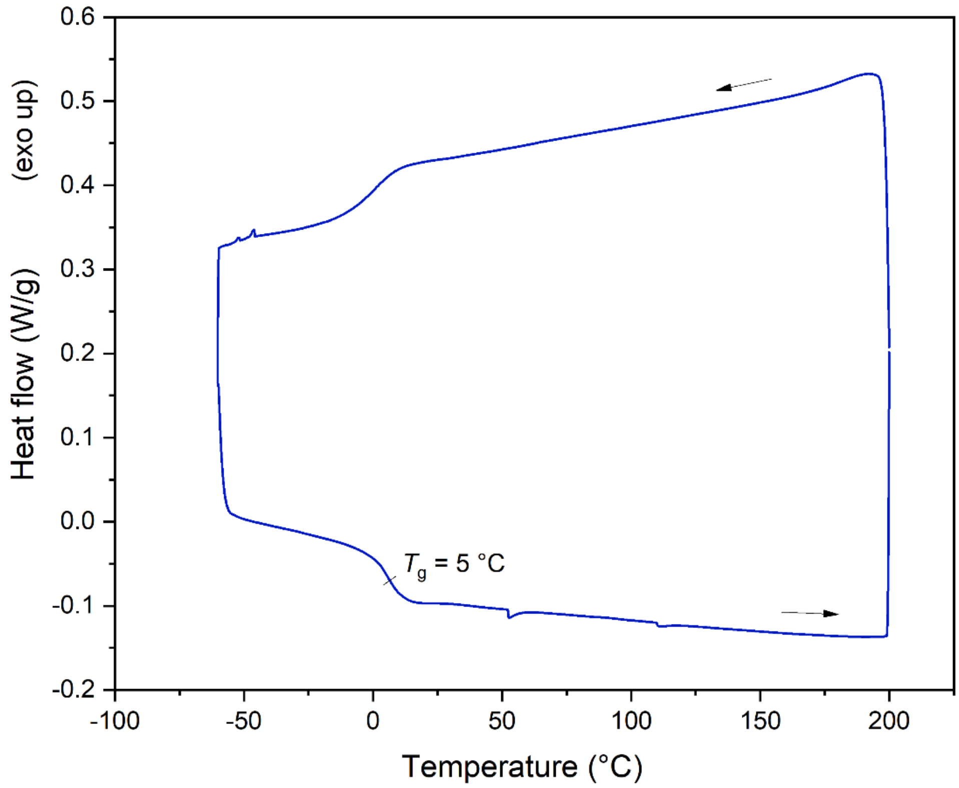

Figure S9.18: DSC thermogram showing the second cooling and heating cycle of a representative nucleoside-based SPE based on monomers **7** & **8** (9:1) with EDT & TMP (9:1) (**SPE-7/8f**, **Table 5**, entry 10) between −60 °C and 200 °C. $\boldsymbol{T_g}$ = 5 °C.

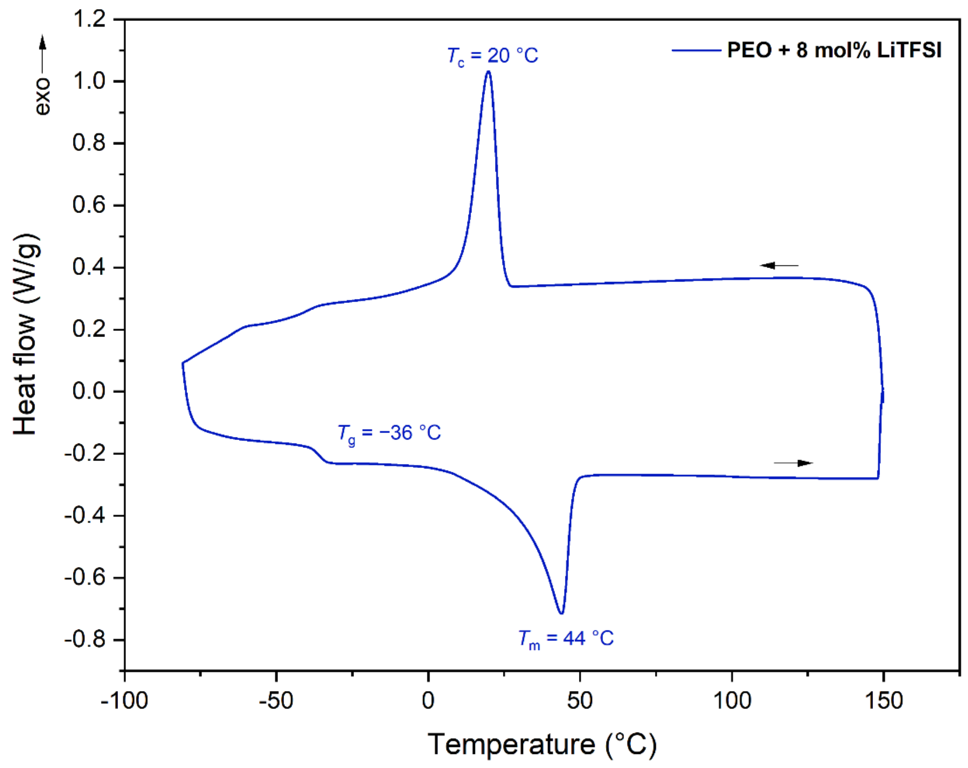

Figure S9.19: DSC thermogram showing the second cooling and heating cycle of a representative SPE prepared with commercial PEO (100 kg mol^{-1}) between −80 °C and 150 °C. $\boldsymbol{T_g}$ = −36 °C, $\boldsymbol{T_m}$ = 44 °C, $\boldsymbol{T_c}$ = 20 °C.

10. Thermogravimetric analysis (TGA)

For all charts, $\boldsymbol{T}_{\mathbf{d,5\%}}$ refers to the onset of thermal degradation where 5% mass loss has been reached. $\boldsymbol{T}_{\mathbf{d,max}}$ refers to the temperature for the maximum of the derivative peak (plotted as the dotted line). All TGA analysis was performed in a chamber purged with inert argon gas.

Co-polymers

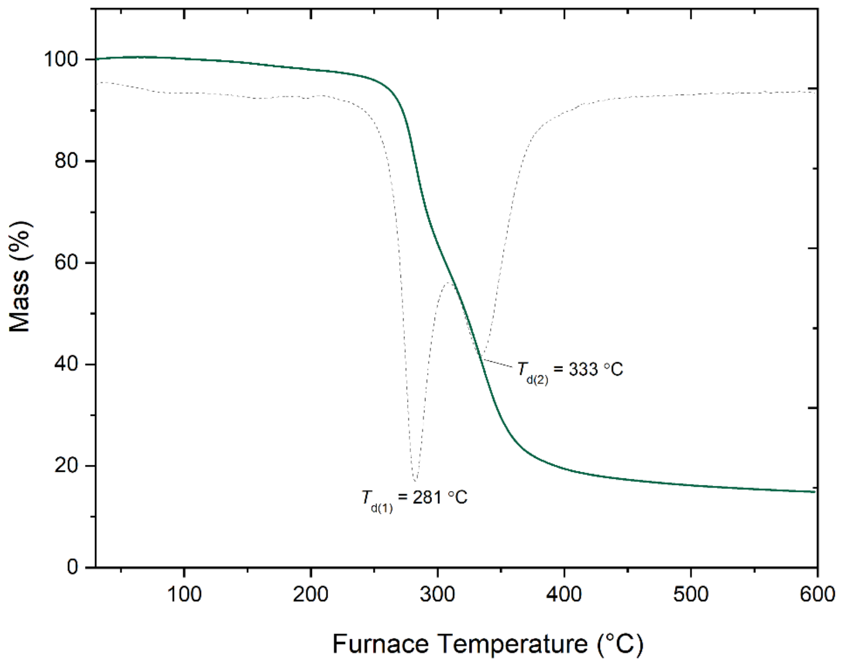

Figure S10.1: TGA trace showing the mass loss of poly(**1**-EDT) (M_n = 6.4 kg mol^{-1}) up to 600 °C. $\boldsymbol{T}_{\mathbf{d,5\%}}$ = 254 °C, $\boldsymbol{T}_{\mathbf{d,max}}$ = 281 and 333 °C.

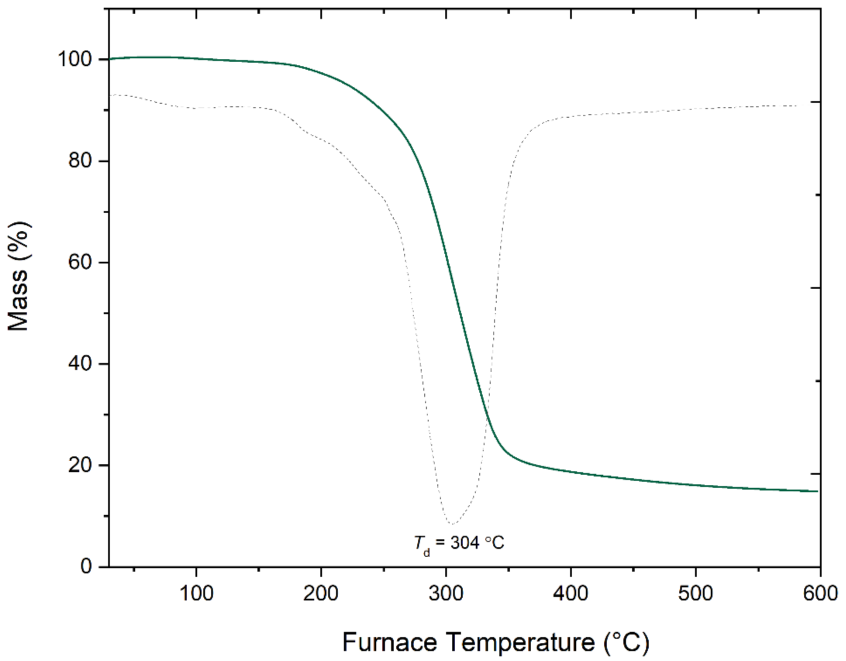

Figure S10.2: TGA trace showing the mass loss of poly(**2**-EDT) (M_n = 13.6 kg mol^{-1}) up to 600 °C. $\boldsymbol{T}_{\mathbf{d,5\%}}$ = 216 °C, $\boldsymbol{T}_{\mathbf{d,max}}$ = 304 °C.

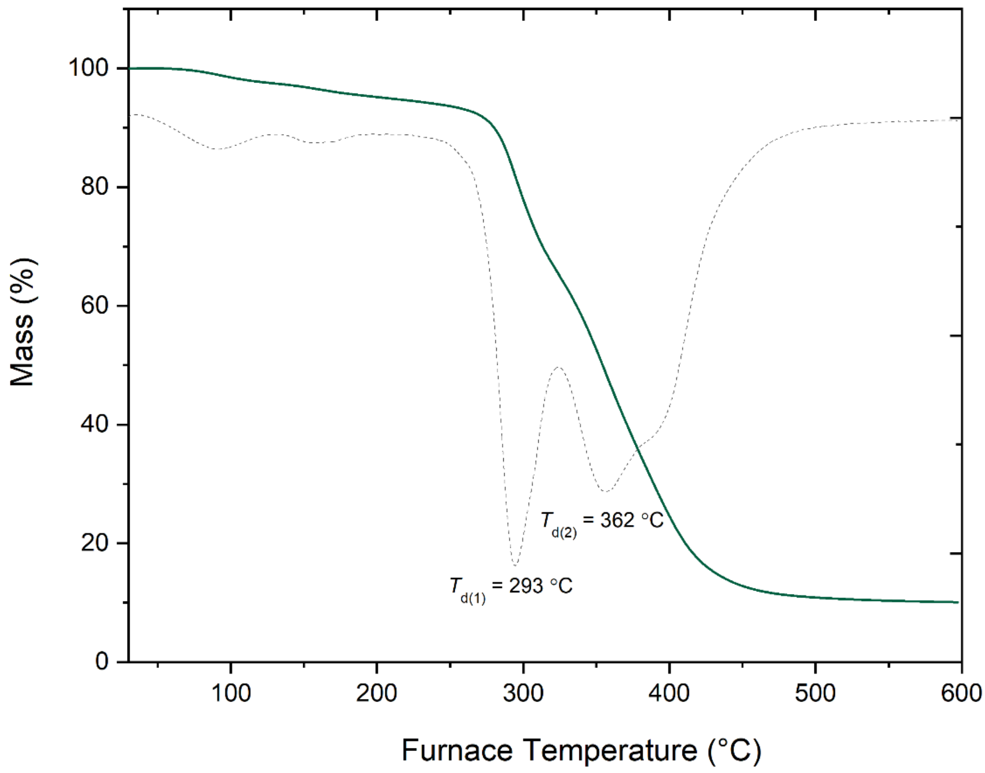

Figure S10.3: TGA trace showing the mass loss of poly(**3**-EDT) (M_n = 12.3 kg mol^{-1}) up to 600 °C. $\boldsymbol{T}_{\mathbf{d,5\%}}$ = 281 °C, $\boldsymbol{T}_{\mathbf{d,max}}$ = 293 and 362 °C.

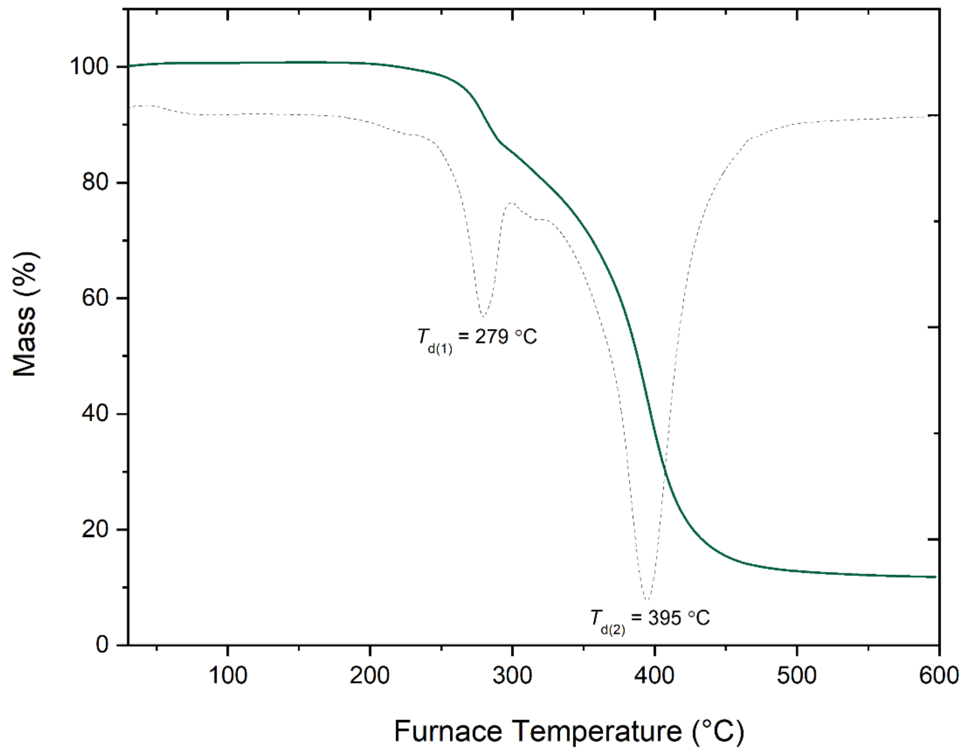

Figure S10.4: TGA trace showing the mass loss of poly(**3**-ODT) (M_n = 12.5 kg mol^{-1}) up to 600 °C. $\boldsymbol{T}_{\mathbf{d,5\%}}$ = 269 °C, $\boldsymbol{T}_{\mathbf{d,max}}$ = 279 and 395 °C.

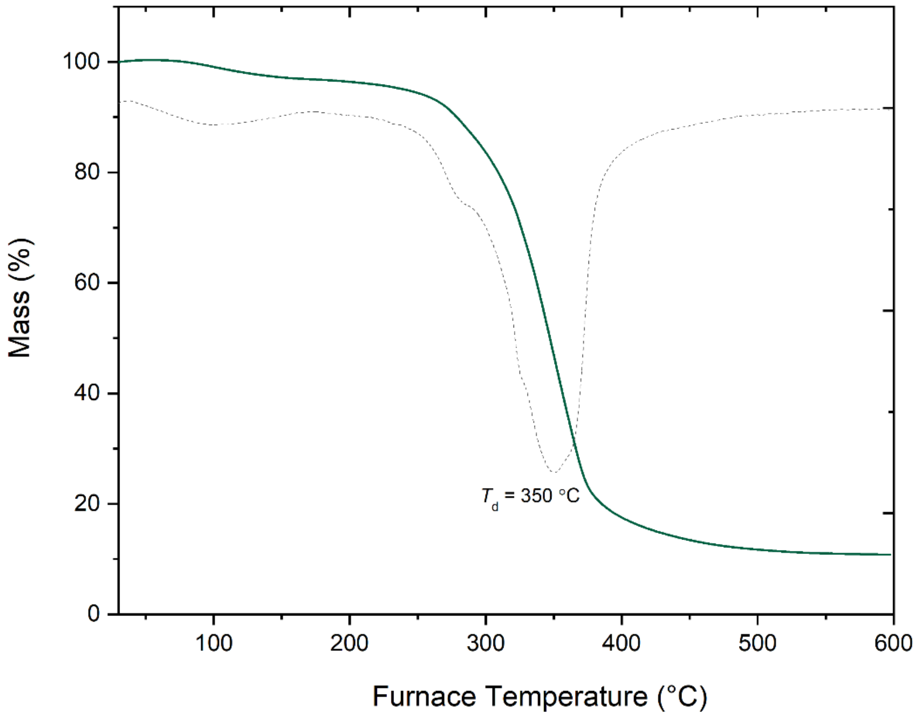

Figure S10.5: TGA trace showing the mass loss of poly(**4**-EDT) (M_n = 10.8 kg mol^{-1}) up to 600 °C. $\boldsymbol{T}_{\mathbf{d,5\%}}$ = 271 °C, $\boldsymbol{T}_{\mathbf{d,max}}$ = 350 °C.

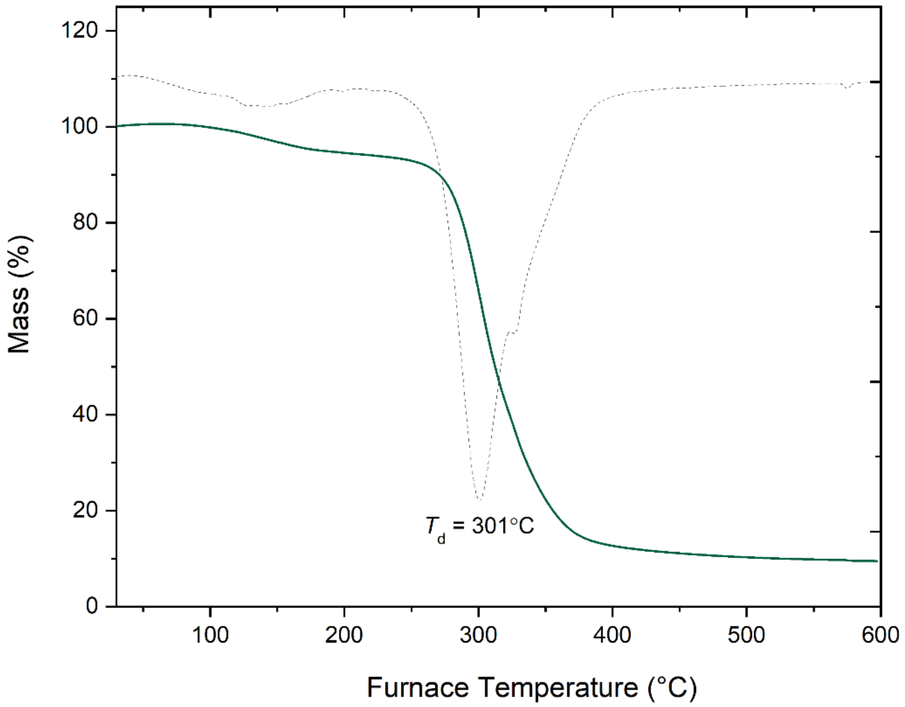

Figure S10.6: TGA trace showing the mass loss of poly(**5**-EDT) (M_n = 3.7 kg mol^{-1}) up to 600 °C. $\boldsymbol{T}_{\mathbf{d,5\%}}$ = 141 °C, $\boldsymbol{T}_{\mathbf{d,max}}$ = 301 °C.

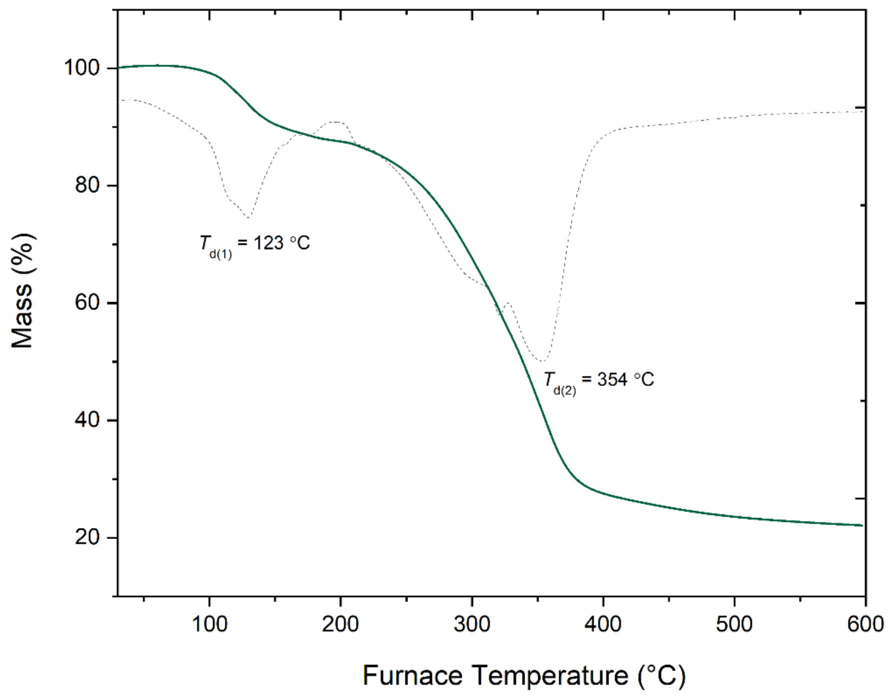

Figure S10.7: TGA trace showing the mass loss of poly(**6**-EDT) (M_n = 2.5 kg mol^{-1}) up to 600 °C. $\boldsymbol{T}_{\mathbf{d,5\%}}$ = 123 °C, $\boldsymbol{T}_{\mathbf{d,max}}$ = 354 °C.

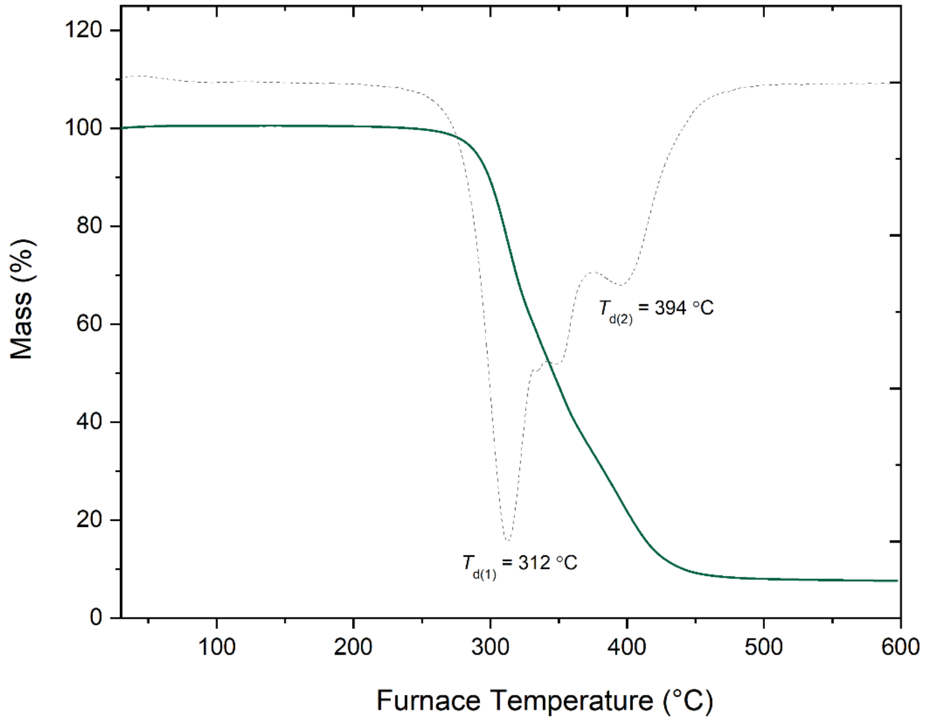

Figure S10.8: TGA trace showing the mass loss of poly(**7**-EDT) (M_n = 14.7 kg mol^{-1}) up to 600 °C. $\boldsymbol{T}_{\mathbf{d,5\%}}$ = 288 °C, $\boldsymbol{T}_{\mathbf{d,max}}$ = 312 and 394 °C.

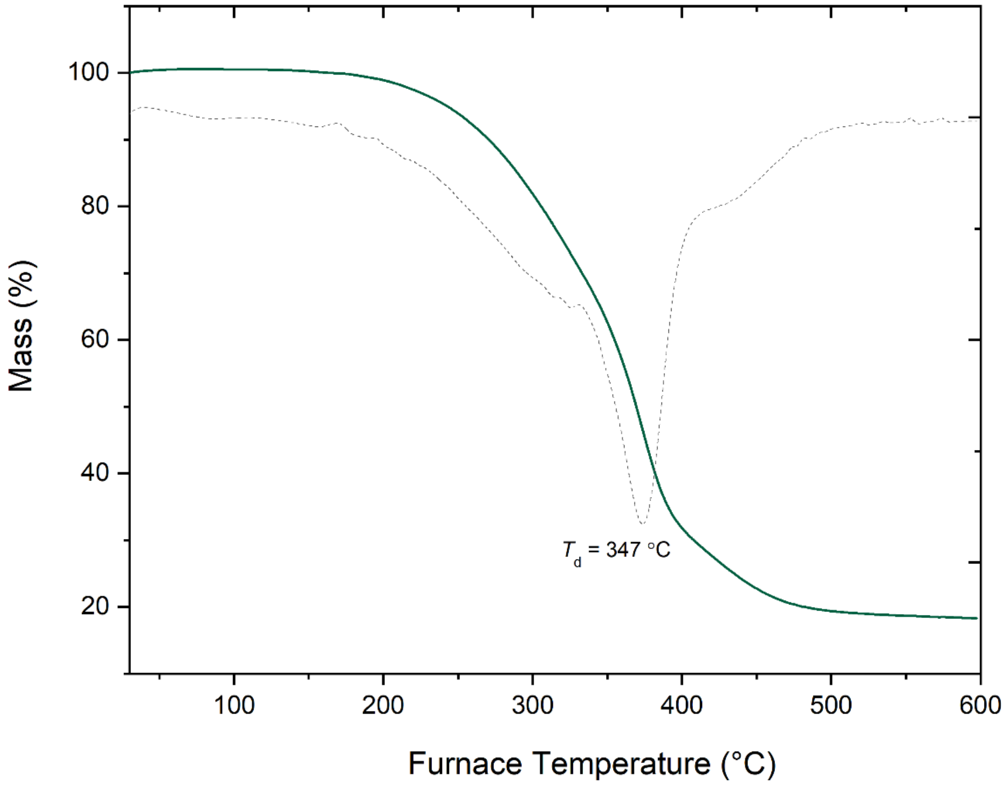

Figure S10.9: TGA trace showing the mass loss of poly(**8**-EDT) (M_n unknown) up to 600 °C. $\boldsymbol{T}_{\mathbf{d,5\%}}$ = 239 °C, $\boldsymbol{T}_{\mathbf{d,max}}$ = 374 °C.

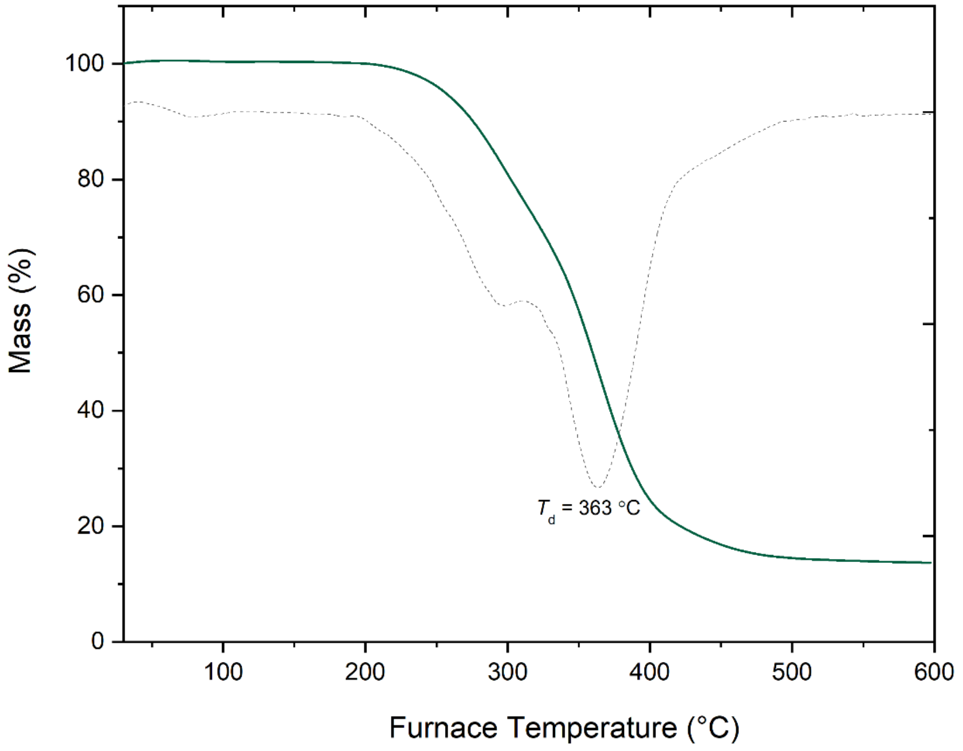

Figure S10.10: TGA trace showing the mass loss of poly(**4**-EDT) (M_n unknown) up to 600 °C. $\boldsymbol{T}_{\mathbf{d,5\%}}$ = 254 °C, $\boldsymbol{T}_{\mathbf{d,max}}$ = 363 °C.

Representative SPEs

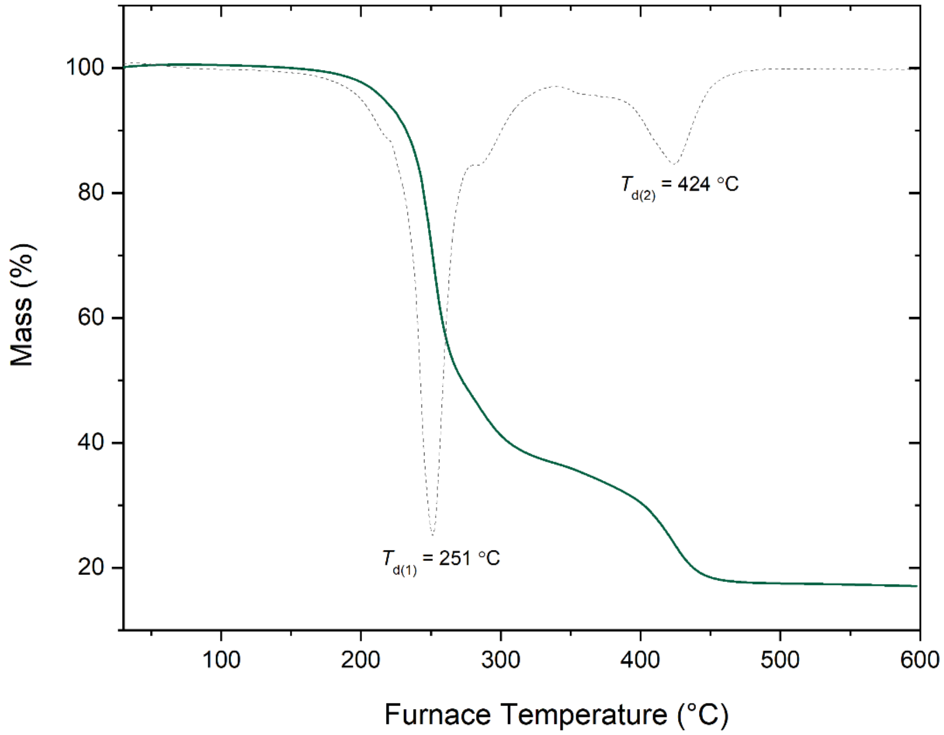

Figure S10.11: TGA trace showing the mass loss of a representative covalently cross-linked SPE based on monomer **1** with EDT (**SPE-1**, **Table 2**) up to 600 °C.
$\boldsymbol{T}_{\mathbf{d,5\%}}$ = 215 °C, $\boldsymbol{T}_{\mathbf{d,max}}$ = 251 and 424 °C.

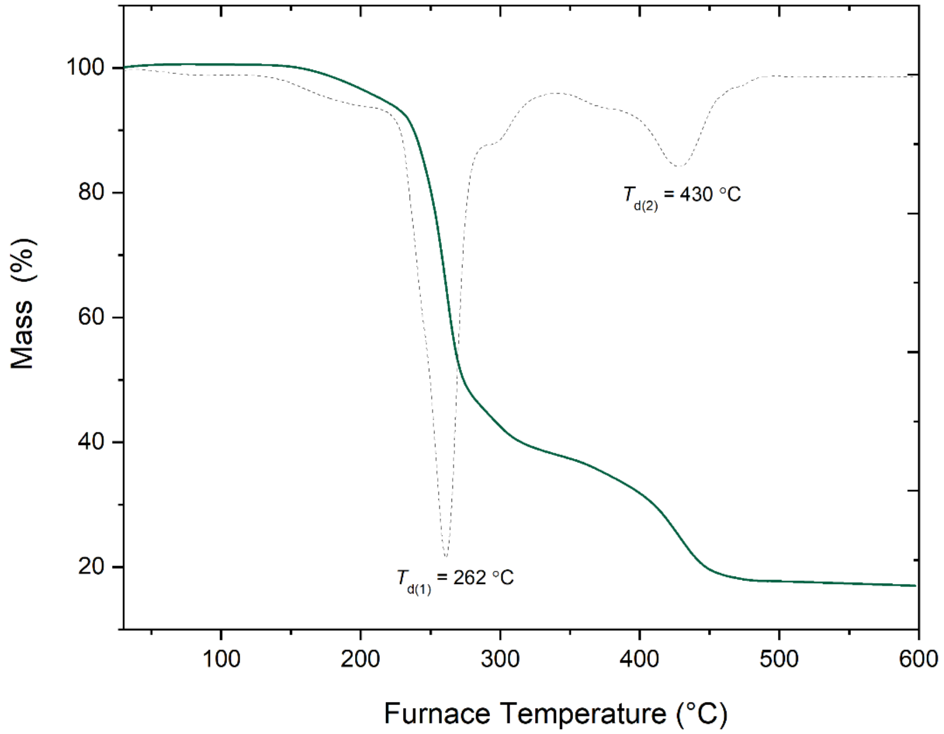

Figure S10.12: TGA trace showing the mass loss of a representative covalently cross-linked SPE based on monomer **2** with EDT (**SPE-2**, **Table 2**) up to 600 °C.
$\boldsymbol{T}_{\mathbf{d,5\%}}$ = 205 °C, $\boldsymbol{T}_{\mathbf{d,max}}$ = 262 and 430 °C.

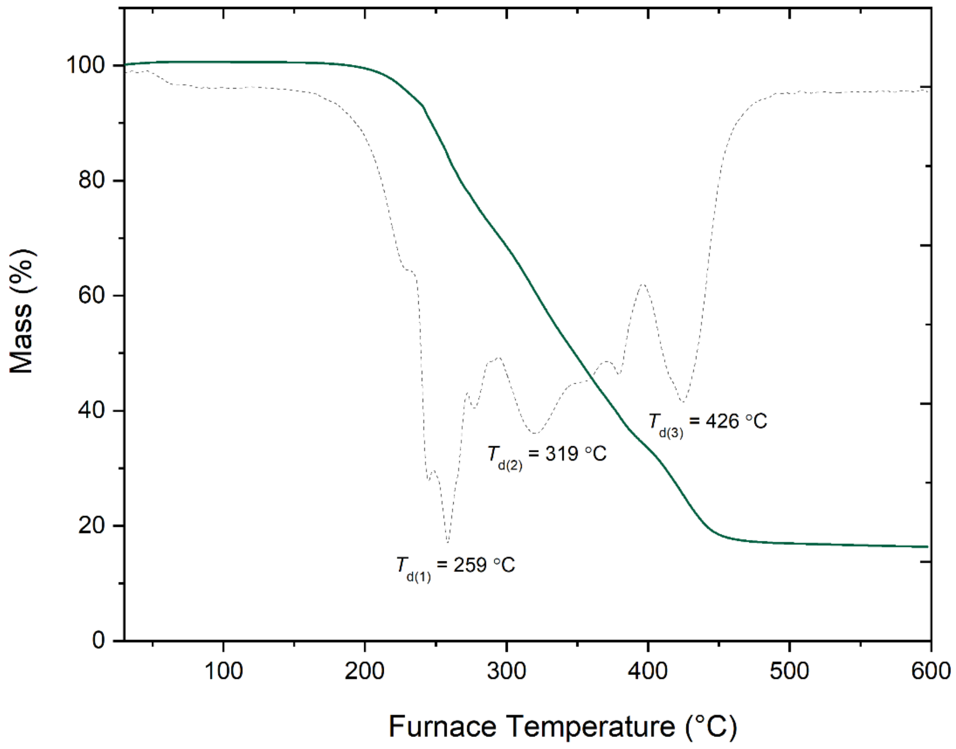

Figure S10.13: TGA trace showing the mass loss of a representative covalently cross-linked SPE based on monomer **3** with EDT (**SPE-3a**, **Table 2**) up to 600 °C. $\boldsymbol{T_{d,5\%}}$ = 229 °C, $\boldsymbol{T_{d,max}}$ = 259, 319 and 426 °C.

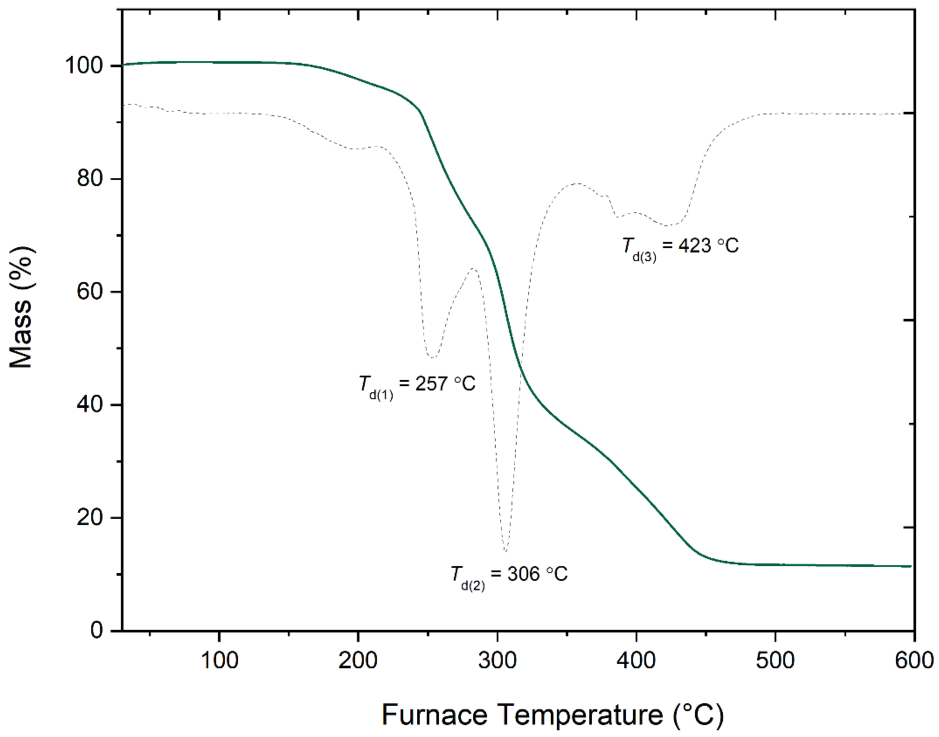

Figure S10.14: TGA trace showing the mass loss of a representative covalently cross-linked SPE based on monomer **4** with EDT (**SPE-4**, **Table 2**) up to 600 °C. $\boldsymbol{T_{d,5\%}}$ = 221 °C, $\boldsymbol{T_{d,max}}$ = 257, 306 and 423 °C.

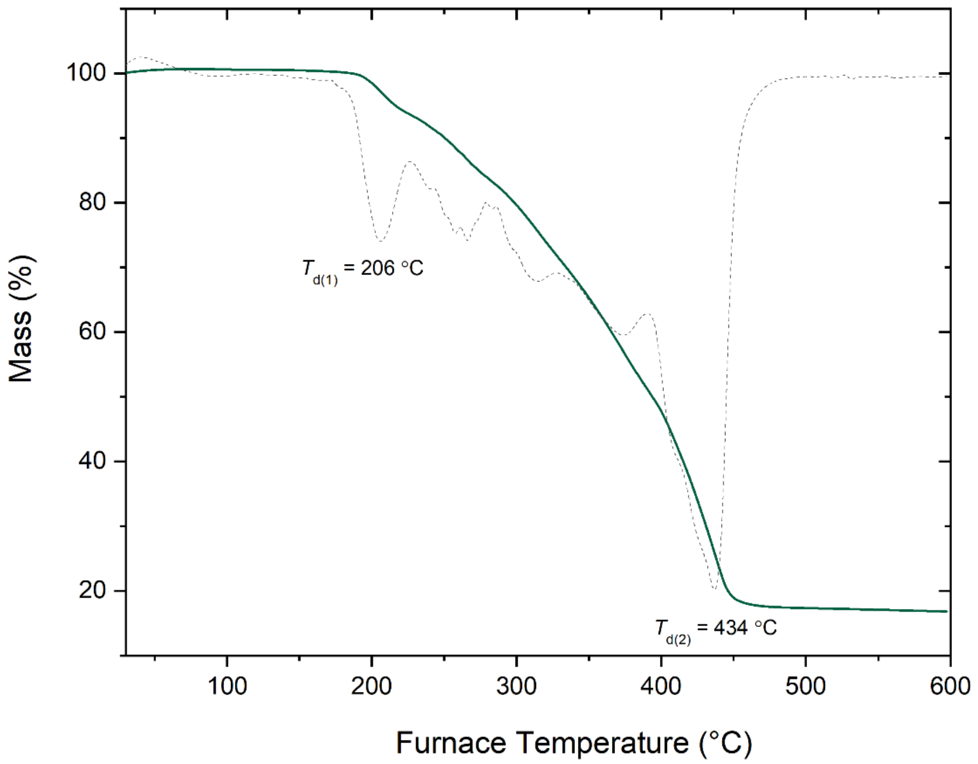

Figure S10.15: TGA trace showing the mass loss of a representative semi-crystalline SPE based on monomer **3** with EDT and ODT up to 600 °C.
$\boldsymbol{T_{d,5\%}}$ = 206 °C, $\boldsymbol{T_{d,max}}$ = 206 and 434 °C.

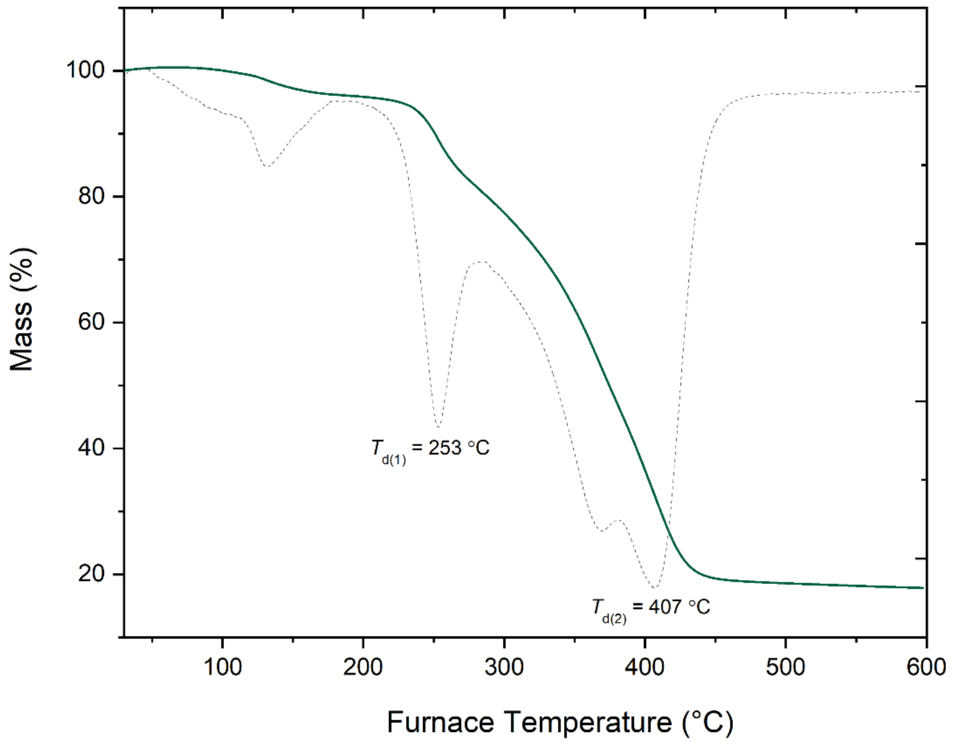

Figure S10.16: TGA trace showing the mass loss of a representative nucleoside-based SPE based on monomers **7** & **8** with EDT (**SPE-7/8d**, **Table 5**) up to 600 °C.
$\boldsymbol{T_{d,5\%}}$ = 249 °C, $\boldsymbol{T_{d,max}}$ = 253 and 407 °C.

11. Electrochemical impedance spectroscopy (EIS)

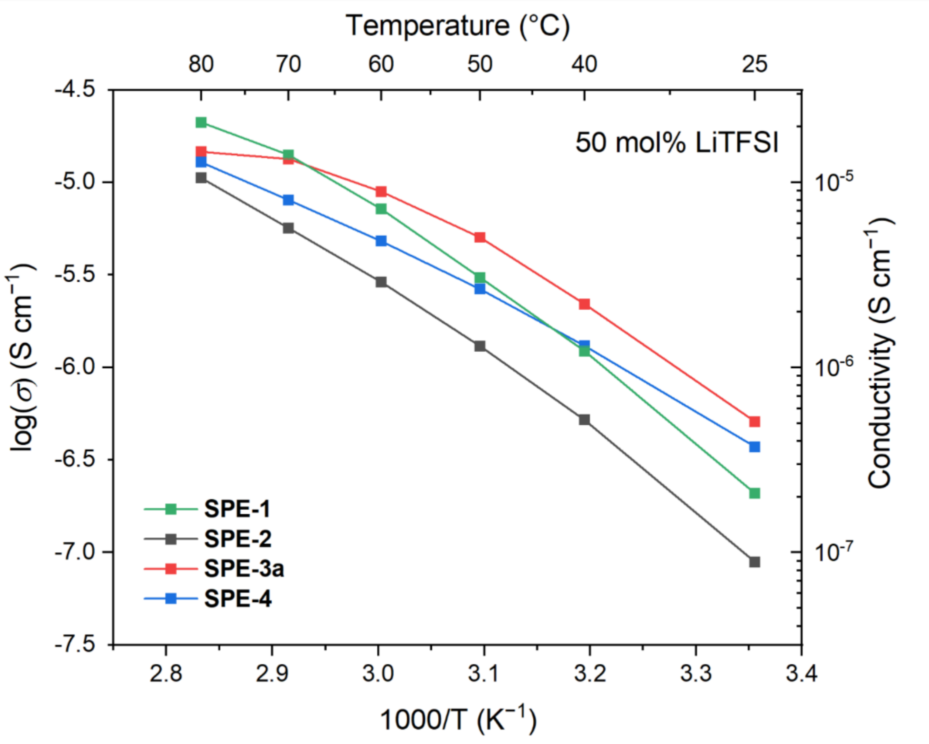

Figure S11.1: Plots of the temperature dependence of the ionic conductivities of **SPEs 1−4** (based on monomers **1–4**).

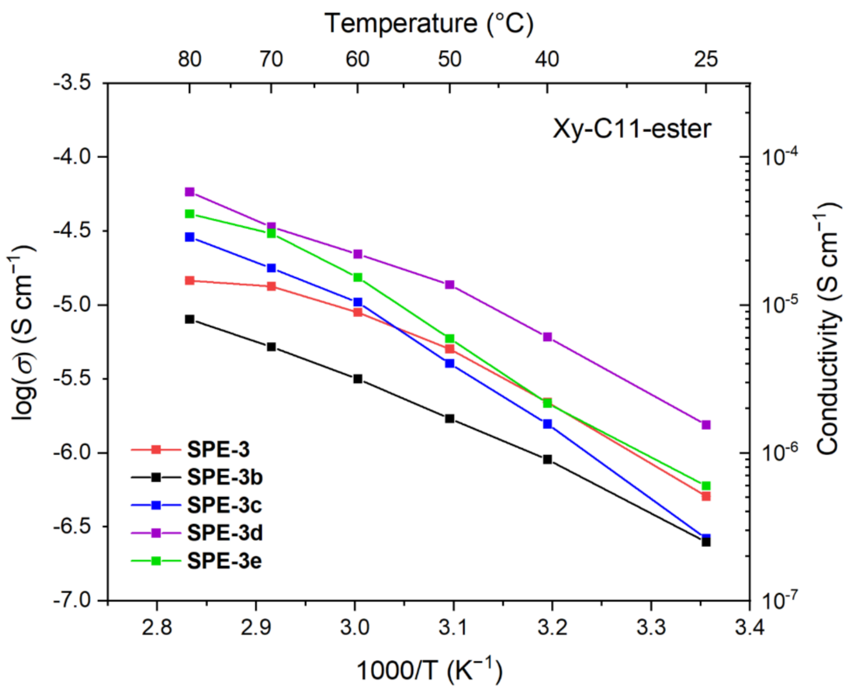

Figure S11.2: Plots of the temperature dependence of the ionic conductivities of **SPEs 1−4** (based on monomer **3**).

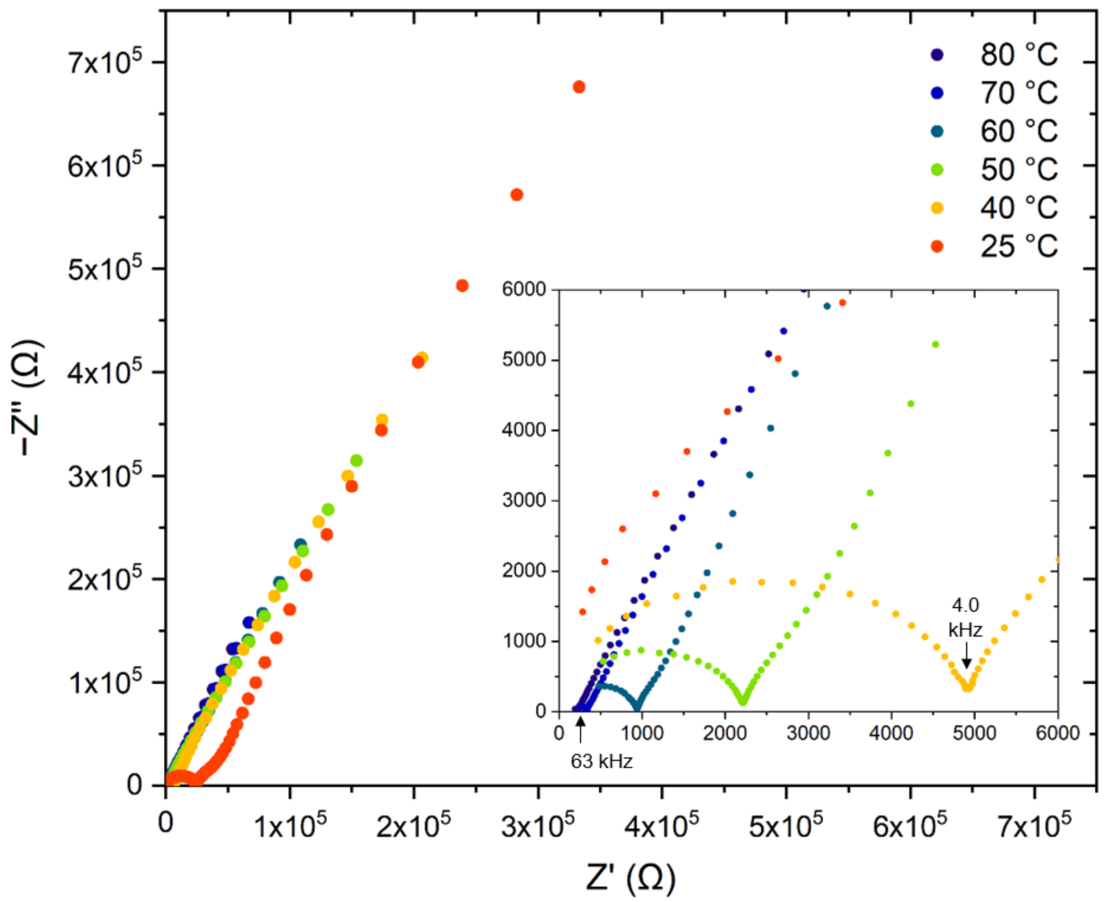

Figure S11.3: Representative Nyquist plots used for the determination of ionic conductivity of **SPE-7/8d** (Thy/Ade (9:1), 100 mol% LiTFSI). Inlaid plot is zoomed in to the high frequency region.

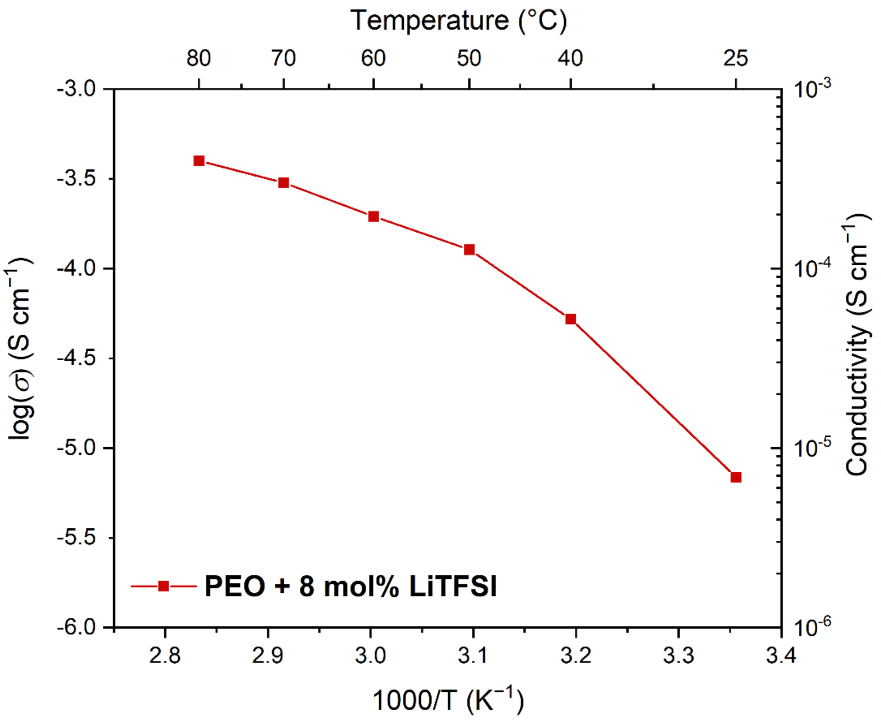

Figure S11.4: Temperature dependence of the ionic conductivity of a SPE prepared with a commercial sample of PEO (M_n = 100 kg mol^{-1}, T_g = −36 °C, T_m = 44 °C).

12. Linear Sweep Voltammetry (LSV)

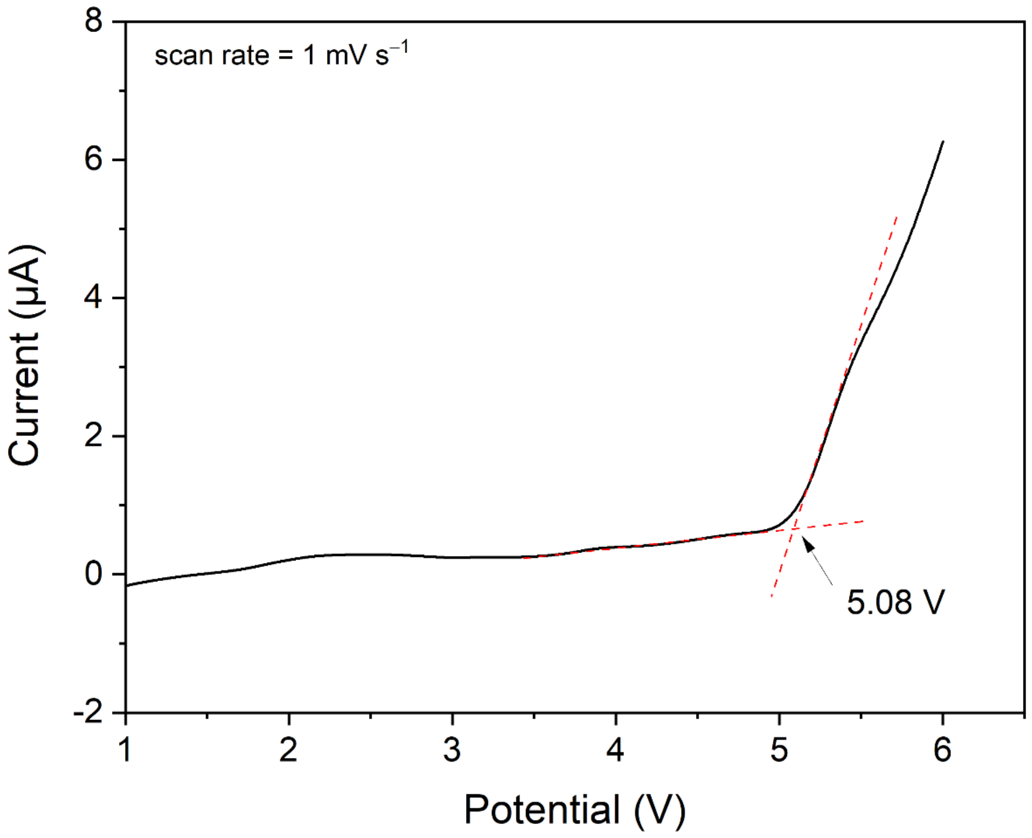

Figure S12.1: Linear sweep voltammograms of **SPE-3d** (measured at 60 °C) with a scan rate of 1 mV s^{-1} from 0−6 V (*vs.* Li/Li^{+}).

13. Transference Number

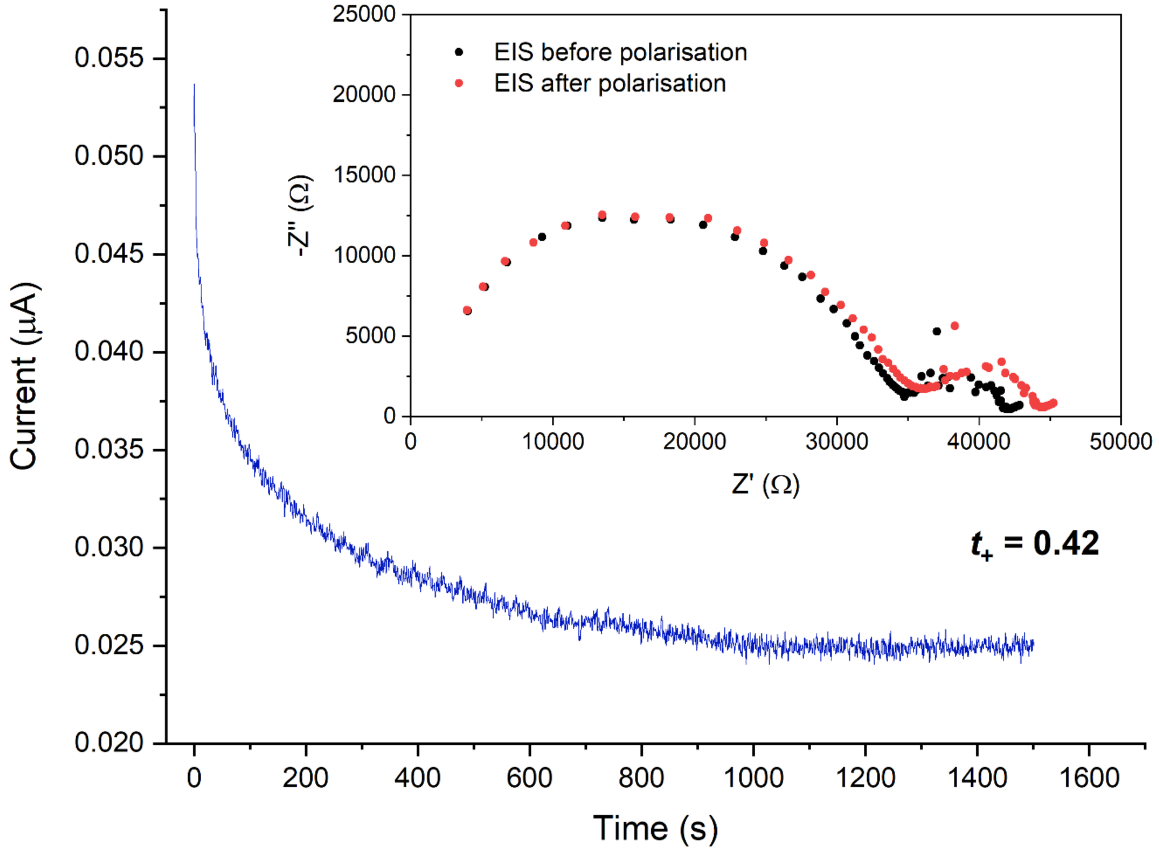

Figure S13.1: Representative chronoamperometry and Nyquist plot (inlaid) obtained for the determination of t_+ for **SPE-3d**. A 10 mV applied polarization voltage was used for the chronoamperometry in a symmetric Li||SPE||Li cell at 60 °C. The Nyquist plot was obtained by EIS recorded in the frequency range of 0.1 Hz to 1 MHz.

14. Size Exclusion Chromatography (SEC)

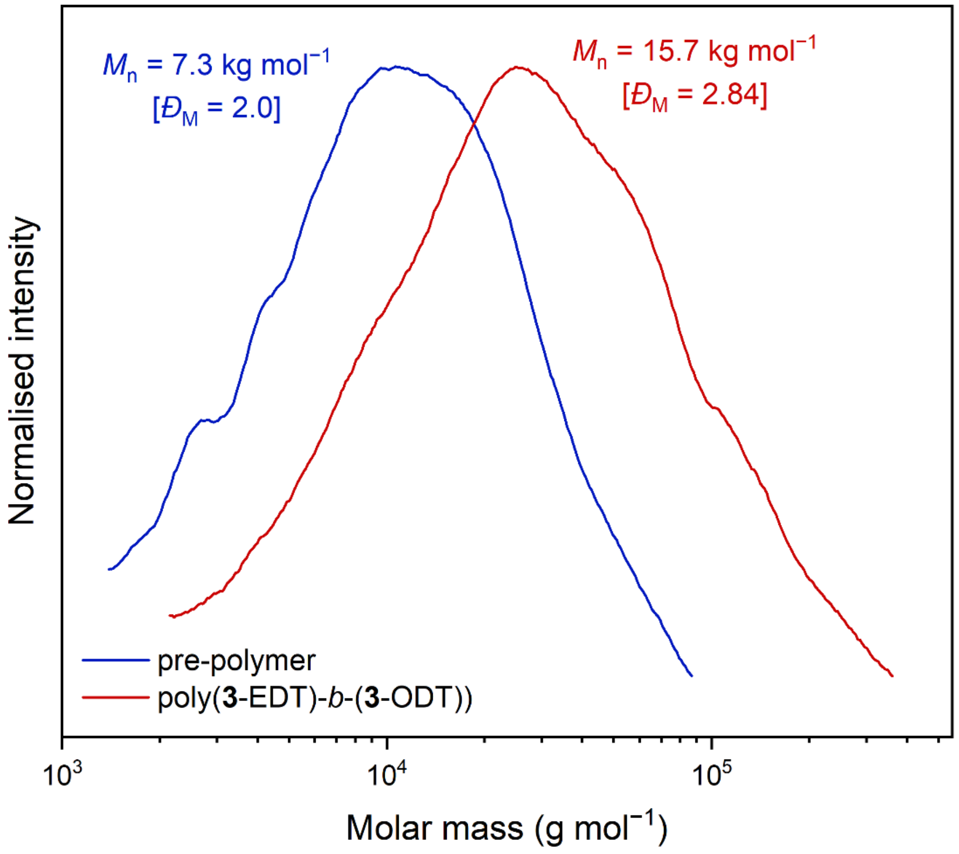

Figure S14.1: Overlaid molar mass distribution chart of poly((**3**-EDT)-*b*-(**3**-ODT)) (Table 3, entry 2) and the pre-formed **3**-EDT block obtained by SEC analysis in THF (Polystyrene calibration standards).

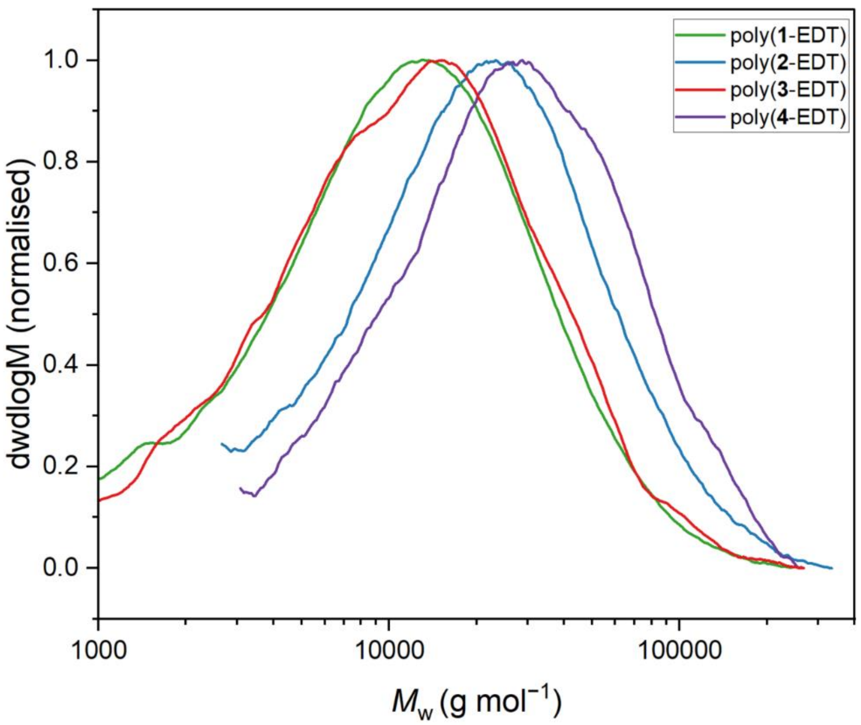

Figure S14.2: Overlaid molar mass distribution charts of xylose-based co-polymers with EDT.

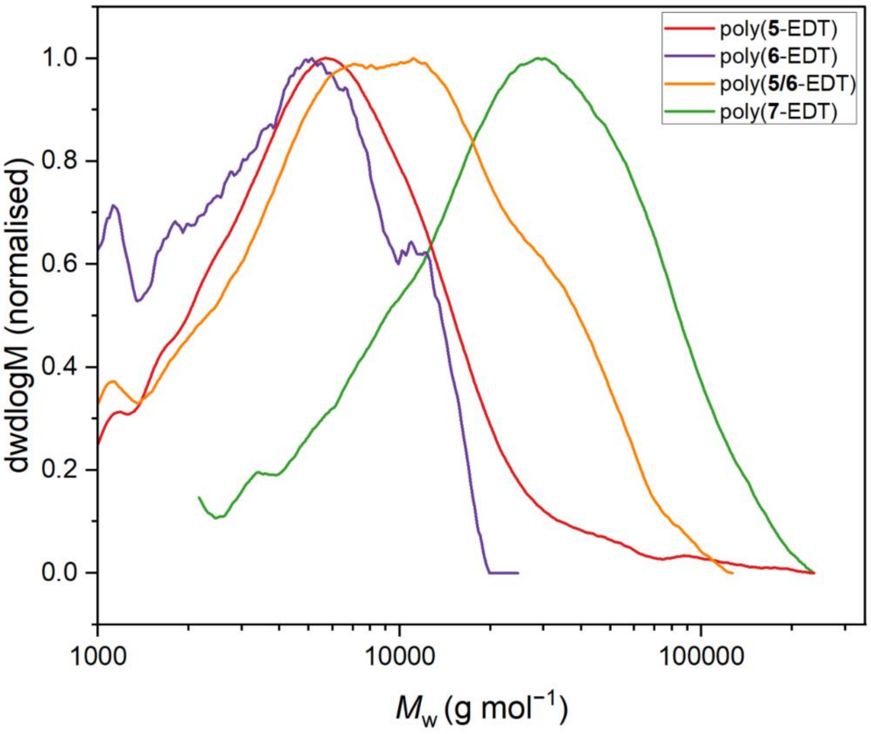

Figure S14.3: Overlaid molar mass distribution charts of nucleoside-based co-polymers with EDT.

15. Electrochemical Stripping/Plating Experiments

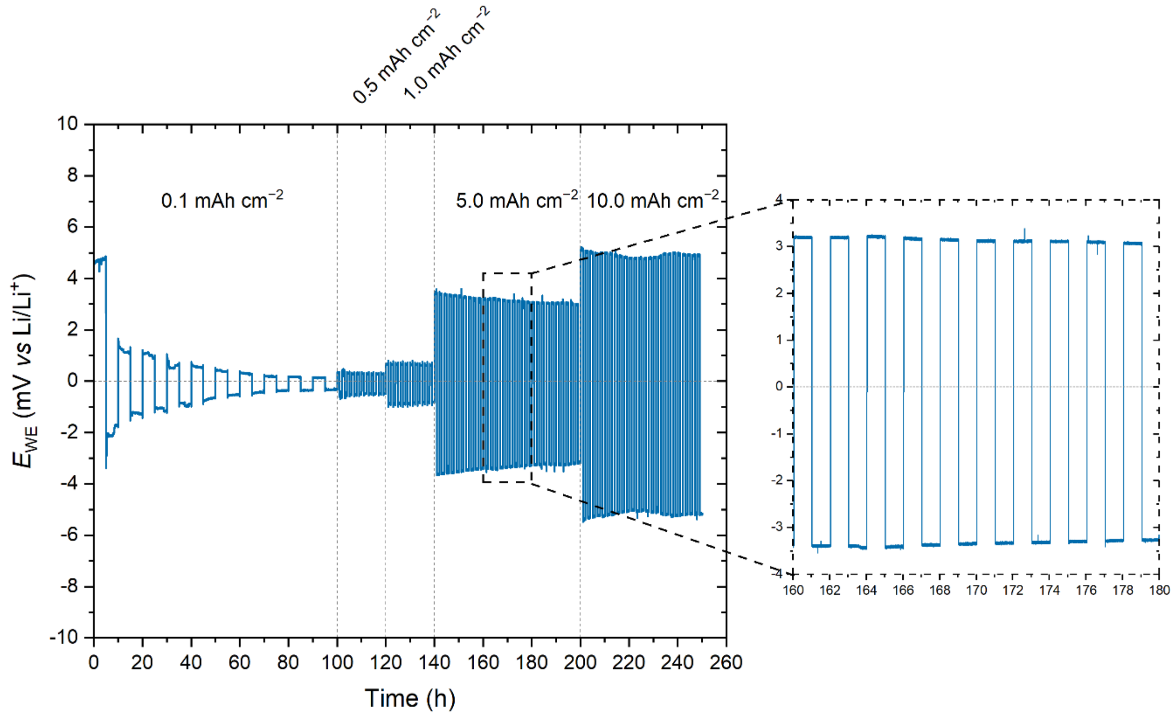

Figure S15.1: Cycling performance of **SPE-7/8d** in a short-circuited symmetric Li//Li cell at 60 °C with half-cycle capacities increasing from 0.1 mAh cm^{-2} to 10.0 mAh cm^{-2}.

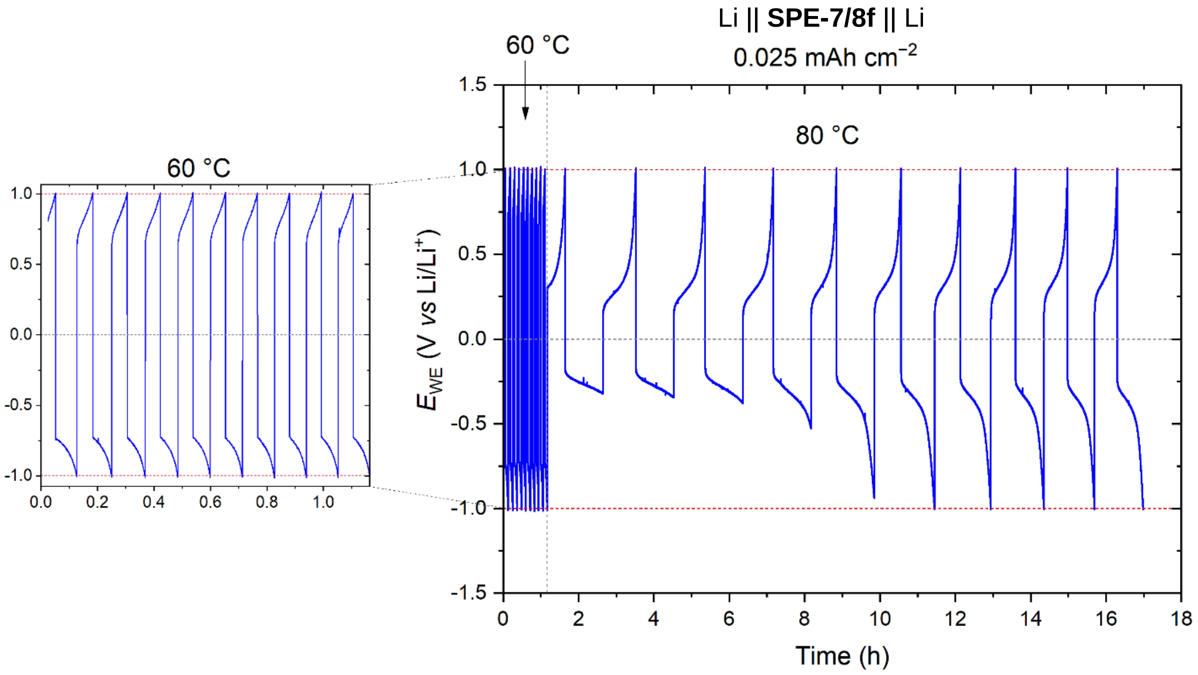

Figure S15.2: Chart showing the cycling performances of **SPE-7/8f** in a symmetric Li//Li cell at 60 °C and 80 °C with one hour half-cycles at a current density of 0.025 mA cm^{-2}.

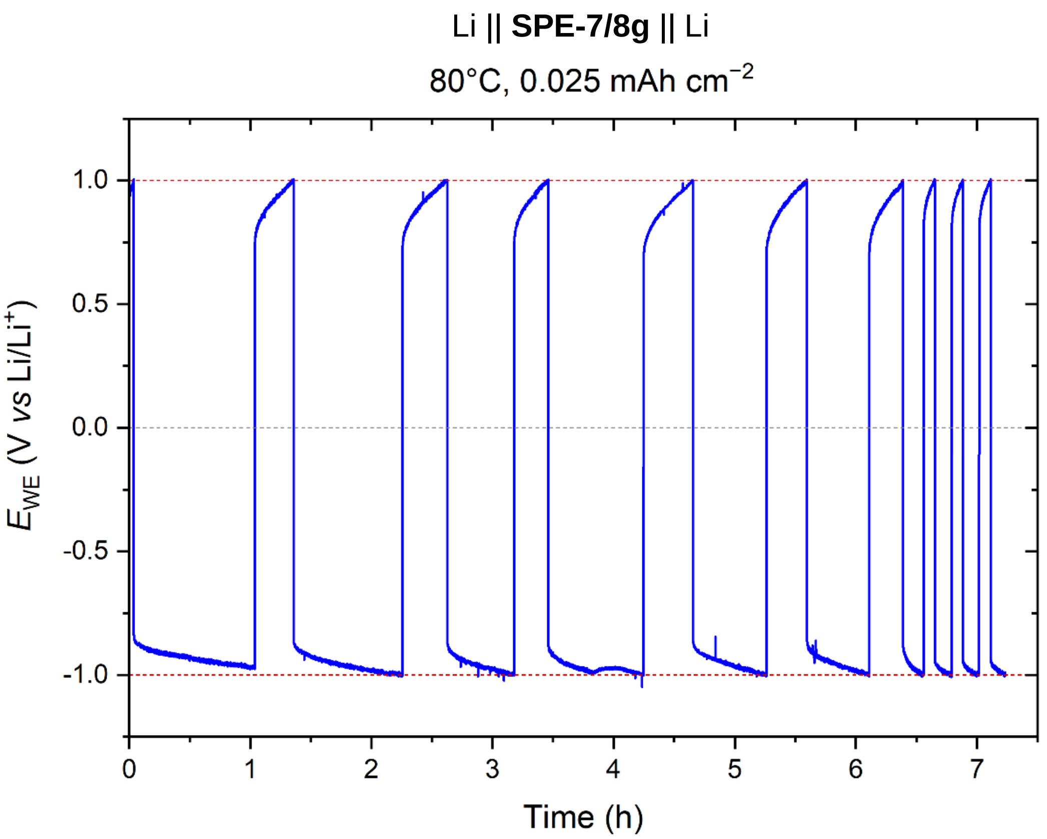

Figure S115.3: Chart showing the cycling performances of **SPE-7/8g** in a symmetric Li//Li cell at 60 °C and 80 °C with one hour half-cycles at a current density of 0.025 mA cm^{-2}.

16. Rheological Self-Healing Experiments

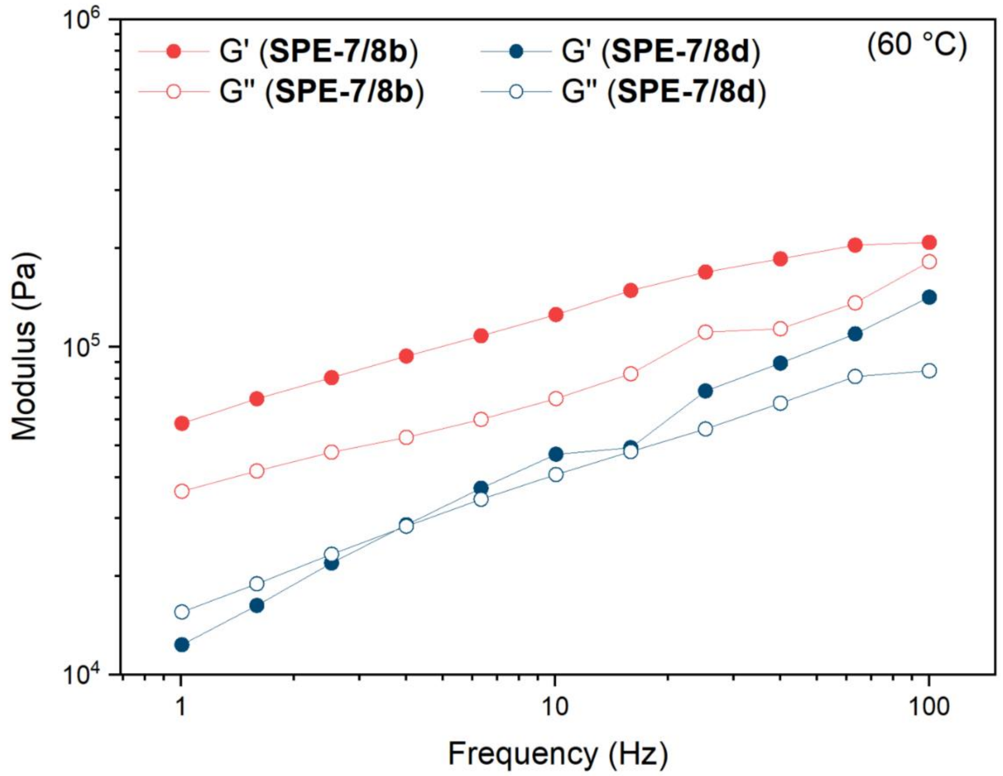

Figure S16.1: Dynamic oscillating frequency sweep of **SPE-7/8b** and **SPE-7/8d** from 1−100 Hz at 60 °C.

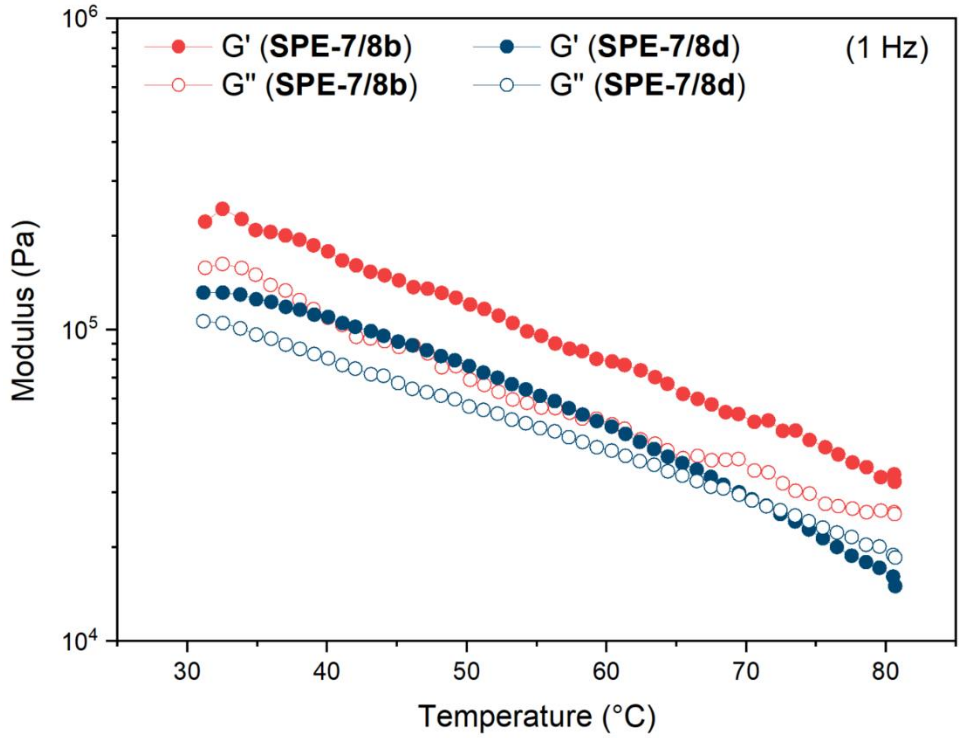

Figure S16.2: Temperature sweep of **SPE-7/8b** and **SPE-7/8d** from 30−80 °C with an oscillating frequency of 1 Hz.

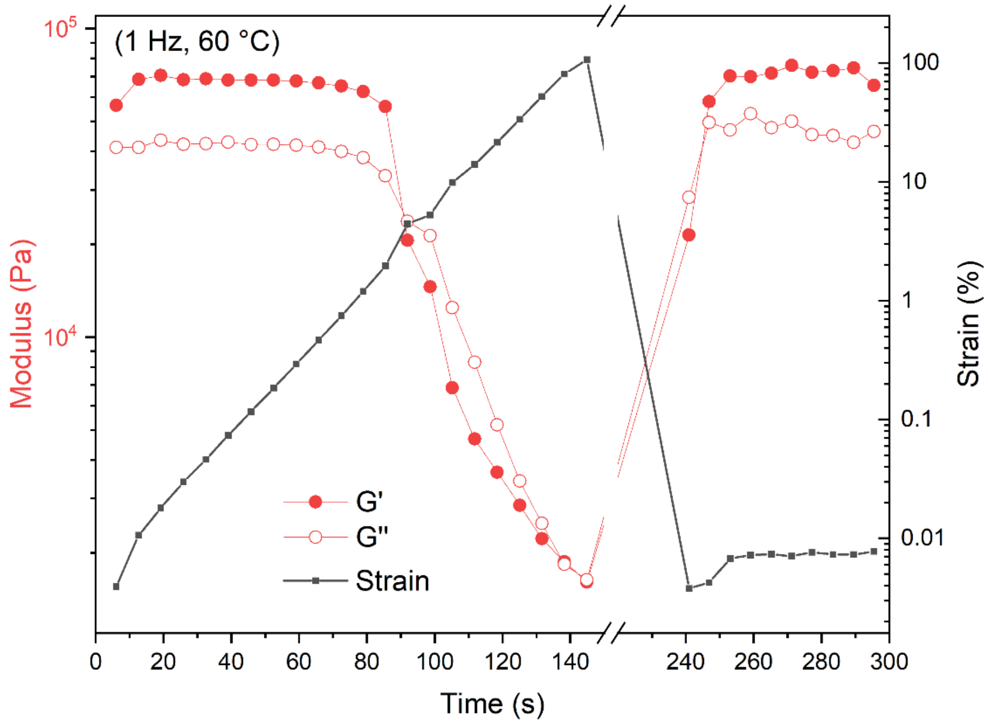

Figure S16.3: Single strain ramp rheological measurement for **SPE-7/8b** measured at 60 °C with an oscillating frequency of 1 Hz.

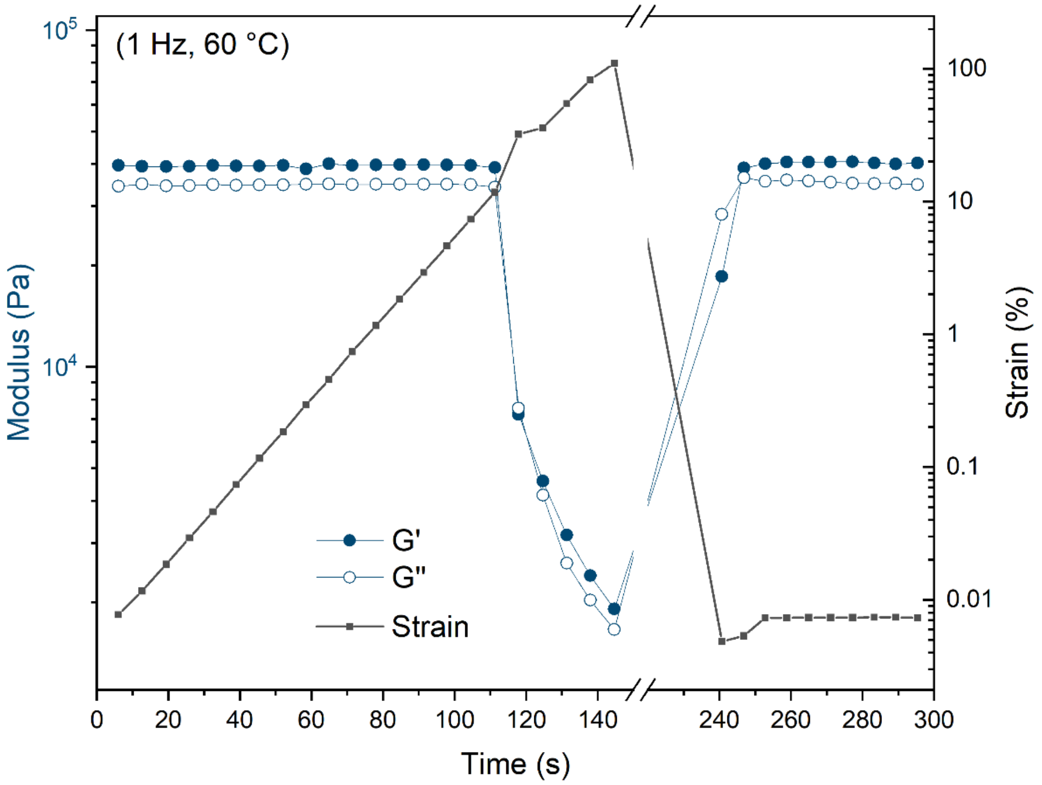

Figure S16.4: Single strain ramp rheological measurement for **SPE-7/8d** measured at 60 °C with an oscillating frequency of 1 Hz.

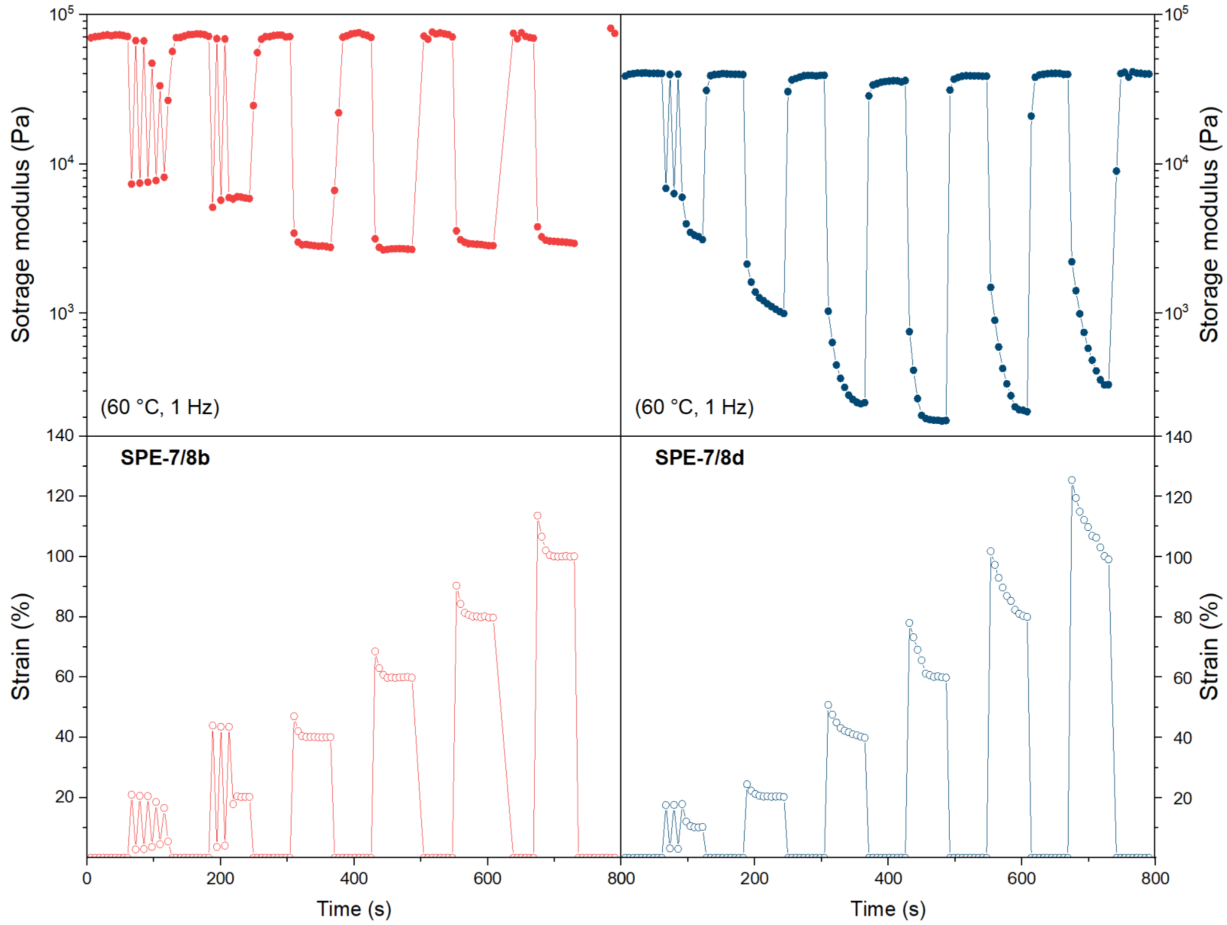

Figure S16.5: Charts showing the rheological self-healing behaviour of **SPE-7/8b** and **SPE-7/8d** with sequential strain steps measured at 60 °C with an oscillating frequency of 1 Hz. The top charts show the measured storage modulus of each material in response to the applied strain which is shown in the bottom charts. The 90 second rest period at the end of each elevated strain segment is omitted for clarity but was performed to allow time for the cross-links to reform.

17. References

1. Piccini, M.; Lightfoot, J.; Dominguez, B. C.; Buchard, A. Xylose-Based Polyethers and Polyesters Via ADMET Polymerization toward Polyethylene-Like Materials. *ACS Appl. Polym. Mater.* **2021**, *3* (11), 5870–5881. https://doi.org/10.1021/acsapm.1c01095
2. Oshinowo, M.; Runge, J. R.; Piccini, M.; Marken, F.; Buchard, A. Crosslinked Xylose-Based Polyester as a Bio-Derived and Degradable Solid Polymer Electrolyte for Li + -Ion Conduction. *J. Mater. Chem. A* **2022**, *10* (12), 6796–6808. https://doi.org/10.1039/D1TA10111K.
3. Evans, J.; Vincent, C. A.; Bruce P. G. *Polymer* **1987**, *28* (13), 2324–2328. https://doi.org/10.1016/0032-3861(87)90394-6
